# Evaluating Large Language Models and Retrieval-Augmented Generation Enhancement for Delivering Guideline-Adherent Nutrition Information for Cardiovascular Disease Prevention: Cross-Sectional Study

**DOI:** 10.2196/78625

**Published:** 2025-10-07

**Authors:** Vijaya Parameswaran, Jenna Bernard, Alec Bernard, Neil Deo, Sean Tsung, Kalle Lyytinen, Christopher Sharp, Fatima Rodriguez, David J Maron, Rajesh Dash

**Affiliations:** 1 School of Medicine Stanford University Palo Alto, CA United States; 2 Tufts University Boston, MA United States; 3 University of Illinois Urbana-Champaign Champaign, IL United States; 4 Stanford University Palo Alto, CA United States; 5 Case Western Reserve University Cleveland, OH United States

**Keywords:** large language models, cardiovascular dietary guidelines, qualitative evaluation, retrieval-augmented generation, artificial intelligence

## Abstract

**Background:**

Cardiovascular disease (CVD) remains the leading cause of death worldwide, yet many web-based sources on cardiovascular (CV) health are inaccessible. Large language models (LLMs) are increasingly used for health-related inquiries and offer an opportunity to produce accessible and scalable CV health information. However, because these models are trained on heterogeneous data, including unverified user-generated content, the quality and reliability of food and nutrition information on CVD prevention remain uncertain. Recent studies have examined LLM use in various health care applications, but their effectiveness for providing nutrition information remains understudied. Although retrieval-augmented generation (RAG) frameworks have been shown to enhance LLM consistency and accuracy, their use in delivering nutrition information for CVD prevention requires further evaluation.

**Objective:**

To evaluate the effectiveness of off-the-shelf and RAG-enhanced LLMs in delivering guideline-adherent nutrition information for CVD prevention, we assessed 3 off-the-shelf models (ChatGPT-4o, Perplexity, and Llama 3-70B) and a Llama 3-70B+RAG model.

**Methods:**

We curated 30 nutrition questions that comprehensively addressed CVD prevention. These were approved by a registered dietitian providing preventive cardiology services at an academic medical center and were posed 3 times to each model. We developed a 15,074-word knowledge bank incorporating the American Heart Association’s 2021 dietary guidelines and related website content to enhance Meta’s Llama 3-70B model using RAG. The model received this and a few-shot prompt as context, included citations in a *Context Source* section, and used vector similarity to align responses with guideline content, with the temperature parameter set to 0.5 to enhance consistency. Model responses were evaluated by 3 expert reviewers against benchmark CV guidelines for appropriateness, reliability, readability, harm, and guideline adherence. Mean scores were compared using ANOVA, with statistical significance set at *P*<.05. Interrater agreement was measured using the Cohen κ coefficient, and readability was estimated using the Flesch-Kincaid readability score.

**Results:**

The Llama 3+RAG model scored higher than the Perplexity, GPT-4o, and Llama 3 models on reliability, appropriateness, guideline adherence, and readability and showed no harm. The Cohen κ coefficient (κ>70%; *P*<.001) indicated high reviewer agreement.

**Conclusions:**

The Llama 3+RAG model outperformed the off-the-shelf models across all measures with no evidence of harm, although the responses were less readable due to technical language. The off-the-shelf models scored lower on all measures and produced some harmful responses. These findings highlight the limitations of off-the-shelf models and demonstrate that RAG system integration can enhance LLM performance in delivering evidence-based dietary information.

## Introduction

### Background

Large language models (LLMs) and generative artificial intelligence (AI) systems are increasingly used in health care [1] and offer an opportunity to produce accessible and scalable educational tools on cardiovascular (CV) health, aligning with the American Heart Association’s (AHA) 2020 impact goals to enhance health literacy [2]. This is especially important as many web-based educational materials on CV disease (CVD) remain inaccessible [3]. However, because LLMs are trained on vast and heterogeneous data, including internet-based unverified user-generated content, the quality and reliability of the food and nutrition information provided, particularly as it pertains to CV health, are uncertain. Retrieval-augmented generation (RAG) is a framework that enhances LLM performance by incorporating external knowledge retrieval mechanisms to generate accurate and contextually relevant responses grounded in specific reference materials [4], and RAG-enhanced models have shown higher consistency and accuracy [5,6].

### Prior Research and This Study

Recent studies have examined the use of LLMs in mental health, online health information seeking, exercise recommendations, and simplifying medical information in various contexts [7-15], but their use and effectiveness regarding providing nutrition information remain understudied and lack benchmark evaluations. Studies have revealed mixed capabilities, demonstrating adequate guideline adherence in basic nutrition recommendations but limitations in specialized applications such as medical nutrition therapy for chronic diseases, nutrient calculations, and accuracy when providing guidance across different languages [16-19]. These studies highlight both promising potential and concerning consistency issues when LLMs address clinical nutrition questions, particularly in specialized domains. Our work extends this emerging literature by systematically comparing multiple model architectures against CV nutrition guidelines using a comprehensive assessment framework. In this study, we enhanced a Llama 3 model with an RAG framework by grounding it in CV dietary guidelines. We then evaluated it against 3 off-the-shelf models (OpenAI’s ChatGPT-4o, Perplexity AI’s Perplexity, and Meta AI’s Llama 3-70B) to answer common nutrition questions in accordance with established CV dietary guidelines. Nutrition questions that comprehensively addressed CVD prevention were developed and reviewed by a registered dietitian specializing in preventive cardiology at an academic medical center. Responses to these questions were qualitatively assessed by expert reviewers for appropriateness, reliability, potential for harm, readability, and adherence to clinical guidelines.

## Methods

### Overview

**Figure 1:**
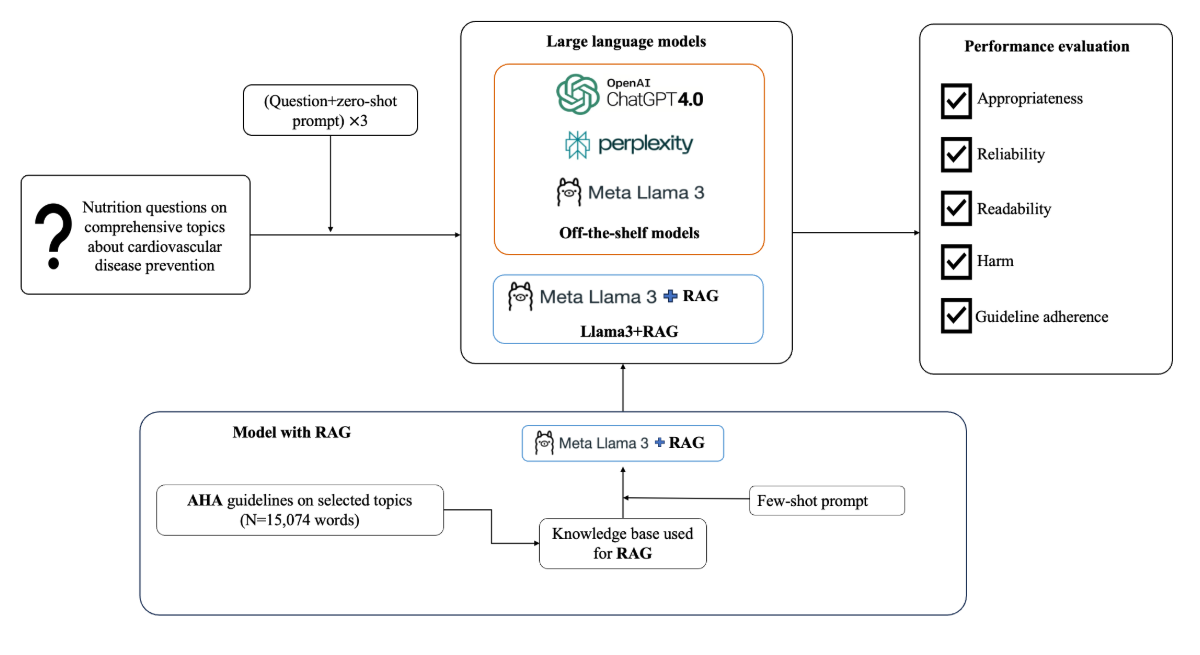
Overview of the research workflow: Responses to nutrition questions were produced using both off-the-shelf and retrieval-augmented generation (RAG)–enhanced large language models (Meta Llama 3 + RAG), grounded in American Heart Association (AHA) dietary guidelines. Model outputs were evaluated for appropriateness, reliability, readability, guideline adherence, and harm.

In the following subsections, we outline the benchmark used for evaluation, question bank development, and components of the Llama 3+RAG model, including the development of the knowledge bank, the prompt strategies used, the implementation of the RAG framework, and the methodology for model evaluation (Figure 1).

### Benchmark

We used the nutrition guidelines for CVD prevention as outlined by the AHA as the benchmark for this study. The AHA guidelines were selected because they are widely recognized, developed through systematic review of the latest evidence, and specifically focused on CVD prevention. This makes them the gold standard among national dietary recommendations for CVD prevention.

### Question Bank

We included 30 nutrition questions that comprehensively addressed CV health, including cooking practices, dietary patterns, and specific food and nutrient recommendations, that were developed and reviewed by a registered dietitian specializing in preventive cardiology at Stanford Medicine. The questions were developed using a systematic process to cover major domains within established CV dietary guidelines, including macronutrient recommendations, dietary patterns, specific food groups, and contemporary topics such as intermittent fasting and ketogenic diets that patients frequently ask clinicians about but are not fully addressed in traditional guidelines. These questions addressed topics such as types of cooking oil; sodium intake; meal ideas; macronutrient needs; the suitability of diets such as the ketogenic diet and intermittent fasting; and the role of foods such as eggs, red meat, and nuts, as well as beverages such as alcohol and caffeine, all in relation to heart health (Multimedia Appendix 1).

### Model Selection

We selected models representing different development paradigms available at the time of our study as of May 2024, to compare their performance in providing CV nutrition guidance. We included GPT-4o due to its strong performance on medical reasoning tasks, including the US Medical Licensing Examination and MedQA benchmarks, and its widespread adoption in clinical research contexts. We chose Perplexity for its distinct retrieval-augmented architecture that integrates real-time information retrieval with generation, potentially offering advantages for evidence-based nutrition guidance through its citation capabilities. Due to concerns about closed-source LLMs such as GPT-4o for sensitive medical applications and the challenges related to HIPAA (Health Insurance Portability and Accountability Act) compliance and data sovereignty requirements that are critical in health care settings, we incorporated Llama 3 as an open-source alternative at the time of the study. While both open- and closed-source model types can operate within regulatory frameworks with appropriate safeguards, Llama 3’s open-source nature provides research advantages through local deployment options and greater transparency for evaluating nutrition guidance performance. In addition, we developed an RAG-enhanced version of Llama 3 to evaluate whether domain-specific enhancement with CV nutrition guidelines could improve performance relative to general-purpose models. This selection enabled meaningful comparison between commercially available systems with different architectural approaches and specialized implementations, providing practical insights for health care organizations considering various implementation approaches for clinical nutrition guidance.

### Knowledge Bank

We developed a 15,074-word knowledge bank to customize Meta AI’s Llama 3-70B model using RAG, which included the AHA’s 2021 scientific statement on dietary and lifestyle recommendations for CVD prevention and scraped subpages from the AHA’s website on healthy eating. Cited sources and authors were included as metadata. The AHA scientific statement was selected as our primary source because it represents peer-reviewed, evidence-based recommendations developed through systematic literature review by leading experts in CV nutrition. A content mapping analysis confirmed that this focused knowledge bank provided explicit guidance for 93% (28/30) of our test questions, demonstrating comprehensive topic coverage despite its concise nature. This approach minimized potential knowledge conflicts that can occur with larger, less curated knowledge banks while ensuring that all retrieved content maintained the highest standards of clinical validity.

### Prompt Strategies

We implemented 2 prompt strategies: a zero-shot prompt, which provided generic information to the models, and a few-shot prompt, which included a sample response within the RAG framework.

### RAG Framework

A retrieval from the knowledge bank and a few-shot prompt were provided to the Llama 3-70B model as context input, with citations included in a section called *Context Source*. For implementation, we created a knowledge bank by extracting chunks from headings and subheadings from the AHA sources. We used the Beijing Academy of Artificial Intelligence’s bge-large-en embedding model to create vector representations of both the knowledge chunks and user queries. These embeddings were stored in a Chroma vector database, and our retrieval system used cosine similarity to identify the top 5 most relevant chunks for each query, which were then provided as context to the model. Model temperatures were adjusted to 0.5 to optimize response consistency and relevance based on preliminary testing across common nutrition questions. At temperatures below 0.4, responses adhered strictly to AHA guideline language but lacked conversational quality and explanatory depth. At temperatures above 0.6, responses became more engaging but included speculative dietary advice. The 0.5 setting optimized the balance between faithful representation of AHA nutrition recommendations and accessible, conversational explanations that effectively addressed common user questions. For example, GPT-4o was asked the following: “What cooking oils are recommended for a heart-healthy diet?” At a temperature of 0.4, the response began with “For a heart-healthy diet, it’s best to choose oils that are high in unsaturated fats and low in saturated fat.” This opening was direct and guideline adherent but limited in explanatory depth. At a temperature of 0.5 (our selected setting), the first sentence offered a balanced explanation with actionable framing: “For a heart-healthy diet, it’s important to choose oils that are high in unsaturated fats, which can help lower bad cholesterol levels and reduce the risk of heart disease.” At a temperature of 0.6, the response opened more conversationally but potentially overelaborately: “When it comes to supporting heart health through your diet, the oils you cook with can make a meaningful difference.” To standardize response format and citation practices, we incorporated a few-shot example demonstrating proper citation format and response structure in the system prompt. The following prompt was used:

You are an expert dietary assistant that gives dietary, and lifestyle recommendations based on questions. Use the following pieces of retrieved context to inform your answer. If you can’t figure out the answer from the context, say “Please reach out to a dietitian or medical practitioner for answers to this question.” Cite your sources specifically by including a section called “Citations”:

This structure ensured that the model appropriately referenced the context and source when generating responses.

### Model Evaluation

We posed each question to the models 3 times using the following zero-shot prompt: “Answer the following question as if you were a dietitian and cite sources from AHA.” Three expert reviewers (JB, AB, and VP) evaluated the responses against the benchmark for reliability, appropriateness, and potential harm, with scores assigned as 0 or 1 for each criterion. Guideline adherence was rated using a scale from 0 to 2: 0 for nonadherence (including responses with no guideline citation or responses linking to nonguideline sources regardless of link functionality), 1 for citing a legitimate guideline (either without a link or with a nonfunctional link), and 2 for citing a legitimate guideline with a technically functional link. Link functionality assessment was conducted only for those links specifically citing guidelines, confirming that they successfully directed to the intended guideline web page. The reviewers resolved disagreements through consensus. Model evaluation measures included reliability (consistency and replicability of results across evaluations under the same conditions), harm (provision or interpretation of incorrect or unsuitable responses that could negatively impact an individual’s health, wellness, or adherence to nutritional guidelines due to misleading, incomplete, inaccurate, or poorly communicated dietary recommendations), guideline adherence (assessed as referencing AHA guidelines in the response and appropriately citing the source), and appropriateness (relevance, accuracy, tone, context, completeness, and clarity of the response). In addition to the binary classification—appropriate or inappropriate—we analyzed response consistency using 2 supplementary metrics: partial appropriateness and diminishing appropriateness. Partial appropriateness indicated inconsistency in performance, defined as the occurrence of at least one inappropriate response across the 3 responses as determined by one or more evaluators. Diminishing appropriateness captured a specific degradation pattern, where the first response was scored as appropriate by at least one evaluator and either the second or third response was subsequently scored as inappropriate by the same evaluator. Interrater agreement was measured using the Cohen κ coefficient, and readability was estimated using the Flesch-Kincaid readability score [16]. The Flesch-Kincaid Grade Level estimates the US school grade needed to understand a text, ranging from 0 to 18; health care materials for the public should target a grade level of approximately 8 (ages of 13-14 years) [17]. Mean scores were compared each model using ANOVA, with statistical significance set at *P*<.05. The Tukey honestly significant difference test of mean differences was used to determine statistically significant differences between groups, whereas η^2^ was calculated to estimate effect sizes for these comparisons.

### Ethical Considerations

This study was deemed to be non–human participant research and exempt from review as a quality improvement initiative by the Stanford University Institutional Review Board (protocol 78053).

## Results

### Model Evaluation

The Llama 3+RAG model scored higher than the Perplexity, GPT-4o, and Llama 3 models across all measures (Table 1).

**Table 1:** Comparison of model performance across 5 key measures.

Measure	Llama 3+RAG^a^, mean (SD)	Perplexity, mean (SD)	GPT-4o, mean (SD)	Llama 3, mean (SD)	Statistics		
					*F* test (*df*)	*P* value	η^2^ (95% CI)
Reliability (score of 0-1)	0.47 (0.44)	0.37 (0.44)	0.09 (0.23)	0.26 (0.40)	5.58 (3, 116)	<.001	0.126 (0.023-0.225)
Appropriateness (score of 0-1)	0.83 (0.28)	0.45 (0.42)	0.55 (0.37)	0.48 (0.44)	5.92 (3, 116)	<.001	0.133 (0.026-0.223)
Guideline adherence (score of 0-2)	2 (0)	0.91 (0.91)	0.15 (0.27)	0.38 (0.41)	74.93 (3, 116)	<.001	0.66 (0.552-0.722)
Readability (score of 0-18)	11.1 (2.4)	9.1 (2.1)	9.4 (1.8)	9.0 (1.9)	6.79 (3, 116)	<.001	0.149 (0.037-0.252)
Harm (score of 0-1)	0 (0)	0.23 (0.43)	0.26 (0.44)	0.53 (0.51)	8.87 (3, 116)	<.001	0.187 (0.062-0.293)

^a^RAG: retrieval-augmented generation.

The Tukey honestly significant difference test for multiple comparisons found statistically significant differences between the Llama 3+RAG model and the off-the-shelf models in terms of mean values of readability (Perplexity: 95% CI 0.636-3.45 and *P*=.001; GPT-4o: 95% CI 0.346-3.16 and *P*=.008; Llama 3: 95% CI 0.699-3.52 and *P*<.001), guideline adherence (Perplexity: 95% CI 0.738-1.43 and *P*<.001; GPT-4o: 95% CI 1.49-2.19 and *P*<.001; Llama 3: 95% CI 1.27-1.97 and *P*<.001), and appropriateness (Perplexity: 95% CI 0.119-0.637 and *P*=.001; GPT-4o: 95% CI 0.015-0.535 and *P*=.03; Llama 3: 95% CI 0.082-0.600 and *P*=.005). The difference in the mean value for reliability was statistically significant between the Llama 3+RAG model and GPT-4o (95% CI 0.128-0.648; *P*<.001), but there were no statistically significant differences compared to Perplexity (95% CI −0.159 to 0.360; *P*=.75) or Llama 3 (95% CI −0.049 to 0.470; *P*=.16). With regard to harm, the difference in the mean value was statistically significant between the Llama 3+RAG model and the off-the-shelf Llama 3 model (95% CI 0.128-0.648; *P*<.001), but there were no statistically significant differences compared to Perplexity (95% CI −0.503 to 0.036; *P*=.12) or GPT-4o (95% CI −0.536 to 0.001; *P*=.06). The Cohen κ coefficient (κ>70%; *P*<.001) indicated high reviewer agreement (Table 1). The model responses to all questions that support the findings of this study are included in Multimedia Appendix 1. Here, the 95% CIs correspond to each measure—readability, guideline adherence, appropriateness, and reliability.

### Reliability

Repeated assessments of the same question produced different answers across attempts, indicating low reliability across all models, although the RAG-enhanced model performed better than the off-the-shelf models. For example, the responses to the question, “How can I reduce sodium in a heart-healthy diet?” posed to the RAG-enhanced model varied across 3 attempts (Table 2). Response 1 emphasized the benefits of reducing sodium intake, and response 2 warned about the negative effects of excessive sodium intake, whereas the third attempt focused on the health consequences of excessive sodium intake without mentioning specific food sources. The Perplexity model’s responses to the same question varied, including firm recommendations with differing amounts (responses of 1500 mg/day and 2000mg/day as shown in Table 2) across responses. The first response advocated for a strict sodium limit, whereas the second one allowed for a higher upper threshold for sodium intake with an ideal target. The third response suggested a broader upper limit for sodium intake while referencing the AHA guidelines and focused on reducing fluid retention and strain on the heart.

**Table 2:** Comparison of responses between the retrieval-augmented generation (RAG)–enhanced Llama 3 and Perplexity models for the following nutrition question: “How can I reduce sodium in a heart-healthy diet?”

	RAG-enhanced Llama 3 model	Perplexity
Response 1	“It is essential to note that reducing sodium intake by even 1,000 mg per day can have a positive impact on blood pressure and heart health. Furthermore, the majority of sodium in our diets comes from packaged, prepared, and restaurant foods, rather than from the saltshaker.”	“Limit sodium to 1,500 mg/day: For those with heart failure or high blood pressure, limit sodium intake to no more than 1,500 mg per day, which is about 2/3 teaspoon of salt.”
Response 2	“It is essential to note that more than 70% of the sodium Americans consume comes from packaged, prepared, and restaurant foods, rather than from the saltshaker. Therefore, it is crucial to be mindful of sodium content in these types of foods and read nutrition labels carefully.”	“Limit total sodium intake to less than 2,000 mg per day, ideally around 1,500 mg or less. This helps reduce fluid retention and strain on the heart.”
Response 3	“It is essential to be mindful of sodium intake because excessive sodium consumption can lead to high blood pressure and heart health issues. Even reducing daily sodium intake by 1,000 mg can have a significant positive impact on blood pressure and heart health.”	“The American Heart Association recommends limiting sodium to less than 2,300 mg per day, with an ideal limit of 1,500 mg for those with heart failure. Reducing sodium can help decrease fluid retention and strain on the heart.”

### Appropriateness

**Figure 2:**
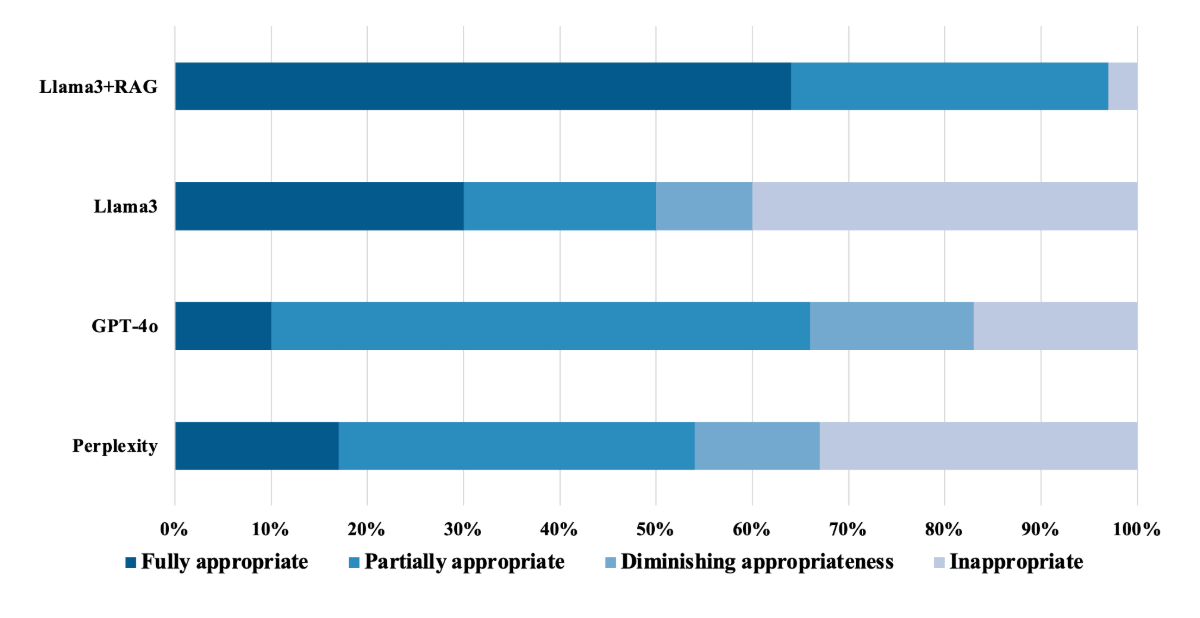
Appropriateness of responses to cardiovascular dietary questions across 4 large language models. The Llama 3+retrieval-augmented generation (RAG) model produced the fewest inappropriate responses (1/30, 3%) and partially appropriate responses (10/30, 33%) compared to Perplexity (inappropriate responses: 10/30, 33%; partially appropriate responses: 11/30, 37%), GPT-4o (inappropriate responses: 5/30, 17%; partially appropriate responses: 17/30, 57%), and Llama 3 (inappropriate responses: 12/30, 40%; partially appropriate responses: 6/30, 20%). Llama 3+RAG responses did not exhibit diminishing appropriateness—defined as a decline in response quality across successive outputs—whereas Perplexity, GPT-4o, and Llama 3 exhibited diminishing appropriateness in 13% (4/30), 17% (5/30), and 10% (3/30) of cases, respectively.

The Llama 3+RAG model generated the fewest inappropriate (1/30, 3%) and partially appropriate (10/30, 33%) responses, with no instances of diminishing appropriateness across repeated outputs. In comparison, Llama 3 generated 20% (6/30) partially appropriate responses, 40% (12/30) inappropriate responses, and 10% (3/30) diminishing appropriate responses; GPT-4o generated 57% (17/30) partially appropriate responses, 17% (5/30) inappropriate responses, and 17% (5/30) diminishing appropriate responses; and Perplexity generated 37% (11/30) partially appropriate responses, 33% (10/30) inappropriate responses, and 13% (4/30) diminishing appropriate responses (Figure 2). In the Llama 3+RAG model, partial appropriateness was primarily due to lack of clarity rather than issues with relevance, accuracy, tone, or context, which were more commonly observed in the off-the-shelf models. For example, the Llama 3+RAG model responded to questions about red meat consumption by recommending whole, unprocessed foods, which was not relevant to the question, making the responses less clear. In contrast, the off-the-shelf models responded to questions about sodium intake with a firm recommendation of 2000 mg for those with heart failure, whereas the benchmark recommends tailoring sodium intake based on the severity of heart failure [18].

### Harm Assessment and Representative Error Examples

No evidence of harm was found in the Llama 3+RAG model responses (Table 1), whereas the off-the-shelf model responses included answers that were unsuitable or could be interpreted incorrectly. The harm score was positive on the following topics: ketogenic diet, recommended calories per day, number of meals per day, low-carbohydrate diet for heart health, carbohydrate types and portions, intermittent fasting, and best diet for CVD prevention. Responses to questions about egg consumption were overly prescriptive and included a firm recommendation of 1 egg per day, which may be incorrect and potentially harmful for patients with hypercholesterolemia. Responses to questions about carbohydrate types included a recommendation of 55% to 60% of caloric intake, which contradicts the benchmark values of 45% to 60%, constitutes guideline misrepresentation, and may harm patients who need to maintain a carbohydrate intake of between 45% and 55% for better macronutrient balance. Responses to questions about number of meals per day were overly prescriptive and included a firm recommendation of 3 meals plus 2 snacks. Similarly, the response to the daily calorie intake question was overly prescriptive and included a firm recommendation of 1600 to 1700 calories. These recommendations may be harmful for patients who require small, frequent meals for blood sugar management and may be too restrictive for those with normal body weight and higher metabolic needs. The response to the question about the ketogenic diet’s heart healthiness constituted guideline misrepresentation by including benefits and suggestions that directly contradicted the benchmark, which cautions that the diet aligns poorly with heart-healthy eating guidelines.

### Guideline Adherence

The Llama 3+RAG model demonstrated 100% adherence to the guidelines and cited one or more AHA sources in every response. It also included citations from other credible sources, such as Harvard Health, the Mayo Clinic, and the Centers for Disease Control and Prevention. However, some of these links pointed to nonexistent web pages. The response to the question on daily protein intake cited Centers for Disease Control and Prevention recommendations on the daily protein goal but provided a link that did not exist. Further verification revealed that the categories referenced in the citation were unavailable, suggesting that the link may have been fictitiously created and never existed. Adherence was lower in the off-the-shelf models, where some responses cited noncredible sources such as Yelp and fictitious citations. In addition, many citations were not referenced in the response text. For example, the Perplexity model’s response to the question about heart-healthy high-protein snacks lacked an AHA source and included 8 local Yelp sources, 1 runner blog post, and 3 commercial websites. Of the 12 links, 3 (25%) could not be verified, and only 1 (8%) was referenced in the content.

### Readability

**Figure 3:**
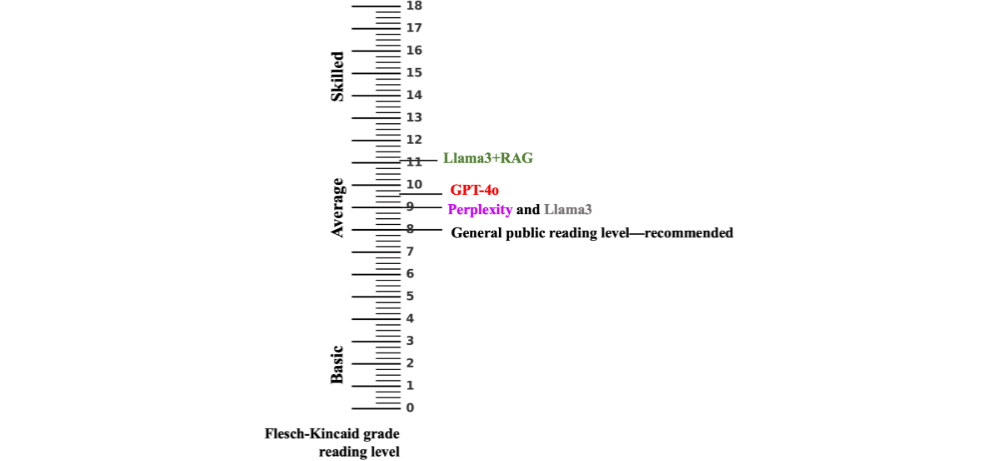
Estimated reading levels of large language model–generated dietary guidance compared to the general public reading level. This readability ruler visualizes mean reading grade levels of outputs generated by 4 large language models—Perplexity (mean 9, SD 2.1), GPT-4o (mean 9.4, SD 1.8), LLaMa 3 (mean 9.0, SD 1.9), and RAG-enhanced model (mean 11.1, SD 2.4)—benchmarked against the estimated US general public reading level (grade 8). The LLaMA3+RAG model responses were less readable (higher grade level) than the off-the-shelf models (F(3,116)=6.79, *P*<.001). .

Llama 3+RAG model responses were less readable than those of the off-the-shelf models. The readability score for the question about carbohydrate types was 7.7 for Perplexity, 8.3 for ChatGPT-4o, 6.1 for Llama 3, and 10.7 for the Llama 3+RAG model. The RAG-enhanced model responses included quotes from the AHA dietary guidelines and a comprehensive explanation with long and detailed sentences. It included definitions, benefits, and examples of food groups in formal language from the benchmark, with detailed reasoning for recommendations. The off-the-shelf models’ responses were organized using headings, bullet points, and examples, with each food category separated clearly using headings such as *Examples* and *Benefits*, providing shorter descriptions with little reasoning for the recommendations (Figure 3).

## Discussion

### Principal Findings

In this study, we integrated Llama 3 with an RAG framework and evaluated it with 3 off-the-shelf models (ChatGPT-4o, Perplexity, and Llama 3-70B) for guideline-based nutrition information related to CVD prevention. The off-the-shelf models scored lower across all measures and generated some harmful content, although their responses were more readable. In contrast, the Llama 3+RAG model produced more reliable, appropriate, and guideline-adherent responses, with no harmful content but with lower readability due to technical language. These results highlight the limitations of off-the-shelf LLMs in clinical nutrition and demonstrate the potential of RAG system integration to reduce harm and improve the appropriateness of LLM-powered digital tools for delivering evidence-based dietary information.

### Comparison to Prior Work

Our findings align with those of previous research showing variable performance of LLMs in specialized nutrition contexts [16,18]. Our work uniquely contributes to the literature by providing a systematic comparison of multiple model architectures within the same CV nutrition evaluation framework. This addresses limitations of previous studies in clinical nutrition [17,20] that primarily evaluated single models or lacked standardized benchmarks and RAG framework evaluations. Our approach reveals important performance differences between model types, offering practical insights for health care institutions considering LLM implementation for clinical nutrition guidance.

The Llama 3+RAG model outperformed the off-the-shelf models in terms of reliability, although there remains room for improvement. The inclusion and consistency of guidelines in the RAG-enhanced model’s responses across the 3 attempts varied in terms of detail, clarity of actionable guidance, and balance between benefits and risks. According to the health belief model’s stages of change, such variations may influence individual perceptions and subsequent behaviors depending on the individual’s stage of change [19]. LLMs are known to be inherently stochastic, generating different responses to the same prompt. As a result, trust in LLMs for clinical use remains a significant challenge due to this inherent variability as inconsistent outputs can lead to misinformed decisions and undermine confidence in these systems [21]. RAG enhancement offers a potential approach to improve consistency by integrating domain-specific retrieval sources, refining prompt design, and grounding outputs in clinical guidelines.

The responses produced by the off-the-shelf models were often inappropriate or partially appropriate or showed declining appropriateness across repeated prompts, whereas RAG enhancement significantly improved the model’s appropriateness, resulting in the fewest inappropriate and partially appropriate responses, with no evidence of diminishing appropriateness. Customization such as fine-tuning on domain-specific datasets reduced partial appropriateness from irrelevant information. However, fine-tuning with multiple small datasets risks overfitting, limiting the model’s ability to generalize across broader contexts. Improving the model’s ability to prioritize relevant context as enabled using RAG rather than relying solely on fine-tuning can enhance response appropriateness while mitigating the risk of overfitting [22]. In this study, the appropriateness of the off-the-shelf models was lower and declined across repeated prompts, a pattern consistent with LLM drift observed in other domains [20,23,24]. While recent guidelines recommend annual re-evaluation of model performance, we observed drift over much shorter intervals, similar to the results of previous studies [25,26]. This degradation may be driven by model updates and weight changes, particularly in closed-source systems such as Perplexity and GPT-4o where versioning is opaque. Although open-source models such as Llama 3 offer greater transparency in principle, our findings show that they are still susceptible to performance drift, highlighting the need for ongoing validation and adaptation [27].

Among the models evaluated, Llama 3 produced the highest number of harmful outputs; however, none were observed after RAG enhancement, suggesting that targeted retrieval significantly reduces harm. Harmful responses and representative failure cases were identified across a range of topics in outputs from Perplexity and GPT-4o, including guideline misrepresentations and overly prescriptive recommendations that overlooked individual needs. For instance, restrictive caloric guidance as observed in off-the-shelf model responses may lead to nutritional deficiencies, disordered eating, or poor adherence (refers to individuals being unable or unwilling to consistently follow the recommended dietary restrictions over time. When AI models provide overly restrictive caloric guidance without considering individual factors, people are likely to abandon these recommendations, rendering them ineffective regardless of their potential theoretical benefits), especially when metabolic, psychological, and medical needs are not considered. These findings underscore the need for a rigorous understanding of representative failure cases and safety mechanisms across off-the-shelf LLMs used in health care contexts, illustrating important limitations for clinical deployment. While keeping humans in the loop is often proposed as a strategy to mitigate harm, this approach alone may not be sufficient to address the risks associated with diminishing human clinical oversight. Guardrails including evidence-based guidelines and domain-specific customization are essential to mitigate these risks. Unverified sources can propagate misinformation, leading to misguided health advice with potential physiological and psychological harm [28,29]. Aligning AI outputs with expert-endorsed clinical standards and training on verified, peer-reviewed data are critical for improving credibility and minimizing the risk of misinformation, as highlighted in studies on LLM misinformation attacks [30,31].

Guideline adherence significantly improved in the Llama 3+RAG model, highlighting the potential of RAG customization and the gap between off-the-shelf LLMs and the level of performance required for clinical use. Trustworthy and verifiable responses are essential for regulators, clinicians, and patients, yet LLMs often generate hallucinated or incorrect URLs, as evidenced in the models evaluated, due to their reliance on next-token prediction. Therefore, implementing rigorous citation verification protocols is imperative in clinical settings, where erroneous information can directly impact patient safety and treatment decisions. This undermines reliability, particularly in critical medical contexts [32]. RAG models with accurate source verification show promise by retrieving information directly from reputable sources via search engines, reducing erroneous references and enhancing trust [33].

The Llama 3+RAG model generated less readable responses, likely due to the more technical language used in nutrition guidelines. Guideline-adherent content often contained specialized terminology and long sentences that decreased readability. Techniques such as zero-shot and few-shot prompting with carefully constructed examples of simplified clinical language, when refined with expert clinician input and clinical judgment, may enhance readability while preserving critical information and preventing information loss, thereby addressing the readability–guideline adherence trade-off [7,8,10,34]. To support this, emerging AI guidelines in medicine should define best practices for prompt engineering to support digital clinician-in-the-loop systems [35,36]. Tiered information presentation where responses include both a plain-language summary followed by more detailed technical content would allow users to access information at their preferred comprehension level. Integration of controlled medical vocabularies paired with consumer-friendly term mapping could systematically balance precision with accessibility. The integration of readability metrics into model evaluation protocols, combined with a composite scoring framework that explicitly weights accuracy, guideline adherence, and readability, could provide a standardized approach for optimizing this trade-off across different clinical contexts and patient populations.

Health care implementation of LLMs requires addressing several critical operational considerations. The computational requirements typically exceed standard clinical IT infrastructure capabilities, whereas retrieval processes in RAG-enhanced models introduce latency that affects time-sensitive workflows. Organizations must choose between substantial upfront hardware investments or scalable but potentially costly application programming interface implementations. These technical and financial considerations are likely to impact deployment decisions, particularly in resource-limited health care settings where infrastructure constraints are most pronounced.

### Limitations

First, this study evaluated only 3 off-the-shelf models and 1 RAG-enhanced model, limiting the generalizability of our findings. Although we selected widely used and representative models, our conclusions about comparative performance may not extend to other available LLMs.

Second, our assessment was conducted at a single point in time, which does not address model drift and account for the rapid evolution of these models through updates and improvements. To mitigate this limitation, we asked each question 3 times and reviewed all 3 responses, which provided some insight into the consistency of model performance but still represents a temporal snapshot rather than a longitudinal assessment of these rapidly evolving systems.

Third, we used a qualitative assessment approach rather than quantitative metrics, which introduces potential subjectivity in evaluation. We mitigated this limitation by using 3 independent expert reviewers and a standardized evaluation framework that used the established AHA CV dietary guidelines as the benchmark, which are derived from national guidelines developed through a rigorous, evidence-based process designed to be unbiased and enhance patient care, but some degree of subjective judgment remained inherent to the evaluation.

Fourth, the absence of patient testing to assess reading comprehension limits our understanding of how end users might interpret the nutrition information provided. While expert review ensures clinical accuracy, the practical impact of model-generated nutrition guidance on patient understanding and behavior remains undetermined within this study’s scope.

### Future Directions

#### Technical Advancement and Evaluation

As LLMs advance in reasoning capabilities and gain trust, establishing quantitative evaluation metrics including readability measures will enable more systematic comparisons between different model approaches. Building on this foundation, specific few-shot prompt strategies must be explored for CVD management to address the observed trade-off between higher guideline adherence and lower readability of outputs. Validating LLM performance and the efficacy of this approach requires comprehensive questions that extend beyond CV nutrition to include other lifestyle topics with established guidelines. To comprehensively assess performance variations, future research would benefit from broader comparative analyses involving smaller, more accessible LLMs and general backbone models such as Mistral alongside domain-specific LLMs such as Meditron or OpenBioLLM-70B. These comparisons would provide insights into relative performance across different architectures and specialized training approaches while also illuminating the practical trade-offs between model size and performance for resource-constrained clinical settings where HIPAA compliance remains essential.

#### Clinical Implementation and Governance

Transitioning from technical development to real-world application, clinical implementation research and patient perspectives are essential to examine how nutrition guidance tools integrate into existing health care workflows. This includes structured studies of patient and clinician acceptance, documentation integration, and quantifiable impacts on consultation efficiency. Complementing these efforts, regulatory and ethical frameworks must establish clear standards for transparency, accountability, and appropriate boundaries in both clinical and consumer contexts. These governance structures should evolve alongside the technology to ensure responsible deployment while maximizing clinical value. As a critical component of this governance, evaluation must consider the emotional nature of nutrition decisions as users’ personal circumstances and engagement patterns with AI have been shown to produce varied psychological responses, requiring monitoring systems that address both technical performance and psychological impact on user satisfaction.

#### Information Quality and Accuracy

A critical research priority for AI-generated guideline information is developing methods to detect 2 related but distinct types of failures: guideline misrepresentation failures (where models incorrectly interpret existing guidelines) and citation hallucinations (where models fabricate nonexistent sources or recommendations). Researchers must evaluate representative failure cases and quantify how frequently LLMs misinterpret guidelines, such as AHA recommendations on sodium restrictions, while simultaneously identifying instances in which models generate entirely fictional guideline content. These complementary detection approaches are essential because guideline information directly impacts patient care decisions, where even subtle deviations from evidence-based guidelines can compromise clinical outcomes.

### Conclusions

Our cross-sectional study evaluating LLM responses to common nutrition questions demonstrated that the RAG-enhanced Llama 3 model grounded in CV dietary guidelines consistently outperformed 3 off-the-shelf models (ChatGPT-4o, Perplexity, and Llama 3-70B) by providing more appropriate, reliable, and guideline-adherent responses to common nutrition questions with no evidence of harm, although these responses were less readable due to their technical language. In contrast, the off-the-shelf models scored lower on all measures and produced harmful content. These findings highlight the limitations of off-the-shelf models and demonstrate that RAG enhancement can improve LLM performance in delivering evidence-based dietary information.

## Appendix

Multimedia Appendix 1 Comprehensive model responses to 30 study questions (Perplexity, GPT-4o, LLaMA3 and RAG-enhanced model). This document includes all the questions (N=30) and responses from the 4 models evaluated in this study. For each question listed, the 3 responses generated by each of the 4 models are included. For example, Perplexity Answer 1 indicates the first response to the question by the Perplexity model, while GPT-4o Answer 3 indicates the third response to the question by the GPT-4o model.

## Abbreviations

AHA: American Heart Association
AI: artificial intelligence
CV: cardiovascular
CVD: cardiovascular disease
HIPAA: Health Insurance Portability and Accountability Act
LLM: large language model
RAG: retrieval-augmented generation

## Data Availability

The datasets generated or analyzed during this study are available in the [dietary_rag] repository. In this repository you will find the implementation code for the retrieval-augmented generation system developed for this study. Application programming interface keys for proprietary services used in this implementation must be obtained separately by researchers wishing to deploy the system. All other research data supporting the findings of this study are available upon request.
