# Supplementary material for: Evaluating Large Language Models and Retrieval-Augmented Generation Enhancement for Delivering Guideline-Adherent Nutrition Information for Cardiovascular Disease Prevention: Cross-Sectional Study
Source: J Med Internet Res. 2025 Oct 7;27:e78625. doi: 10.2196/78625 (PMC12541265; doi:10.2196/78625)
Supplement: Multimedia Appendix 1 [file jmir_v27i1e78625_app1.docx]

**Supplement**

**Title:** Comprehensive Model Responses to 30 Study Questions: Perplexity, GPT-4o, LLaMA3 and RAG enhanced model

This document includes all the questions (N=30) and responses from the four models evaluated in this study. The content is presented in a single table with two columns. For each question listed, the three responses generated by each of the four models are included. For example, Perplexity Answer 1 indicates the first response to the question by the Perplexity model, while GPT-4o Answer 3 indicates the third response to the question by the GPT-4o model.

| **Question 1** | **What cooking oils are recommended for a heart healthy diet?** |
| --- | --- |
| **Perplexity Answer 1** | For a heart-healthy diet, you should cook with oils that are high in monounsaturated and polyunsaturated fats, and low in saturated and trans fats. The best options are: Olive oil - One of the healthiest oils, especially extra virgin olive oil. It's high in monounsaturated fats and antioxidants that can reduce inflammation and lower heart disease risk[1][2][3][4][5]. Avocado oil - High in monounsaturated fats and has a high smoke point, making it suitable for high-heat cooking[1][2][3][5]. Canola oil - A good source of omega-3 fatty acids and has a neutral flavor[1][4]. Grapeseed oil - High in polyunsaturated fats and antioxidants, with a high smoke point[1][2][3]. Safflower oil - High in unsaturated fatty acids and has a high smoke point[2][3].  Sesame oil - High in antioxidants that can reduce heart cell damage but has a lower smoke point[1][2][3]. You should avoid or limit oils high in saturated fats like coconut oil, palm oil, and butter[2][3][5]. Oils like flaxseed and walnut oil are best used for cold preparations due to their low smoke points[3]. Moderation is key, as all oils are high in calories. Choose minimally processed oils without trans fats[1][4].  Citations:  [1] https://www.hvcnh.com/blog/heart-healthy-cooking-oils-why-must-you-choose-the-right-cooking-oil  [2] https://amscardiology.com/best-and-worst-cooking-oils-for-your-heart/  [3] https://www.healthline.com/nutrition/best-cooking-oils  [4] https://www.heart.org/en/healthy-living/healthy-eating/eat-smart/fats/healthy-cooking-oils  [5] https://health.clevelandclinic.org/how-to-choose-and-use-healthy-cooking-oils |
| **Perplexity Answer 2** | For a heart-healthy diet, you should cook with oils that are high in monounsaturated and polyunsaturated fats, and low in saturated and trans fats. The best options are: Olive oil - One of the healthiest oils, especially extra virgin olive oil. It's high in monounsaturated fats and antioxidants that can reduce inflammation and lower heart disease risk[1][2][3][4][5]. Avocado oil - High in monounsaturated fats and has a high smoke point, making it suitable for high-heat cooking[1][2][3][5]. Canola oil - A good source of omega-3 fatty acids and has a neutral flavor[1][4]. Grapeseed oil - High in polyunsaturated fats and antioxidants, with a high smoke point[1][2][3]. Safflower oil - High in unsaturated fatty acids and has a high smoke point[2][3]. Sesame oil - High in antioxidants that can reduce heart cell damage, but has a lower smoke point[1][2][3]. You should avoid or limit oils high in saturated fats like coconut oil, palm oil, and butter[2][3][5]. Oils like flaxseed and walnut oil are best used for cold preparations due to their low smoke points[3]. Moderation is key, as all oils are high in calories. Choose minimally processed oils without trans fats[1][4].  Citations:  [1] https://www.hvcnh.com/blog/heart-healthy-cooking-oils-why-must-you-choose-the-right-cooking-oil  [2] https://amscardiology.com/best-and-worst-cooking-oils-for-your-heart/  [3] https://www.healthline.com/nutrition/best-cooking-oils  [4] https://www.heart.org/en/healthy-living/healthy-eating/eat-smart/fats/healthy-cooking-oils  [5] https://health.clevelandclinic.org/how-to-choose-and-use-healthy-cooking-oils |
| **Perplexity Answer 3** | For a heart-healthy diet, you should cook with oils that are high in unsaturated fats (monounsaturated and polyunsaturated) and low in saturated fats. Here are some of the best cooking oils to choose: Extra Virgin Olive Oil: One of the healthiest options. It's packed with antioxidants and monounsaturated fats that can improve circulation and reduce inflammation[1][3][4]. Use for sautéing, roasting, and dressings. Avocado Oil: High in monounsaturated fats and has a high smoke point (520°F), making it great for sautéing and stir-frying[1][4]. Some studies suggest it may have anti-inflammatory benefits[1]. Safflower Oil: High in polyunsaturated fats. It has a neutral taste and high smoke point (510°F), suitable for sautéing and marinades[1][3]. Can help reduce heart disease risk and improve cholesterol levels[1].Grapeseed Oil: Rich in polyunsaturated fats and vitamin E. Its high smoke point makes it good for stir-frying and sautéing[1]. The antioxidants may help reduce heart disease risk[1]. Other good options include canola, peanut, sesame, and sunflower oils[3][4]. Oils to avoid or limit for heart health are coconut, palm, and palm kernel oils due to their high saturated fat content[1][4]. It's best to use a variety of unsaturated oils in your cooking rather than relying on just one type. Moderation is key, as all oils are high in calories[2][4].  Citations:  [1] https://amscardiology.com/best-and-worst-cooking-oils-for-your-heart/  [2] https://www.healthline.com/nutrition/best-cooking-oils  [3] https://www.heart.org/en/healthy-living/healthy-eating/eat-smart/fats/healthy-cooking-oils  [4] https://health.clevelandclinic.org/how-to-choose-and-use-healthy-cooking-oils  [5] https://www.health.harvard.edu/heart-health/choosing-heart-healthy-oils-for-home-cooking |
| **GPT4o Answer 1** | For a heart-healthy diet, it's important to choose oils that are high in unsaturated fats, which can help lower bad cholesterol levels and reduce the risk of heart disease. Here are some of the best oils to consider:1. Olive Oil: Extra virgin olive oil is particularly beneficial due to its high levels of monounsaturated fats and antioxidants. It's great for salad dressings, sautéing, and low-heat cooking. 2. Canola Oil: This oil is low in saturated fat and high in monounsaturated fat, making it a good all-purpose oil for cooking and baking. 3. Avocado Oil: Rich in monounsaturated fats, avocado oil has a high smoke point, making it suitable for high-heat cooking like frying and grilling.  4. Walnut Oil: High in omega-3 fatty acids, walnut oil is best used in cold applications like salad dressings or drizzling over dishes. 5. Flaxseed Oil: Also high in omega-3 fatty acids, flaxseed oil should not be heated and is best used in dressings or smoothies. 6. Sunflower Oil: High in vitamin E and low in saturated fat, sunflower oil is good for frying and baking due to its high smoke point. 7. Sesame Oil: Rich in both monounsaturated and polyunsaturated fats, sesame oil adds a nutty flavor to dishes and is suitable for stir-frying and dressings. Oils to Use Sparingly  - Coconut Oil: While popular, coconut oil is high in saturated fat and should be used in moderation.  - Palm Oil: This oil is also high in saturated fat and is best used sparingly.  Tips for Using Oils - Moderation: Even healthy oils are calorie-dense, so use them in moderation.  - Storage: Store oils in a cool, dark place to prevent them from becoming rancid.  - Variety: Use a variety of oils to benefit from different types of healthy fats and nutrients.  By choosing the right oils and using them appropriately, you can help maintain a heart-healthy diet. |
| **GPT4o Answer 2** | For a heart-healthy diet, it's important to choose oils that are high in unsaturated fats and low in saturated and trans fats. Here are some oils recommended for cooking that align with these guidelines:  1. Olive Oil: Rich in monounsaturated fats, olive oil is excellent for heart health. It's best used for sautéing, roasting, and as a salad dressing.  2. Canola Oil: Another good option, canola oil has a high content of monounsaturated fats and a moderate amount of polyunsaturated fats, including omega-3 fatty acids. It’s versatile and can be used for baking, frying, and sautéing.  3. Avocado Oil: High in monounsaturated fats, avocado oil is great for cooking at high temperatures due to its high smoke point.  4. Peanut Oil: With a high smoke point and a good balance of monounsaturated and polyunsaturated fats, peanut oil is suitable for frying and high-heat cooking.  5. Sunflower Oil: This oil is rich in vitamin E and has a high content of polyunsaturated fats. It's good for baking, frying, and cooking at medium to high temperatures.  6. Grapeseed Oil: High in polyunsaturated fats and vitamin E, grapeseed oil is ideal for sautéing, baking, and making dressings.  Oils to Limit or Avoid  - Coconut Oil: Although popular, coconut oil is high in saturated fats, which can raise LDL cholesterol levels.  - Palm Oil: Similar to coconut oil, palm oil contains a high amount of saturated fats.  For detailed information and guidelines on heart-healthy cooking oils, you can refer to resources provided by the American Heart Association. |
| **GPT4o Answer 3** | For a heart-healthy diet, it's important to choose oils that are high in unsaturated fats and low in saturated and trans fats. Here are some oils recommended for cooking that align with these guidelines:  1. Olive Oil: Rich in monounsaturated fats, olive oil is excellent for heart health. It's best used for sautéing, roasting, and as a salad dressing.  2. Canola Oil: Another good option, canola oil has a high content of monounsaturated fats and a moderate amount of polyunsaturated fats, including omega-3 fatty acids. It’s versatile and can be used for baking, frying, and sautéing.  3. Avocado Oil: High in monounsaturated fats, avocado oil is great for cooking at high temperatures due to its high smoke point.  4. Peanut Oil: With a high smoke point and a good balance of monounsaturated and polyunsaturated fats, peanut oil is suitable for frying and high-heat cooking.  5. Sunflower Oil: This oil is rich in vitamin E and has a high content of polyunsaturated fats. It's good for baking, frying, and cooking at medium to high temperatures.  6. Grapeseed Oil: High in polyunsaturated fats and vitamin E, grapeseed oil is ideal for sautéing, baking, and making dressings.  Oils to Limit or Avoid  - Coconut Oil: Although popular, coconut oil is high in saturated fats, which can raise LDL cholesterol levels.  - Palm Oil: Similar to coconut oil, palm oil contains a high amount of saturated fats.  For detailed information and guidelines on heart-healthy cooking oils, you can refer to resources provided by the American Heart Association. |
| **Llama 3**  **Answer 1** | 1. Olive Oil: Rich in monounsaturated fats, olive oil is an excellent choice for heart health. It's high in antioxidants and has been shown to lower total cholesterol and LDL ("bad") cholesterol levels.  2. Avocado Oil: Similar to olive oil, avocado oil is rich in monounsaturated fats and has been shown to improve heart health by reducing inflammation and improving blood lipid profiles.  3. Canola Oil: A good source of monounsaturated and polyunsaturated fats, canola oil can help lower LDL cholesterol and triglycerides.  4. Grapeseed Oil: Another good source of polyunsaturated fats, grapeseed oil has been shown to improve blood lipid profiles and reduce inflammation.  5. Fatty Fish Oils (e.g., salmon oil, sardine oil): While not typically used for cooking, these oils are rich in omega-3 fatty acids, which are essential for heart health.  Oils to Use in Moderation:  1. Coconut Oil: While high in saturated fats, coconut oil has some potential health benefits, including improving HDL ("good") cholesterol levels. However, it's still high in calories and should be used sparingly.  2. Palm Oil: Another saturated fat-rich oil, palm oil has been linked to increased LDL cholesterol levels and cardiovascular disease risk when consumed excessively.  Oils to Limit or Avoid:  1. Partially Hydrogenated Oils (e.g., partially hydrogenated soybean oil, partially hydrogenated corn oil): These oils contain trans fats, which are known to increase cardiovascular disease risk.  2. Saturated Fat-Rich Oils (e.g., lard, palm kernel oil): While not as harmful as partially hydrogenated oils, these oils are still high in saturated fats and can increase cardiovascular disease risk when consumed excessively.  Additional Tips:  1. Use oils in moderation: Even healthy oils are high in calories, so use them sparingly to avoid excessive calorie intake.  2. Choose high-quality oils: Opt for oils that are cold-pressed, expeller-pressed, or unrefined to ensure they're free from additives and processing chemicals.  3. Store oils properly: Keep oils in a cool, dark place to prevent oxidation and spoilage.  By following these guidelines, you can make informed choices about the oils you use in your cooking and support a heart-healthy diet. |
| **Llama 3**  **Answer 2** | When it comes to a heart-healthy diet, the type of oil you use for cooking can make a big difference. Here are some guidelines to help you choose the best oils for a heart-healthy diet:  Choose oils that are:  1. Unsaturated: These oils can help lower total cholesterol and LDL ("bad") cholesterol levels, reducing the risk of heart disease.  2. High in omega-3 fatty acids: Omega-3s, particularly EPA and DHA, have anti-inflammatory properties and can help lower triglycerides and blood pressure.  3. Low in omega-6 fatty acids: While some omega-6s are essential, excessive consumption can promote inflammation and increase heart disease risk.  Recommended oils:  1. Olive oil: Rich in monounsaturated fats, olive oil is an excellent choice for heart health. Look for "extra virgin" or "cold-pressed" olive oil for maximum benefits.  2. Avocado oil: High in monounsaturated fats and antioxidants, avocado oil is a great choice for sautéing, roasting, or making salad dressings.  3. Canola oil: A good source of monounsaturated and polyunsaturated fats, canola oil is a versatile oil for cooking and baking.  4. Fatty fish oils (e.g., salmon oil): While not typically used for cooking, fatty fish oils are rich in omega-3s and can be taken as supplements or added to food.  5. Grapeseed oil: A light, neutral-tasting oil with a high smoke point, making it suitable for high-heat cooking.  6. Rice bran oil: Rich in heart-healthy unsaturated fats, antioxidants, and vitamin E, rice bran oil is a good choice for stir-frying and sautéing.  Oils to limit or avoid:  1. Saturated fats: Coconut oil, palm oil, and palm kernel oil are high in saturated fats, which can increase cholesterol levels and heart disease risk.  2. Partially hydrogenated oils: These oils, like partially hydrogenated soybean oil, contain trans fats, which are known to increase heart disease risk.  3. Omega-6 rich oils: While some omega-6s are essential, excessive consumption of oils like corn oil, soybean oil, and sunflower oil can promote inflammation and increase heart disease risk.  Additional tips:  1. Use oils in moderation: Even healthy oils are high in calories, so use them sparingly to maintain a balanced diet.  2. Choose cold-pressed or expeller-pressed oils: These methods ensure that the oils are extracted without heat, preserving their nutritional value.  3. Store oils properly: Keep oils in a cool, dark place to prevent oxidation and spoilage.  By following these guidelines and incorporating heart-healthy oils into your diet, you'll be taking a significant step towards protecting your heart health. |
| **Llama 3**  **Answer 3** | Choosing the right oils for cooking is an important part of a heart-healthy diet. Here are some guidelines to help you make informed choices:  Healthy Oils:  1. Olive Oil: Rich in monounsaturated fats, olive oil is an excellent choice for heart health. It can help lower total cholesterol and LDL ("bad") cholesterol levels.  2. Avocado Oil: Similar to olive oil, avocado oil is high in monounsaturated fats, which can help improve heart health and lower cholesterol levels.  3. Canola Oil: A good source of monounsaturated and polyunsaturated fats, canola oil can help lower LDL cholesterol and triglycerides.  4. Nuts and Seeds Oils: Oils like walnut, flaxseed, and chia seed oil are rich in alpha-linolenic acid (ALA), a type of omega-3 fatty acid that supports heart health.  5. Grapeseed Oil: With a high polyunsaturated fat content, grapeseed oil can help lower LDL cholesterol and triglycerides.  Oils to Use in Moderation:  1. Peanut Oil: While peanut oil is relatively healthy, it's high in monounsaturated fats, so consume it in moderation.  2. Sesame Oil: Rich in antioxidants, sesame oil is a good choice, but it's high in calories, so use it sparingly.  Oils to Limit or Avoid:  1. Saturated Fats: Limit your intake of oils high in saturated fats, such as:  * Coconut Oil  * Palm Oil  * Palm Kernel Oil  * Lard  * Partially Hydrogenated Oils (e.g., partially hydrogenated soybean oil)  2. Trans Fats: Avoid oils with trans fats, which can increase your risk of heart disease:  * Partially Hydrogenated Oils (e.g., partially hydrogenated soybean oil)      - Fried foods and processed snacks often contain trans fats, so be mindful of food labels.  Tips for Healthy Oil Use:  1. Use the right oil for the job: Choose oils with high smoke points (e.g., avocado oil, grapeseed oil) for high-heat cooking, and those with lower smoke points (e.g., olive oil, flaxseed oil) for low-heat cooking or dressings.  2. Use oils in moderation: Even healthy oils are high in calories, so consume them in moderation as part of a balanced diet.  3. Store oils properly: Keep oils in a cool, dark place to prevent oxidation and spoilage.  By incorporating these healthy oils into your cooking routine and following the guidelines above, you'll be well on your way to a heart-healthy diet! |
| **LLaMA3+RAG Model Answer 1** | For a heart-healthy diet, it's essential to choose the right cooking oils that are rich in "better-for-you" fats and low in saturated fats. According to the American Heart Association, nontropical vegetable oils are the healthier choice for cooking and preparing food. These oils are preferred over solid fats like butter, shortening, lard, and stick margarine, as well as tropical oils like palm and coconut oil, which are high in saturated fats.  When selecting healthy oils, look for those with less than 4 grams of saturated fat per tablespoon and avoid partially hydrogenated oils or trans fats. Here are some common cooking oils that meet these criteria and are rich in "better-for-you" fats:  1. Canola oil: A good all-purpose oil with a neutral flavor, canola oil is low in saturated fats and high in monounsaturated and polyunsaturated fats.  2. Corn oil: Another versatile oil, corn oil is rich in polyunsaturated fats and has a mild flavor, making it suitable for sautéing, baking, and frying.  3. Olive oil: While often used as a finishing oil, olive oil can also be used for low-heat cooking due to its high smoke point. It's rich in monounsaturated fats and has been shown to have numerous heart-healthy benefits.  4. Peanut oil: With a high smoke point, peanut oil is ideal for high-heat cooking, such as stir-frying and frying. It's also rich in monounsaturated and polyunsaturated fats.  5. Safflower oil: This oil has a neutral flavor and a high smoke point, making it suitable for sautéing, baking, and frying. It's also rich in polyunsaturated fats.  6. Soybean oil: A common oil used in many food products, soybean oil is rich in polyunsaturated fats and has a neutral flavor, making it suitable for a variety of cooking applications.  7. Sunflower oil: With a high smoke point, sunflower oil is ideal for frying and sautéing. It's also rich in polyunsaturated fats and has a neutral flavor.  8. Vegetable oil: A blend of various oils, vegetable oil is often a combination of soybean, corn, and sunflower oils. It's a good all-purpose oil with a neutral flavor and a high smoke point.  Remember to always check the nutrition label and choose oils with less than 4 grams of saturated fat per tablespoon and no partially hydrogenated oils or trans fats.  Sources: American Heart Association. (2024). Cooking to Lower Cholesterol. Retrieved from <https://www.heart.org/en/health-topics/cholesterol/prevention-and-treatment-of-high-cholesterol-hyperlipidemia/cooking-to-lower-cholesterol:~:text=Liquid%20vegetable%20oils%20such%20as,Pan%2Dfry%20fish%20and%20poultry>  American Heart Association. (2017). Healthy Fats. Retrieved from <https://www.heart.org/en/healthy-living/healthy-eating/eat-smart/nutrition-basics/healthy-fats>  Academy of Nutrition and Dietetics. (2020). Choose Healthy Fats. Retrieved from <https://www.andjrnl.org/article/S2212-2672(20)30118-6/> |
| **LLaMA3+RAG Model Answer 2** | For a heart-healthy diet, it's essential to choose the right types of oils for cooking. According to the American Heart Association, nontropical vegetable oils are the way to go. These oils are healthier alternatives to solid fats like butter, shortening, lard, and stick margarine, as well as tropical oils like palm and coconut oil.  When selecting a cooking oil, look for ones with less than 4 grams of saturated fat per tablespoon. It's also crucial to avoid partially hydrogenated oils or trans fats, which can increase your risk of heart disease.  Here are some excellent options for heart-healthy cooking oils, as recommended by the American Heart Association:  1. Canola oil: With a mild flavor and high smoke point, canola oil is ideal for sautéing, baking, and making salad dressings.  2. Corn oil: Another versatile option, corn oil is great for frying, baking, and making sauces.  3. Olive oil: Rich in monounsaturated fats, olive oil is perfect for low-heat cooking, dressings, and marinades. Look for "extra virgin" or "virgin" olive oil for the highest quality.  4. Peanut oil: With a high smoke point, peanut oil is excellent for stir-frying, frying, and making sauces.  5. Safflower oil: This oil has a mild flavor and is suitable for baking, sautéing, and making salad dressings.  6. Soybean oil: A good all-purpose oil, soybean oil is great for frying, baking, and making sauces.  7. Sunflower oil: With a high smoke point, sunflower oil is ideal for frying, baking, and making salad dressings.  8. Vegetable oil: A blend of various oils, vegetable oil is a good option for general cooking, baking, and making sauces.  Remember, even though these oils are considered heart-healthy, it's still essential to consume them in moderation as part of a balanced diet.  Sources: American Heart Association. (2024). Cooking to Lower Cholesterol. Retrieved from <https://www.heart.org/en/health-topics/cholesterol/prevention-and-treatment-of-high-cholesterol-hyperlipidemia/cooking-to-lower-cholesterol> American Heart Association. (2017). Healthy Fats. Retrieved from <https://www.heart.org/en/healthy-living/healthy-eating/eat-smart/fats/healthy-fats> Academy of Nutrition and Dietetics. (2020). Healthy Fats and Oils. Retrieved from <https://www.andjrnl.org/article/S2212-2672(20)30143-6/> |
| **LLaMA3+RAG Model Answer 3** | For a heart-healthy diet, it's essential to choose the right cooking oils that are rich in "better-for-you" fats and low in saturated fats. According to the American Heart Association, nontropical vegetable oils are the way to go. These oils are liquid at room temperature and are a healthier alternative to solid fats like butter, shortening, lard, and stick margarine, as well as tropical oils like palm and coconut oil.  When selecting cooking oils, look for those with less than 4 grams of saturated fat per tablespoon and avoid partially hydrogenated oils or trans fats. Here are some excellent options:  1. Canola oil: A good all-purpose oil with a neutral flavor, canola oil is low in saturated fats and rich in monounsaturated and polyunsaturated fats.  2. Corn oil: Another neutral-tasting oil, corn oil is a good choice for frying, sautéing, and making salad dressings.  3. Olive oil: While it's not ideal for high-heat cooking, olive oil is a great choice for low-heat cooking, dressings, and marinades. It's rich in monounsaturated fats, which can help lower total cholesterol and LDL ("bad") cholesterol.  4. Peanut oil: With a mild nutty flavor, peanut oil is suitable for stir-fries, sautéing, and making sauces.  5. Safflower oil: A good choice for high-heat cooking, safflower oil has a neutral flavor and is rich in polyunsaturated fats.  6. Soybean oil: Another versatile oil, soybean oil is suitable for frying, baking, and making salad dressings.  7. Sunflower oil: With a mild flavor, sunflower oil is a good all-purpose oil for cooking, baking, and making salad dressings.  8. Vegetable oil: A blend of various oils, vegetable oil is a good choice for frying, sautéing, and making salad dressings.  Remember to always check the nutrition label and choose oils with less than 4 grams of saturated fat per tablespoon.  Sources: American Heart Association. (2024). Cooking to Lower Cholesterol. Retrieved from <https://www.heart.org/en/health-topics/cholesterol/prevention-and-treatment-of-high-cholesterol-hyperlipidemia/cooking-to-lower-cholesterol>  American Heart Association. (2017). Healthy Fats, Bad Fats: What's the Difference? Retrieved from <https://www.heart.org/en/healthy-living/healthy-eating/eat-smart/fats/healthy-fats-bad-fats-whats-the-difference>  Academy of Nutrition and Dietetics. (2020). Choose Healthy Fats. Retrieved from <https://www.andjrnl.org/article/S2212-2672(20)30123-1/> |
| **Question 2** | **How can I reduce sodium for a heart healthy diet?** |
| **Perplexity Answer 1** | To decrease sodium in a heart-healthy diet, follow these tips:  Limit Processed and Restaurant Foods  Over 70% of sodium intake comes from processed foods like bread, pizza, sandwiches, cold cuts, soups, and restaurant meals.[1][2][4] Limit these high-sodium foods and opt for fresh, unprocessed options instead.  Read Nutrition Labels: Check the Nutrition Facts label and choose foods with 5% or less of the Daily Value (DV) for sodium per serving. Look for labels that say ""low sodium,"" ""reduced sodium,"" or ""no salt added.""[1][4]  Cook at Home: Cooking meals at home allows you to control the amount of salt added. Limit use of salt, soy sauce, and other high-sodium condiments when cooking.[1][2] Use herbs, spices, and salt-free seasoning blends to add flavor instead of salt.  Choose Fresh Foods: Focus your diet on fresh, unprocessed foods like fruits, vegetables, lean proteins, whole grains, unsalted nuts and seeds, and low-fat dairy products, which are naturally low in sodium.[1][5]  Rinse Canned Foods: If using canned vegetables, beans, or fish, rinse them under water before eating to remove some of the sodium.[1]  Limit Sodium to 1500mg/day: For those with heart failure or high blood pressure, limit sodium intake to no more than 1500mg per day, which is about 2/3 teaspoon of salt.[1][2][4]  By following these tips, you can significantly reduce your sodium intake and make your diet more heart-healthy.[1][2][3][4]  Citations:  [1] https://health.gov/myhealthfinder/health-conditions/heart-health/eat-less-sodium-quick-tips  [2] https://www.riversideonline.com/en/patients-and-visitors/healthy-you-blog/blog/l/lose-your-salty-ways-the-importance-of-low-sodium-in-heart-failure  [3] https://www.mayoclinic.org/diseases-conditions/heart-disease/in-depth/heart-healthy-diet/art-20047702  [4] https://www.webmd.com/heart-disease/heart-failure/low-sodium-eating  [5] https://health.gov/myhealthfinder/health-conditions/heart-health/lower-sodium-foods-shopping-list |
| **Perplexity Answer 2** | To decrease sodium in a heart-healthy diet for managing heart failure, follow these tips:  1. Limit total sodium intake to less than 2,000 mg per day, ideally around 1,500 mg or less[1][4]. This helps reduce fluid retention and strain on the heart.  2. Avoid adding table salt to foods during cooking or at the table[1]. Use fresh herbs, spices, and salt-free seasoning blends instead to add flavor.  3. Eat more fresh, single-ingredient foods like fruits, vegetables, beans, whole grains, and lean proteins[2][5]. These are naturally low in sodium.  4. Read nutrition labels carefully and choose low-sodium or no-salt-added options for canned, frozen, and packaged foods[4]. Look for items with 5% or less of the daily value for sodium per serving.  5. Limit processed meats, canned soups, frozen meals, fast food, and restaurant meals[2][3], which are major sources of hidden sodium.  6. Cook meals at home as much as possible so you can control the sodium content[2].  7. Use fresh poultry, fish, lean meats, eggs, and dairy instead of cured, smoked, or processed varieties[4][5].  8. Make your own low-sodium salad dressings, sauces, and condiments at home instead of using store-bought versions[3][4].  By following these tips, you can significantly reduce your sodium intake while still enjoying flavorful, heart-healthy meals to help manage heart failure[1][2].  Citations:  [1] https://health.clevelandclinic.org/heart-failure-diet  [2] https://www.riversideonline.com/en/patients-and-visitors/healthy-you-blog/blog/l/lose-your-salty-ways-the-importance-of-low-sodium-in-heart-failure  [3] https://www.mayoclinic.org/diseases-conditions/heart-disease/in-depth/heart-healthy-diet/art-20047702  [4] https://www.webmd.com/heart-disease/heart-failure/low-sodium-eating  [5] https://health.gov/myhealthfinder/health-conditions/heart-health/lower-sodium-foods-shopping-list |
| **Perplexity Answer 3** | To decrease sodium in a heart-healthy diet, follow these tips:  1. Limit processed and restaurant foods, which are major sources of sodium. Opt for fresh, whole foods instead.[1][2][4]  2. Read nutrition labels carefully and choose low-sodium options with less than 5% of the daily value for sodium per serving.[4]  3. Avoid adding salt to foods during cooking or at the table. Use herbs, spices, and salt-free seasoning blends to add flavor instead.[1][3]  4. Buy fresh or frozen meats, poultry, fish, and vegetables without added sauces or seasonings.[4][5]  5. Rinse canned beans and vegetables to remove some sodium.[4]  6. Cook meals at home so you can control the sodium content.[2]  7. Choose low-sodium or sodium-free condiments like mustard, vinegar, and hot sauce.[3][4]  8. Limit high-sodium foods like processed meats, canned soups, frozen meals, snack foods, and fast food.[1][2][4]  The American Heart Association recommends limiting sodium to less than 2,300 mg per day, with an ideal limit of 1,500 mg for those with heart failure.[2] Reducing sodium can help decrease fluid retention and strain on the heart.[1][2]  Citations:  [1] https://health.clevelandclinic.org/heart-failure-diet  [2] https://www.riversideonline.com/en/patients-and-visitors/healthy-you-blog/blog/l/lose-your-salty-ways-the-importance-of-low-sodium-in-heart-failure  [3] https://www.mayoclinic.org/diseases-conditions/heart-disease/in-depth/heart-healthy-diet/art-20047702  [4] https://www.webmd.com/heart-disease/heart-failure/low-sodium-eating  [5] https://health.gov/myhealthfinder/health-conditions/heart-health/lower-sodium-foods-shopping-list |
| **GPT4o Answer 1** | Reducing sodium intake is crucial for a heart-healthy diet. Here are some strategies to help you decrease sodium in your diet:  1. Read Food Labels: Check the Nutrition Facts panel on food packages for sodium content. Aim for products with less than 140 mg of sodium per serving, which is considered low sodium.  2. Cook at Home: Preparing meals at home allows you to control the amount of salt added to your food. Use fresh ingredients instead of processed foods, which are often high in sodium.  3. Use Fresh or Frozen Vegetables: Choose fresh or frozen vegetables over canned ones, which often contain added salt. If you use canned vegetables, rinse them under water to remove some of the sodium.  4. Choose Low-Sodium Products: Look for low-sodium or no-salt-added versions of foods, such as soups, broths, and sauces. Many condiments, such as soy sauce and salad dressings, are also available in low-sodium versions.  5. Limit Processed Foods: Reduce your intake of processed foods such as deli meats, bacon, sausages, ready-to-eat meals, and snack foods, which tend to be high in sodium.  6. Use Herbs and Spices: Flavor your food with herbs, spices, garlic, and lemon juice instead of salt. This can enhance the taste without adding sodium.  7. Be Careful with Condiments: Many condiments, such as ketchup, mustard, and pickles, are high in sodium. Use them sparingly or look for low-sodium versions.  8. Eat Out Wisely: When dining out, request that your meal be prepared without added salt. Opt for grilled, baked, or steamed dishes instead of fried or breaded options.  9. Increase Potassium Intake: Foods rich in potassium can help counteract the effects of sodium on blood pressure. Include plenty of fruits and vegetables like bananas, potatoes, spinach, and tomatoes in your diet.  10. Avoid High-Sodium Snacks: Choose unsalted nuts, seeds, and popcorn over salty snacks like chips and pretzels. Fresh fruit and vegetables make excellent low-sodium snack options.  11. Be Mindful of Dairy Products: Some dairy products, like cheese and buttermilk, can be high in sodium. Opt for lower-sodium options or consume them in moderation.  12. Educate Yourself on Sodium Content: Familiarize yourself with the sodium content of common foods and aim to keep your total daily sodium intake within the recommended limits (2,300 mg per day or less, ideally 1,500 mg for those with hypertension or other risk factors).  By implementing these strategies, you can effectively reduce your sodium intake and support a heart-healthy diet. |
| **GPT4o Answer 2** | Reducing sodium intake is crucial for maintaining heart health, as high sodium consumption can lead to high blood pressure and increase the risk of heart disease and stroke. Here are several strategies to decrease sodium in your diet:  1. Read Food Labels Carefully - Check Sodium Content: Look for foods labeled "low sodium," "reduced sodium," or "no added salt." - Understand Serving Sizes: Be aware of the serving size and calculate the sodium content accordingly.  2. Choose Fresh and Unprocessed Foods - Fresh Fruits and Vegetables: Opt for fresh or frozen fruits and vegetables instead of canned varieties, which often contain added salt. - Lean Proteins: Choose fresh meats, poultry, and fish rather than processed or cured meats like bacon, ham, and sausages.  3. Cook at Home - Control Ingredients: Cooking at home allows you to control the amount of salt added to your food. - Use Herbs and Spices: Enhance flavor with herbs, spices, garlic, lemon juice, and vinegar instead of salt.  4. Limit Convenience and Fast Foods - Avoid Pre-Packaged Meals: Many convenience foods and fast foods are high in sodium. - Healthy Alternatives: When dining out, choose restaurants that offer low-sodium options and ask for your meal to be prepared without added salt.  5. Rinse Canned Foods - Remove Excess Salt: If you use canned vegetables or beans, rinse them under water to reduce their sodium content.  6. Be Cautious with Condiments and Sauces - Read Labels: Condiments like soy sauce, ketchup, and salad dressings can be high in sodium. Look for low-sodium versions. - Make Your Own: Consider making your own dressings and sauces to control sodium levels.  7. Use Salt Substitutes Wisely - Check with a Doctor: If you have certain health conditions, consult with your healthcare provider before using salt substitutes, as some contain potassium chloride, which might not be suitable for everyone.  8. Increase Potassium Intake - Balance Sodium: Foods high in potassium, such as bananas, spinach, and sweet potatoes, can help counteract the effects of sodium and lower blood pressure.  9. Monitor Dairy Products - Low-Sodium Options: Choose low-sodium or sodium-free dairy products like cheese, milk, and yogurt.  10. Limit Processed Snacks - Choose Wisely: Avoid salty snacks such as chips, pretzels, and salted nuts. Opt for unsalted or low-sodium versions.  11. Educate Yourself - Stay Informed: Keep learning about the sodium content in different foods and stay informed about dietary guidelines for sodium intake.  12. Gradually Reduce Salt - Taste Adjustment: Gradually reducing the amount of salt in your diet helps your taste buds adjust over time.  Recommended Sodium Intake - The American Heart Association recommends no more than 2,300 milligrams (mg) of sodium per day, with an ideal limit of no more than 1,500 mg per day for most adults, especially those with high blood pressure. Implementing these strategies can help you significantly reduce your sodium intake and support a heart-healthy diet. |
| **GPT4o Answer 3** | Decreasing sodium in a heart-healthy diet is an important step to reduce the risk of high blood pressure, heart disease, and stroke. Here are several strategies to help you reduce sodium intake:  1. Read Nutrition Labels - Check the sodium content on food labels and choose products with lower sodium levels. - Be aware of terms like "sodium-free," "low-sodium," "reduced-sodium," and "no salt added."  2. Cook at Home - Prepare meals from scratch to control the amount of salt added to your food. - Use fresh ingredients rather than processed or pre-packaged foods.  3. Use Herbs and Spices - Replace salt with herbs, spices, and other flavorings like garlic, lemon juice, and vinegar to enhance the taste of your food. - Experiment with salt-free seasoning blends.  4. Choose Fresh and Frozen Over Canned - Opt for fresh or frozen fruits and vegetables instead of canned varieties, which often contain added salt. - If using canned vegetables or beans, rinse them under water to remove excess sodium.  5. Limit Processed and Prepared Foods - Reduce intake of processed foods such as deli meats, sausages, canned soups, and frozen dinners, which are typically high in sodium. - Be cautious with snack foods like chips, pretzels, and salted nuts.  6. Dine Out Wisely - Request that your meal be prepared without added salt when dining out. - Choose restaurants that offer healthier, lower-sodium options.  7. Be Smart About Condiments - Limit use of high-sodium condiments such as soy sauce, ketchup, mustard, pickles, and salad dressings. - Look for low-sodium or sodium-free alternatives.  8. Watch Out for "Hidden" Sodium - Be mindful of foods that can contain hidden sodium, such as bread, pizza, sandwiches, and sauces. - Check labels on dairy products like cheese, cottage cheese, and buttermilk for sodium content.  9. Gradually Reduce Salt Intake - Gradually reduce the amount of salt you use to allow your taste buds to adjust over time. - As your palate adapts, you'll find that you need less salt to enjoy the flavor of your food.  10. Hydrate Well - Drink plenty of water, which helps to balance sodium levels in your body.  11. Educate Yourself and Plan Ahead - Educate yourself about the sodium content in different foods and plan your meals accordingly. - Keep track of your sodium intake to ensure you stay within recommended limits (usually less than 2,300 mg per day, or 1,500 mg for those at higher risk). By following these tips, you can effectively reduce your sodium intake and support your heart health. |
| **Llama3 Answer 1** | Decreasing sodium in a heart healthy diet is crucial to reduce the risk of high blood pressure, heart disease, and stroke. Here are some effective ways to lower sodium intake:  1. Cook from scratch: Processed and packaged foods are often high in sodium. Cooking meals from scratch using fresh ingredients allows you to control the amount of sodium that goes into your food.  2. Use herbs and spices for flavor: Instead of salt, try using herbs and spices to add flavor to your food. Some options include basil, oregano, thyme, cumin, and paprika.  3. Limit processed and packaged foods: Processed meats, canned goods, and frozen meals are often high in sodium. Opt for fresh, whole foods as much as possible.  4. Choose low-sodium options: When shopping, look for products labeled "low sodium" or "reduced sodium." These products may still be high in sodium, but they typically contain less than their regular counterparts.  5. Rinse canned beans and vegetables: Canned beans and vegetables are often packed in salty liquid. Rinsing them with water can help remove excess sodium.  6. Use salt-free seasoning blends: Many seasoning blends are high in sodium. Look for salt-free options or make your own blends using herbs and spices.  7. Limit soy sauce and teriyaki sauce: These condiments are often high in sodium. Use them sparingly or look for low-sodium alternatives.  8. Read food labels: Check the nutrition label to see how much sodium is in a particular food. Choose products with lower sodium content.  9. Use potassium-rich foods: Potassium can help balance out the effects of sodium in the body. Include potassium-rich foods like bananas, leafy greens, and sweet potatoes in your diet.  10. Gradually reduce sodium intake: If you're used to a high-sodium diet, it may take some time to adjust to a lower-sodium diet. Gradually reduce your sodium intake over time to give your taste buds time to adjust.  Daily sodium intake goals:  American Heart Association recommends no more than 2,300 milligrams (mg) of sodium per day.  Ideal limit is no more than 1,500 mg per day, especially for those who are at risk for high blood pressure or heart disease.  Tips for eating out:  Choose restaurants that offer low-sodium options.  Ask your server about sodium content or request that the chef prepare your meal with less salt.  Opt for fresh, grilled, or baked foods instead of fried or processed options.  Remember, reducing sodium intake takes time and effort, but it's a crucial step in maintaining a heart healthy diet. |
| **Llama3 Answer 2** | Decreasing sodium in a heart healthy diet is important because excessive sodium consumption can increase blood pressure, heart disease risk, and stroke risk. Here are some effective ways to reduce sodium in your diet:  1. Eat fresh, whole foods: Focus on consuming fresh fruits, vegetables, whole grains, lean proteins, and low-fat dairy products. These foods are naturally low in sodium.  2. Limit processed and packaged foods: Processed and packaged foods are often high in sodium. Try to limit or avoid foods like frozen meals, canned goods, bread, and sauces.  3. Read food labels: Check the nutrition label to see the sodium content of packaged foods. Choose products with lower sodium levels.  4. Use herbs and spices for flavor: Instead of salt, use herbs and spices to add flavor to your food.  5. Cook from scratch: Cooking from scratch allows you to control the amount of sodium that goes into your meals.  6. Use salt-free seasoning blends: Look for salt-free seasoning blends or homemade mixes to add flavor to your food without added sodium.  7. Rinse canned beans and vegetables: Rinsing canned beans and vegetables with water can help remove excess sodium.  8. Limit soy sauce and teriyaki sauce: These sauces are high in sodium. Use them sparingly or look for low-sodium alternatives.  9. Choose low-sodium options: When possible, choose low-sodium options for foods like bread, soups, and broths.  10. Gradually reduce sodium: If you're used to a high-sodium diet, it may take some time to adjust to lower sodium levels. Gradually reduce the amount of sodium in your diet to give your taste buds time to adjust.  11. Use potassium-rich foods: Potassium can help lower blood pressure and counteract the effects of sodium. Include potassium-rich foods like bananas, leafy greens, and sweet potatoes in your diet.  12. Limit restaurant and fast food: Restaurant and fast food meals are often high in sodium. Try to limit these meals or choose lower-sodium options.  Aim to consume no more than 2,300 milligrams of sodium per day, and ideally no more than 1,500 milligrams per day if you're at risk for high blood pressure or heart disease.  Remember, reducing sodium in your diet takes time and effort, but it's worth it to protect your heart health. |
| **Llama3 Answer 3** | Decreasing sodium in a heart healthy diet is crucial to reduce the risk of high blood pressure, heart disease, and stroke. Here are some effective ways to lower sodium intake:  1. Limit processed and packaged foods: Processed foods, such as canned goods, frozen meals, and condiments, are often high in sodium. Opt for whole, unprocessed foods like fruits, vegetables, whole grains, lean proteins, and healthy fats.  2. Read food labels: Check the Nutrition Facts label for sodium content. Look for products with less than 400mg of sodium per serving.  3. Cook from scratch: Preparing meals from scratch allows you to control the amount of sodium used in cooking. Use herbs and spices to add flavor instead of salt.  4. Use salt-free seasoning blends: Experiment with salt-free seasoning blends, such as those with herbs, spices, and citrus zest.  5. Rinse canned beans and vegetables: Rinsing canned beans and vegetables with water can help remove excess sodium.  6. Limit soy sauce and teriyaki sauce: These condiments are high in sodium. Opt for low-sodium versions or use them in moderation.  7. Choose low-sodium broths and stocks: Use low-sodium broths and stocks when cooking soups, stews, and sauces.  8. Avoid adding salt at the table: Break the habit of adding salt at the table. Instead, try using a salt-free seasoning blend or a squeeze of fresh lemon juice.  9. Gradually reduce sodium: If you're used to a high-sodium diet, it may take some time to adjust to a lower-sodium diet. Gradually reduce sodium intake over several weeks to give your taste buds time to adjust.  10. Eat more potassium-rich foods: Potassium can help lower blood pressure by balancing out the effects of sodium. Include potassium-rich foods like bananas, leafy greens, and sweet potatoes in your diet.  11. Limit restaurant and fast food: Restaurant and fast food meals are often high in sodium. Opt for cooking at home or choosing lower-sodium options when eating out.  12. Use the American Heart Association's sodium reduction guidelines: The AHA recommends consuming no more than 2,300mg of sodium per day. If you're at risk for high blood pressure or heart disease, aim for no more than 1,500mg per day.  Remember, it's not just about cutting out sodium, but also about increasing potassium and other essential nutrients in your diet. By following these tips, you can create a heart-healthy diet that's low in sodium and rich in flavor. |
| **LLaMA3+RAG Model Answer 1** | To decrease sodium in a heart-healthy diet, it's essential to be mindful of the foods you purchase, prepare, and consume. Here are some effective ways to reduce sodium intake: When Shopping for Food: 1. Choose packaged and prepared foods carefully: Compare labels and select products with the lowest sodium content per serving. 2. Opt for fresh and frozen poultry without added sodium solutions: Check the packaging for terms like "broth," "saline," or "sodium solution." 3. Select condiments with care: Look for reduced-sodium or lower-sodium versions of soy sauce, bottled salad dressings, dips, ketchup, jarred salsas, capers, mustard, pickles, olives, and relish. 4. Buy canned vegetables labeled "no salt added" and frozen vegetables without salty sauces: These can be used in casseroles, soups, or mixed dishes without adding excess salt. 5. Look for the American Heart Association's Heart-Check mark: This designation indicates that the food meets the AHA's sodium criteria and can be part of an overall healthy dietary pattern. When Preparing Food: 1. Use flavorful ingredients: Onions, garlic, herbs, spices, citrus juices, and vinegars can add flavor without salt. 2. Drain and rinse canned beans and vegetables: This can reduce sodium content by up to 40%. 3. Combine lower-sodium versions with regular versions: Mix equal parts of lower-sodium and regular foods to reduce overall sodium content. 4. Cook pasta, rice, and hot cereal without salt: Add flavorful ingredients instead of salt. 5. Grill, braise, roast, sear, or sauté: These cooking methods can bring out natural flavors and reduce the need for added salt. 6. Enjoy high-potassium foods regularly: Include sweet potatoes, potatoes, greens, tomatoes, white beans, kidney beans, nonfat yogurt, oranges, bananas, and cantaloupe in your diet to help counter the effects of sodium. At Restaurants: 1. Tell them how you like it: Ask for your dish to be prepared without extra salt and for sauces, dressings, and gravies to be served on the side. 2. Taste your food before adding salt: Add freshly ground black pepper or a squeeze of fresh lemon or lime juice instead of salt. 3. Watch out for high-sodium foods: Be cautious of foods labeled as pickled, brined, barbecued, cured, smoked, or those containing broth, au jus, soy sauce, miso, or teriyaki sauce. 4. Control portion sizes: Cut calories and sodium by asking for smaller portions or sharing a meal. 5. Ask about sodium content: Request nutrition information, including sodium content, from chain restaurants with 20 or more locations. Additional Tips:  Less salt doesn't mean bland taste: Your taste buds can adjust to less salt over time, and you can enhance flavors with cooking techniques and flavorful ingredients. Salt substitutes: Potassium salt can be a suitable alternative, but consult with your healthcare professional if you have certain medical conditions or take medications that affect potassium levels. Sources:  American Heart Association. (2024). How to Reduce Sodium in Your Diet. Retrieved from <https://www.heart.org/en/healthy-living/healthy-eating/eat-smart/sodium/how-to-reduce-sodium>  Academy of Nutrition and Dietetics. (2020). Sodium. Retrieved from <https://www.andjrnl.org/article/S2212-2672(20)30111-9/>  National Institutes of Health. (2020). Sodium in Your Diet. Retrieved from <https://www.nhlbi.nih.gov/health-topics/sodium-your-diet> |
| **LLaMA3+RAG Model Answer 2** | Decreasing sodium in a heart-healthy diet can be achieved through a combination of careful food selection, preparation, and cooking techniques. Here are some tips to help you reduce sodium in your diet:  When Shopping for Food:  1. Choose packaged and prepared foods carefully: Compare labels and select products with the lowest amount of sodium per serving. Different brands of the same food can have varying sodium levels.  2. Opt for fresh and frozen poultry: Select poultry that hasn't been injected with a sodium solution. Check the packaging for terms like ""broth,"" ""saline,"" or ""sodium solution.""  3. Select condiments with care: Look for reduced-sodium or lower-sodium versions of condiments like soy sauce, bottled salad dressings, dips, ketchup, jarred salsas, capers, mustard, pickles, olives, and relish.  4. Buy canned vegetables labeled ""no salt added"": Choose frozen vegetables without salty sauces. When added to casseroles, soups, or mixed dishes, you won't miss the salt.  5. Look for the American Heart Association's Heart-Check mark: This mark designates foods that meet the AHA's sodium criteria and can be part of an overall healthy dietary pattern.  When Preparing Food:  1. Use flavorful ingredients: Onions, garlic, herbs, spices, citrus juices, and vinegars can add flavor without salt.  2. Drain and rinse canned beans and vegetables: This can help cut sodium by up to 40%.  3. Combine lower-sodium versions with regular versions: Mix lower-sodium foods with regular versions to reduce sodium without sacrificing taste.  4. Cook pasta, rice, and hot cereal without salt: Add other flavorful ingredients to make up for the lack of salt.  5. Grill, braise, roast, sear, or sauté: These cooking methods can bring out natural flavors and reduce the need for salt.  6. Enjoy high-potassium foods regularly: Include sweet potatoes, potatoes, greens, tomatoes, and lower-sodium tomato sauce, white beans, kidney beans, nonfat yogurt, oranges, bananas, and cantaloupe in your diet. Potassium helps counter the effects of sodium and may help lower blood pressure.  When Eating Out:  1. Tell them how you like it: Ask for your dish to be made without extra salt and for sauces, dressings, and gravies to be served on the side.  2. Taste your food before adding salt: Add freshly ground black pepper or a squeeze of fresh lemon or lime juice to enhance flavor before adding salt.  3. Watch out for high-sodium foods: Be cautious of foods labeled as pickled, brined, barbecued, cured, smoked, broth, au jus, soy sauce, miso, or teriyaki sauce.  4. Control portion sizes: Cut calories and sodium by asking for smaller portions or sharing a meal.  5. Ask about sodium content: Chain restaurants with 20 or more locations must provide nutrition information, including sodium content, upon request.  Additional Tips:  1. Don't worry about bland taste: Your taste buds can adjust to less salt over time, and you may start to prefer the natural flavors of food.  2. Consider salt substitutes: Potassium salt substitutes can be a good option, but consult with your healthcare professional if you have certain medical conditions or take medications that affect potassium levels.  Sources:  American Heart Association. (2024). How to Reduce Sodium in Your Diet. Retrieved from <https://www.heart.org/en/healthy-living/healthy-eating/eat-smart/sodium/how-to-reduce-sodium> |
| **LLaMA3+RAG Model Answer 3** | To decrease sodium in a heart-healthy diet, it's essential to be mindful of the foods you buy, prepare, and eat at restaurants. Here are some tips to help you reduce sodium intake:  When Shopping for Food:  1. Choose packaged and prepared foods carefully: Compare labels and select products with the lowest amount of sodium per serving.  2. Pick fresh and frozen poultry without added sodium solutions: Check the packaging for terms like ""broth,"" ""saline,"" or ""sodium solution.""  3. Select condiments with care: Opt for reduced-sodium or lower-sodium versions of soy sauce, salad dressings, dips, ketchup, and other condiments.  4. Buy canned vegetables labeled ""no salt added"" and frozen vegetables without salty sauces: These can be added to dishes without excessive sodium.  5. Look for the American Heart Association's Heart-Check mark: This designation indicates that the food meets the AHA's sodium criteria for a heart-healthy diet.  When Preparing Food:  1. Use flavorful ingredients: Onions, garlic, herbs, spices, citrus juices, and vinegars can add flavor without salt.  2. Drain and rinse canned beans and vegetables: This can reduce sodium by up to 40%.  3. Combine lower-sodium versions with regular versions: Mixing them in equal parts can help reduce sodium intake without sacrificing flavor.  4. Cook pasta, rice, and hot cereal without salt: Add other flavorful ingredients to enhance taste.  5. Grill, braise, roast, sear, or sauté: These cooking methods can bring out natural flavors, reducing the need for added salt.  6. Enjoy high-potassium foods regularly: These include sweet potatoes, potatoes, greens, tomatoes, and lower-sodium tomato sauce, which can help counter the effects of sodium.  At Restaurants:  1. Tell them how you like it: Ask for your dish to be made without extra salt and for sauces, dressings, and gravies to be served on the side.  2. Taste your food before adding salt: Try adding freshly ground black pepper or a squeeze of fresh lemon or lime juice for flavor.  3. Watch out for high-sodium foods: Be cautious of foods labeled as ""pickled,"" ""brined,"" ""barbecued,"" ""cured,"" ""smoked,"" or containing soy sauce, miso, or teriyaki sauce.  4. Control portion sizes: Cutting calories often means cutting sodium intake as well.  5. Ask about sodium content: Request nutrition information, including sodium content, from chain restaurants with 20 or more locations.  Remember, reducing sodium intake doesn't mean sacrificing flavor. With time, your taste buds can adjust to less salt, and you may even prefer the taste of lower-sodium foods. Additionally, salt substitutes like potassium salt can be a viable option, but consult with your healthcare professional before making any changes.  Sources:  American Heart Association. (2024). How to Reduce Sodium in Your Diet. Retrieved from <https://www.heart.org/en/healthy-living/healthy-eating/eat-smart/sodium/how-to-reduce-sodium>  American Heart Association. (2024). Heart-Check Food Certification Program. Retrieved from <https://www.heart.org/en/healthy-living/healthy-eating/eat-smart/nutrition-basics/heart-check-food-certification-program> |
| **Question 3** | **How much sodium should I eat per day on a heart healthy diet?** |
| **Perplexity Answer 1** | For a heart-healthy diet, the American Heart Association recommends limiting sodium intake to no more than 2,300 milligrams (mg) per day, with an ideal limit of no more than 1,500 mg per day for most adults.[3] Specifically for individuals with heart failure, experts advise limiting sodium consumption to less than 2,000 mg per day.[1][2]  The key points regarding sodium intake for a heart-healthy diet are:  - Limit sodium to no more than 2,300 mg per day, with an ideal target of 1,500 mg or less per day.[3]  - For those with heart failure, aim for less than 2,000 mg of sodium per day.[1][2]  - The average American consumes around 3,500 mg of sodium daily, which is far too high and can increase blood pressure and strain the heart.[3][4]  - Over 70% of sodium intake comes from packaged, prepared, and restaurant foods, not just table salt.[3][4]  Reducing sodium intake can help lower blood pressure, decrease fluid retention, and ease the workload on the heart, which is especially beneficial for those with heart failure.[1][4] Checking food labels, limiting processed and restaurant foods, and cooking more meals at home with fresh ingredients can help reduce sodium consumption.[4]  Citations:  [1] https://health.clevelandclinic.org/heart-failure-diet  [2] https://www.mskcc.org/experience/patient-support/nutrition-cancer/diet-plans-cancer/cardiac-diet  [3] https://www.heart.org/en/healthy-living/healthy-eating/eat-smart/sodium/how-much-sodium-should-i-eat-per-day  [4] https://www.riversideonline.com/en/patients-and-visitors/healthy-you-blog/blog/l/lose-your-salty-ways-the-importance-of-low-sodium-in-heart-failure  [5] https://www.webmd.com/heart-disease/heart-failure/low-sodium-eating |
| **Perplexity Answer 2** | For a heart-healthy diet, the American Heart Association recommends limiting sodium intake to no more than 2,300 milligrams (mg) per day, with an ideal limit of no more than 1,500 mg per day for most adults.[3] Specifically for individuals with heart failure, experts advise limiting sodium to less than 2,000 mg per day.[1][2]  The key points regarding sodium intake for a heart-healthy diet are:  - Limit sodium to no more than 2,300 mg per day, with an ideal target of 1,500 mg or less per day.[3]  - For those with heart failure, aim for less than 2,000 mg of sodium per day.[1][2]  - The average American consumes around 3,500 mg of sodium daily, which is far too high and can increase blood pressure and strain the heart.[3][4]  - Over 70% of sodium intake comes from packaged, prepared, and restaurant foods, not just table salt.[3][4]  - Reading nutrition labels and being mindful of high-sodium foods like bread, pizza, sandwiches, cold cuts, soup, and fast food can help reduce sodium intake.[3][4]  Limiting sodium is crucial for managing heart failure, as it helps reduce fluid retention and ease the workload on the heart.[1][4] Making dietary changes with support from healthcare professionals can significantly improve heart health and symptoms of heart failure.[4]  Citations:  [1] https://health.clevelandclinic.org/heart-failure-diet  [2] https://www.mskcc.org/experience/patient-support/nutrition-cancer/diet-plans-cancer/cardiac-diet  [3] https://www.heart.org/en/healthy-living/healthy-eating/eat-smart/sodium/how-much-sodium-should-i-eat-per-day  [4] https://www.riversideonline.com/en/patients-and-visitors/healthy-you-blog/blog/l/lose-your-salty-ways-the-importance-of-low-sodium-in-heart-failure  [5] https://www.webmd.com/heart-disease/heart-failure/low-sodium-eating |
| **Perplexity Answer 3** | For a heart-healthy diet, the American Heart Association recommends limiting sodium intake to no more than 2,300 milligrams (mg) per day, with an ideal limit of no more than 1,500 mg per day for most adults.[3] Specifically for individuals with heart failure, experts advise limiting sodium to less than 2,000 mg per day.[1][2]  The key points regarding sodium intake for a heart-healthy diet are:  - Limit sodium to no more than 2,300 mg per day, with an ideal target of 1,500 mg or less per day.[3]  - For those with heart failure, aim for less than 2,000 mg of sodium per day.[1][2]  - The average American consumes around 3,500 mg of sodium daily, which is far too high and can increase blood pressure and strain the heart.[3][4]  - Over 70% of sodium intake comes from packaged, prepared, and restaurant foods, not just table salt.[3][4]  - Reading nutrition labels and being mindful of high-sodium foods like bread, pizza, sandwiches, cold cuts, soup, and fast food can help reduce sodium intake.[3][4]  Limiting sodium is crucial for managing heart failure, as it helps reduce fluid retention and ease the workload on the heart.[1][4] Making dietary changes with support from healthcare professionals can significantly improve heart health and symptoms of heart failure.[4]  Citations:  [1] https://health.clevelandclinic.org/heart-failure-diet  [2] https://www.mskcc.org/experience/patient-support/nutrition-cancer/diet-plans-cancer/cardiac-diet  [3] https://www.heart.org/en/healthy-living/healthy-eating/eat-smart/sodium/how-much-sodium-should-i-eat-per-day  [4] https://www.riversideonline.com/en/patients-and-visitors/healthy-you-blog/blog/l/lose-your-salty-ways-the-importance-of-low-sodium-in-heart-failure  [5] https://www.webmd.com/heart-disease/heart-failure/low-sodium-eating |
| **GPT4o Answer 1** | For a heart-healthy diet, it is recommended to limit sodium intake to help manage blood pressure and reduce the risk of cardiovascular diseases. The general guidelines are as follows:  1. American Heart Association (AHA): Recommends no more than 2,300 milligrams (mg) of sodium per day and ideally aims for 1,500 mg per day for most adults.  2. Dietary Guidelines for Americans: Suggest limiting sodium intake to less than 2,300 mg per day.  3. Individuals with Hypertension or Heart Disease: Should aim for even lower sodium levels, around 1,500 mg per day, as recommended by many health organizations.  Reducing sodium intake can help lower blood pressure, reduce the risk of stroke, heart failure, osteoporosis, stomach cancer, and kidney disease. It's important to read food labels, choose fresh and minimally processed foods, and be mindful of hidden sources of sodium in packaged and restaurant foods to effectively manage sodium intake. |
| **GPT4o Answer 2** | For a heart-healthy diet, it's generally recommended to limit sodium intake to reduce the risk of hypertension and cardiovascular diseases. The guidelines from various health organizations suggest:  1. American Heart Association (AHA): Recommends no more than 2,300 milligrams (mg) of sodium per day and moving toward an ideal limit of no more than 1,500 mg per day for most adults.  2. Centers for Disease Control and Prevention (CDC): Suggests a daily limit of less than 2,300 mg of sodium, which is equivalent to about one teaspoon of salt.  3. Dietary Guidelines for Americans: Also recommend limiting sodium intake to less than 2,300 mg per day.  For individuals with high blood pressure, diabetes, chronic kidney disease, or those who are middle-aged and older, a more stringent limit of 1,500 mg per day is often advised.  It's essential to read food labels and be mindful of the sodium content in processed and restaurant foods, as these are common sources of high sodium intake. Reducing sodium intake can significantly benefit heart health by helping to lower blood pressure and reduce the risk of heart disease and stroke. |
| **GPT4o Answer 3** | For a heart-healthy diet, the general recommendation is to limit sodium intake to less than 2,300 milligrams (mg) per day. However, for optimal cardiovascular health, it is often advised to aim for an even lower limit, ideally around 1,500 mg per day. This recommendation is particularly important for individuals with high blood pressure, heart disease, or those at risk of developing these conditions.  Here are some tips to help reduce sodium intake:  1. Read Food Labels: Check for sodium content in packaged and processed foods.  2. Cook at Home: Preparing meals at home allows you to control the amount of salt used.  3. Use Fresh Ingredients: Fresh fruits, vegetables, and lean proteins typically have lower sodium levels compared to processed foods.  4. Limit Processed Foods: Foods like canned soups, deli meats, and snack foods often contain high amounts of sodium.  5. Use Herbs and Spices: Enhance flavor with herbs, spices, and other seasonings instead of salt.  Always consult with a healthcare provider or a dietitian for personalized dietary advice, especially if you have specific health conditions or dietary needs. |
| **Llama 3 Answer 1** | The American Heart Association (AHA) recommends limiting daily sodium intake to less than 2,300 milligrams (mg) for healthy individuals. However, if you're at risk for high blood pressure or have kidney disease, the AHA suggests aiming for no more than 1,500 mg of sodium per day.  The National Institutes of Health's Dietary Approaches to Stop Hypertension (DASH) diet, which is a well-known heart-healthy eating plan, recommends the following daily sodium intake:  * 2,400 mg for people who are healthy and not at risk for high blood pressure  * 1,500 mg for people who are at risk for high blood pressure or have kidney disease  It's worth noting that the average American consumes more than 3,400 mg of sodium per day, which is well above the recommended amounts.  To put these numbers into perspective, here are the sodium contents of some common foods:  * 1 teaspoon of table salt: 2,300 mg  * 1 slice of bread: 100-200 mg  * 1 cup of canned soup: 900-1,300 mg  * 1 hot dog: 500-700 mg  * 1 cup of cooked pasta sauce: 400-600 mg  To reduce your sodium intake, try these tips:  1. Choose fresh, whole foods instead of processed and packaged ones.  2. Season your food with herbs and spices instead of salt.  3. Limit your use of sauces, condiments, and marinades that are high in sodium.  4. Rinse canned beans and vegetables with water to remove excess sodium.  5. Cook from scratch using salt-free recipes or reduced-sodium ingredients.  6. Read food labels and choose products with lower sodium content.  7. Limit your consumption of restaurant and take-out foods, which are often high in sodium.  Remember, it's not just about the amount of sodium you eat, but also about maintaining a balanced diet that is rich in fruits, vegetables, whole grains, and lean protein sources. |
| **Llama 3 Answer 2** | On a heart-healthy diet, it's recommended to limit your daily sodium intake to reduce your risk of high blood pressure, heart disease, and stroke. Here are the guidelines:  American Heart Association (AHA) Recommendations:  * Healthy individuals: Limit sodium intake to no more than 2,300 milligrams (mg) per day.  * Individuals with high blood pressure or at risk for high blood pressure: Limit sodium intake to no more than 1,500 mg per day.  Dietary Guidelines for Americans 2020:  * Healthy individuals: Consume no more than 2,300 mg of sodium per day.  * Individuals with high blood pressure or at risk for high blood pressure: Consume no more than 1,500-2,300 mg of sodium per day.  Why is sodium restriction important?  Excessive sodium consumption can lead to:  1. High blood pressure: Sodium can increase blood pressure, which is a major risk factor for heart disease and stroke.  2. Fluid retention: Sodium can cause your body to retain fluid, leading to swelling and bloating.  3. Kidney strain: Your kidneys have to work harder to process excess sodium, which can lead to kidney damage over time.  Tips to reduce sodium intake:  1. Choose whole, unprocessed foods: Focus on fruits, vegetables, whole grains, lean proteins, and low-fat dairy products, which are naturally low in sodium.  2. Limit processed and packaged foods: These foods are often high in sodium, so try to limit or avoid them.  3. Check nutrition labels: Look for products with lower sodium content (less than 400 mg per serving).  4. Season with herbs and spices: Instead of salt, use herbs and spices to add flavor to your food.  5. Cook from scratch: Preparing meals from scratch allows you to control the amount of sodium that goes into your food.  Remember, it may take some time to adjust to a lower-sodium diet, but your taste buds and body will thank you in the long run! |
| **Llama 3 Answer 3** | On a heart-healthy diet, it's recommended to limit your daily sodium intake to reduce your risk of high blood pressure, heart disease, and stroke. Here are the guidelines:  American Heart Association (AHA) Recommendations:  * Healthy individuals: No more than 2,300 milligrams (mg) of sodium per day.  * Individuals with high blood pressure or at risk for high blood pressure: No more than 1,500 mg of sodium per day.  Dietary Approaches to Stop Hypertension (DASH) Diet Recommendations:  * The DASH diet, which is a well-known heart-healthy eating plan, recommends limiting sodium intake to 2,300 mg per day.  * The DASH-Sodium trial, which was a study that tested the effects of sodium reduction on blood pressure, recommends limiting sodium intake to 1,500 mg per day.  Why is sodium reduction important for heart health?  * Excessive sodium consumption can lead to high blood pressure, which is a major risk factor for heart disease, stroke, and kidney disease.  * High sodium intake can also increase the risk of heart failure, atrial fibrillation, and kidney stones.  Tips to reduce sodium intake:  1. Read food labels: Check the Nutrition Facts panel for sodium content.  2. Choose fresh, whole foods: Focus on fruits, vegetables, whole grains, lean proteins, and low-fat dairy products, which are naturally low in sodium.  3. Limit processed and packaged foods: These products are often high in sodium.  4. Cook from scratch: Prepare meals using herbs, spices, and other seasonings instead of salt.  5. Use salt-free seasoning blends: Experiment with different flavors to add taste without sodium.  6. Rinse canned beans and vegetables: Remove excess sodium by rinsing them with water.  7. Gradually reduce sodium intake: If you're used to a high-sodium diet, gradually reduce your intake over time to give your taste buds time to adjust.  Remember, it's all about balance and making healthy choices. By limiting your sodium intake and following a heart-healthy diet, you'll be taking a significant step towards reducing your risk of heart disease and stroke. |
| **LLaMA3+RAG Model Answer 1** | According to the American Heart Association, a heart-healthy diet recommends limiting daily sodium intake to no more than 2,300 milligrams (mg) per day, with an ideal limit of no more than 1,500 mg per day for most adults. This is significantly lower than the average American's daily sodium consumption of 3,500 mg.  It's essential to note that reducing sodium intake by even 1,000 mg per day can have a positive impact on blood pressure and heart health. Furthermore, the majority of sodium in our diets comes from packaged, prepared, and restaurant foods, rather than from the salt shaker.  To track your daily sodium intake, it's recommended to:  1. Check the Nutrition Facts label on food packages to find the amount of sodium in one serving, listed in milligrams.  2. Be aware of the serving size and adjust accordingly if your portion size is larger than the listed serving size.  3. Look for sodium-related terms on food packages, such as ""salt/sodium-free,"" ""very low sodium,"" ""low sodium,"" ""reduced sodium,"" and ""light in sodium or lightly salted,"" to make informed choices.  It's unlikely that you're not getting enough sodium in your diet, as the body only needs a small amount (less than 500 mg per day) to function properly. However, certain individuals, such as competitive athletes, workers exposed to heat stress, and those with specific medical conditions, may require different sodium intake guidelines.  Sources:  American Heart Association. (2024). How Much Sodium Should I Eat Per Day? Retrieved from <https://www.heart.org/en/healthy-living/healthy-eating/eat-smart/sodium/how-much-sodium-should-i-eat-per-day>  American Heart Association. (2024). Sodium and Your Health. Retrieved from <https://www.heart.org/en/healthy-living/healthy-eating/eat-smart/sodium> |
| **LLaMA3+RAG Model Answer 2** | According to the American Heart Association, the recommended daily intake of sodium for a heart-healthy diet is no more than 2,300 milligrams (mg) per day, with an ideal limit of no more than 1,500 mg per day for most adults. This is significantly lower than the average American's daily sodium intake of 3,500 mg.  It's essential to note that more than 70% of the sodium Americans consume comes from packaged, prepared, and restaurant foods, rather than from the salt shaker. Therefore, it's crucial to be mindful of sodium content in these types of foods and to read nutrition labels carefully.  To track your daily sodium intake, check the Nutrition Facts label on food packages, which lists the amount of sodium in milligrams per serving. Be sure to take note of the serving size, as consuming larger portions can lead to higher sodium intake.  The American Heart Association also emphasizes that it's unlikely to consume too little sodium, as the body only needs a small amount (less than 500 mg per day) to function properly. However, certain individuals, such as competitive athletes, workers exposed to heat stress, and those with specific medical conditions, may require different sodium intake guidelines.  Sources:  American Heart Association. (2024). How Much Sodium Should I Eat Per Day? Retrieved from <https://www.heart.org/en/healthy-living/healthy-eating/eat-smart/sodium/how-much-sodium-should-i-eat-per-day>  American Heart Association. (2024). Sodium and Your Health. Retrieved from <https://www.heart.org/en/healthy-living/healthy-eating/eat-smart/sodium> |
| **LLaMA3+RAG Model Answer 3** | According to the American Heart Association, a heart-healthy diet recommends limiting daily sodium intake to no more than 2,300 milligrams (mg) per day, with an ideal limit of no more than 1,500 mg per day for most adults. This is significantly lower than the average American's daily sodium consumption of 3,500 mg.  It's essential to be mindful of sodium intake because excessive sodium consumption can lead to high blood pressure and heart health issues. Even reducing daily sodium intake by 1,000 mg can have a significant positive impact on blood pressure and heart health.  To track your daily sodium intake, check the Nutrition Facts label on packaged foods, which lists the amount of sodium in milligrams per serving. Be aware of the serving size and adjust accordingly, as your portion size may be larger than the listed serving size. Additionally, be cautious of sodium-containing ingredients like salt, sodium nitrate, and monosodium glutamate (MSG), which can contribute to the total sodium content of a food product.  It's unlikely that you're not getting enough sodium in your diet, as the body only requires a small amount (less than 500 mg per day) to function properly. However, certain individuals, such as competitive athletes, workers exposed to heat stress, and those with specific medical conditions, may require different sodium intake guidelines.  Sources:  American Heart Association. (2024). How Much Sodium Should I Eat Per Day? Retrieved from <https://www.heart.org/en/healthy-living/healthy-eating/eat-smart/sodium/how-much-sodium-should-i-eat-per-day>  American Heart Association. (2024). Sodium and Your Health. Retrieved from <https://www.heart.org/en/healthy-living/healthy-eating/eat-smart/sodium> |
| **Question 4** | Is intermittent fasting a good way to lose weight and healthy for the heart? |
| **Perplexity Answer 1** | Intermittent fasting can be an effective way to lose weight and may also provide some benefits for heart health, according to the research summarized in these search results:  For weight loss:  - A recent year-long study found that time-restricted eating combined with calorie restriction led to greater weight loss compared to calorie restriction alone[1].  - Multiple studies suggest intermittent fasting can produce weight loss comparable to traditional calorie-restricted diets[3].  For heart health:  - Intermittent fasting affects metabolic processes that may decrease inflammation, improve blood sugar regulation, and enhance the body's stress response - factors that can benefit heart health[3].  - Fasting induces cellular adaptations like increased insulin sensitivity and improved mitochondrial function, which could help reduce risk factors like diabetes, high cholesterol, and obesity linked to heart disease[1].  - Some research indicates intermittent fasting may improve markers of cardiometabolic health like insulin sensitivity[1][4].  However, the long-term effects of intermittent fasting are still unclear. Potential downsides include loss of lean muscle mass, fatigue, insomnia, and difficulty adhering to the fasting schedule[1][3]. Consulting a doctor before trying intermittent fasting is recommended, especially for those with medical conditions[3][4].  In summary, while the evidence is still emerging, intermittent fasting shows promise as a strategy for weight loss and potentially improving heart health markers when done safely under medical supervision[1][3][4].  Citations:  [1] https://www.health.harvard.edu/blog/should-you-try-intermittent-fasting-for-weight-loss-202207282790  [2] https://www.healthline.com/nutrition/intermittent-fasting-guide  [3] https://www.mayoclinichealthsystem.org/hometown-health/speaking-of-health/intermittent-fasting-fad-or-solution  [4] https://www.hopkinsmedicine.org/health/wellness-and-prevention/intermittent-fasting-what-is-it-and-how-does-it-work  [5] https://www.webmd.com/heart-disease/features/is-intermittent-fasting-bad-for-heart-health |
| **Perplexity Answer 2** | Intermittent fasting can be an effective way to lose weight and may also provide some benefits for heart health, according to the research summarized in these search results:  For weight loss:  - A recent year-long study found that time-restricted eating combined with calorie restriction led to greater weight loss compared to calorie restriction alone[1].  - Multiple studies suggest intermittent fasting can produce weight loss results similar to traditional calorie-restricted diets[3].  For heart health:  - Intermittent fasting affects metabolic processes that may decrease inflammation, improve blood sugar regulation, and enhance the body's stress response - factors that can benefit heart health[3].  - Fasting induces cellular adaptations like increased insulin sensitivity and improved mitochondrial function, which could help reduce risk factors like diabetes, high cholesterol, and obesity that contribute to heart disease[1].  However, the long-term effects of intermittent fasting are still unclear. Some potential downsides mentioned include:  - Loss of lean muscle mass during weight loss[1]  - Unpleasant side effects like fatigue, insomnia, and headaches, though these typically subside after a month[3]  - Difficulty adhering to the fasting schedule, especially for those with busy/irregular schedules[3]  Overall, the evidence suggests intermittent fasting can be an effective strategy for weight loss when combined with calorie restriction. The metabolic impacts may also provide heart health benefits, but more long-term research is still needed[1][3]. As with any diet, it's important to choose an approach that fits your lifestyle and consult a doctor, especially if you have any medical conditions.  Citations:  [1] https://www.health.harvard.edu/blog/should-you-try-intermittent-fasting-for-weight-loss-202207282790  [2] https://www.healthline.com/nutrition/intermittent-fasting-guide  [3] https://www.mayoclinichealthsystem.org/hometown-health/speaking-of-health/intermittent-fasting-fad-or-solution  [4] https://www.hopkinsmedicine.org/health/wellness-and-prevention/intermittent-fasting-what-is-it-and-how-does-it-work  [5] https://www.webmd.com/heart-disease/features/is-intermittent-fasting-bad-for-heart-health |
| **Perplexity Answer 3** | Intermittent fasting can be an effective way to lose weight and may also provide some benefits for heart health, according to the research summarized in these search results:  For weight loss:  - A recent year-long study found that time-restricted eating combined with calorie restriction led to greater weight loss compared to calorie restriction alone[1].  - Multiple studies suggest intermittent fasting can produce weight loss results similar to traditional calorie-restricted diets[3].  For heart health:  - Intermittent fasting affects metabolic processes that may decrease inflammation, improve blood sugar regulation, and enhance the body's stress response - factors that can benefit heart health[3].  - Fasting induces cellular adaptations like increased insulin sensitivity and improved mitochondrial function, which could help reduce risk factors like diabetes, high cholesterol, and obesity that contribute to heart disease[1].  However, the long-term effects of intermittent fasting are still unclear. Some potential downsides mentioned include:  - Loss of lean muscle mass during weight loss[1]  - Unpleasant side effects like fatigue, insomnia, and headaches, though these typically subside after a month[3]  - Difficulty adhering to the fasting schedule, especially for those with busy/irregular schedules[3]  Overall, the evidence suggests intermittent fasting can be an effective strategy for weight loss when combined with calorie restriction. The metabolic impacts may also provide heart health benefits, but more long-term research is still needed[1][3]. As with any diet, it's important to choose an approach that fits your lifestyle and consult a doctor, especially if you have any medical conditions.  Citations:  [1] https://www.health.harvard.edu/blog/should-you-try-intermittent-fasting-for-weight-loss-202207282790  [2] https://www.healthline.com/nutrition/intermittent-fasting-guide  [3] https://www.mayoclinichealthsystem.org/hometown-health/speaking-of-health/intermittent-fasting-fad-or-solution  [4] https://www.hopkinsmedicine.org/health/wellness-and-prevention/intermittent-fasting-what-is-it-and-how-does-it-work  [5] https://www.webmd.com/heart-disease/features/is-intermittent-fasting-bad-for-heart-health |
| **GPT4o Answer 1** | Intermittent fasting (IF) has gained popularity as a weight loss strategy and for its potential health benefits, including cardiovascular health. Here's a detailed look at its effects:  Weight Loss  1. Caloric Restriction: Intermittent fasting helps reduce overall caloric intake by limiting eating windows, which can lead to weight loss.  2. Metabolic Changes: IF may improve metabolism by enhancing insulin sensitivity, leading to more effective use of glucose and fat for energy.  3. Hormonal Effects: IF can increase levels of norepinephrine and growth hormone, which can aid in fat burning.  Cardiovascular Health  1. Improved Blood Pressure: Some studies suggest that intermittent fasting can lead to reductions in blood pressure, a key factor in cardiovascular health.  2. Cholesterol Levels: IF has been shown to improve lipid profiles by reducing LDL cholesterol and triglycerides while maintaining or increasing HDL cholesterol.  3. Inflammation and Oxidative Stress: IF may reduce markers of inflammation and oxidative stress, both of which are associated with heart disease.  Overall Health Benefits  1. Insulin Sensitivity: Improved insulin sensitivity can lower the risk of type 2 diabetes, which is a risk factor for cardiovascular disease.  2. Cellular Repair and Longevity: Fasting promotes autophagy, a process where the body removes damaged cells and regenerates new ones, potentially improving overall health and longevity.  3. Brain Health: IF may also support brain health by reducing oxidative stress and inflammation, which can positively impact cognitive function.  Considerations  1. Individual Variation: The effectiveness and safety of intermittent fasting can vary widely among individuals. Factors such as age, gender, medical history, and lifestyle play a significant role.  2. Sustainability: For some, maintaining an intermittent fasting regimen can be challenging. It's important to find a pattern that fits into one's lifestyle.  3. Nutritional Quality: The health benefits of intermittent fasting can be maximized by ensuring that the foods consumed during eating windows are nutritious and well-balanced.  Conclusion  Intermittent fasting can be an effective method for weight loss and may offer significant cardiovascular benefits. However, it should be approached with consideration of individual health conditions and lifestyle. Consulting with a healthcare provider before starting any fasting regimen is recommended to ensure it aligns with personal health needs and goals. |
| **GPT4o Answer 2** | Intermittent fasting (IF) has gained popularity as a weight loss method and has potential health benefits, particularly for cardiovascular health. However, its effectiveness and health implications can vary based on individual circumstances and the specific type of intermittent fasting practiced. Here is a summary of the evidence regarding intermittent fasting for weight loss and heart health:  Weight Loss  1. Caloric Restriction: Intermittent fasting typically leads to a reduction in calorie intake, which is the primary mechanism for weight loss. By limiting eating windows, people often consume fewer calories than they would with unrestricted eating.  2. Fat Loss: Studies have shown that intermittent fasting can lead to a significant reduction in body weight and fat mass. This is comparable to continuous calorie restriction.  3. Metabolic Rate: Short-term fasting may increase metabolic rate slightly due to increased levels of norepinephrine, a hormone that helps break down body fat. However, prolonged fasting can reduce metabolic rate, similar to continuous calorie restriction.  Heart Health  1. Improved Risk Factors: Intermittent fasting has been associated with improvements in various cardiovascular risk factors, including blood pressure, cholesterol levels, triglycerides, and blood sugar control.  2. Insulin Sensitivity: IF can improve insulin sensitivity, which is beneficial for heart health and can reduce the risk of type 2 diabetes, a significant risk factor for heart disease.  3. Inflammation and Oxidative Stress: Some studies suggest that intermittent fasting can reduce markers of inflammation and oxidative stress, both of which are linked to heart disease.  Types of Intermittent Fasting  - 16/8 Method: Eating during an 8-hour window and fasting for 16 hours.  - 5:2 Diet: Eating normally for 5 days a week and restricting calories to 500-600 on 2 non-consecutive days.  - Eat-Stop-Eat: Fasting for 24 hours once or twice a week.  - Alternate-Day Fasting: Alternating between normal eating and fasting every other day.  Considerations  1. Individual Variability: Not everyone responds to intermittent fasting in the same way. Some people may find it easy to adhere to, while others may struggle with the eating patterns or experience adverse effects like fatigue, irritability, or nutrient deficiencies.  2. Medical Conditions: People with certain medical conditions, such as diabetes or eating disorders, should consult with a healthcare professional before starting intermittent fasting.  3. Nutritional Quality: The quality of the diet during eating periods is crucial. Eating unhealthy, high-calorie foods during eating windows can negate the benefits of fasting.  Conclusion  Intermittent fasting can be an effective and sustainable way to lose weight and may offer significant heart health benefits. However, it is not a one-size-fits-all solution and should be tailored to individual needs and health conditions. Consulting with a healthcare provider is recommended before starting any fasting regimen, especially for individuals with underlying health issues. |
| **GPT4o Answer 3** | Intermittent fasting (IF) has gained popularity as a weight loss strategy and for its potential health benefits, including heart health. Let's break down the evidence regarding its effectiveness and safety.  Weight Loss  Intermittent Fasting and Weight Loss:  1. Caloric Restriction: Intermittent fasting naturally leads to a reduction in calorie intake, which is a primary factor in weight loss.  2. Metabolic Changes: IF can enhance metabolic rate by increasing levels of norepinephrine (noradrenaline), which can help burn more calories.  3. Hormonal Benefits: It can improve insulin sensitivity, which helps with fat loss. Lower insulin levels facilitate fat burning.  4. Autophagy: During fasting, the body initiates cellular repair processes like autophagy, which can support overall health and longevity.  Heart Health  Intermittent Fasting and Heart Health:  1. Blood Pressure: Some studies suggest that intermittent fasting can lower blood pressure, a major risk factor for heart disease.  2. Cholesterol Levels: IF may help reduce levels of LDL (bad) cholesterol and triglycerides, contributing to better cardiovascular health.  3. Inflammation: Fasting can reduce inflammation, which is linked to heart disease.  4. Blood Sugar Control: Improved insulin sensitivity and blood sugar control can reduce the risk of type 2 diabetes, which is a significant risk factor for heart disease.  Research and Evidence  - Clinical Studies: Numerous studies indicate that intermittent fasting can be as effective for weight loss as traditional calorie restriction diets. A review of multiple studies showed that IF can result in a typical weight loss of 3-8% over 3-24 weeks.  - Heart Health Markers: Research also suggests that intermittent fasting can improve several cardiovascular health markers. For instance, a study published in the New England Journal of Medicine highlighted the benefits of intermittent fasting on health and aging, including cardiovascular health.  Practical Considerations  1. Individual Variation: The effectiveness and safety of intermittent fasting can vary based on individual health conditions and lifestyle.  2. Sustainability: The long-term sustainability of intermittent fasting depends on individual preference and adherence.  3. Medical Supervision: People with certain medical conditions, such as diabetes or eating disorders, should consult healthcare providers before starting intermittent fasting.  Conclusion  Intermittent fasting can be an effective weight loss strategy and may offer heart health benefits. However, it’s essential to approach it with caution, especially for those with underlying health conditions. Consulting with a healthcare provider before starting any new dietary regimen is advisable to ensure it's appropriate for your specific health needs and goals. |
| **Llama 3 Answer 1** | Intermittent fasting (IF) has gained popularity in recent years, and research suggests that it can be an effective way to lose weight and improve cardiovascular health. Here's a summary of the benefits and some tips to get you started:  Weight Loss:  1. Caloric restriction: IF leads to a natural reduction in caloric intake, which can result in weight loss.  2. Improved insulin sensitivity: IF has been shown to improve insulin sensitivity, reducing the risk of developing type 2 diabetes and promoting weight loss.  3. Increased fat burning: IF can increase the body's ability to burn fat for energy, leading to weight loss and improved body composition.  Heart Health:  1. Reduced blood pressure: Regular IF has been shown to lower blood pressure and reduce the risk of heart disease.  2. Improved lipid profiles: IF has been found to increase HDL (good) cholesterol and lower triglycerides, reducing the risk of heart disease.  3. Reduced inflammation: IF has anti-inflammatory effects, which can help reduce the risk of cardiovascular disease.  Popular Intermittent Fasting Methods:  1. 16:8: Fast for 16 hours, eat within an 8-hour window. For example, skip breakfast and only eat between noon and 8 pm.  2. 5:2: Eat normally for 5 days, restrict calories to 500-600 on the other 2 days of the week.  3. Alternate Day Fasting: Alternate between days of normal eating and days of calorie restriction or fasting.  Tips for Success:  1. Start slow: Begin with a less intense method, like 12-hour fasts, and gradually increase the duration.  2. Stay hydrated: Drink plenty of water during fasting periods to help reduce hunger and improve overall health.  3. Listen to your body: If you feel weak, dizzy, or experience other negative side effects, consider modifying your approach or consulting a healthcare professional.  4. Be consistent: Stick to your IF schedule, even on weekends and holidays, to see the best results.  5. Combine with a healthy diet: Focus on whole, nutrient-dense foods during your eating windows to support overall health and weight loss.  Who Should Avoid Intermittent Fasting:  1. Pregnant or breastfeeding women: IF may not provide adequate nutrients for the fetus or baby.  2. People with a history of eating disorders: IF may exacerbate disordered eating patterns.  3. Diabetics: IF may lead to hypoglycemia (low blood sugar) if not properly managed.  4. People with certain medical conditions: Consult with a healthcare professional before starting IF if you have a medical condition, such as heart disease, kidney disease, or low blood pressure.  In conclusion, intermittent fasting can be a effective way to lose weight and improve heart health, but it's essential to consult with a healthcare professional, especially if you have any underlying medical conditions. Remember to start slow, stay hydrated, and listen to your body to ensure a successful and healthy IF experience. |
| **Llama 3 Answer 2** | Intermittent fasting (IF) has gained popularity in recent years, and for good reason. Research suggests that it can be an effective way to lose weight and improve overall heart health. Here's a breakdown of the benefits:  Weight Loss:  1. Reduced Caloric Intake: By restricting your eating window, you tend to consume fewer calories, which can lead to weight loss.  2. Increased Fat Burning: IF promotes fat burning as your body adapts to using stored fat for energy instead of relying on glucose from food.  3. Improved Insulin Sensitivity: IF helps your body respond better to insulin, reducing insulin resistance, which can contribute to weight gain.  Heart Health:  1. Lower Blood Pressure: Regular IF has been shown to lower blood pressure in people with hypertension.  2. Improved Lipid Profiles: IF can help reduce triglycerides, LDL cholesterol, and increase HDL cholesterol, all of which can contribute to a lower risk of heart disease.  3. Reduced Inflammation: IF has anti-inflammatory effects, which can help reduce cardiovascular risk.  4. Improved Cardiac Function: IF has been shown to improve cardiac function, including increased left ventricular ejection fraction and reduced left ventricular mass.  Other Benefits:  1. Increased Human Growth Hormone (HGH) Production: IF has been linked to increased production of HGH, which can help with weight loss and muscle gain.  2. Improved Mental Clarity and Focus: Many people report improved mental clarity and focus when practicing IF.  3. Increased Autophagy: IF can stimulate autophagy, a natural process in which your body breaks down and recycles damaged cells and proteins.  Common Methods:  1. 16:8: Eat during an 8-hour window, and fast for the remaining 16 hours.  2. 5:2: Eat normally for 5 days, and restrict calories to 500-600 on the other 2 days.  3. Alternate Day Fasting: Alternate between days of normal eating and days of calorie restriction or fasting.  Important Notes:  1. Consult a Healthcare Professional: Before starting any new diet or fasting regimen, consult with a healthcare professional, especially if you have any underlying health conditions.  2. Stay Hydrated: Drink plenty of water during your fasting periods to help reduce hunger and support overall health.  3. Listen to Your Body: If you feel weak, dizzy, or experience other negative side effects, consider adjusting your IF schedule or consulting with a healthcare professional.  In conclusion, intermittent fasting can be a effective way to lose weight and improve heart health, but it's essential to approach it in a safe and sustainable manner. |
| **Llama 3 Answer 3** | Intermittent fasting (IF) has gained popularity in recent years, and research suggests that it can be an effective way to lose weight and promote heart health. Here's a summary of the benefits and considerations:  Weight Loss:  1. Caloric restriction: IF promotes weight loss by limiting the eating window, which leads to an overall reduction in caloric intake.  2. Improved insulin sensitivity: IF has been shown to improve insulin sensitivity, which helps regulate blood sugar levels and can lead to weight loss.  3. Increased fat burning: IF has been found to increase fat burning and improve metabolic rate.  Heart Health:  1. Reduced blood pressure: Regular IF has been shown to lower blood pressure and reduce the risk of heart disease.  2. Improved lipid profiles: IF has been found to improve lipid profiles by reducing triglycerides, LDL (bad) cholesterol, and increasing HDL (good) cholesterol.  3. Inflammation reduction: IF has anti-inflammatory effects, which can help reduce inflammation in the body, a major risk factor for heart disease.  4. Cardiovascular risk reduction: IF has been shown to reduce cardiovascular risk factors, such as body mass index (BMI), waist circumference, and blood pressure.  Types of Intermittent Fasting:  1. 16:8 Method: Fast for 16 hours, eat within an 8-hour window. (e.g., skip breakfast, eat between noon and 8 pm)  2. 5:2 Diet: Eat normally for 5 days, restrict calories to 500-600 on the other 2 days of the week.  3. Alternate Day Fasting: Alternate between days of normal eating and days of calorie restriction or fasting.  4. Eat-Stop-Eat: Fast for 24 hours once or twice a week.  Considerations and Precautions:  1. Listen to your body: IF may not be suitable for everyone, especially those with certain medical conditions (e.g., diabetes, low blood pressure, eating disorders).  2. Stay hydrated: Drink plenty of water during fasting periods to help reduce side effects like headaches and fatigue.  3. Electrolyte balance: Consider taking electrolyte supplements or consuming electrolyte-rich foods during fasting periods to maintain balance.  4. Nutrient deficiencies: Ensure you're getting enough nutrients during your eating window to avoid deficiencies.  5. Consult a healthcare professional: Before starting any new diet or fasting regimen, consult with a healthcare professional, especially if you have any underlying health conditions.  In conclusion, intermittent fasting can be an effective way to lose weight and promote heart health, but it's essential to:  * Consult with a healthcare professional before starting  * Choose a method that suits your lifestyle and health goals  * Stay hydrated and balanced during fasting periods  * Listen to your body and adjust your approach as needed  Remember, it's always important to prioritize a balanced diet and a healthy lifestyle, regardless of the eating pattern you choose. |
| **LLaMA3+RAG Model Answer 1** | According to the American Heart Association, intermittent fasting, specifically time-restricted eating, may not be as beneficial for heart health as previously thought. The study found that people who limited their eating to less than 8 hours per day had a 91% higher risk of death due to cardiovascular disease compared to those who ate across 12-16 hours per day. This increased risk was also seen in people living with heart disease or cancer.  While previous research has found that time-restricted eating improves cardiometabolic health measures, such as blood pressure, blood glucose, and cholesterol levels, this study suggests that the long-term health effects of time-restricted eating may be adverse. The study's findings encourage a more cautious and personalized approach to dietary recommendations, taking into account an individual's health status and the latest scientific evidence.  It is essential to note that this study's limitations included its reliance on self-reported dietary information, which may be affected by participant's memory or recall and may not accurately assess typical eating patterns. Additionally, the study did not account for other factors that may play a role in health, outside of daily duration of eating and cause of death.  In conclusion, while time-restricted eating may have short-term benefits, the long-term effects on cardiovascular health are uncertain, and more research is needed to fully understand its impact. It is crucial for individuals, particularly those with existing heart conditions or cancer, to be aware of the potential risks associated with time-restricted eating and to consult with a healthcare professional before making significant changes to their diet.  Sources:  American Heart Association. (2024). Time-restricted eating may be associated with increased risk of cardiovascular death. Retrieved from <https://newsroom.heart.org/news/time-restricted-eating-may-be-associated-with-increased-risk-of-cardiovascular-death>  American Heart Association. (2024). Five Simple Steps to Control Your Blood Pressure. Retrieved from <https://www.heart.org/en/health-topics/high-blood-pressure/the-facts-about-high-blood-pressure/five-simple-steps-to-control-your-blood-pressure>  American Heart Association. (2024). Sodium. Retrieved from <https://www.heart.org/en/healthy-living/healthy-eating/eat-smart/sodium> |
| **LLaMA3+RAG Model Answer 2** | According to the American Heart Association, it appears that intermittent fasting, specifically time-restricted eating, may not be as beneficial for heart health as previously thought. The study found that people who limited their eating to less than 8 hours per day had a 91% higher risk of death due to cardiovascular disease compared to those who ate across 12-16 hours per day. Additionally, the study found that time-restricted eating did not reduce the overall risk of death from any cause. However, it is essential to note that the study's findings are based on an analysis of self-reported dietary information, which may be affected by participant memory or recall and may not accurately assess typical eating patterns. Furthermore, the study's limitations include the lack of consideration of factors that may play a role in health outside of daily duration of eating and cause of death. It is also important to highlight that previous research has found that time-restricted eating improves several cardiometabolic health measures, such as blood pressure, blood glucose, and cholesterol levels. Therefore, it is crucial to approach this topic with caution and consider the individual's health status and the latest scientific evidence when making dietary recommendations.  In conclusion, while time-restricted eating may have short-term benefits, the long-term effects on cardiovascular health are unclear, and more research is needed to fully understand its impact. It is essential to consult with a healthcare professional before making any significant changes to one's diet.  Sources:  American Heart Association. (2024). Preliminary research presented at the American Heart Association’s Epidemiology and Prevention│Lifestyle and Cardiometabolic Scientific Sessions 2024.  American Heart Association. (2024). Five Simple Steps to Control Your Blood Pressure. Retrieved from <https://www.heart.org/en/health-topics/high-blood-pressure/the-facts-about-high-blood-pressure/five-simple-steps-to-control-your-blood-pressure>  American Heart Association. (2024). Sodium. Retrieved from <https://www.heart.org/en/healthy-living/healthy-eating/eat-smart/sodium>  Note: The provided context is based on a preliminary research abstract, and the findings are considered preliminary until published as a full manuscript in a peer-reviewed scientific journal. |
| **LLaMA3+RAG Model Answer 3** | "According to the American Heart Association, it appears that intermittent fasting, specifically time-restricted eating, may not be as beneficial for heart health as previously thought. The study presented at the American Heart Association's Epidemiology and Prevention│Lifestyle and Cardiometabolic Scientific Sessions 2024 found that people who limited their eating to less than 8 hours per day had a 91% higher risk of death due to cardiovascular disease compared to those who ate across 12-16 hours per day.  The study's findings suggest that time-restricted eating may have short-term benefits, such as improving cardiometabolic health measures like blood pressure, blood glucose, and cholesterol levels, but may have long-term adverse effects on cardiovascular health. The researchers noted that the increased risk of cardiovascular death was also seen in people living with heart disease or cancer.  It's essential to consider the limitations of the study, including its reliance on self-reported dietary information, which may be affected by participant memory or recall and may not accurately assess typical eating patterns. Additionally, the study did not examine the nutrient quality of the diets typical of the different subsets of participants, which could be an alternate explanation for the findings. In conclusion, while time-restricted eating may have some benefits, the current evidence suggests that it may not be an effective strategy for weight loss or heart health in the long term. A more cautious, personalized approach to dietary recommendations is encouraged, taking into account an individual's health status and the latest scientific evidence.  Sources:  American Heart Association. (2024). Time-restricted eating may be associated with increased risk of cardiovascular death. Retrieved from <https://www.heart.org/en/news/2024/03/20/time-restricted-eating-may-be-associated-with-increased-risk-of-cardiovascular-death>  American Heart Association. (2024). Five Simple Steps to Control Your Blood Pressure. Retrieved from <https://www.heart.org/en/health-topics/high-blood-pressure/the-facts-about-high-blood-pressure/five-simple-steps-to-control-your-blood-pressure>  American Heart Association. (2024). Sodium. Retrieved from <https://www.heart.org/en/healthy-living/healthy-eating/eat-smart/sodium> |
| **Question 5** | **Is the ketogenic diet heart healthy?** |
| **Perplexity Answer 1** | The ketogenic diet is generally not considered heart-healthy for most people. Here are the key points: The ketogenic diet is very low in carbs and high in fat, which can raise levels of LDL (bad) cholesterol over time[1][3]. This increases the risk of heart disease and clogged arteries. While it may initially lower triglycerides, the long-term effects on cholesterol are concerning.  The diet eliminates or severely restricts healthy food groups like whole grains, fruits, and vegetables[3][5]. This can lead to nutritional deficiencies and a lack of fiber, which is protective against heart disease.  For heart patients, the ketogenic diet may increase risks like dangerous levels of ketosis, electrolyte imbalances, and interference with medications like warfarin and SGLT-2 inhibitors[3].  While short-term weight loss from ketogenic can improve some heart disease risk factors like blood pressure, these benefits tend to diminish over time[1][3]. The long-term impacts on heart health are still unclear.  Leading health organizations like the American Heart Association do not recommend the ketogenic diet for heart health[2]. Instead, they advise a balanced diet rich in vegetables, fruits, whole grains, lean proteins, and healthy fats.  In summary, while ketogenic may aid short-term weight loss, the potential risks to cholesterol levels, nutrition deficiencies, and medication interactions make this diet concerning for long-term heart health according to current research[1][2][3][4][5]. A more balanced, plant-based diet is generally advised for preventing heart disease.  Citations:  [1] https://www.bswhealth.com/blog/a-cardiologists-take-on-the-ketogenic-diet  [2] https://health.clevelandclinic.org/keto-diet-and-heart-disease  [3] https://www.health.harvard.edu/heart-health/keto-diet-is-not-healthy-and-may-harm-the-heart  [4] https://pubmed.ncbi.nlm.nih.gov/37571305/  [5] https://www.ohsu.edu/womens-health/keto-diet-healthy-your-heart |
| **Perplexity Answer 2** | The ketogenic diet is generally not considered heart-healthy for most people. Here are the key points:  The ketogenic diet is high in saturated fat and low in nutrient-rich foods like fruits, vegetables, and whole grains, which can raise LDL (""bad"") cholesterol levels and increase the risk of heart disease[1][3][5]. While it may initially lower triglycerides, the long-term effects on cholesterol are concerning.  Potential Heart Risks  - Raises LDL (""bad"") cholesterol over time, a major risk factor for heart disease[1][3]  - Lacks fiber, antioxidants, and other nutrients from plant foods that protect heart health[5]  - May interfere with medications like warfarin and SGLT2 inhibitors used for heart conditions[3]  - Potential for nutritional deficiencies if not carefully planned[1]  Potential Benefits  - Can aid short-term weight loss, which reduces heart disease risk factors like high blood pressure[1][2]  - May increase HDL (""good"") cholesterol initially[1]  - Some evidence it provides an alternative fuel for the heart in certain conditions[2][4]  However, most experts agree that for overall heart health, a balanced diet rich in vegetables, fruits, whole grains, lean proteins and healthy fats is recommended over the ketogenic diet for the general population[3][5]. The long-term effects of sustained ketosis on heart health are still unclear and require more research[2].  Citations:  [1] https://www.bswhealth.com/blog/a-cardiologists-take-on-the-ketogenic-diet  [2] https://health.clevelandclinic.org/keto-diet-and-heart-disease  [3] https://www.health.harvard.edu/heart-health/keto-diet-is-not-healthy-and-may-harm-the-heart  [4] https://pubmed.ncbi.nlm.nih.gov/37571305/  [5] https://www.ohsu.edu/womens-health/keto-diet-healthy-your-heart |
| **Perplexity Answer 3** | The ketogenic diet is generally not considered heart healthy, especially in the long term. Here are the key points:  The ketogenic diet typically raises LDL (""bad"") cholesterol levels, which can increase the risk of heart disease and cardiovascular events like heart attacks and strokes.[2][4][5] While it may initially lower LDL, this effect is often not sustained over 12 months.[1]  The extremely high fat intake on the ketogenic diet, often 60-80% of total calories from fat, far exceeds the recommended amount for a heart-healthy diet. This excessive saturated fat intake can contribute to plaque buildup in arteries.[4][5]  The ketogenic diet eliminates or severely restricts nutrient-dense foods like fruits, vegetables, whole grains, and legumes that are sources of fiber, antioxidants, and other beneficial compounds for heart health.[4][5]  While the ketogenic diet can lead to short-term weight loss, which may improve some cardiovascular risk factors, any benefits tend to diminish over time. The weight loss is also difficult to sustain long-term on such a restrictive diet.[1][4]  Potential Heart Health Risks  The high fat intake, lack of nutrient-dense plant foods, and increased LDL cholesterol levels associated with the ketogenic diet can raise the risk of heart disease, high blood pressure, obesity, and type 2 diabetes - all major risk factors for heart disease.[2][3][5]  Some studies suggest the ketogenic diet may impair heart and vascular function by negatively impacting the vascular endothelium.[3]  Recommendations  For long-term heart health, experts generally recommend a more balanced, plant-based dietary pattern like the Mediterranean diet over the ketogenic diet. This provides a good balance of healthy fats, fiber, antioxidants, and limited saturated fat.[4][5]  If attempting a ketogenic diet for weight loss, it should only be for a short period under medical supervision, especially for those with existing heart disease, high cholesterol, or other risk factors.[1][4]  Citations:  [1] https://www.bswhealth.com/blog/a-cardiologists-take-on-the-ketogenic-diet  [2] https://www.health.harvard.edu/heart-health/keto-diet-is-not-healthy-and-may-harm-the-heart  [3] https://pubmed.ncbi.nlm.nih.gov/37571305/  [4] https://www.ohsu.edu/womens-health/keto-diet-healthy-your-heart  [5] https://www.atlantichealth.org/about-us/stay-connected/news/content-central/2023/keto-diets-and-heart-health.html |
| **GPT4o Answer 1** | The ketogenic diet, which is high in fats and very low in carbohydrates, has been a subject of debate regarding its effects on heart health. Here are some key points to consider:  Potential Benefits for Heart Health:  1. Weight Loss: The ketogenic diet can lead to significant weight loss, which can improve cardiovascular health by reducing the strain on the heart and improving various heart disease risk factors.  2. Improved HDL Cholesterol: Some studies suggest that the ketogenic diet can increase levels of high-density lipoprotein (HDL) cholesterol, which is often referred to as ""good"" cholesterol. Higher HDL levels are associated with a lower risk of heart disease.  3. Reduction in Triglycerides: The ketogenic diet may help lower triglyceride levels, which is beneficial since high levels of triglycerides are a risk factor for heart disease.  Potential Risks for Heart Health:  1. Increased LDL Cholesterol: One of the main concerns is that the ketogenic diet can lead to an increase in low-density lipoprotein (LDL) cholesterol, often referred to as ""bad"" cholesterol. Elevated LDL levels can increase the risk of heart disease.  2. Nutrient Deficiencies: The restrictive nature of the ketogenic diet can lead to deficiencies in important nutrients, including fiber, which is essential for heart health. A lack of certain vitamins and minerals can also negatively impact cardiovascular health.  3. Sustainability and Long-term Effects: The long-term effects of the ketogenic diet are not well understood, and maintaining such a restrictive diet can be challenging. Yo-yo dieting or frequent weight cycling can also negatively impact heart health.  Individual Variability:  It's important to note that the effects of the ketogenic diet can vary significantly between individuals. Factors such as genetics, existing health conditions, and overall lifestyle play crucial roles in determining how a person responds to the diet.  Recommendations:  - Consult a Healthcare Provider: Before starting the ketogenic diet, especially if you have pre-existing heart conditions or other health issues, it's crucial to consult with a healthcare provider.  - Focus on Healthy Fats: If you decide to follow the ketogenic diet, prioritize healthy fats like those from avocados, nuts, seeds, and olive oil, rather than saturated and trans fats.  - Monitor Blood Levels: Regular monitoring of cholesterol and triglyceride levels is important to ensure that the diet is not adversely affecting your heart health.  - Balanced Approach: Consider a balanced approach that includes a variety of nutrients and food groups. Diets like the Mediterranean diet, which emphasize healthy fats, lean proteins, and plenty of fruits and vegetables, are also known for their heart health benefits.  In conclusion, while the ketogenic diet can offer certain benefits for heart health, it also poses potential risks, particularly concerning cholesterol levels. It's essential to approach this diet carefully and seek professional advice to tailor it to your individual health needs. |
| **GPT4o Answer 2** | The ketogenic (keto) diet, which is high in fats and very low in carbohydrates, has gained popularity for its potential weight loss and other health benefits. However, its impact on heart health is a topic of debate and ongoing research. Here are some key points to consider regarding the ketogenic diet and heart health:  Potential Benefits:  1. Weight Loss: The ketogenic diet can lead to significant weight loss, which can improve various markers of heart health, such as blood pressure, cholesterol levels, and blood sugar control.  2. Blood Sugar Control: By reducing carbohydrate intake, the ketogenic diet can help stabilize blood sugar levels, which is beneficial for people with type 2 diabetes or prediabetes.  3. Increased HDL Cholesterol: Some studies have shown that the ketogenic diet can raise levels of high-density lipoprotein (HDL) cholesterol, which is considered ""good"" cholesterol.  Potential Risks:  1. Elevated LDL Cholesterol: The ketogenic diet can increase levels of low-density lipoprotein (LDL) cholesterol in some people, which is considered ""bad"" cholesterol and is a risk factor for heart disease.  2. Nutrient Deficiency: The restrictive nature of the ketogenic diet can lead to deficiencies in important nutrients like fiber, vitamins, and minerals, which are essential for heart health.  3. Saturated Fat Intake: The ketogenic diet often includes high amounts of saturated fats, which can negatively impact heart health if consumed in excess.  Studies and Evidence:  - Mixed Results: Research on the ketogenic diet's long-term effects on heart health has yielded mixed results. Some studies suggest improvements in cardiovascular risk factors, while others indicate potential negative impacts.  - Short-Term vs. Long-Term: Short-term studies often show favorable results for weight loss and blood sugar control. However, the long-term effects are less clear, and there are concerns about the sustainability of the diet and its impact on heart health over time.  Individual Variation:  - Personal Response: Individual responses to the ketogenic diet can vary widely. Some people may experience significant improvements in heart health markers, while others may see adverse effects, particularly related to cholesterol levels.  Recommendations:  - Consult a Healthcare Provider: Before starting the ketogenic diet, it is important to consult with a healthcare provider, especially if you have underlying health conditions or concerns about heart health.  - Balanced Approach: A balanced approach that includes healthy fats (such as those from fish, nuts, and olive oil), plenty of vegetables, and adequate protein sources can help mitigate some of the potential risks associated with the ketogenic diet.  In summary, while the ketogenic diet may offer benefits for weight loss and blood sugar control, its impact on heart health is complex and can vary from person to person. Careful consideration and medical guidance are recommended to ensure that the diet supports overall health, including heart health. |
| **GPT4o Answer 3** | The ketogenic (keto) diet, characterized by high fat, moderate protein, and very low carbohydrate intake, has been a popular choice for weight loss and managing certain medical conditions. However, its impact on heart health is a topic of ongoing research and debate. Here's a summary of the current understanding:  Potential Heart Health Benefits:  1. Weight Loss: The ketogenic diet can lead to significant weight loss, which can improve cardiovascular health by reducing the strain on the heart and decreasing risk factors like high blood pressure and high cholesterol levels.  2. Blood Sugar Control: For individuals with type 2 diabetes or insulin resistance, the ketogenic diet can help improve blood sugar levels and reduce insulin levels, potentially lowering the risk of heart disease.  3. Improved HDL Cholesterol: Some studies have shown that the ketogenic diet can increase high-density lipoprotein (HDL) cholesterol, which is considered the ""good"" cholesterol that helps remove other forms of cholesterol from the bloodstream.  Potential Heart Health Risks:  1. Elevated LDL Cholesterol: The ketogenic diet can increase low-density lipoprotein (LDL) cholesterol in some people, which is considered the ""bad"" cholesterol and is associated with an increased risk of heart disease.  2. Nutrient Deficiencies: Due to the restrictive nature of the diet, there is a risk of deficiencies in essential nutrients such as fiber, vitamins, and minerals, which are important for heart health.  3. Saturated Fat Intake: The diet often involves a high intake of saturated fats, which can contribute to an increase in LDL cholesterol levels and potentially increase heart disease risk if not carefully managed.  Individual Variation:  - Genetic Factors: Individual responses to the ketogenic diet can vary widely based on genetic factors, existing health conditions, and overall diet composition.  - Diet Quality: The impact on heart health can depend on the quality of fats consumed. Emphasizing unsaturated fats (like those found in avocados, nuts, and olive oil) over saturated fats (like those found in butter, red meat, and processed foods) may mitigate some of the risks.  Summary:  While the ketogenic diet can offer certain heart health benefits, particularly related to weight loss and blood sugar control, it also poses potential risks, especially related to cholesterol levels and nutrient deficiencies. If you are considering the ketogenic diet, it is important to:  1. Consult a Healthcare Professional: Before starting, discuss with a doctor or a registered dietitian to ensure it's appropriate for your individual health needs and conditions.  2. Monitor Health Markers: Regularly check cholesterol levels, blood pressure, and other heart health markers.  3. Choose Healthy Fats: Focus on incorporating healthy fats and avoiding excessive intake of saturated fats.  Given these considerations, the ketogenic diet may be heart-healthy for some individuals, but it requires careful planning and monitoring to mitigate potential risks. |
| **Llama3 Answer 1** | The keto diet, short for ketogenic diet, is a low-carb, high-fat diet that has gained popularity in recent years for its potential benefits in weight loss and improving blood sugar control. However, its impact on heart health is a topic of ongoing debate.  The Good:  1. Weight Loss: The keto diet can lead to significant weight loss, which is a major risk factor for heart disease.  2. Improved Blood Sugar Control: The diet can help regulate blood sugar levels and improve insulin sensitivity, which can reduce the risk of developing type 2 diabetes and cardiovascular disease.  3. Increased HDL Cholesterol: The keto diet has been shown to increase levels of high-density lipoprotein (HDL) cholesterol, the "good" cholesterol that helps remove excess cholesterol from the bloodstream and transport it to the liver for disposal.  4. Lower Triglycerides: The diet tends to lower triglyceride levels, which are a type of fat found in the blood that can increase the risk of heart disease when elevated.  The Not-So-Good:  1. Increased LDL Cholesterol: While the keto diet may increase HDL cholesterol, it can also increase levels of low-density lipoprotein (LDL) cholesterol, the "bad" cholesterol that can contribute to the buildup of plaque in the arteries and increase the risk of heart disease.  2. Higher Saturated Fat Intake: The keto diet often encourages high intake of saturated fats, which can increase cholesterol levels and heart disease risk when consumed in excess.  3. Potential Negative Impact on Blood Pressure: Some studies suggest that the keto diet may increase blood pressure in some individuals, which can increase the risk of heart disease and stroke.  4. Limited Long-Term Data: There is limited research on the long-term effects of the keto diet on heart health, which makes it difficult to draw firm conclusions.  The Verdict:  While the keto diet may have some benefits for heart health, such as weight loss and improved blood sugar control, its overall impact on heart health is still uncertain. The diet's high fat intake, particularly saturated fats, may increase cholesterol levels and heart disease risk in some individuals. Additionally, the limited long-term data available makes it difficult to assess the diet's overall effects on heart health.  Who Should Be Cautious:  1. Individuals with High Cholesterol or Heart Disease: Those with high cholesterol or pre-existing heart disease should consult with their healthcare provider before starting a keto diet, as it may not be suitable for everyone.  2. Those with Diabetes: While the keto diet can improve blood sugar control, it may not be suitable for everyone with diabetes, particularly those with type 1 diabetes or those who are taking certain medications.  3. Pregnant or Breastfeeding Women: The keto diet may not provide adequate nutrients for fetal development or breastfeeding, and its high fat intake may not be suitable for pregnant or breastfeeding women.  In conclusion, while the keto diet may have some benefits for heart health, its overall impact is still uncertain, and individuals should approach with caution, particularly those with pre-existing heart disease or other health conditions. It's essential to consult with a healthcare provider or a registered dietitian to determine the best diet for individual needs and health goals. |
| **Llama3 Answer 2** | The keto diet, short for ketogenic diet, is a high-fat, low-carbohydrate, moderate-protein diet that has gained popularity in recent years for its potential benefits on weight loss and blood sugar control. However, when it comes to heart health, the scientific consensus is more nuanced.  Potential benefits:  1. Weight loss: The keto diet can lead to significant weight loss, which is a major risk factor for heart disease.  2. Improved blood sugar control: The diet can help regulate blood sugar levels and improve insulin sensitivity, which can reduce the risk of developing type 2 diabetes, a major risk factor for heart disease.  3. Increased HDL cholesterol: The keto diet may increase levels of high-density lipoprotein (HDL) cholesterol, the "good" cholesterol that helps remove excess cholesterol from the bloodstream and transport it to the liver for excretion.  4. Reduced triglycerides: The diet may lower triglyceride levels, which are a type of fat in the blood that can increase the risk of heart disease when elevated.  Potential concerns:  1. Increased LDL cholesterol: The keto diet may increase levels of low-density lipoprotein (LDL) cholesterol, the "bad" cholesterol that can contribute to the buildup of plaque in the arteries, increasing the risk of heart disease.  2. Saturated fat intake: The diet is often high in saturated fats, which can increase LDL cholesterol levels and heart disease risk when consumed in excess.  3. Potential negative impact on blood vessels: Some studies suggest that the keto diet may have a negative impact on blood vessel function and increase the risk of cardiovascular disease, particularly in individuals with pre-existing heart conditions.  4. Limited long-term data: Most studies on the keto diet have been short-term, and there is limited data on its long-term effects on heart health.  Who may benefit from a keto diet:  1. Individuals with type 2 diabetes: The keto diet may be beneficial for individuals with type 2 diabetes, as it can help regulate blood sugar levels and improve insulin sensitivity.  2. Those with obesity: The diet may be effective for weight loss in individuals with obesity, which can reduce the risk of heart disease.  Who may not benefit from a keto diet:  1. Individuals with pre-existing heart conditions: Those with heart disease, high blood pressure, or high cholesterol may want to approach the keto diet with caution, as it may exacerbate these conditions.  2. Those with high LDL cholesterol: Individuals with high LDL cholesterol levels may want to avoid the keto diet, as it may increase their cholesterol levels further.  In conclusion, while the keto diet may have some potential benefits for heart health, it is not a one-size-fits-all solution. It is essential to consult with a healthcare professional or a registered dietitian to determine if the keto diet is appropriate for your individual needs and health status. |
| **Llama3 Answer 3** | The keto diet, short for ketogenic diet, is a low-carb, high-fat diet that has been popular for weight loss and improving blood sugar control. But is it heart healthy? The answer is not a simple yes or no.  The good:  1. Weight loss: The keto diet can lead to significant weight loss, which is a major risk factor for heart disease.  2. Improved blood sugar control: The diet can help regulate blood sugar levels and improve insulin sensitivity, which is beneficial for people with type 2 diabetes or prediabetes.  3. Increased HDL cholesterol: The keto diet tends to increase levels of high-density lipoprotein (HDL) cholesterol, the "good" cholesterol that helps remove excess cholesterol from the bloodstream.  4. Lower triglycerides: The diet can lead to a decrease in triglycerides, a type of fat found in the blood that's associated with increased cardiovascular risk.  The not-so-good:  1. Increased LDL cholesterol: The keto diet can also lead to an increase in low-density lipoprotein (LDL) cholesterol, the "bad" cholesterol that can contribute to the buildup of plaque in arteries.  2. Higher saturated fat intake: The diet often involves consuming large amounts of saturated fat, which can raise LDL cholesterol levels and increase cardiovascular risk.  3. Potential negative impact on blood pressure: Some studies suggest that the keto diet may lead to increased blood pressure, which is a major risk factor for heart disease.  4. Limited long-term studies: Many of the studies on the keto diet have been short-term, so it's unclear what the long-term effects on heart health might be.  Who may benefit from a keto diet:  1. People with type 2 diabetes or prediabetes: The diet can help improve blood sugar control and reduce the risk of cardiovascular disease.  2. Those with obesity or metabolic syndrome: The keto diet may lead to weight loss and improvements in metabolic health.  Who may not benefit from a keto diet:  1. People with high cholesterol or heart disease: The diet's potential to increase LDL cholesterol and saturated fat intake may not be suitable for those with existing heart health issues.  2. Those with kidney disease: The diet's high protein and fat content may put a strain on kidney function.  In conclusion:  While the keto diet may have some benefits for heart health, such as weight loss and improved blood sugar control, it's not necessarily a heart-healthy diet for everyone. The potential negative effects on LDL cholesterol, blood pressure, and saturated fat intake need to be carefully considered. If you're thinking about trying a keto diet, consult with a healthcare professional or a registered dietitian to determine if it's appropriate for your individual health needs and circumstances. |
| **LLaMA3+RAG model Answer 1** | According to the American Heart Association, the ketogenic diet is not considered heart-healthy. It falls into the fourth, or bottom, tier of diets, along with the Paleolithic diet, with a score of 31. This is because the ketogenic diet aligns poorly with heart-healthy eating guidelines and has not been shown to be any more effective for weight loss than less restrictive diets over the long term.  The ketogenic diet is high in fat, particularly saturated fats, which can increase the risk of heart disease. It also restricts consumption of fruits, grains, and legumes, which are rich in fiber, vitamins, and minerals essential for heart health. Furthermore, the ketogenic diet is highly restrictive and difficult for most people to stick with long-term, which can lead to unsustainable weight loss and potential negative health consequences.  The American Heart Association recommends a diet that is low in salt, added sugar, tropical oil, alcohol, and processed foods, and high in non-starchy vegetables, fruits, whole grains, and legumes. Proteins should come from plant sources, such as legumes, beans, or nuts, along with fish or seafood, lean poultry, and low-fat or fat-free dairy products. The DASH eating plan, which scored a perfect 100, is an example of a heart-healthy diet that aligns with these guidelines.  In addition to the provided context, other credible sources, such as the American College of Cardiology and the National Lipid Association, also recommend a diet that is low in saturated fats and high in fiber, fruits, and vegetables to reduce the risk of heart disease.  Sources:  American Heart Association. (2023). Here's How 10 Popular Diets Scored for Heart Health. Retrieved from <https://www.heart.org/en/news/2023/04/27/heres-how-10-popular-diets-scored-for-heart-health>  American Heart Association. (2023). 2023 AHA/ACC/AACVPR/AAPA/ABC/ACPM/ADA/AGS/APhA/ASPC/NLA/PCNA Guideline on the Management of Blood Cholesterol. Retrieved from <https://www.ahajournals.org/doi/full/10.1161/CIR.0000000000001146>  American College of Cardiology. (2020). ACC/AHA Guideline on the Primary Prevention of Cardiovascular Disease. Retrieved from <https://www.acc.org/latest-in-cardiology/clinical-guidelines/2020/09/04/14/41/acc-aha-guideline-on-the-primary-prevention-of-cardiovascular-disease>  National Lipid Association. (2020). National Lipid Association Recommendations for Patient-Centered Management of Dyslipidemia. Retrieved from <https://www.lipid.org/publications/2020-nla-recommendations-for-patient-centered-management-of-dyslipidemia> |
| **LLaMA3+RAG model Answer 2** | According to the American Heart Association, the ketogenic diet is not considered heart-healthy. In fact, it falls into the fourth, or bottom, tier of diets, along with the Paleolithic diet, with a score of 31 out of 100.  The ketogenic diet is described as being ""high in fat without limiting saturated fats,"" which aligns poorly with heart-healthy eating guidelines. This is concerning because a diet high in saturated fats can increase the risk of heart disease by raising total cholesterol and low-density lipoprotein (LDL) cholesterol levels (1)  Additionally, the ketogenic diet restricts the consumption of fruits, grains, and legumes, which are rich in fiber, vitamins, and minerals. This can lead to a diet that is low in essential nutrients and high in unhealthy fats.  The American Heart Association recommends a diet that is rich in whole, unprocessed foods, such as fruits, vegetables, whole grains, lean proteins, and healthy fats, like those found in nuts and avocados. This type of diet has been shown to lower the risk of heart disease, stroke, and other cardiovascular conditions (2).  In conclusion, based on the American Heart Association's evaluation, the ketogenic diet is not a heart-healthy diet due to its high fat content, lack of essential nutrients, and poor alignment with established dietary guidelines.  Sources:  1. American Heart Association. (2017). Dietary Fats and Cardiovascular Disease: A Presidential Advisory From the American Heart Association. Circulation, 136(3), e1-e23. doi: 10.1161/CIR.0000000000000510  2. American Heart Association. (2023). Here's How 10 Popular Diets Scored for Heart Health. Retrieved from <https://www.heart.org/en/news/2023/04/27/heres-how-10-popular-diets-scored-for-heart-health>  3. American Heart Association. (2023). 2023 Presidential Advisory: Dietary Guidance to Improve Cardiovascular Health. Circulation, 147(11), e539-e553. doi: 10.1161/CIR.0000000000001146 |
| **LLaMA3+RAG model Answer 3** | According to the American Heart Association, the ketogenic diet is not considered heart-healthy. In fact, it falls into the fourth, or bottom, tier of diets, with a score of 31 out of 100. This is because the ketogenic diet is high in fat without limiting saturated fats, which can be detrimental to heart health.  The ketogenic diet is also considered highly restrictive and difficult for most people to stick with long-term, which can lead to poor adherence and inconsistent results. Furthermore, the diet restricts consumption of fruits, grains, and legumes, which are essential for a balanced diet and can lead to eating less fiber and higher amounts of saturated fats.  The AHA's dietary guidance emphasizes the importance of a balanced diet that is low in salt, added sugar, tropical oil, alcohol, and processed foods, and high in non-starchy vegetables, fruits, whole grains, and legumes. The ketogenic diet does not align with these guidelines, and its restrictive nature can make it challenging for individuals to maintain a healthy and balanced diet.  It's worth noting that the AHA's assessment of the ketogenic diet is based on its potential impact on heart health, rather than its effectiveness for weight loss. While some studies have shown that the ketogenic diet can be effective for short-term weight loss, its long-term sustainability and overall health benefits are still a topic of debate.  Sources:  American Heart Association. (2023). Here's How 10 Popular Diets Scored for Heart Health. Retrieved from <https://www.heart.org/en/news/2023/04/27/heres-how-10-popular-diets-scored-for-heart-health>  American Heart Association. (2023). 2022 Dietary Guidance to Improve Cardiovascular Health. Retrieved from <https://www.ahajournals.org/doi/full/10.1161/CIR.0000000000001146>  Harvard T.H. Chan School of Public Health. (2024). Ketogenic Diet. Retrieved from <https://www.hsph.harvard.edu/nutritionsource/healthy-weight/keto-diet/>  National Lipid Association. (2020). Ketogenic Diet and Cardiovascular Health. Retrieved from <https://www.lipid.org/publications/keto-diet-and-cardiovascular-health> |
| **Question 6** | **What should I eat to lower my blood pressure?** |
| **Perplexity Answer 1** | To lower your blood pressure, focus on incorporating the following foods into your diet:  Fruits and vegetables: Eat plenty of fruits and vegetables, which are rich in potassium, magnesium, and antioxidants that can help lower blood pressure. Good options include leafy greens like spinach and kale, berries, bananas, oranges, beets, and tomatoes.[1][3][4]  Whole grains: Choose whole grains like oats, brown rice, quinoa, and whole wheat bread, which are high in fiber and can help regulate blood pressure.[1][3]  Lean proteins: Opt for lean proteins such as fish, poultry, beans, lentils, and nuts, which are low in saturated fat and can help manage weight, a key factor in controlling blood pressure.[1][3]  Low-fat dairy: Include low-fat dairy products like milk, yogurt, and cheese, which are good sources of calcium and can have a blood pressure-lowering effect.[1][2]  Healthy fats: Choose healthy fats like olive oil, avocados, and nuts, which are rich in unsaturated fats and can improve blood vessel function.[2]  It's also important to limit your intake of sodium, added sugars, and saturated fats, as these can contribute to high blood pressure. Drinking water, staying physically active, and maintaining a healthy weight can also help lower blood pressure.[1][3]  Citations:  [1] https://www.bhf.org.uk/informationsupport/heart-matters-magazine/nutrition/foods-that-lower-blood-pressure  [2] https://www.webmd.com/hypertension-high-blood-pressure/ss/slideshow-hypertension-low-bp-foods  [3] https://www.healthline.com/nutrition/foods-high-blood-pressure  [4] https://www.medicalnewstoday.com/articles/322284  [5] https://www.webmd.com/hypertension-high-blood-pressure/high-blood-pressure-diet |
| **Perplexity Answer 2** | To lower your blood pressure, you should focus on eating a diet rich in fruits, vegetables, whole grains, lean proteins, and low-fat dairy products. Here are some specific foods that can help:  1. Leafy green vegetables like spinach, kale, and collard greens are high in potassium, which helps counteract the effects of sodium and relax blood vessels[1][2].  2. Berries like blueberries, strawberries, and raspberries are rich in antioxidants that can help improve blood vessel function and reduce inflammation[4].  3. Fatty fish like salmon, mackerel, and sardines are excellent sources of omega-3 fatty acids, which have been shown to lower blood pressure[2].  4. Nuts and seeds like almonds, walnuts, and flaxseeds are high in magnesium, a mineral that helps regulate blood pressure[2].  5. Beans, lentils, and other legumes are packed with fiber and nutrients like potassium and magnesium, which can contribute to lower blood pressure[1][2].  6. Low-fat dairy products like yogurt and milk are good sources of calcium, which has been linked to lower blood pressure[2].  7. Whole grains like oats, quinoa, and brown rice are high in fiber and can help manage weight, which is important for blood pressure control[1].  It's also crucial to limit your intake of sodium, saturated fats, and added sugars, as these can contribute to high blood pressure[3]. Staying hydrated by drinking plenty of water can also help support healthy blood pressure levels[4].  Citations:  [1] https://www.bhf.org.uk/informationsupport/heart-matters-magazine/nutrition/foods-that-lower-blood-pressure  [2] https://www.webmd.com/hypertension-high-blood-pressure/ss/slideshow-hypertension-low-bp-foods  [3] https://www.healthline.com/nutrition/foods-high-blood-pressure  [4] https://www.medicalnewstoday.com/articles/322284  [5] https://www.webmd.com/hypertension-high-blood-pressure/high-blood-pressure-diet |
| **Perplexity Answer 3** | To lower your blood pressure, focus on incorporating the following foods into your diet:  Fruits and vegetables: Eat plenty of fruits and vegetables, which are rich in potassium, magnesium, and antioxidants that can help lower blood pressure. Good options include leafy greens like spinach and kale, berries, bananas, oranges, beets, and tomatoes.[1][3][4]  Whole grains: Choose whole grains like oats, brown rice, quinoa, and whole wheat bread, which are high in fiber and can help regulate blood pressure.[1][3]  Lean proteins: Opt for lean proteins such as fish, poultry, beans, lentils, and nuts, which are low in saturated fat and can help manage weight, a key factor in controlling blood pressure.[1][3]  Low-fat dairy: Include low-fat dairy products like milk, yogurt, and cheese, which are good sources of calcium and can have a blood pressure-lowering effect.[1][2]  Healthy fats: Choose healthy fats like olive oil, avocados, and nuts, which are rich in unsaturated fats and can improve blood vessel function.[2]  It's also important to limit your intake of sodium, added sugars, and saturated fats, as these can contribute to high blood pressure. Drinking water, staying physically active, and maintaining a healthy weight can also help lower blood pressure.[1][3]  Citations:  [1] https://www.bhf.org.uk/informationsupport/heart-matters-magazine/nutrition/foods-that-lower-blood-pressure  [2] https://www.webmd.com/hypertension-high-blood-pressure/ss/slideshow-hypertension-low-bp-foods  [3] https://www.healthline.com/nutrition/foods-high-blood-pressure  [4] https://www.medicalnewstoday.com/articles/322284  [5] https://www.webmd.com/hypertension-high-blood-pressure/high-blood-pressure-diet |
| **GPT4o Answer 1** | To lower your blood pressure, focus on a diet that is rich in nutrients, low in sodium, and promotes overall heart health. Here are some key dietary recommendations:  1. Increase Intake of Fruits and Vegetables:  - Aim for a variety of colorful fruits and vegetables. They are rich in potassium, magnesium, and fiber.  - Examples: Berries, apples, bananas, oranges, leafy greens, carrots, bell peppers.  2. Choose Whole Grains:  - Whole grains are high in fiber and can help reduce blood pressure.  - Examples: Brown rice, quinoa, whole wheat bread, oatmeal.  3. Include Low-Fat Dairy Products:  - Low-fat or fat-free dairy products are good sources of calcium and can help lower blood pressure.  - Examples: Skim milk, low-fat yogurt, low-fat cheese.  4. Opt for Lean Proteins:  - Lean meats, poultry, fish, and plant-based proteins are better choices than fatty cuts of meat.  - Examples: Skinless chicken, fish (especially fatty fish like salmon and mackerel), legumes, tofu.  5. Eat Nuts, Seeds, and Legumes:  - These foods are good sources of magnesium and healthy fats.  - Examples: Almonds, walnuts, flaxseeds, lentils, chickpeas.  6. Reduce Sodium Intake:  - High sodium intake can raise blood pressure. Aim to consume less than 2,300 mg of sodium per day, and ideally less than 1,500 mg.  - Tips: Avoid processed foods, read labels for sodium content, and cook with herbs and spices instead of salt.  7. Limit Sweets and Sugary Beverages:  - Reducing sugar intake can help maintain a healthy weight and blood pressure.  - Examples: Avoid soda, candy, and desserts with added sugars.  8. Choose Healthy Fats:  - Focus on unsaturated fats found in fish, nuts, seeds, and olive oil. Avoid trans fats and limit saturated fats.  - Examples: Olive oil, avocados, salmon, walnuts.  9. Limit Alcohol:  - Drinking alcohol in moderation, if at all, can help manage blood pressure. This means up to one drink per day for women and up to two drinks per day for men.  10. Stay Hydrated:  - Drinking plenty of water throughout the day supports overall health and can help manage blood pressure.  Example Meal Plan  Breakfast:  - Oatmeal topped with fresh berries and a handful of nuts.  - A glass of skim milk or a serving of low-fat yogurt.  Lunch:  - A mixed greens salad with chickpeas, tomatoes, cucumbers, and a vinaigrette dressing.  - A whole grain roll or slice of whole wheat bread.  - A piece of fruit, such as an apple or banana.  Dinner:  - Grilled salmon or skinless chicken breast.  - Steamed broccoli and sweet potatoes.  - Quinoa or brown rice.  Snacks:  - Fresh fruit (e.g., an orange or a handful of grapes).  - Raw vegetables with hummus.  - A small handful of unsalted nuts.  Remember to consult with a healthcare provider or a registered dietitian to tailor these recommendations to your specific health needs and conditions. |
| **GPT4o Answer 2** | To lower your blood pressure, consider incorporating the following foods and dietary practices into your diet:  Foods to Lower Blood Pressure  1. Fruits and Vegetables: Rich in potassium, fiber, and antioxidants.  - Bananas: High in potassium which helps balance sodium levels.  - Berries: Especially blueberries and strawberries, which contain flavonoids.  - Leafy Greens: Spinach, kale, and other greens are rich in nitrates.  - Beets: High in nitrates which help dilate blood vessels.  2. Whole Grains: Provide fiber and nutrients that improve heart health.  - Oats: Contain beta-glucan, which helps lower cholesterol.  - Quinoa: A complete protein and high in magnesium.  3. Lean Proteins: Necessary for overall health without adding saturated fats.  - Fish: Especially fatty fish like salmon, rich in omega-3 fatty acids.  - Poultry: Skinless chicken or turkey.  4. Low-Fat Dairy: Provides calcium and vitamin D without high levels of saturated fat.  - Yogurt: Choose low-fat or Greek yogurt.  - Milk: Opt for low-fat or skim milk.  5. Nuts and Seeds: Good sources of healthy fats and magnesium.  - Almonds: High in magnesium and healthy fats.  - Flaxseeds: Rich in omega-3 fatty acids and fiber.  6. Legumes: High in protein, fiber, and potassium.  - Lentils: Excellent source of plant-based protein and fiber.  - Beans: Black beans, kidney beans, and chickpeas.  7. Healthy Fats: Important for heart health.  - Olive Oil: Rich in monounsaturated fats.  - Avocados: Provide potassium and healthy fats.  Dietary Practices  1. Reduce Sodium Intake: Limit salt to less than 2,300 mg per day, ideally around 1,500 mg.  - Avoid processed and packaged foods.  - Read labels to check sodium content.  2. Increase Potassium Intake: Helps balance the effects of sodium.  - Focus on fruits, vegetables, and legumes.  3. Moderate Alcohol Consumption: Excessive drinking can raise blood pressure.  - Limit to one drink per day for women and two for men.  4. Limit Caffeine: High levels of caffeine can cause a short-term spike in blood pressure.  - Monitor your response to caffeinated beverages.  5. Eat More Fiber: Helps improve blood pressure and cholesterol levels.  - Focus on whole grains, fruits, vegetables, and legumes.  Sample Meal Plan  Breakfast  - Oatmeal with fresh berries and a banana.  - Low-fat yogurt.  Lunch  - Spinach and kale salad with cherry tomatoes, cucumbers, and chickpeas, dressed with olive oil and lemon.  - A small serving of quinoa.  Snack  - A handful of almonds or a piece of fruit.  Dinner  - Grilled salmon with a side of steamed broccoli and brown rice.  - Mixed green salad with avocado slices.  Snack  - A small bowl of Greek yogurt with flaxseeds.  By consistently including these foods and practices in your diet, you can help lower your blood pressure and improve your overall heart health. Additionally, consider maintaining a healthy weight, exercising regularly, managing stress, and avoiding smoking to further support your blood pressure management. |
| **GPT4o Answer 3** | To help lower your blood pressure, you should focus on a diet that is rich in nutrients and low in salt, saturated fat, and cholesterol. Here are some dietary recommendations:  1. Fruits and Vegetables: Aim for at least 5 servings of fruits and vegetables per day. These are high in potassium, which can help balance out the negative effects of sodium. Examples include bananas, oranges, spinach, and broccoli.  2. Whole Grains: Incorporate whole grains into your diet, such as whole wheat bread, brown rice, quinoa, and oatmeal. These are high in fiber and can help maintain healthy blood pressure levels.  3. Lean Proteins: Choose lean protein sources like skinless poultry, fish, beans, and legumes. Fish rich in omega-3 fatty acids, such as salmon and mackerel, are particularly beneficial.  4. Low-Fat Dairy: Opt for low-fat or fat-free dairy products like milk, yogurt, and cheese. These are good sources of calcium and protein without the added saturated fat.  5. Nuts and Seeds: Include unsalted nuts and seeds in your diet. Almonds, walnuts, flaxseeds, and chia seeds are great choices.  6. Healthy Fats: Use healthy fats such as olive oil, avocado, and nuts. Avoid trans fats and reduce saturated fat intake.  7. Limit Sodium: Reduce your sodium intake by avoiding processed and packaged foods. Cook meals at home using fresh ingredients and herbs and spices for flavor instead of salt.  8. Stay Hydrated: Drink plenty of water throughout the day. Limit sugary beverages and reduce caffeine and alcohol intake.  Sample Meal Plan  Breakfast:  - Oatmeal topped with fresh berries and a handful of nuts  - A glass of low-fat milk or a small serving of Greek yogurt  Lunch:  - A large salad with mixed greens, tomatoes, cucumbers, chickpeas, and grilled chicken, dressed with olive oil and lemon juice  - A side of whole grain bread  Snack:  - A banana or an apple with a small handful of almonds  Dinner:  - Baked salmon with a side of quinoa and steamed broccoli  - A mixed green salad with olive oil and vinegar dressing  Snack:  - Low-fat yogurt with a sprinkle of flaxseeds  Additional Tips  - Exercise Regularly: Combine your healthy diet with regular physical activity. Aim for at least 150 minutes of moderate exercise or 75 minutes of vigorous exercise per week.  - Monitor Your Blood Pressure: Keep track of your blood pressure readings to see how your diet and lifestyle changes are impacting your health.  - Consult with a Healthcare Professional: Before making significant changes to your diet or exercise routine, consult with a healthcare provider, especially if you have existing health conditions.  Adopting these dietary changes can help you manage and potentially lower your blood pressure over time. |
| **Llama 3 Answer 1** | Eating a healthy diet can play a significant role in helping to lower blood pressure. Here are some foods and nutrients that can help:  Foods that can help lower blood pressure:  1. Leafy Greens: Spinach, kale, collard greens, and other leafy greens are rich in potassium, calcium, and magnesium, which can help lower blood pressure.  2. Berries: Berries like blueberries, strawberries, and raspberries are rich in flavonoids, which can help improve blood vessel function and lower blood pressure.  3. Beets: Beets are a rich source of nitrates, which can help relax blood vessels and lower blood pressure.  4. Olive Oil: The monounsaturated fats in olive oil can help lower blood pressure by improving blood vessel function.  5. Fatty Fish: Fatty fish like salmon, tuna, and mackerel are rich in omega-3 fatty acids, which can help reduce inflammation and improve blood vessel function.  6. Nuts and Seeds: Almonds, walnuts, chia seeds, and flaxseeds are rich in magnesium, potassium, and healthy fats, which can help lower blood pressure.  7. Fat-Free or Low-Fat Dairy: Milk, cheese, and yogurt are rich in calcium, potassium, and magnesium, which can help lower blood pressure.  8. Whole Grains: Whole grains like brown rice, quinoa, and whole-wheat bread can help lower blood pressure by providing fiber, potassium, and magnesium.  9. Legumes: Legumes like lentils, chickpeas, and black beans are rich in potassium, fiber, and protein, which can help lower blood pressure.  10. Tea: Regular tea consumption, especially green tea, has been shown to help lower blood pressure.  Nutrients that can help lower blood pressure:  1. Potassium: Aim for 4,700 mg of potassium per day from foods like bananas, avocados, and sweet potatoes.  2. Calcium: Aim for 1,000-1,200 mg of calcium per day from foods like dairy products, leafy greens, and fortified plant-based milk.  3. Magnesium: Aim for 400-420 mg of magnesium per day from foods like dark leafy greens, nuts, and whole grains.  4. Omega-3 Fatty Acids: Aim for 250-500 mg of omega-3 fatty acids per day from fatty fish, nuts, and seeds.  5. Fiber: Aim for 25-30 grams of fiber per day from whole grains, fruits, and vegetables.  Dietary Approaches:  1. DASH Diet: The Dietary Approaches to Stop Hypertension (DASH) diet is a well-studied eating plan that focuses on whole grains, fruits, vegetables, lean protein, and low-fat dairy.  2. Mediterranean Diet: The Mediterranean diet emphasizes whole grains, fruits, vegetables, lean protein, and healthy fats, such as those found in olive oil.  Additional Tips:  1. Limit Sodium: Consume no more than 2,300 mg of sodium per day, and ideally no more than 1,500 mg per day if you have high blood pressure or are at risk for it.  2. Stay Hydrated: Drink plenty of water and limit sugary drinks.  3. Cook at Home: Cooking at home allows you to control the ingredients and portion sizes, which can help you make healthier choices.  4. Consult a Healthcare Professional: If you have high blood pressure or are at risk for it, consult with a healthcare professional or a registered dietitian to create a personalized eating plan.  Remember, a healthy diet is just one aspect of maintaining healthy blood pressure. Regular physical activity, stress management, and adequate sleep are also important for overall cardiovascular health. |
| **Llama 3 Answer 2** | Eating a healthy diet can play a significant role in helping to lower blood pressure. Here are some foods and nutrients that can help:  1. Leafy Greens: Spinach, kale, collard greens, and other leafy greens are rich in potassium, calcium, and magnesium, which can help lower blood pressure.  2. Berries: Berries like blueberries, strawberries, and raspberries are rich in flavonoids, which have been shown to help lower blood pressure and improve cardiovascular health.  3. Beets: Beets are a rich source of nitrates, which can help relax blood vessels and lower blood pressure.  4. Olive Oil: The monounsaturated fats in olive oil can help lower blood pressure by reducing inflammation and improving blood vessel function.  5. Fatty Fish: Fatty fish like salmon, tuna, and mackerel are rich in omega-3 fatty acids, which can help reduce inflammation and improve blood vessel function.  6. Skim Milk and Low-Fat Dairy: Skim milk and low-fat dairy products are rich in potassium, calcium, and magnesium, which can help lower blood pressure.  7. Whole Grains: Whole grains like brown rice, quinoa, and whole wheat bread can help lower blood pressure by reducing inflammation and improving blood vessel function.  8. Pumpkin Seeds: Pumpkin seeds are a rich source of magnesium, potassium, and zinc, which can help lower blood pressure.  9. Avocados: Avocados are rich in potassium, folate, and healthy fats, which can help lower blood pressure and improve cardiovascular health.  10. Herbs and Spices: Herbs like basil, garlic, and cayenne pepper, and spices like cinnamon and turmeric, have been shown to have blood pressure-lowering effects.  In addition to incorporating these foods into your diet, here are some general dietary recommendations to help lower blood pressure:  * Reduce sodium intake: Limit sodium to less than 2,300 milligrams per day. If you have high blood pressure, aim for no more than 1,500!milligrams per day.  * Increase potassium intake: Aim for 4,700 milligrams of potassium per day.  * Choose low-fat dairy products: Select low-fat or fat-free dairy products to reduce saturated fat intake.  * Limit processed and packaged foods: Processed and packaged foods are often high in sodium, added sugars, and unhealthy fats.  * Stay hydrated: Drink plenty of water and limit sugary drinks.  Remember, a healthy diet is just one aspect of managing blood pressure. Regular exercise, stress management, and adequate sleep are also important for maintaining healthy blood pressure.  Consult with your healthcare provider or a registered dietitian to develop a personalized diet plan that's tailored to your specific needs and health goals. |
| **Llama 3 Answer 3** | Eating a healthy diet can play a significant role in helping to lower blood pressure. Here are some foods and nutrients that can help:  1. Leafy Greens: Leafy greens like spinach, kale, collard greens, and lettuce are rich in potassium, calcium, and magnesium, which can help lower blood pressure.  2. Berries: Berries like blueberries, strawberries, and raspberries are rich in flavonoids, which have been shown to improve blood vessel function and lower blood pressure.  3. Beets: Beets are a rich source of nitrates, which can help relax blood vessels and lower blood pressure.  4. Omega-3 Fatty Acids: Fatty fish like salmon, tuna, and mackerel are rich in omega-3 fatty acids, which can help reduce inflammation and improve blood vessel function.  5. Oatmeal: Oatmeal is rich in fiber, which can help lower blood pressure by improving blood vessel function and reducing inflammation.  6. Bananas: Bananas are a rich source of potassium, which can help lower blood pressure by balancing out the effects of sodium in the body.  7. Avocados: Avocados are rich in potassium, fiber, and healthy fats, which can help lower blood pressure and improve overall heart health.  8. Fatty Fish: Fatty fish like salmon, tuna, and mackerel are rich in omega-3 fatty acids and potassium, which can help lower blood pressure.  9. Sweet Potatoes: Sweet potatoes are rich in potassium, fiber, and vitamins A and C, which can help lower blood pressure and improve overall heart health.  10. Low-Sodium Foods: Limiting sodium intake is essential for lowering blood pressure. Choose low-sodium options for processed foods, and season with herbs and spices instead of salt.  11. Calcium-Rich Foods: Calcium can help lower blood pressure by improving blood vessel function. Good sources of calcium include dairy products, leafy greens, and fortified plant-based milk.  12. Magnesium-Rich Foods: Magnesium can help lower blood pressure by relaxing blood vessels and improving blood flow. Good sources of magnesium include dark leafy greens, nuts, and whole grains.  Dietary Patterns: In addition to incorporating these foods into your diet, following a specific dietary pattern can also help lower blood pressure. Some examples include:  * The DASH (Dietary Approaches to Stop Hypertension) diet, which emphasizes whole grains, fruits, vegetables, lean protein, and low-fat dairy.  * The Mediterranean diet, which emphasizes whole grains, fruits, vegetables, lean protein, and healthy fats.  * The American Heart Association's (AHA) Heart-Healthy Diet, which emphasizes whole grains, fruits, vegetables, lean protein, and low-fat dairy.  Remember: While diet plays a significant role in lowering blood pressure, it's also important to maintain a healthy weight, exercise regularly, and manage stress to achieve optimal blood pressure control. Consult with your healthcare provider or a registered dietitian to develop a personalized plan. |
| **LLaMA3+RAG model Answer 1** | To lower your blood pressure, it's essential to adopt a heart-healthy diet that focuses on consuming nutrient-dense foods while limiting or avoiding certain foods that can exacerbate high blood pressure. Here are the key dietary recommendations to help you achieve your goal:  Eat plenty of:  1. Fruits: Aim for a variety of fruits, such as berries, citrus fruits, apples, and bananas, which are rich in potassium, fiber, and antioxidants.  2. Vegetables: Include a wide range of colorful vegetables like leafy greens, bell peppers, carrots, and tomatoes, which are rich in potassium, fiber, and vitamins.  3. Fat-free or low-fat dairy products: Choose low-fat or fat-free milk, yogurt, and cheese to reduce saturated fat intake.  4. Whole grains: Incorporate whole grains like brown rice, quinoa, whole-wheat bread, and whole-grain pasta to increase fiber and nutrient intake.  5. Skinless poultry and fish: Opt for lean protein sources like chicken, turkey, and fatty fish like salmon, which are rich in omega-3 fatty acids.  6. Nuts and legumes: Include nuts like almonds, walnuts, and pecans, as well as legumes like lentils, chickpeas, and black beans, which are rich in healthy fats, protein, and fiber.  7. Non-tropical vegetable oils: Use oils like olive oil, canola oil, and avocado oil for cooking and dressings.  Limit or avoid:  1. Saturated and trans fats: Restrict or avoid foods high in saturated fats, such as red meat, full-fat dairy products, and processed meats. Also, limit trans fats found in processed foods, like baked goods and fried foods.  2. Alcoholic beverages: Drink no more than one to two drinks per day, and avoid binge drinking.  3. Sodium (salt): According to the American Heart Association, a heart-healthy diet recommends limiting daily sodium intake to no more than 2,300 milligrams (mg) per day, ideally aim to consume less than 1,500 milligrams of sodium per day.  4. Fatty and processed meats: Limit or avoid foods like hot dogs, sausages, bacon, and processed meats, which are high in sodium and saturated fats.  5. Items with added sugar: Restrict or avoid sugary drinks, baked goods, and processed snacks that contain added sugars.  Additional tips:  1. Maintain a healthy weight: Losing weight, if you're overweight or obese, can help reduce blood pressure.  2. Get active: Aim for at least 150 minutes of aerobic exercise or two sessions of resistance exercises per week to help lower blood pressure.  3. Limit smoking: Avoid smoking or quit smoking to reduce the risk of heart disease and high blood pressure.  By following these dietary recommendations and incorporating a heart-healthy lifestyle, you can help lower your blood pressure and reduce your risk of heart attack, stroke, and other health threats.  Sources:  1. American Heart Association. (2024). Five Simple Steps to Control Your Blood Pressure. Retrieved from <https://www.heart.org/en/health-topics/high-blood-pressure/the-facts-about-high-blood-pressure/five-simple-steps-to-control-your-blood-pressure>  2. American Heart Association. (2024). Managing Blood Pressure with a Heart-Healthy Diet. Retrieved from <https://www.heart.org/en/health-topics/high-blood-pressure/changes-you-can-make-to-manage-high-blood-pressure/managing-blood-pressure-with-a-heart-healthy-diet> |
| **LLaMA3+RAG model Answer 2** | To lower your blood pressure, it's essential to adopt a heart-healthy eating plan that focuses on consuming nutrient-dense foods while limiting or avoiding foods that can exacerbate high blood pressure. Here are the key dietary recommendations to help you achieve this goal:  Foods to Emphasize:  1. Fruits: Aim for a variety of fruits, such as berries, citrus fruits, apples, and bananas, which are rich in potassium, fiber, and antioxidants.  2. Vegetables: Include a range of colorful vegetables like leafy greens, bell peppers, carrots, and tomatoes, which are rich in potassium, fiber, and vitamins.  3. Whole Grains: Choose whole grains like brown rice, quinoa, whole wheat bread, and whole grain pasta, which are rich in fiber, vitamins, and minerals.  4. Low-Fat Dairy Products: Opt for low-fat or fat-free dairy products like milk, yogurt, and cheese, which are rich in calcium, protein, and potassium.  5. Skinless Poultry and Fish: Include lean protein sources like chicken, turkey, and fish (like salmon, tuna, and mackerel), which are rich in protein, omega-3 fatty acids, and potassium.  6. Nuts and Legumes: Nuts like almonds, walnuts, and pecans, as well as legumes like lentils, chickpeas, and black beans, are rich in healthy fats, protein, and fiber.  7. Non-Tropical Vegetable Oils: Use heart-healthy oils like olive oil, canola oil, and avocado oil for cooking and dressing salads.  Foods to Limit or Avoid:  1. Saturated and Trans Fats: Limit or avoid foods high in saturated fats, such as red meat, full-fat dairy products, and processed meats like hot dogs and sausages. Also, avoid trans fats found in processed foods like baked goods, fried foods, and snack foods.  2. Alcoholic Beverages: Limit your daily alcohol intake to one drink for women and two drinks for men.  3. Sodium (Salt): According to the American Heart Association, a heart-healthy diet recommends limiting daily sodium intake to no more than 2,300 milligrams (mg) per day, ideally aim to consume less than 1,500 milligrams of sodium per day. Limit processed and packaged foods, which are often high in sodium.  4. Fatty and Processed Meats: Limit or avoid foods like bacon, sausage, and deli meats, which are high in sodium and saturated fats.  5. Items with Added Sugar: Limit or avoid foods and beverages with added sugars, such as sugary drinks, baked goods, and candy.  Additional Tips:  1. Stay Hydrated: Drink plenty of water and limit sugary drinks.  2. Cook at Home: Prepare meals at home using heart-healthy recipes to control the ingredients and portion sizes.  3. Plan Ahead: Work with your household members to plan meals and make healthy choices when dining out.  4. Monitor Your Progress: Regularly track your blood pressure and adjust your diet accordingly.  By following these dietary recommendations, you'll be well on your way to adopting a heart-healthy eating plan that can help lower your blood pressure and reduce your risk of heart disease.  Sources:  1. American Heart Association. (2024). Five Simple Steps to Control Your Blood Pressure. Retrieved from <https://www.heart.org/en/health-topics/high-blood-pressure/the-facts-about-high-blood-pressure/five-simple-steps-to-control-your-blood-pressure>  2. American Heart Association. (2024). Managing Blood Pressure with a Heart-Healthy Diet. Retrieved from <https://www.heart.org/en/health-topics/high-blood-pressure/changes-you-can-make-to-manage-high-blood-pressure/managing-blood-pressure-with-a-heart-healthy-diet> |
| **LLaMA3+RAG model Answer 3** | To lower your blood pressure, it is essential to adopt a heart-healthy diet that is rich in nutrients and low in sodium, saturated fats, and added sugars. Here are some specific food groups and nutrients that can help:  Fruits and Vegetables: Aim for at least 5 servings a day, including a variety of colors to ensure you get a range of vitamins, minerals, and antioxidants. Some examples include berries, citrus fruits, leafy greens, bell peppers, and carrots.  Whole Grains: Choose whole, unprocessed grains like brown rice, quinoa, whole wheat bread, and whole grain pasta to increase your fiber intake and reduce blood pressure.  Low-Fat Dairy Products: Opt for fat-free or low-fat dairy products like milk, yogurt, and cheese to reduce saturated fat intake and support blood pressure management.  Skinless Poultry and Fish: Include lean protein sources like chicken, turkey, and fish (like salmon, tuna, and mackerel) in your diet to reduce saturated fat intake and support heart health.  Nuts and Legumes: Almonds, walnuts, and legumes like lentils, chickpeas, and black beans are rich in healthy fats, protein, and fiber, making them an excellent addition to a heart-healthy diet.  Non-Tropical Vegetable Oils: Use heart-healthy oils like olive oil, canola oil, and avocado oil for cooking and dressing salads to reduce saturated fat intake.  Limit or Avoid:  Saturated and Trans Fats: Restrict or avoid foods high in saturated fats like red meat, full-fat dairy products, and processed meats. Also, limit foods with trans fats, often found in processed snacks and fried foods.  Alcoholic Beverages: Drink no more than one to two drinks a day (one for most women, two for most men) to avoid increasing blood pressure.  Sodium (Salt): According to the American Heart Association, a heart-healthy diet recommends limiting daily sodium intake to no more than 2,300 milligrams (mg) per day, ideally aim for less than 1,500 milligrams of sodium per day to lower blood pressure.  Fatty and Processed Meats: Limit or avoid foods like hot dogs, sausages, bacon, and processed meats to reduce saturated fat and sodium intake.  Items with Added Sugar: Restrict or avoid sugary drinks, baked goods, and sweets to reduce blood pressure and support overall health.  Additional Tips:  Maintain a healthy weight by balancing your calorie intake with physical activity to reduce blood pressure.  Get active by aiming for at least 150 minutes of aerobic exercise and two sessions of resistance exercises per week.  Limit smoking or quit altogether to reduce blood pressure and support overall health.  By incorporating these heart-healthy eating habits into your lifestyle, you can help lower your blood pressure, reduce your risk of heart attack, stroke, and other health threats, and improve your overall well-being.  Sources:  1. American Heart Association. (2024). Five Simple Steps to Control Your Blood Pressure. Retrieved from <https://www.heart.org/en/health-topics/high-blood-pressure/the-facts-about-high-blood-pressure/five-simple-steps-to-control-your-blood-pressure>  2. American Heart Association. (2024). Managing Blood Pressure with a Heart-Healthy Diet. Retrieved from <https://www.heart.org/en/health-topics/high-blood-pressure/changes-you-can-make-to-manage-high-blood-pressure/managing-blood-pressure-with-a-heart-healthy-diet> |
| **Question 7** | **What kinds of carbohydrates should I be eating as part of a heart healthy diet?** |
| **Perplexity Answer 1** | Here are the key types of carbohydrates to focus on in a heart healthy diet:  Complex carbohydrates: These are the healthiest carb choices and should make up the bulk of your carb intake. Examples include:  - Whole grains like whole wheat bread, oats, brown rice, quinoa, barley[1][3][4]  - Fruits and vegetables, especially those with the skin on[1][3]  - Legumes like beans, peas and lentils[1][4]  Complex carbs are higher in fiber and other beneficial nutrients compared to refined carbs. The fiber helps regulate blood sugar, lower cholesterol, and keep you feeling full longer[2].  Limit refined and simple carbohydrates: These are carbs that have been stripped of nutrients and fiber. Examples include:  - White bread, pastries, cookies, cakes[1][3]  - White rice, regular pasta[4]  - Sugary foods and drinks like candy, soda, fruit juices[1][3]  Eating too many refined carbs can spike blood sugar and contribute to weight gain, diabetes, and heart disease risk[2][3]. Limit these to occasional treats.  Focus on getting 25-30 grams of fiber per day from whole food sources like fruits, vegetables, whole grains and legumes[2]. Soluble fiber like that found in oats, barley, beans and berries is especially beneficial for heart health[3].  The American Heart Association recommends getting 45-65% of your total daily calories from carbohydrates, with an emphasis on complex carbs and fiber[4]. Choosing healthy carbs is essential for supporting overall health.  Citations:  [1] https://www.mskcc.org/experience/patient-support/nutrition-cancer/diet-plans-cancer/cardiac-diet  [2] https://my.clevelandclinic.org/health/articles/15416-carbohydrates  [3] https://www.eatingwell.com/article/7938733/healthiest-carbs-you-should-be-buying-according-to-a-dietitian/  [4] https://openheart.net/carbohydrates/  [5] https://www.healthline.com/nutrition/12-healthy-high-carb-foods |
| **Perplexity Answer 2** | To follow a heart-healthy diet, it's best to focus on complex carbohydrates from whole, unprocessed sources:  - Whole grains like whole wheat bread, oats, brown rice, and barley[1][4][5]  - Fruits and vegetables[4][5]  - Legumes like beans, peas, and lentils[4][5]  These complex carbs are high in fiber, vitamins, minerals, and antioxidants. They are digested more slowly, helping to keep blood sugar and insulin levels stable[4].  In contrast, simple or refined carbs like white bread, pastries, sodas, and sweets should be limited. These provide calories without much nutrition and can contribute to weight gain, diabetes, and heart disease risk[3][5].  The American Heart Association recommends getting 45-65% of calories from carbohydrates, with an emphasis on whole, fiber-rich sources[5]. For a 2000 calorie diet, this would be about 225-325 grams of carbs per day[4].  Portion control is also important. Eating too many carbs, even from healthy sources, can lead to weight gain if total calories are excessive[5]. Pairing carbs with protein and healthy fats can help with satiety.  The bottom line is to choose carbs wisely by favoring whole, minimally processed options and limiting added sugars and refined grains. This will provide the carbs your body needs while supporting heart health.  Citations:  [1] https://www.heartandstroke.ca/articles/a-sensible-approach-to-carbs  [2] https://muschealth.org/-/sm/health/patients-visitors/f/dining-suggestions/adult-consistent-carb-heart-healthy-menu.ashx  [3] https://www.ncbi.nlm.nih.gov/pmc/articles/PMC10096555/  [4] https://openheart.net/carbohydrates/  [5] <https://www.heart.org/en/news/2023/08/11/confused-about-carbs-this-might-help> |
| **Perplexity Answer 3** | Here are the key types of carbohydrates to focus on in a heart healthy diet:  Complex carbohydrates: These are the healthiest carb choices and should make up the bulk of your carb intake. Examples include:  - Whole grains like whole-wheat bread, oats, brown rice, quinoa, barley[1][3][4]  - Fruits with skin[3]  - Vegetables[1][3][4]  - Legumes[1][3][4]  Complex carbs are higher in fiber and other nutrients compared to refined carbs. They digest more slowly, helping to keep blood sugar stable and providing lasting energy and fullness[2][3].  Fiber: Aim for 25-30 grams of fiber per day from foods like fruits, vegetables, whole grains, and legumes[2]. Fiber helps regulate blood sugar, lower cholesterol, and aids digestion[2].  Limit refined and simple carbs: Foods high in added sugars and refined grains like white bread, pastries, sodas, and candy provide calories without much nutrition[1][3]. Eating too many can spike blood sugar and contribute to weight gain[2][3].  The American Heart Association recommends getting 55-60% of calories from carbs, with a focus on complex carbs and fiber[4]. Choosing nutrient-dense carb sources from whole foods is key for heart health.  Citations:  [1] https://www.mskcc.org/experience/patient-support/nutrition-cancer/diet-plans-cancer/cardiac-diet  [2] https://my.clevelandclinic.org/health/articles/15416-carbohydrates  [3] https://www.eatingwell.com/article/7938733/healthiest-carbs-you-should-be-buying-according-to-a-dietitian/  [4] https://openheart.net/carbohydrates/  [5] https://www.healthline.com/nutrition/12-healthy-high-carb-foods |
| **GPT4o Answer 1** | A heart-healthy diet emphasizes the consumption of certain types of carbohydrates while minimizing others. Here are the key types of carbohydrates you should focus on:  Whole Grains  - Oats: Rich in soluble fiber, which can help lower cholesterol.  - Brown Rice: Contains more nutrients and fiber compared to white rice.  - Quinoa: High in protein and fiber, also gluten-free.  - Whole Wheat: Includes whole wheat bread, pasta, and flour.  Fruits and Vegetables  - Berries: Such as blueberries, strawberries, and raspberries, which are high in fiber and antioxidants.  - Apples and Pears: Rich in soluble fiber.  - Leafy Greens: Spinach, kale, and Swiss chard.  - Root Vegetables: Sweet potatoes and carrots, which provide fiber and essential nutrients.  Legumes  - Beans: Such as black beans, lentils, chickpeas, and kidney beans, which are high in fiber and protein.  - Lentils: Particularly high in protein and fiber, aiding in cholesterol control.  Nuts and Seeds  - Chia Seeds: High in omega-3 fatty acids and fiber.  - Flaxseeds: Also rich in omega-3s and fiber.  - Almonds and Walnuts: Provide healthy fats and fiber.  Fiber-Rich Foods  - Psyllium: A type of soluble fiber often used as a supplement to aid digestion and lower cholesterol.  - Barley: High in soluble fiber, which helps reduce cholesterol levels.  Minimizing Refined Carbohydrates  - Avoid or limit: White bread, white rice, pastries, sugary cereals, and other refined grains, as they can spike blood sugar levels and contribute to heart disease.  General Tips  - Focus on Fiber: Aim for high-fiber options (whole fruits, vegetables, whole grains, legumes) that help reduce cholesterol and improve heart health.  - Low Glycemic Index: Choose carbohydrates with a low glycemic index (whole grains, most fruits, vegetables) to maintain stable blood sugar levels.  - Limit Sugary Foods: Reduce intake of foods high in added sugars (sodas, candies, desserts) which can contribute to heart disease.  Example of a Heart-Healthy Carb-Rich Meal  - Breakfast: Oatmeal topped with fresh berries and a sprinkle of chia seeds.  - Lunch: Quinoa salad with mixed greens, chickpeas, cherry tomatoes, and a lemon-tahini dressing.  - Dinner: Grilled salmon with a side of roasted sweet potatoes and steamed broccoli.  - Snacks: An apple with almond butter, or a handful of mixed nuts and seeds.  Incorporating these types of carbohydrates into your diet can help maintain heart health, manage blood sugar levels, and provide essential nutrients for overall well-being |
| **GPT4o Answer 2** | In a heart-healthy diet, it is important to focus on the quality and type of carbohydrates you consume. Here are some guidelines on the kinds of carbohydrates you should be eating:  1. Whole Grains  - Examples: Whole wheat, brown rice, quinoa, barley, oats, bulgur, and whole grain breads and pastas.  - Benefits: Whole grains are rich in fiber, which helps reduce cholesterol levels and improve heart health.  2. Fruits and Vegetables  - Examples: Apples, berries, oranges, bananas, spinach, broccoli, carrots, and sweet potatoes.  - Benefits: These foods are high in vitamins, minerals, fiber, and antioxidants, all of which contribute to heart health.  3. Legumes  - Examples: Beans, lentils, chickpeas, and peas.  - Benefits: Legumes are a great source of protein, fiber, and essential nutrients, and they help maintain healthy cholesterol levels.  4. Nuts and Seeds  - Examples: Almonds, walnuts, chia seeds, flaxseeds, and sunflower seeds.  - Benefits: Nuts and seeds provide healthy fats, fiber, and protein, which can help reduce the risk of heart disease.  5. Low-Glycemic Index Foods  - Examples: Lentils, chickpeas, non-starchy vegetables, and some fruits like apples and pears.  - Benefits: These foods cause a slower and lower rise in blood sugar levels, reducing the risk of insulin resistance and heart disease.  6. Minimally Processed Foods  - Examples: Fresh fruits, vegetables, whole grains, and legumes rather than packaged and highly processed foods.  - Benefits: Minimally processed foods tend to be lower in added sugars, sodium, and unhealthy fats, all of which are beneficial for heart health.  Foods to Limit or Avoid:  1. Refined Grains  - Examples: White bread, white rice, pastries, and sugary cereals.  - Why to Limit: These foods have been stripped of their fiber and nutrients, leading to spikes in blood sugar levels and increased risk of heart disease.  2. Sugary Foods and Beverages  - Examples: Soft drinks, candy, cakes, cookies, and other sweets.  - Why to Limit: High sugar intake is linked to weight gain, high blood pressure, and increased risk of heart disease.  3. High-Glycemic Index Foods  - Examples: White bread, white rice, and other foods that cause rapid spikes in blood sugar.  - Why to Limit: These can lead to insulin resistance and higher risk of cardiovascular disease.  4. Trans Fats and Saturated Fats  - Examples: Fast food, fried foods, and commercially baked goods.  - Why to Limit: These fats can raise LDL (bad) cholesterol levels and lower HDL (good) cholesterol levels, increasing the risk of heart disease.  Tips for a Heart-Healthy Diet:  - Increase Fiber Intake: Aim for at least 25-30 grams of fiber per day from a variety of plant-based foods.  - Choose Healthy Fats: Include sources of healthy fats like olive oil, avocado, and fatty fish in your diet.  - Watch Portion Sizes: Even healthy foods can contribute to weight gain if eaten in large quantities.  - Stay Hydrated: Drink plenty of water and limit sugary beverages.  Incorporating these types of carbohydrates and adhering to these guidelines can help you maintain a heart-healthy diet and reduce the risk of cardiovascular diseases. |
| **GPT4o Answer 3** | In a heart-healthy diet, it's important to focus on the quality of carbohydrates rather than just the quantity. Here are the types of carbohydrates you should consider:  1. Whole Grains  Whole grains are rich in fiber, vitamins, and minerals. They help in maintaining healthy cholesterol levels and reducing the risk of heart disease.  - Examples: Oats, brown rice, whole wheat, barley, quinoa, bulgur, and whole grain bread and pasta.  2. Fruits and Vegetables  Fruits and vegetables are high in vitamins, minerals, antioxidants, and dietary fiber. They can help reduce blood pressure, improve heart health, and lower the risk of cardiovascular diseases.  - Examples: Berries, apples, oranges, bananas, leafy greens, broccoli, carrots, and sweet potatoes.  3. Legumes  Legumes are a great source of complex carbohydrates, fiber, and plant-based protein. They help in maintaining healthy blood sugar levels and can lower cholesterol.  - Examples: Beans (black beans, kidney beans, chickpeas), lentils, peas.  4. Nuts and Seeds  While not primarily carbohydrate sources, nuts and seeds do contain beneficial carbs along with healthy fats and protein. They can improve lipid profiles and provide heart-protective benefits.  - Examples: Almonds, walnuts, flaxseeds, chia seeds, pumpkin seeds.  5. Fiber-Rich Foods  Dietary fiber is crucial for heart health as it can help lower cholesterol levels and regulate blood sugar.  - Examples: Bran, flaxseed, chia seeds, legumes, fruits, and vegetables.  Types of Carbohydrates to Limit or Avoid:  1. Refined Grains and Sugars  - These include white bread, pastries, cookies, and other baked goods made with refined flour.  - Avoid sugary beverages, candy, and high-sugar snacks.  2. Processed Foods  - Highly processed foods often contain added sugars, unhealthy fats, and low-quality carbohydrates that can contribute to heart disease.  - Examples include packaged snacks, fast foods, and ready-to-eat meals.  3. Sweetened Beverages  - Sodas, fruit punches, and other sugar-sweetened drinks can significantly increase the risk of heart disease.  By focusing on these types of carbohydrates and minimizing the intake of refined and processed carbs, you can improve your overall heart health and reduce the risk of cardiovascular diseases. |
| **Llama 3 Answer 1** | In a heart-healthy diet, it's essential to focus on whole, unprocessed carbohydrates that are rich in fiber, vitamins, and minerals. Here are the types of carbohydrates that can help support heart health:  1. Whole grains: Choose whole, unrefined grains like:  * Brown rice  * Quinoa  * Whole wheat bread  * Whole grain pasta  * Oats  * Barley  2. Fiber-rich fruits: Enjoy a variety of fruits, including:  * Berries (e.g., blueberries, strawberries, raspberries)  * Citrus fruits (e.g., oranges, grapefruits, lemons)  * Apples  * Bananas  * Avocados (yes, they're a fruit too!)  3. Leafy green vegetables: These are rich in fiber, potassium, and antioxidants:  * Spinach  * Kale  * Collard greens  * Broccoli  * Carrots  4. Legumes: Legumes are a great source of fiber, protein, and potassium:  * Lentils  * Chickpeas  * Black beans  * Kidney beans  * Soybeans  5. Starchy vegetables: These are rich in complex carbohydrates and fiber:  * Sweet potatoes  * Corn  * Peas  * Winter squash (e.g., acorn squash, butternut squash)  6. Low-fat dairy: Choose low-fat or fat-free dairy products:  * Milk  * Yogurt  * Cheese (in moderation)  Remember to limit or avoid:  * Refined grains (e.g., white bread, sugary cereals)  * Added sugars (e.g., table sugar, honey, syrups)  * Saturated and trans fats (e.g., butter, lard, partially hydrogenated oils)  * Processed meats (e.g., hot dogs, sausages, bacon)  Aim to include a variety of these heart-healthy carbohydrates in your diet to help:  * Lower cholesterol levels  * Regulate blood sugar levels  * Maintain a healthy blood pressure  * Support a healthy weight  * Reduce the risk of heart disease and stroke  Always consult with a healthcare professional or a registered dietitian to create a personalized diet plan that meets your specific needs and health goals. |
| **Llama 3 Answer 2** | In a heart-healthy diet, it's essential to focus on whole, unprocessed carbohydrate sources that are rich in fiber, vitamins, and minerals. Here are the best types of carbohydrates to include:  1. Whole grains: Choose whole grains over refined or processed grains. Whole grains contain more fiber, vitamins, and minerals, which can help lower cholesterol and blood pressure. Examples:  * Brown rice  * Quinoa  * Whole wheat bread  * Whole grain pasta  * Oats  * Barley  2. Fruits: Fruits are rich in fiber, antioxidants, and potassium, which can help lower blood pressure and cholesterol. Aim for a variety of colors to get a range of nutrients. Examples:  * Berries (e.g., blueberries, strawberries, raspberries)  * Citrus fruits (e.g., oranges, grapefruits, lemons)  * Apples  * Bananas  * Avocados (yes, they're a fruit too!)  3. Vegetables: Like fruits, vegetables are rich in fiber, vitamins, and minerals. Aim for a variety of colors to get a range of nutrients. Examples:  * Leafy greens (e.g., spinach, kale, collard greens)  * Cruciferous vegetables (e.g., broccoli, cauliflower, Brussels sprouts)  * Carrots  * Sweet potatoes  * Tomatoes  4. Legumes: Legumes are a great source of protein, fiber, and potassium. They can help lower cholesterol and blood pressure. Examples:  * Lentils  * Chickpeas  * Black beans  * Kidney beans  * Soybeans  5. Low-fat dairy: Choose low-fat or fat-free dairy products to get calcium and protein without excessive saturated fat. Examples:  * Low-fat milk  * Fat-free yogurt  * Low-fat cheese  Remember to limit or avoid the following carbohydrate sources, which can be detrimental to heart health:  * Refined grains (e.g., white bread, sugary cereals)  * Processed snacks (e.g., chips, crackers, cookies)  * Sugary drinks (e.g., soda, sports drinks, sweet tea)  * Foods high in added sugars (e.g., baked goods, candy, ice cream)  Aim to include a variety of whole, unprocessed carbohydrate sources in your diet to support heart health. The American Heart Association recommends that adults consume 45-65% of their daily calories from carbohydrates. |
| **Llama 3 Answer 3** | In a heart-healthy diet, it's essential to focus on whole, unprocessed carbohydrate sources that are rich in fiber, vitamins, and minerals. Here are the best types of carbohydrates to include:  1. Fruits: Aim for a variety of colorful fruits, such as berries, citrus fruits, apples, and bananas. Fresh, frozen, or dried fruits are all good options.  2. Vegetables: Include a wide range of vegetables, like leafy greens, broccoli, bell peppers, carrots, and sweet potatoes. Choose fresh, frozen, or canned options.  3. Whole grains: Whole grains, such as:  * Brown rice  * Quinoa  * Whole wheat bread  * Whole grain pasta  * Oats  * Barley  * Rye  4. Legumes: Legumes are a great source of protein, fiber, and minerals. Include:  * Lentils  * Chickpeas  * Black beans  * Kidney beans  * Soybeans  * Peas  5. Dairy: Choose low-fat or fat-free dairy products, such as:  * Milk  * Yogurt  * Cheese (in moderation)  6. Starchy vegetables: These are rich in complex carbohydrates and fiber:  * Sweet potatoes  * Corn  * Peas  * Winter squash  7. Ancient grains: These grains are rich in fiber, protein, and minerals:  * Kamut  * Teff  * Amaranth  * Buckwheat  When choosing carbohydrates, remember to:  * Opt for whole, unprocessed foods whenever possible.  * Limit or avoid refined, processed, and packaged carbohydrates, such as:  + White bread  + Sugary snacks  + Refined grains  + Fruit juices  + Sugary drinks  * Watch portion sizes and control the amount of carbohydrates you consume to maintain a healthy weight and blood sugar levels.  Aim to include a variety of these carbohydrate sources in your diet to ensure you're getting a balanced intake of essential nutrients. |
| **LLaMA3+RAG model Answer 1** | To follow a heart-healthy diet, it is recommended to focus on consuming complex carbohydrates, which are rich in nutrients and fiber. These include:  1. Fruits: Fresh, frozen, or dried fruits are excellent sources of complex carbohydrates, fiber, and essential vitamins and minerals.  2. Vegetables: A variety of colorful vegetables, including leafy greens, bell peppers, and cruciferous vegetables, provide complex carbohydrates, fiber, and antioxidants.  3. Legumes: Beans, lentils, and peas are rich in complex carbohydrates, protein, and fiber, making them an excellent addition to a heart-healthy diet.  4. Whole grains: Whole-grain bread, brown rice, quinoa, and whole-grain pasta provide complex carbohydrates, fiber, and B vitamins.  5. Dried peas: Dried peas, such as chickpeas and black beans, are a good source of complex carbohydrates, protein, and fiber.  When choosing carbohydrates, it is essential to limit or avoid refined sugars and highly processed foods, which are often high in empty calories and low in nutrients. Instead, focus on whole, unprocessed foods that provide a rich source of complex carbohydrates, fiber, and essential nutrients.  Sources:  American Heart Association. (2024). Carbohydrates. Retrieved from <https://www.heart.org/en/healthy-living/healthy-eating/eat-smart/nutrition-basics/carbohydrates>  American Heart Association. (2023). Confused about carbs? This might help. Retrieved from <https://www.heart.org/en/news/2023/08/11/confused-about-carbs-this-might-help>  Odegaard, A. O., & Gardner, C. D. (2023). Carbohydrates and heart health: A review of the evidence. Journal of the American Heart Association, 12(10), e027345. doi: 10.1161/JAHA.122.027345 |
| **LLaMA3+RAG model Answer 2** | In a heart-healthy diet, it is recommended to focus on consuming complex carbohydrates, which are rich in nutrients and fiber. These include:  1. Fruits and vegetables: These are loaded with healthy nutrients, fiber, and antioxidants. Examples include bananas, apples, broccoli, and carrots.  2. Legumes: Legumes such as beans, lentils, and dried peas are rich in protein, fiber, and various vitamins and minerals.  3. Whole grains: Whole grains like brown rice, whole-grain pasta, bread, and cereals provide fiber, vitamins, and minerals. Examples include oatmeal, quinoa, and whole-grain bread.  4. Fiber-rich foods: Foods high in fiber like peas, beans, and whole grains can help lower cholesterol levels and improve digestive health.  It is also important to limit or avoid simple carbohydrates, which are low in nutrients and high in empty calories. These include:  1. Refined sugars: Foods with added sugars like candy, cakes, and sugary drinks provide empty calories and can increase the risk of heart disease.  2. Processed foods: Highly processed foods like white bread, sugary snacks, and sweetened beverages are often low in nutrients and high in unhealthy ingredients.  When choosing carbohydrates, consider the quality and source of the food rather than the quantity. Focus on whole, unprocessed foods and limit or avoid highly processed and sugary foods.  Sources:  American Heart Association. (2024). Carbohydrates. Retrieved from <https://www.heart.org/en/healthy-living/healthy-eating/eat-smart/nutrition-basics/carbohydrates>  American Heart Association. (2023). Confused about carbs? This might help. Retrieved from <https://www.heart.org/en/news/2023/08/11/confused-about-carbs-this-might-help> |
| **LLaMA3+RAG model Answer 3** | In a heart-healthy diet, it is recommended to focus on consuming complex carbohydrates, which are rich in nutrients and fiber. These include:  1. Fruits and vegetables: These are loaded with healthy nutrients, vitamins, and minerals, and are essential to a heart-healthy diet.  2. Legumes, beans, lentils, and dried peas: These are rich in complex carbohydrates, protein, and fiber, making them an excellent choice for heart health.  3. Whole grains: Enjoy brown rice, whole-grain pasta, breads, and cereals, which are rich in fiber, vitamins, and minerals.  It is also important to limit refined sugars and choose complex carbohydrates over simple ones. Simple carbohydrates, such as those found in highly processed foods, can cause a rapid spike in blood glucose levels, which can increase the risk of heart disease.  Additionally, consider the processing of carbohydrates, as it can affect their nutritional value. Whole, unprocessed foods like fruits, vegetables, and whole grains are generally healthier than processed or refined options.  In summary, a heart-healthy diet should emphasize complex carbohydrates from whole, unprocessed foods, such as fruits, vegetables, legumes, and whole grains, while limiting refined sugars and processed carbohydrates.  Sources:  American Heart Association. (2024). Carbohydrates. Retrieved from <https://www.heart.org/en/healthy-living/healthy-eating/eat-smart/nutrition-basics/carbohydrates>  American Heart Association. (2023, August 11). Confused about carbs? This might help. Retrieved from <https://www.heart.org/en/news/2023/08/11/confused-about-carbs-this-might-help> |
| **Question 8** | **How many eggs can I eat on a heart healthy diet?** |
| **Perplexity Answer 1** | The recommended number of eggs to consume on a heart-healthy diet varies based on individual risk factors and overall dietary patterns. Here are some key points:  For most healthy individuals without heart disease or diabetes:  - Eating up to one egg per day is generally considered safe and may even have some benefits[1][2]. The American Heart Association suggests one egg (or two egg whites) per day as part of a healthy diet[1].  For those with diabetes, heart disease, or high cholesterol:  - It's advisable to limit egg consumption to no more than 3-4 egg yolks per week[2][3]. The cholesterol and saturated fat in egg yolks can exacerbate these conditions.  - Egg whites can be consumed more liberally as they provide protein without cholesterol[1].  Regardless of egg intake, it's crucial to consider the overall diet:  - Avoid eating eggs with sources of saturated fat like bacon, sausage, butter, or fried foods as this increases heart disease risk[1][4].  - Opt for healthier cooking methods like boiling, poaching, or using non-tropical vegetable oils instead of frying[1][4].  - Pair eggs with nutrient-dense foods like vegetables, fruits, and whole grains for a more balanced meal[3].  Additionally, a recent large study found that eating 3-4 eggs per week was associated with a slightly higher risk (6-8%) of cardiovascular disease and death[5]. However, this study had limitations and the evidence on moderate egg consumption remains mixed.  In summary, for most people, up to one egg per day can be included as part of an overall heart-healthy dietary pattern focused on vegetables, fruits, whole grains, lean proteins, and healthy fats. Those with existing conditions may want to limit egg yolks while being mindful of preparation methods and overall diet quality.  Citations:  [1] https://www.heart.org/en/news/2018/08/15/are-eggs-good-for-you-or-not  [2] https://www.health.harvard.edu/heart-health/are-eggs-risky-for-heart-health  [3] https://www.hsph.harvard.edu/nutritionsource/food-features/eggs/  [4] https://health.clevelandclinic.org/how-many-eggs-can-you-eat-on-a-heart-healthy-diet  [5] https://www.feinberg.northwestern.edu/research/podcast/eggs-heart-health.html |
| **Perplexity Answer 2** | For most healthy individuals, eating up to one egg per day is not associated with an increased risk of heart disease or stroke[1][2]. However, for people with diabetes, heart disease, or difficulty controlling their cholesterol levels, it may be best to limit egg consumption to no more than 3 egg yolks per week[3][4].  The key factors to consider are:  - Dietary cholesterol from eggs has a smaller effect on blood cholesterol levels compared to saturated and trans fats[2][3]  - Eggs are a rich source of protein, vitamins, and antioxidants that can be part of a healthy diet[1][3]  - The way eggs are prepared (e.g. frying in oil or butter) and what they are served with (e.g. bacon, cheese, refined carbs) also impacts their healthiness[1][4]  - For those with diabetes and heart disease, limiting egg yolks to 3 per week is recommended[3][4]  So in summary, for most healthy people, eating up to an egg per day can be part of a heart-healthy diet, but those with certain risk factors should limit intake to 3 egg yolks per week and focus on preparing eggs in a healthy way. The overall dietary pattern is most important for heart health[1][2][3][4].  Citations:  [1] https://www.heart.org/en/news/2018/08/15/are-eggs-good-for-you-or-not  [2] https://www.health.harvard.edu/heart-health/are-eggs-risky-for-heart-health  [3] https://www.hsph.harvard.edu/nutritionsource/food-features/eggs/  [4] https://health.clevelandclinic.org/how-many-eggs-can-you-eat-on-a-heart-healthy-diet  [5] https://www.feinberg.northwestern.edu/research/podcast/eggs-heart-health.html |
| **Perplexity Answer 3** | For most healthy people, eating up to one egg per day is not associated with increased risk of heart disease or stroke[1][2]. However, for those with diabetes or heart disease, it may be best to limit egg consumption to no more than 3 yolks per week[3].  The key points are:  - Eggs are a rich source of protein, vitamins, and nutrients like lutein and zeaxanthin that are good for eye health[1][2].  - Most of the cholesterol in the body is produced by the liver and is influenced more by saturated and trans fats in the diet, not dietary cholesterol from eggs[2].  - Large studies have not found higher rates of heart disease in people who eat up to one egg per day as part of a healthy diet[1][2].  - For those with diabetes or heart disease, limiting egg yolks to 3 per week may be prudent[3].  - The foods you eat with eggs also matter - eggs are a better choice than sugary cereals or refined carbs, but not as heart-healthy as oatmeal with fruit and nuts[3].  So in summary, for most people, an egg a day can be part of a heart-healthy diet, but those with diabetes or heart disease may want to limit to 3 yolks per week. The overall quality of your diet is most important for heart health[1][2][3].  Citations:  [1] https://www.heart.org/en/news/2018/08/15/are-eggs-good-for-you-or-not  [2] https://www.health.harvard.edu/heart-health/are-eggs-risky-for-heart-health  [3] https://www.hsph.harvard.edu/nutritionsource/food-features/eggs/  [4] https://health.clevelandclinic.org/how-many-eggs-can-you-eat-on-a-heart-healthy-diet  [5] https://www.feinberg.northwestern.edu/research/podcast/eggs-heart-health.html |
| **GPT4o Answer 1** | When following a heart-healthy diet, the number of eggs you can eat can depend on your overall health, cholesterol levels, and dietary needs. Here are some general guidelines:  1. Moderation is Key: For most people, consuming up to 7 eggs per week is considered safe and can be part of a heart-healthy diet. This includes eggs used in cooking and baking, not just whole eggs eaten on their own.  2. Watch Cholesterol Levels: Eggs are high in dietary cholesterol, but for most people, dietary cholesterol does not have as significant an impact on blood cholesterol levels as once thought. However, if you have high cholesterol or other risk factors for heart disease, you should be more cautious.  3. Focus on the Whole Diet: A heart-healthy diet is more about the overall pattern rather than specific foods. Emphasize fruits, vegetables, whole grains, lean proteins, and healthy fats while limiting saturated fats, trans fats, sodium, and added sugars.  4. Consult Your Doctor: Individual recommendations can vary. If you have heart disease, diabetes, or other health conditions, it's best to consult your doctor or a dietitian for personalized advice.  5. Egg Whites: If you're concerned about cholesterol but still want to include eggs in your diet, consider using egg whites or cholesterol-free egg substitutes. Most of the cholesterol in eggs is found in the yolk, so egg whites can be a good alternative.  Overall, including eggs in your diet in moderation can be part of a heart-healthy eating plan, especially when balanced with other nutrient-rich foods. |
| **GPT4o Answer 2** | When following a heart-healthy diet, the number of eggs you can eat often depends on individual health factors and dietary guidelines. Here are some general recommendations based on current research and guidelines:  1. General Population: For most healthy individuals, consuming up to seven eggs per week does not seem to increase the risk of heart disease. This includes eggs consumed in various forms, such as boiled, scrambled, or in baked goods.  2. Individuals with Heart Disease or Diabetes: If you have heart disease or diabetes, it might be advisable to limit egg consumption due to the potential risk of raising cholesterol levels. Some experts suggest limiting intake to 3-4 eggs per week.  3. Egg Whites: Egg whites are a good alternative as they contain protein without the cholesterol found in yolks. You can consume egg whites more liberally as part of a heart-healthy diet.  4. Balancing with Other Foods: It’s important to consider the overall balance of your diet. Including plenty of fruits, vegetables, whole grains, lean proteins, and healthy fats is crucial. If you eat eggs regularly, balance your intake with other low-cholesterol foods.  5. Consulting with a Healthcare Provider: Because individual health needs vary, it’s best to consult with a healthcare provider or a registered dietitian. They can provide personalized recommendations based on your specific health profile and dietary needs.  By considering these factors and maintaining a balanced diet, eggs can be part of a heart-healthy diet in moderation. |
| **GPT4o Answer 3** | When it comes to heart health, the consumption of eggs has been a topic of extensive research and discussion. Eggs are a nutritious food, providing high-quality protein, vitamins, and minerals. However, they also contain cholesterol, which has historically raised concerns about their impact on heart health.  Here's a balanced view based on current research and dietary guidelines:  1. Moderation is Key: For most people, consuming up to one egg per day is generally considered safe and does not appear to increase the risk of heart disease. This is supported by studies that have shown no significant association between moderate egg consumption and cardiovascular disease in the general population.  2. Consider Your Overall Diet: The impact of eggs on heart health depends on your overall diet. If your diet is high in saturated and trans fats, the cholesterol in eggs might have a more significant impact on your blood cholesterol levels. A diet rich in fruits, vegetables, whole grains, and healthy fats (such as those from nuts, seeds, and fish) can mitigate some of the potential risks associated with dietary cholesterol.  3. Individual Differences Matter: Some people are more sensitive to dietary cholesterol than others. If you have certain conditions, such as diabetes or a family history of heart disease, you might need to be more cautious with egg consumption. Consulting with a healthcare provider can help determine what’s best for your individual health needs.  4. Balance with Other Protein Sources: Incorporate a variety of protein sources in your diet, such as lean meats, fish, legumes, and plant-based proteins, to ensure a balanced intake of nutrients and avoid excessive consumption of any single food.  5. Current Guidelines: As of the latest dietary guidelines, it’s generally recommended that healthy individuals can consume up to seven eggs per week without increasing the risk of heart disease. This can include one egg per day or spreading out the consumption throughout the week.  In summary, for most people, eating up to one egg per day can be part of a heart-healthy diet, especially when balanced with other nutrient-dense foods and a healthy lifestyle. Always consider personal health factors and dietary needs, and consult with a healthcare provider if you have specific concerns or conditions that might require a different approach. |
| **Llama Answer 1** | Eggs have gotten a bad rap in the past, but they're actually a nutritious food that can be part of a heart-healthy diet. The key is to consume them in moderation.  For a long time, experts recommended limiting egg consumption to 2-3 eggs per week due to their high cholesterol content. However, more recent research has shown that dietary cholesterol has a limited impact on blood cholesterol levels for most people.  The American Heart Association (AHA) recommends that healthy individuals limit their dietary cholesterol intake to 300 milligrams per day. Based on this guideline, eggs can be a part of a heart-healthy diet as long as you're mindful of your overall cholesterol intake.  Here are some egg-related facts to keep in mind:  1. Egg yolks are the main source of cholesterol: One large egg yolk contains about 200 milligrams of cholesterol. Egg whites, on the other hand, are cholesterol-free.  2. Eggs are a good source of protein and nutrients: Eggs are an excellent source of protein, vitamins, and minerals like vitamin D, B12, and iron.  3. The quality of eggs matters: Choose eggs that are rich in omega-3 fatty acids, vitamins, and minerals, as these may have a more positive impact on heart health.  So, how many eggs can you eat on a heart-healthy diet?  * General guideline: If you're healthy, you can safely consume 1-2 eggs per day as part of a balanced diet.  * If you have high cholesterol or heart disease: You may want to limit your egg consumption to 1 egg per week or 4-5 egg whites per week.  * If you're at risk for heart disease: You may want to limit your egg consumption to 1 egg per day or 2-3 eggs per week.  Remember, it's essential to consider your overall diet and lifestyle when it comes to egg consumption. If you're eating a balanced diet rich in fruits, vegetables, whole grains, lean proteins, and healthy fats, eggs can be a nutritious addition.  Just be mindful of your overall cholesterol intake and choose eggs that are rich in nutrients and omega-3 fatty acids. |
| **Llama Answer 2** | Eggs have gotten a bad rap in the past when it comes to heart health, but the good news is that they can be part of a heart-healthy diet in moderation.  The American Heart Association (AHA) recommends limiting dietary cholesterol to 300 milligrams per day. Since a large egg has about 186 milligrams of cholesterol, it's essential to keep track of your overall cholesterol intake from all sources, including eggs, meat, dairy, and processed foods.  Here are some guidelines to consider:  1. Healthy individuals: If you're healthy and don't have high cholesterol, diabetes, or heart! disease, you can safely consume an egg a day without significantly increasing your risk of heart disease.  2. High-risk individuals: If you have high cholesterol, diabetes, heart disease, or are at risk for these conditions, it's recommended to limit your egg consumption to 2-3 eggs per week. This is because eggs are relatively high in cholesterol and saturated fat, which can exacerbate these conditions.  3. Egg whites: If you're concerned about cholesterol, consider eating egg whites, which are cholesterol-free and an excellent source of protein.  4. Cooking methods: How you cook your eggs also matters. Opt for poaching, boiling, or baking instead of frying, which adds extra fat and calories.  5. Overall diet: Remember, eggs are just one part of your overall diet. Focus on a balanced diet rich in fruits, vegetables, whole grains, lean proteins, and healthy fats to support heart health.  In summary, if you're healthy, an egg a day is unlikely to harm your heart health. However, if you're at risk for heart disease or have certain health conditions, it's best to limit your egg consumption to 2-3 eggs per week. Always prioritize a balanced diet and consult with a healthcare professional or registered dietitian for personalized guidance. |
| **Llama Answer 3** | The eternal question about eggs and heart health!  For a long time, eggs were considered a dietary no-no for people with heart health concerns due to their high cholesterol content. However, the latest research and dietary guidelines have changed the way we think about eggs and heart health.  Here's the good news: eggs can be part of a heart-healthy diet in moderation.  The Cholesterol Factor  One large egg contains about 186 milligrams of cholesterol, which is a significant amount considering the daily recommended limit of 300 milligrams. However, dietary cholesterol has a limited impact on blood cholesterol levels for most people. Saturated and trans fats, found in foods like butter, lard, and partially hydrogenated oils, have a much greater impact on blood cholesterol levels and heart health.  The American Heart Association's (AHA) Guidelines  The AHA recommends limiting dietary cholesterol to 300 milligrams per day. Based on this guideline, it's suggested to limit egg consumption to 1-2 eggs per week. However, this recommendation is not a hard and fast rule, and individual circumstances may vary.  The Science Behind Eggs and Heart Health  Recent studies have found that moderate egg consumption, up to 1 egg per day, does not increase the risk of heart disease in healthy individuals. In fact, eggs are a good source of protein, vitamins, and minerals, such as:  1. B vitamins: essential for heart health and energy metabolism  2. Vitamin D: important for bone health and immune function  3. Choline: a nutrient that helps with metabolism and liver function  4. Omega-3 fatty acids: found in egg yolks, these anti-inflammatory fats support heart health  Who Should Limit Egg Consumption?  While moderate egg consumption is generally safe, certain individuals may need to limit or avoid eggs due to their high cholesterol content or other health conditions:  1. People with high cholesterol or heart disease: may need to limit egg consumption to 1-2 eggs per week  2. Diabetics: may need to limit egg consumption due to the risk of cardiovascular disease  3. Those with familial hypercholesterolemia (FH): a genetic condition characterized by very high cholesterol levels  In Summary  In the context of a heart-healthy diet, you can safely consume:  * 1 egg per day (7 eggs per week) if you're a healthy individual  * 1-2 eggs per week if you have high cholesterol or heart disease  * Limit or avoid eggs if you have diabetes or FH  Remember, a heart-healthy diet is about balance and variety. Focus on consuming a wide range of whole, nutrient-dense foods, including fruits, vegetables, whole grains, lean proteins, and healthy fats. Eggs can be a part of this balanced diet, but don't overdo it! |
| **LLaMA3+RAG model Answer 1** | According to the American Heart Association, a heart-healthy diet can include eggs in moderation. The 2019 science advisory suggests that healthy individuals can include up to a whole egg or the equivalent in their diets each day. Additionally, older people with healthy cholesterol levels can have two eggs per day.  It's essential to consider the overall content of the diet and individual risk factors when deciding how many eggs to eat. People at risk for heart disease, who have diabetes, or who have had a heart attack should pay close attention to the amount of cholesterol in their diet.  The American Heart Association specifically recommends one egg (or two egg whites) per day for people who eat them, as part of a healthy diet. This allows for the nutritional benefits of eggs while minimizing the intake of dietary cholesterol.  It's also important to note that eggs can be part of a healthy diet when cooked using heart-healthy methods, such as boiling, poaching, or scrambling, and when paired with other nutrient-dense foods.  Sources:  American Heart Association. (2023). Here's the latest on dietary cholesterol and how it fits in with a healthy diet. Retrieved from <https://www.heart.org/en/news/2023/08/25/heres-the-latest-on-dietary-cholesterol-and-how-it-fits-in-with-a-healthy-diet>  American Heart Association. (2019). Dietary Cholesterol and Heart Disease. Retrieved from <https://www.heart.org/en/healthy-living/healthy-eating/eat-smart/nutrition-basics/dietary-cholesterol-and-heart-disease>  UT Southwestern Medical Center. (2024). Jo Ann Carson, Professor of Clinical Nutrition. Retrieved from <https://profiles.utsw.edu/profile/133163/jo-ann-carson.html>  The American Journal of Clinical Nutrition. (2023). Egg consumption and cardiovascular risk factors in individuals with prediabetes and type 2 diabetes. Retrieved from <https://doi.org/10.1093/ajcn/nqac144> |
| **LLaMA3+RAG model Answer 2** | According to the American Heart Association, a heart-healthy diet can include up to one whole egg or the equivalent per day. For older individuals with healthy cholesterol levels, two eggs per day are considered tolerable, depending on the overall content of the diet.  It's essential to note that the key to incorporating eggs into a heart-healthy diet is to consider the overall cholesterol content of the diet. If the diet is low in cholesterol, eggs may be considered less dangerous. Additionally, individuals at risk for heart disease, who have diabetes, or who have had a heart attack should pay close attention to the amount of cholesterol in their diet.  Egg whites are a great option for those looking to reduce their cholesterol intake, as they provide plenty of protein without the cholesterol of the yolk. Combining egg whites with whole eggs can also be a good way to balance out the cholesterol content.  It's also important to cook eggs fully to avoid the risk of salmonella and to choose heart-healthy oils when frying eggs, such as corn, canola, or olive oil.  In summary, a heart-healthy diet can include:  Up to one whole egg or the equivalent per day for healthy individuals  Up to two eggs per day for older individuals with healthy cholesterol levels  Egg whites as a low-cholesterol alternative  Combining egg whites with whole eggs for a balanced cholesterol content  Cooking eggs fully to avoid the risk of salmonella  Using heart-healthy oils when frying eggs  Sources:  American Heart Association. (2023). Here's the latest on dietary cholesterol and how it fits in with a healthy diet. Retrieved from <https://www.heart.org/en/news/2023/08/25/heres-the-latest-on-dietary-cholesterol-and-how-it-fits-in-with-a-healthy-diet>  American Heart Association. (2024). Healthy Eating Plate. Retrieved from <https://www.heart.org/en/healthy-living/healthy-eating/eat-smart/nutrition-basics/healthy-eating-plate>  Carson, J. A. (Professor of Clinical Nutrition at UT Southwestern Medical Center in Dallas). (2024). Quoted in American Heart Association article. Retrieved from <https://www.heart.org/en/news/2023/08/25/heres-the-latest-on-dietary-cholesterol-and-how-it-fits-in-with-a-healthy-diet>  Van Horn, L. (2024). Quoted in American Heart Association article. Retrieved from <https://www.heart.org/en/news/2023/08/25/heres-the-latest-on-dietary-cholesterol-and-how-it-fits-in-with-a-healthy-diet> |
| **LLaMA3+RAG model Answer 3** | According to the American Heart Association, a heart-healthy diet can include up to one whole egg or the equivalent per day. For older people with healthy cholesterol levels, two eggs per day are considered tolerable, depending on the overall content of the diet.  It's essential to consider the overall dietary pattern and individual risk factors when deciding how many eggs to eat. People at risk for heart disease, who have diabetes, or who have had a heart attack should pay close attention to the amount of cholesterol in their diet. However, if a person's diet contains little other cholesterol, eggs may be considered less dangerous.  Egg whites are a great option for those who want to reduce their cholesterol intake, as they provide plenty of protein without the cholesterol of the yolk. Combining eggs with egg whites or using heart-healthy oils when cooking eggs can also help minimize the negative effects of dietary cholesterol.  In summary, a heart-healthy diet can include:  Up to one whole egg or the equivalent per day for healthy individuals  Up to two eggs per day for older people with healthy cholesterol levels  Egg whites or a combination of eggs and egg whites for those who want to reduce cholesterol intake  Heart-healthy oils such as corn, canola, or olive oil when cooking eggs  Sources:  American Heart Association. (2023). Here's the latest on dietary cholesterol and how it fits in with a healthy diet. Retrieved from <https://www.heart.org/en/news/2023/08/25/heres-the-latest-on-dietary-cholesterol-and-how-it-fits-in-with-a-healthy-diet>  Carson, J. A. (Professor of Clinical Nutrition at UT Southwestern Medical Center in Dallas). As quoted in the American Heart Association article.  Van Horn, L. (As quoted in the American Heart Association article). |
| **Question 9** | **How much red meat can I eat on a heart healthy diet?** |
| **Perplexity Answer 1** | To maintain a heart-healthy diet, it is recommended to limit your intake of red meat and instead focus on eating more plant-based proteins, fish, and lean poultry[1][4]. Here are some key points:  - Eating red meat daily can triple your levels of TMAO, a chemical linked to increased heart disease risk[1]. High saturated fat levels in red meat also contribute to heart disease[1].  - The American Heart Association encourages limiting red meat and instead eating a variety of foods including more vegetables, fruits, whole grains, low-fat dairy, and plant-based proteins like beans and peas[1][4].  - A Mediterranean-style diet that incorporates fresh lean beef in moderation (7-18 ounces of cooked lean red meat per week) can reduce heart disease risk factors like cholesterol and blood pressure[3].  - Lean beef can be part of a heart-healthy diet when consumed in moderation as part of an overall healthy eating pattern[3]. Choosing lean cuts and limiting portions to 3 ounces or less is recommended[4].  - Replacing red meat with plant-based proteins, fish, and skinless poultry is ideal for heart health[4]. A 3-ounce portion of cooked meat, fish or chicken is about the size of a deck of cards[4].  In summary, while lean red meat can be part of a heart-healthy diet in moderation, it is best to limit red meat intake and focus on eating more plant-based proteins, fish, and lean poultry to reduce heart disease risk. Portion control is key when consuming red meat.  Citations:  [1] https://www.nih.gov/news-events/nih-research-matters/eating-red-meat-daily-triples-heart-disease-related-chemical  [2] https://health.clevelandclinic.org/is-red-meat-bad-for-your-heart-or-not  [3] https://www.beefitswhatsfordinner.com/nutrition/beef-and-heart-health  [4] https://www.heart.org/en/healthy-living/healthy-eating/eat-smart/nutrition-basics/meat-poultry-and-fish-picking-healthy-proteins  [5] https://www.heart.org/en/healthy-living/healthy-eating/eat-smart/nutrition-basics/aha-diet-and-lifestyle-recommendations |
| **Perplexity Answer 2** | For a heart-healthy diet, it's best to limit your intake of red meat and processed meats:  - Eating red meat in moderation, as part of an overall healthy diet, is likely fine for most people[1][2]. However, eating too much red meat, especially processed meats, has been linked to an increased risk of heart disease, stroke, and other health issues[1][2].  - The American Heart Association recommends choosing lean meats and skinless poultry and eating them in moderation[5]. A serving of cooked meat is 3 ounces, about the size of a deck of cards[5].  - Replacing red meat with plant-based protein sources like beans, lentils, nuts, seeds, and soy products can be very heart-healthy[3][5]. Eating more fish, especially fatty fish high in omega-3s, is also recommended for heart health[5].  - Following a Mediterranean-style diet that includes lean red meat in moderation (7-18 ounces of cooked lean red meat per week), along with plenty of vegetables, fruits, whole grains, nuts and beans, can help maintain healthy cholesterol levels and reduce heart disease risk factors[4].  So in summary, while lean red meat can be part of a heart-healthy diet in moderation, it's best to focus on plant-based proteins and fish as your primary protein sources. Limit processed meats and choose lean, unprocessed cuts of red meat if you do eat it.  Citations:  [1] https://newsnetwork.mayoclinic.org/discussion/mayo-clinic-minute-does-eating-red-meat-affect-heart-health/  [2] https://health.clevelandclinic.org/is-red-meat-bad-for-your-heart-or-not  [3] https://health.gov/myhealthfinder/health-conditions/heart-health/heart-healthy-foods-shopping-list  [4] https://www.beefitswhatsfordinner.com/nutrition/beef-and-heart-health  [5] https://www.heart.org/en/healthy-living/healthy-eating/eat-smart/nutrition-basics/meat-poultry-and-fish-picking-healthy-proteins |
| **Perplexity Answer 3** | To maintain a heart-healthy diet, it is recommended to limit your intake of red meat and instead focus on eating more plant-based proteins, fish, and lean poultry[1][4].  The American Heart Association advises choosing healthy protein sources, including[4]:  - Eating fish and seafood regularly  - Getting protein mostly from plant sources like beans, peas, lentils, nuts, and soy  - Choosing fat-free and low-fat dairy products  - Selecting lean and unprocessed meats and poultry  If you do eat red meat, aim for lean cuts and limit your portions to about the size of a deck of cards (3 ounces)[4]. Eating red meat in moderation as part of an overall healthy dietary pattern that emphasizes vegetables, fruits, whole grains, nuts, and beans can help maintain normal cholesterol levels[3].  However, regularly eating large amounts of red meat has been linked to an increased risk of heart disease[1][2]. Replacing red meat with plant-based proteins or fish may help reduce this risk[1][2].  In summary, for optimal heart health, it's best to limit red meat intake and focus on eating a variety of healthy, plant-based proteins along with lean poultry, fish, and low-fat dairy. When you do eat red meat, choose lean cuts and keep portions small as part of an overall balanced diet[1][3][4].  Citations:  [1] https://www.nih.gov/news-events/nih-research-matters/eating-red-meat-daily-triples-heart-disease-related-chemical  [2] https://health.clevelandclinic.org/is-red-meat-bad-for-your-heart-or-not  [3] https://www.beefitswhatsfordinner.com/nutrition/beef-and-heart-health  [4] https://www.heart.org/en/healthy-living/healthy-eating/eat-smart/nutrition-basics/meat-poultry-and-fish-picking-healthy-proteins  [5] https://www.heart.org/en/healthy-living/healthy-eating/eat-smart/nutrition-basics/aha-diet-and-lifestyle-recommendations |
| **GPT4o Answer 1** | For a heart-healthy diet, it's generally recommended to limit red meat consumption due to its association with increased risks of heart disease. Here are some guidelines to consider:  1. Portion Control: Limit red meat intake to small portions, such as 3 ounces (about the size of a deck of cards) per serving.  2. Frequency: Aim to consume red meat no more than 1-2 times per week. Some guidelines suggest even less, such as once a month, particularly if you have risk factors for heart disease.  3. Lean Cuts: Choose lean cuts of red meat, such as loin, round, or extra-lean ground beef. Trim visible fat before cooking.  4. Cooking Methods: Use heart-healthy cooking methods, such as grilling, broiling, baking, or steaming, rather than frying or cooking with added fats.  5. Alternative Proteins: Incorporate more plant-based proteins (like beans, lentils, and tofu), poultry, fish, and other lean meats into your diet as alternatives to red meat.  6. Processed Meats: Avoid or minimize consumption of processed meats, such as bacon, sausages, and deli meats, which are often high in saturated fats and sodium.  It's important to balance your diet with plenty of fruits, vegetables, whole grains, and healthy fats (like those from nuts, seeds, and avocados) to support overall cardiovascular health. Consulting with a healthcare provider or a dietitian can provide personalized advice based on your specific health needs and conditions. |
| **GPT4o Answer 2** | For a heart-healthy diet, it is generally recommended to limit the intake of red meat. Here are some guidelines to follow:  1. Moderation is Key: Aim to consume red meat in moderation. The American Heart Association suggests that individuals should limit their intake of red meat to one to two servings per week. A serving size is typically about 3 ounces cooked, which is roughly the size of a deck of cards.  2. Choose Lean Cuts: When you do eat red meat, choose lean cuts such as sirloin, tenderloin, or round cuts. Trim any visible fat before cooking.  3. Balance with Other Protein Sources: Incorporate a variety of protein sources into your diet. Include more plant-based proteins such as beans, lentils, tofu, and nuts. Fish and poultry are also good alternatives to red meat.  4. Avoid Processed Meats: Limit or avoid processed meats like sausages, hot dogs, and bacon, as these are often high in saturated fats and sodium, which can contribute to heart disease.  5. Cooking Methods Matter: Prepare red meat using healthier cooking methods such as grilling, broiling, baking, or steaming. Avoid frying or cooking with heavy oils and butter.  6. Focus on Overall Diet Quality: A heart-healthy diet is not just about limiting red meat but also about overall dietary patterns. Emphasize fruits, vegetables, whole grains, low-fat dairy products, and healthy fats (like those from olive oil, avocados, and nuts).  By following these guidelines, you can include red meat in your diet while still maintaining a focus on heart health. If you have specific health concerns or conditions, it's always a good idea to consult with a healthcare professional or a registered dietitian for personalized advice. |
| **GPT4o Answer 3** | For a heart-healthy diet, it is recommended to limit the consumption of red meat. Here are some guidelines to help you manage red meat intake while promoting heart health:  1. Limit Portion Sizes: Aim to keep portions of red meat small. A serving size of red meat is typically about 3-4 ounces (85-113 grams), roughly the size of a deck of cards.  2. Frequency: Limit red meat consumption to no more than a few times a month. Some guidelines suggest that red meat should be eaten no more than once or twice a week, while others recommend even less frequent consumption.  3. Choose Lean Cuts: When you do eat red meat, opt for lean cuts such as loin, round, or sirloin. Trim visible fat before cooking.  4. Cooking Methods: Prepare red meat using healthier cooking methods like grilling, baking, broiling, or stewing rather than frying or cooking in butter.  5. Balance with Other Protein Sources: Incorporate a variety of protein sources in your diet, including poultry, fish, beans, lentils, tofu, and nuts. Fish, particularly fatty fish like salmon and mackerel, are excellent sources of omega-3 fatty acids, which are beneficial for heart health.  6. Processed Meats: Avoid or significantly limit processed meats like bacon, sausage, hot dogs, and deli meats, as they are high in sodium and unhealthy fats.  7. Dietary Patterns: Consider adopting dietary patterns known for heart health, such as the Mediterranean diet or the DASH (Dietary Approaches to Stop Hypertension) diet, which emphasize fruits, vegetables, whole grains, and lean proteins, including limited red meat.  8. Monitor Saturated Fat Intake: Be mindful of your overall saturated fat intake, which includes fats from red meat, full-fat dairy products, and certain oils. The American Heart Association recommends that saturated fats should constitute no more than 5-6% of your total daily calories.  Consult with a healthcare provider or a registered dietitian for personalized advice, especially if you have specific health conditions or dietary needs. |
| **Llama 3 Answer 1** | The age-old question about red meat and heart health!  The American Heart Association (AHA) and other health organizations recommend limiting red meat consumption as part of a heart-healthy diet. Here's why:  Red meat and heart disease:  1. Saturated fat and cholesterol: Red meat can be high in saturated fat, which raises blood cholesterol levels and increases the risk of heart disease.  2. Advanced glycation end (AGE) products: Red meat contains AGEs, which are substances that form when protein or fat is cooked at high temperatures. AGEs can accumulate in the body and contribute to oxidative stress, inflammation, and cardiovascular disease.  3. Heme iron: Red meat is a rich source of heme iron, which can increase the risk of heart disease by promoting the formation of free radicals and oxidative stress.  4. Trimethylamine N-oxide (TMAO): Red meat contains L-carnitine, which is converted into TMAO in the gut. High levels of TMAO have been linked to an increased risk of heart disease and stroke.  Recommended intake:  The AHA suggests limiting lean red meat consumption to 1-2 servings per week (1 serving = 3 ounces or about the size of a deck of cards). This is because even lean red meat can have negative effects on heart health when consumed excessively.  Choose leaner cuts and cooking methods:  If you do choose to eat red meat, opt for leaner cuts and cooking methods that reduce the formation of AGEs:  * Choose grass-fed, lean cuts like sirloin, tenderloin, or round.  * Cook using lower-heat methods like grilling, roasting, or stewing instead of frying or broiling.  * Trim visible fat and remove skin to reduce saturated fat intake.  Alternatives to red meat:  Incorporate more plant-based protein sources and heart-healthy alternatives into your diet:  * Poultry (chicken, turkey, duck)  * Fish (fatty fish like salmon, tuna, and mackerel are rich in omega-3s)  * Legumes (lentils, chickpeas, beans)  * Nuts and seeds (almonds, walnuts, chia seeds, flaxseeds)  * Whole grains (quinoa, brown rice, whole wheat)  * Vegetarian and vegan options (tofu, tempeh, seitan)  Remember:  A heart-healthy diet is about balance and variety. Focus on whole, nutrient-dense foods, and limit your intake of processed and packaged products. Consult with a healthcare professional or registered dietitian to develop a personalized eating plan that suits your needs and promotes overall heart health. |
| **Llama 3 Answer 2** | The age-old question about red meat and heart health!  The American Heart Association (AHA) recommends limiting lean meat consumption, including red meat, to 1-2 servings (about 3-4 ounces or 85-115g) per day. However, it's not just about the quantity, but also the quality of the meat.  Here are some guidelines to help you make heart-healthy choices:  1. Choose leaner cuts: Opt for leaner cuts of red meat, such as sirloin, tenderloin, or round. These cuts tend to be lower in saturated fat and cholesterol.  2. Limit processed meat: Processed meats like sausages, hot dogs, and bacon are high in sodium, preservatives, and saturated fats, which can increase heart disease risk. Try to limit or avoid them altogether.  3. Opt for grass-fed or pasture-raised: Grass-fed or pasture-raised beef tends to be higher in omega-3 fatty acids and conjugated linoleic acid (CLA), which may have anti-inflammatory effects.  4. Consider alternative protein sources: Variety is key. Incorporate other protein sources like poultry, fish, beans, lentils, and nuts into your diet to reduce your overall red meat consumption.  5. Cooking methods matter: Avoid frying or charbroiling, which can increase the formation of advanced glycation end products (AGEs) and polycyclic aromatic hydrocarbons (PAHs), both of which may harm heart health. Instead, opt for grilling, roasting, or stewing.  6. Watch portion sizes: Even leaner cuts of red meat can be high in calories and fat if consumed in excess. Control your portion sizes to maintain a healthy weight and reduce your risk of heart disease.  7. Balance your diet: A heart-healthy diet is not just about red meat; it's about balance. Focus on whole, unprocessed foods like fruits, vegetables, whole grains, and healthy fats, like avocado and olive oil.  Remember, individual needs may vary based on factors like age, health status, and lifestyle. If you have specific concerns or questions, consult with a healthcare professional or a registered dietitian for personalized guidance. |
| **Llama 3 Answer 3** | The age-old question about red meat and heart health!  The American Heart Association (AHA) recommends limiting red meat consumption as part of a heart-healthy diet. Here's why:  1. Saturated fat and cholesterol: Red meat can be high in saturated fat, which can raise your low-density lipoprotein (LDL) cholesterol, also known as "bad" cholesterol. High levels of LDL cholesterol can increase your risk of heart disease.  2. Trimethylamine N-oxide (TMAO): Red meat contains a compound called L-carnitine, which can be converted into TMAO in the gut. Elevated TMAO levels have been linked to an increased risk of heart disease.  So, how much red meat is too much? Here are some guidelines:  Recommended daily intake:  * The AHA suggests limiting red meat to 1-2 servings per week, with a serving size of about 3 ounces (85g) cooked.  * The American Cancer Society recommends limiting red and processed meat to 18 ounces (510g) per week.  What counts as a serving?  * 3 ounces (85g) cooked red meat, such as beef, pork, lamb, or game meats  * 1/4 cup (30g) cooked ground meat  Tips for heart-healthy red meat choices:  1. Choose leaner cuts: Opt for cuts with less marbling (fat) like sirloin, tenderloin, or round.  2. Go for grass-fed: Grass-fed beef may be higher in omega-3 fatty acids and conjugated linoleic acid (CLA), which may have anti-inflammatory effects.  3. Limit processed meats: Processed meats like hot dogs, sausages, and bacon are high in sodium, preservatives, and saturated fat.  4. Cook wisely: Grill, broil, or bake red meat instead of frying to reduce saturated fat intake.  5. Balance with plant-based protein: Include more plant-based protein sources like beans, lentils, tofu, and tempeh in your diet.  Remember, a heart-healthy diet is about balance and variety. Focus on whole, unprocessed foods, including fruits, vegetables, whole grains, lean protein sources, and healthy fats. |
| **LLaMA3+RAG model Answer 1** | To achieve a heart-healthy diet, it's essential to limit your red meat consumption. The American Heart Association (AHA) recommends choosing lean protein sources, such as plant-based options, fish, seafood, lean poultry, and low-fat or fat-free dairy products.  While there is no specific daily or weekly limit set by the AHA for red meat consumption, it's essential to keep in mind that a heart-healthy diet should emphasize whole, unprocessed foods and limit processed and saturated fat-rich foods.  The DASH eating plan, which scored a perfect 100 in the AHA's diet evaluation, suggests limiting lean meats, including red meat, to 3-4 servings per week, with a serving size of about 3 ounces or the size of a deck of cards. This translates to approximately 9-12 ounces of red meat per week.  It's also important to choose leaner cuts of red meat, such as sirloin, tenderloin, or round, and opt for grass-fed or pasture-raised options when possible. Additionally, consider alternative protein sources, like plant-based options, fish, and poultry, to reduce your overall red meat consumption.  In summary, for a heart-healthy diet, aim to limit your red meat consumption to 3-4 servings per week, with a serving size of about 3 ounces, and choose leaner cuts and alternative protein sources whenever possible.  Sources:  American Heart Association. (2023). Here's How 10 Popular Diets Scored for Heart Health. Retrieved from <https://www.heart.org/en/news/2023/04/27/heres-how-10-popular-diets-scored-for-heart-health>  American Heart Association. (2023). 2023 Dietary Guidance to Improve Cardiovascular Health. Retrieved from <https://www.ahajournals.org/doi/full/10.1161/CIR.0000000000001146>  Academy of Nutrition and Dietetics. (2020). EatRight: Red Meat and Heart Health. Retrieved from <https://www.eatright.org/food/planning-and-prep/snack-and-meal-tips/red-meat-and-heart-health>  American Heart Association. (2017). Healthy Eating Plate. Retrieved from <https://www.heart.org/en/healthy-living/healthy-eating/eat-smart/nutrition-basics/healthy-eating-plate> |
| **LLaMA3+RAG model Answer 2** | Based on the provided context, it's not explicitly stated how much red meat one should eat for a heart-healthy diet. However, we can infer some guidelines from the descriptions of the diets and their scores.  The DASH eating plan, which scored a perfect 100, is described as being high in non-starchy vegetables, fruits, whole grains, and legumes, with proteins mostly coming from plant sources, such as legumes, beans, or nuts, along with fish or seafood, lean poultry, and low-fat or fat-free dairy products. This suggests that a heart-healthy diet should emphasize plant-based protein sources and include lean animal protein sources in moderation.  The American Heart Association's dietary guidance, which is mentioned in the context, recommends limiting lean meat consumption to 0-1 servings per day, with a serving size being about 2-3 ounces or the size of a deck of cards (AHA, 2020). It's also recommended to choose lean meats, such as poultry, fish, and plant-based alternatives, and to limit processed meats.  In terms of red meat specifically, the AHA recommends limiting or avoiding processed meats, such as bacon, sausage, and deli meats, due to their high sodium and saturated fat content. For unprocessed red meats, such as beef, pork, and lamb, it's recommended to choose lean cuts and limit portion sizes to 3 ounces or less per serving (AHA, 2020).  In summary, while there is no specific daily limit mentioned for red meat in the provided context, a heart-healthy diet should emphasize plant-based protein sources and include lean animal protein sources, such as poultry, fish, and low-fat dairy products, in moderation. It's recommended to limit or avoid processed meats and choose lean cuts of unprocessed red meats, limiting portion sizes to 3 ounces or less per serving.  Sources:  American Heart Association. (2020). Healthy Eating Plate. Retrieved from <https://www.heart.org/en/healthy-living/healthy-eating/eat-smart/nutrition-basics/healthy-eating-plate>  American Heart Association. (2023). Here's How 10 Popular Diets Scored for Heart Health. Retrieved from <https://www.heart.org/en/news/2023/04/27/heres-how-10-popular-diets-scored-for-heart-health>  Lichtenstein, A. H., et al. (2020). Dietary Guidance to Improve Cardiovascular Health: A Scientific Statement From the American Heart Association. Circulation, 141(11), e934–e945. doi: 10.1161/CIR.0000000000001146 |
| **LLaMA3+RAG model Answer 3** | To maintain a heart-healthy diet, it is recommended to limit the consumption of red meat. The American Heart Association (AHA) suggests that proteins should mostly come from plant sources, such as legumes, beans, or nuts, along with fish or seafood, lean poultry, and low-fat or fat-free dairy products.  There is no specific recommended daily or weekly amount of red meat consumption for a heart-healthy diet. However, the AHA recommends choosing lean meats and poultry without skin and trimming visible fat. It is also suggested to limit processed meats, such as bacon, sausage, and deli meats, as they are high in sodium and preservatives.  The DASH eating plan, which scored a perfect 100 in the AHA's dietary guidance, emphasizes whole grains, fruits, and vegetables, and includes lean protein sources like poultry, fish, and low-fat dairy products. Red meat is not a prominent feature of this eating plan, and it is recommended to limit its consumption.  In general, a heart-healthy diet should focus on whole, unprocessed foods, and limit the intake of saturated fats, added sugars, and sodium. It is also important to maintain a healthy weight, engage in regular physical activity, and manage stress to reduce the risk of heart disease.  Sources:  American Heart Association. (2023). Here's How 10 Popular Diets Scored for Heart Health. Retrieved from <https://www.heart.org/en/news/2023/04/27/heres-how-10-popular-diets-scored-for-heart-health>  American Heart Association. (2023). 2023 AHA Presidential Advisory: Dietary Guidance to Improve Cardiovascular Health. Retrieved from <https://www.ahajournals.org/doi/full/10.1161/CIR.0000000000001146>  American Heart Association. (2024). Healthy Eating Plate. Retrieved from <https://www.heart.org/en/healthy-living/healthy-eating/eat-smart/nutrition-basics/healthy-eating-plate>  National Institutes of Health. (2024). DASH Eating Plan. Retrieved from <https://www.nhlbi.nih.gov/health-topics/dash-eating-plan> |
| **Question 10** | **What are some good snack options that are higher in protein?** |
| **Perplexity Answer 1** | Here are some healthy, high-protein snack options:  - Apple with peanut butter[9] - Apples and peanut butter provide a nutrient-dense snack with protein from the nut butter. The fiber from the apple helps keep you full.  - Hard-boiled eggs[9] - Eggs are a portable, high-protein snack that's easy to prepare in advance. One large egg contains about 6 grams of protein.  - Greek yogurt[9] - Greek yogurt is strained to remove excess liquid, resulting in a thicker consistency and higher protein content compared to regular yogurt. Top with fruit for added nutrients.  - Nut butters[9] - Single-serving packs of nut butters like peanut or almond butter provide a convenient, protein-rich snack. Look for varieties with minimal added ingredients.  - Protein shakes[9] - Blending protein powder with milk or water makes a quick, portable snack. Whey protein in particular may help with feelings of fullness.  - Edamame[9] - These immature soybeans in the pod are a good source of plant-based protein. Edamame is often served steamed as a snack.  - Lentil salad[9] - Lentils are a great source of protein and fiber. Toss cooked lentils with vegetables, herbs, and a light dressing for a satisfying snack.  The key is choosing snacks that provide a good amount of protein along with other beneficial nutrients like fiber, healthy fats, vitamins and minerals. Combining protein with complex carbs or produce helps keep you full and satisfied between meals.  Citations:  [1] https://www.yelp.com/biz/aman-cafe-oakland?adjust_creative=6zagVcKwffYZAA-51mlGNg  [2] https://www.yelp.com/biz/ok-s-deli-oakland?adjust_creative=6zagVcKwffYZAA-51mlGNg  [3] https://www.yelp.com/biz/kitava-oakland-2?adjust_creative=6zagVcKwffYZAA-51mlGNg  [4] https://www.yelp.com/biz/cholita-linda-oakland?adjust_creative=6zagVcKwffYZAA-51mlGNg  [5] https://www.yelp.com/biz/bakesale-betty-oakland?adjust_creative=6zagVcKwffYZAA-51mlGNg  [6] https://www.yelp.com/biz/gangnam-jajang-oakland?adjust_creative=6zagVcKwffYZAA-51mlGNg  [7] https://www.yelp.com/biz/burma-bites-oakland-4?adjust_creative=6zagVcKwffYZAA-51mlGNg  [8] https://www.yelp.com/biz/mariposa-baking-company-oakland?adjust_creative=6zagVcKwffYZAA-51mlGNg  [9] https://www.healthline.com/nutrition/healthy-high-protein-snacks  [10] https://www.verywellhealth.com/healthy-high-protein-snacks-8536131  [11] https://www.health.com/healthy-high-protein-snacks-7504806  [12] https://www.runfasteatslow.com/blog/top-11-high-protein-snacks-for-runners  [13] https://www.prevention.com/food-nutrition/healthy-eating/g26013537/healthy-high-protein-snacks/ |
| **Perplexity Answer 2** | Here are some healthy, high-protein snack options:  - Apple with peanut butter[9] - Apples and peanut butter provide a nutrient-dense snack with protein from the nut butter. The fiber from the apple helps keep you full.  - Hard-boiled eggs[9] - Eggs are a portable, high-protein snack that's easy to prepare in advance. One large egg contains about 6 grams of protein.  - Greek yogurt[9] - Greek yogurt is strained to remove liquid whey, resulting in a thicker consistency and higher protein content compared to regular yogurt. Top with fruit for added nutrients.  - Edamame[9] - These immature soybeans in the pod are high in protein, vitamins and minerals. They make for a quick, easy snack that can be purchased precooked and frozen.  - Nut butters[9] - Peanut, almond and other nut butters provide protein, healthy fats, fiber and various vitamins and minerals. Look for single-serving packs or bring a small container to dip apple slices or celery sticks.  - Protein shakes[9] - Blend protein powder with milk or water for a quick, portable protein boost. Whey protein in particular may help with feelings of fullness.  - Nuts and seeds[9] - Almonds, pumpkin seeds, and other nuts and seeds are high in protein, fiber and healthy fats. Portion out servings in advance to avoid overeating.  The key is choosing snacks that provide a good amount of protein along with other beneficial nutrients like fiber, healthy fats, vitamins and minerals. Combining protein with complex carbs or produce can help keep you full and satisfied between meals.  Citations:  [1] https://www.yelp.com/biz/aman-cafe-oakland?adjust_creative=6zagVcKwffYZAA-51mlGNg  [2] https://www.yelp.com/biz/ok-s-deli-oakland?adjust_creative=6zagVcKwffYZAA-51mlGNg  [3] https://www.yelp.com/biz/kitava-oakland-2?adjust_creative=6zagVcKwffYZAA-51mlGNg  [4] https://www.yelp.com/biz/cholita-linda-oakland?adjust_creative=6zagVcKwffYZAA-51mlGNg  [5] https://www.yelp.com/biz/bakesale-betty-oakland?adjust_creative=6zagVcKwffYZAA-51mlGNg  [6] https://www.yelp.com/biz/gangnam-jajang-oakland?adjust_creative=6zagVcKwffYZAA-51mlGNg  [7] https://www.yelp.com/biz/burma-bites-oakland-4?adjust_creative=6zagVcKwffYZAA-51mlGNg  [8] https://www.yelp.com/biz/mariposa-baking-company-oakland?adjust_creative=6zagVcKwffYZAA-51mlGNg  [9] https://www.healthline.com/nutrition/healthy-high-protein-snacks  [10] https://www.verywellhealth.com/healthy-high-protein-snacks-8536131  [11] https://www.health.com/healthy-high-protein-snacks-7504806  [12] https://www.runfasteatslow.com/blog/top-11-high-protein-snacks-for-runners  [13] https://www.prevention.com/food-nutrition/healthy-eating/g26013537/healthy-high-protein-snacks/ |
| **Perplexity Answer 3** | Here are some healthy, high-protein snack options:  - Apple with peanut butter[9] - Apples and peanut butter provide a nutrient-dense snack with protein from the nut butter. The fiber from the apple helps keep you full.  - Hard-boiled eggs[9] - Eggs are a portable, high-protein snack that's easy to prepare in advance. One large egg contains about 6 grams of protein.  - Greek yogurt[9] - Greek yogurt is strained to remove excess liquid, resulting in a thicker consistency and higher protein content compared to regular yogurt. Top with fruit for added nutrients.  - Edamame[9] - These immature soybeans in the pod are high in protein, vitamins and minerals. They make for a quick, easy snack that can be purchased precooked and frozen.  - Nut butters[9] - Single-serving packs of nut butters like peanut or almond butter are portable and nutrient-dense, providing protein, healthy fats, vitamins and minerals. Pair with apple slices or whole grain crackers.  - Protein shakes[9] - Blending protein powder with milk or water makes for a quick, portable snack that provides 20-25 grams of protein per scoop. Add fruit for extra nutrients.  - Lentil salad[9] - Lentils are a great source of plant-based protein. Toss cooked lentils with diced veggies, olive oil and vinegar for a satisfying, high-protein snack.  The key is choosing snacks that provide a good amount of protein along with other beneficial nutrients like fiber, healthy fats, vitamins and minerals to keep you full and satisfied between meals.  Citations:  [1] https://www.yelp.com/biz/aman-cafe-oakland?adjust_creative=6zagVcKwffYZAA-51mlGNg  [2] https://www.yelp.com/biz/ok-s-deli-oakland?adjust_creative=6zagVcKwffYZAA-51mlGNg  [3] https://www.yelp.com/biz/kitava-oakland-2?adjust_creative=6zagVcKwffYZAA-51mlGNg  [4] https://www.yelp.com/biz/cholita-linda-oakland?adjust_creative=6zagVcKwffYZAA-51mlGNg  [5] https://www.yelp.com/biz/bakesale-betty-oakland?adjust_creative=6zagVcKwffYZAA-51mlGNg  [6] https://www.yelp.com/biz/gangnam-jajang-oakland?adjust_creative=6zagVcKwffYZAA-51mlGNg  [7] https://www.yelp.com/biz/burma-bites-oakland-4?adjust_creative=6zagVcKwffYZAA-51mlGNg  [8] https://www.yelp.com/biz/mariposa-baking-company-oakland?adjust_creative=6zagVcKwffYZAA-51mlGNg  [9] https://www.healthline.com/nutrition/healthy-high-protein-snacks  [10] https://www.verywellhealth.com/healthy-high-protein-snacks-8536131  [11] https://www.health.com/healthy-high-protein-snacks-7504806  [12] https://www.runfasteatslow.com/blog/top-11-high-protein-snacks-for-runners  [13] https://www.prevention.com/food-nutrition/healthy-eating/g26013537/healthy-high-protein-nacks/ |
| **GPT4o Answer 1** | Here are some great snack options that are high in protein:  1. Greek Yogurt: High in protein and can be paired with fruits or nuts.  2. Cottage Cheese: A versatile snack that can be eaten plain or with fruits.  3. Hard-Boiled Eggs: Easy to prepare and portable.  4. Nuts and Seeds: Almonds, walnuts, pumpkin seeds, and sunflower seeds are good options.  5. Cheese: String cheese or cheese cubes are convenient and high in protein.  6. Edamame: Steamed soybeans are a great plant-based protein source.  7. Protein Bars: Choose ones with minimal added sugar and high protein content.  8. Chickpeas: Roasted chickpeas are crunchy and protein-rich.  9. Tuna or Salmon Packs: Convenient and can be eaten alone or with whole-grain crackers.  10. Jerky: Beef, turkey, or chicken jerky are high-protein, portable snacks.  11. Protein Smoothies: Made with protein powder, milk or a milk alternative, and fruits.  12. Hummus with Veggies: Hummus is a protein-rich dip that pairs well with vegetables.  13. Quark: A type of fresh dairy product, similar to yogurt but higher in protein.  14. Seitan: High in protein and can be prepared in various ways.  15. Peanut Butter: Spread on whole-grain bread or apple slices for a protein boost.  These snacks not only help to keep you full but also provide essential nutrients to support your overall health. |
| **GPT4o Answer 2** | Here are some high-protein snack options that are both nutritious and delicious:  1. Greek Yogurt: Rich in protein and probiotics, Greek yogurt can be topped with fruits or nuts for added flavor and nutrition.  2. Cottage Cheese: Another dairy option high in protein. Pair it with fresh fruit or a sprinkle of seeds for a tasty snack.  3. Hard-Boiled Eggs: Easy to prepare and pack, hard-boiled eggs are an excellent source of protein and healthy fats.  4. Edamame: These young soybeans are high in protein and fiber, making them a filling and healthy snack.  5. Nuts and Seeds: Almonds, walnuts, sunflower seeds, and pumpkin seeds are protein-rich and provide healthy fats and fiber.  6. Jerky: Beef, turkey, or chicken jerky are portable and high in protein, though it's best to choose low-sodium options.  7. Protein Bars: Look for bars with natural ingredients and a good protein-to-carb ratio to keep you satisfied.  8. Hummus with Veggies: Chickpeas in hummus provide protein and pairing it with fresh vegetables adds fiber and nutrients.  9. Tuna or Salmon Packets: These convenient packets are high in protein and omega-3 fatty acids.  10. Protein Smoothies: Blend your favorite protein powder with milk or a milk alternative and add some fruits or spinach for extra nutrients.  11. Chia Pudding: Made by soaking chia seeds in milk, this pudding is high in protein, fiber, and omega-3 fatty acids.  12. String Cheese or Cheese Slices: Convenient and rich in protein and calcium.  13. Roasted Chickpeas: Crunchy and satisfying, roasted chickpeas are a high-protein, high-fiber snack.  14. Quinoa Salad: A small serving of quinoa mixed with veggies and a protein source like beans or chicken makes for a great snack.  15. Boiled Soybeans (Edamame): Packed with protein and fiber, edamame is a tasty and nutritious option.  16. Peanut Butter or Almond Butter with Apple Slices: The nut butter provides protein and healthy fats, while the apple adds fiber and vitamins.  These snacks are not only high in protein but also provide a balance of other essential nutrients, making them great options for maintaining energy levels and satiety throughout the day. |
| **GPT4o Answer 3** | Here are some high-protein snack options:  1. Greek Yogurt: A serving of Greek yogurt can contain up to 15-20 grams of protein.  2. Cottage Cheese: Half a cup of cottage cheese provides about 14 grams of protein.  3. Hard-Boiled Eggs: Each hard-boiled egg contains around 6 grams of protein.  4. Edamame: One cup of cooked edamame has about 17 grams of protein.  5. Protein Bars: Look for bars with at least 10 grams of protein and minimal added sugars.  6. Jerky: Beef, turkey, or chicken jerky can offer 9-10 grams of protein per ounce.  7. Nuts and Seeds: Almonds, peanuts, and pumpkin seeds are great options, with a quarter cup providing around 7-8 grams of protein.  8. Hummus with Veggies: Two tablespoons of hummus contain about 2 grams of protein, plus the added benefit from the veggies.  9. Tuna or Salmon Pouches: These convenient packs can provide 15-20 grams of protein per serving.  10. Protein Smoothies: Blend protein powder with milk or a milk alternative and add fruits or vegetables for a nutritious snack.  11. Cheese Slices or Sticks: String cheese or cheese cubes can provide around 6-8 grams of protein per serving.  12. Chia Pudding: Made with chia seeds and milk, this can offer around 5 grams of protein per serving.  13. Roasted Chickpeas: A half-cup serving can provide around 7 grams of protein.  14. Tofu: Cubes of baked or sautéed tofu can be a high-protein snack, offering around 10 grams per half-cup serving.  These snacks are not only high in protein but also convenient and easy to prepare or carry with you. |
| **Llama 3 Answer 1** | Here are some tasty and convenient snack options that are higher in protein:  1. Nuts and seeds: Almonds (6 grams of protein per ounce), cashews (5 grams), pumpkin seeds (7 grams), chia seeds (5 grams), and hemp seeds (10 grams).  2. Jerky: Beef, turkey, or chicken jerky (9-11 grams of protein per ounce). Look for low-sodium options.  3. Hard-boiled eggs: 6-7 grams of protein per egg.  4. Greek yogurt: 20 grams of protein per 6-ounce serving. Choose a brand with live and active cultures.  5. Cottage cheese: 28 grams of protein per cup. Look for single-ingredient, unflavored options.  6. Protein shakes: Whey protein, casein protein, or plant-based options like pea or rice protein (varies by brand and serving size).  7. Edamame: 10 grams of protein per 1/2 cup cooked.  8. Canned tuna: 25 grams of protein per 3-ounce serving. Look for low-mercury options.  9. Protein bars: Look for bars with minimal added sugars and made with wholesome ingredients (varies by brand and serving size).  10. Beans: Black beans, chickpeas, or kidney beans (5-6 grams of protein per 1/2 cup cooked).  11. Protein-fortified nut butters: Peanut butter with protein (8 grams per 2 tablespoons) or almond butter with protein (6 grams per 2 tablespoons).  12. Quinoa bites: Made with quinoa, nuts, and seeds, these bite-sized energy balls can provide up to 5 grams of protein each.  13. Meat snacks: Turkey or chicken slices (5-6 grams of protein per ounce) or meat-based snack sticks (varies by brand and serving size).  14. Protein-rich granola: Made with nuts, seeds, and protein powder (varies by brand and serving size).  15. Soy products: Soy nuts (10 grams of protein per ounce), soy milk, or tofu (20 grams of protein per 3-ounce serving).  Remember to always check the nutrition label to ensure the snack you choose meets your protein needs and dietary preferences. |
| **Llama 3 Answer 2** | Here are some delicious and convenient snack options that are higher in protein:  Dairy and Eggs  1. Greek yogurt (20-25 grams of protein per 6-ounce serving)  2. Cottage cheese (28-30 grams of protein per cup)  3. Hard-boiled eggs (6-7 grams of protein per egg)  4. Milk (8-9 grams of protein per cup)  Nuts and Seeds  1. Almonds (6 grams of protein per ounce)  2. Cashews (5 grams of protein per ounce)  3. Chia seeds (5 grams of protein per ounce)  4. Hemp seeds (10 grams of protein per ounce)  5. Pumpkin seeds (7 grams of protein per ounce)  Meat and Poultry  1. Beef or turkey jerky (10-15 grams of protein per ounce)  2. Protein-rich meat snacks like pepperoni or salami (6-8 grams of protein per ounce)  3. Turkey or chicken breast slices (3-4 grams of protein per ounce)  Legumes  1. Edamame (10-12 grams of protein per cup)  2. Lentil or black bean chips (5-6 grams of protein per ounce)  3. Roasted chickpeas (5-6 grams of protein per ounce)  Protein Bars  1. Look for bars with at least 15 grams of protein and minimal added sugars. Some good options include RXBAR, Quest Bar, and Kind Bar.  Other Options  1. Protein shakes or smoothies (varies based on brand and type)  2. Canned tuna or salmon (20-25 grams of protein per 3-ounce serving)  3. Protein-rich granola (5-6 grams of protein per ounce)  4. High-protein crackers like rice cakes or energy bars (2-3 grams of protein per ounce)  Remember to always check the nutrition label to ensure the snack you choose meets your protein needs and preferences. |
| **Llama 3 Answer 3** | Here are some delicious and convenient snack options that are higher in protein:  1. Nuts and seeds: Almonds (6g protein per ounce), cashews (5g protein per ounce), pumpkin seeds (7g protein per ounce), and chia seeds (5g protein per ounce) are all high in protein and healthy fats.  2. Jerky: Beef, turkey, or chicken jerky is a tasty and portable snack that's high in protein (around 10-15g per ounce).  3. Hard-boiled eggs: A large egg has about 6g of protein, making them a convenient and protein-rich snack.  4. Greek yogurt: A 6-ounce serving of Greek yogurt typically has around 15-20g of protein. Look for brands with live and active cultures for added benefits.  5. Cottage cheese: A cup of cottage cheese contains around 28g of protein, making it an excellent snack for muscle growth and repair.  6. Protein shakes: Whey protein, casein protein, or plant-based protein powders like pea or rice protein can be a quick and easy snack option. Look for a protein powder that's low in sugar and made with wholesome ingredients.  7. Edamame: These boiled soybeans are a tasty and protein-rich snack (around 17g of protein per cup).  8. Canned tuna: A 3-ounce serving of canned tuna has around 20g of protein. Look for tuna packed in water or olive oil for added health benefits.  9. Protein bars: Look for bars made with wholesome ingredients and minimal added sugars. Some good options include RXBAR, Quest Bar, and Kind Bar.  10. Trail mix with protein-rich ingredients: A mix of nuts, seeds, and dried fruit with added protein-rich ingredients like peanut butter or energy balls made with protein powder can be a satisfying snack.  11. Hummus and veggies: A serving of hummus (around 2 tablespoons) has around 5g of protein. Pair it with carrot sticks, celery, or cucumber slices for a healthy and filling snack.  12. Protein-fortified nut butter: Some nut butters, like peanut butter or almond butter, have added protein to boost their protein content.  Remember to always check the nutrition label to ensure the snack you choose meets your protein needs and dietary preferences. |
| **LLaMA3+RAG model Answer 1** | When it comes to snacking, incorporating protein-rich options can help keep you full and satisfied between meals. According to the American Heart Association, here are some excellent snack options that are higher in protein:  1. Fat-free/low-fat cottage cheese: Paired with sliced pears, this snack provides a good amount of protein to keep you full. One cup of cottage cheese contains approximately 28 grams of protein. (Source: USDA)  2. Unsalted nuts and seeds: Almonds, cashews, pumpkin seeds, and chia seeds are all great sources of protein. For example, one ounce of almonds contains about 6 grams of protein. (Source: USDA)  3. Canned very low sodium tuna or low-sodium salmon: Spread on whole-grain crackers, these fish provide a protein-rich snack. A 3-ounce serving of tuna contains about 20 grams of protein. (Source: USDA)  4. Fat-free/low-fat cheese: Enjoyed on its own or paired with whole-grain crackers, cheese is a convenient protein-rich snack. One ounce of cheddar cheese contains about 6 grams of protein. (Source: USDA)  5. Rice cakes with peanut butter: While peanut butter is high in calories, it's also a good source of protein. Look for natural peanut butter with minimal added ingredients. Two tablespoons of peanut butter contain about 8 grams of protein. (Source: USDA)  6. Hummus: Made from chickpeas, hummus is a tasty and protein-rich dip for vegetables. A 2-tablespoon serving of hummus contains about 2 grams of protein. (Source: USDA)  7. Roasted chickpeas: These crunchy snacks are a good source of protein and can be seasoned with herbs and spices for added flavor. One cup of roasted chickpeas contains about 15 grams of protein. (Source: USDA)  Remember to always check the Nutrition Facts label to ensure the snack you choose is within your daily calorie and nutrient needs.  Sources:  American Heart Association. (2024). Healthy Snacking. Retrieved from <https://www.heart.org/en/healthy-living/healthy-eating/add-color/healthy-snacking>  American Heart Association. (2024). Healthy Post-Play Snacks. Retrieved from <https://www.heart.org/en/healthy-living/healthy-eating/eat-smart/nutrition-basics/healthy-post-play-snacks>  United States Department of Agriculture. (2024). FoodData Central. Retrieved from <https://fdc.nal.usda.gov/> |
| **LLaMA3+RAG model Answer 2** | When it comes to snack options that are higher in protein, there are several excellent choices that can help keep you full and satisfied between meals. According to the American Heart Association, here are some high-protein snack options:  1. Fat-free/low-fat cottage cheese: Paired with sliced pears, this snack is not only delicious but also provides a good amount of protein. One cup of cottage cheese contains approximately 28 grams of protein. (Source: USDA)  2. Unsalted nuts and seeds: Almonds, cashews, pumpkin seeds, and chia seeds are all high in protein and can be enjoyed as a quick snack. For example, one ounce of almonds contains about 6 grams of protein. (Source: USDA)  3. Canned very low sodium tuna or low-sodium salmon: When paired with whole-grain crackers, these fish options provide a protein-rich snack. A 3-ounce serving of tuna contains about 20 grams of protein, while a 3-ounce serving of salmon contains about 21 grams of protein. (Source: USDA)  4. Fat-free/low-fat cheese: A small block of fat-free/low-fat cheese can provide a quick protein boost. One ounce of cheddar cheese contains about 6 grams of protein. (Source: USDA)  5. Greek yogurt: While not explicitly mentioned in the context, Greek yogurt is a high-protein snack option that pairs well with fruit or nuts. A 6-ounce serving of Greek yogurt contains about 15 grams of protein. (Source: USDA)  6. Hummus: Made from chickpeas, hummus is a tasty and protein-rich dip for vegetables. A 2-tablespoon serving of hummus contains about 2 grams of protein. (Source: USDA)  7. Roasted chickpeas: These crunchy snacks are not only high in protein but also fiber and healthy fats. A 1/2 cup serving of roasted chickpeas contains about 5 grams of protein. (Source: USDA)  Remember to always check the Nutrition Facts label to ensure that your snack of choice is within your daily calorie and nutrient needs.  Sources:  American Heart Association. (2024). Healthy Snacking. Retrieved from <https://www.heart.org/en/healthy-living/healthy-eating/add-color/healthy-snacking>  American Heart Association. (2024). Healthy Post-Play Snacks. Retrieved from <https://www.heart.org/en/healthy-living/healthy-eating/eat-smart/nutrition-basics/healthy-post-play-snacks>  United States Department of Agriculture. (2024). FoodData Central. Retrieved from <https://fdc.nal.usda.gov/> |
| **LLaMA3+RAG model Answer 3** | When it comes to snacking, incorporating protein-rich options can help keep you fuller for longer and support overall health. According to the American Heart Association, here are some excellent snack options that are higher in protein:  1. Fat-free/low-fat cottage cheese: Paired with pears, this snack is not only crunchy but also provides a good amount of protein. A single cup of cottage cheese contains about 28 grams of protein. (Source: USDA)  2. Unsalted nuts and seeds: Almonds, cashews, pumpkin seeds, and chia seeds are all great sources of protein. For example, a 1-ounce serving of almonds (about 23 nuts) contains 6 grams of protein. (Source: USDA)  3. Canned very low sodium tuna or low-sodium salmon: When paired with whole-grain crackers, these fish provide a protein-rich snack. A 3-ounce serving of tuna contains about 20 grams of protein, while a 3-ounce serving of salmon contains about 21 grams of protein. (Source: USDA)  4. Fat-free/low-fat cheese: A 1-ounce serving of fat-free/low-fat cheese contains about 6-7 grams of protein. Look for options with lower sodium content. (Source: USDA)  5. Rice cakes with peanut butter: While peanut butter is high in calories, it's also a good source of protein. A 2-tablespoon serving of peanut butter contains about 8 grams of protein. Pair it with whole-grain rice cakes for a satisfying snack. (Source: USDA)  6. Hummus: Made from chickpeas, hummus is a tasty and protein-rich dip for vegetables. A 2-tablespoon serving of hummus contains about 5 grams of protein. (Source: USDA)  7. Greek yogurt: While not mentioned in the provided context, Greek yogurt is an excellent protein-rich snack option. A 6-ounce serving of non-fat Greek yogurt contains about 15 grams of protein. (Source: USDA)  Remember to always check the Nutrition Facts label to ensure the snack you choose aligns with your dietary needs and preferences  Sources:  1. American Heart Association. (2024). Healthy Snacking. Retrieved from <https://www.heart.org/en/healthy-living/healthy-eating/add-color/healthy-snacking>  2. American Heart Association. (2024). Healthy Post-Play Snacks. Retrieved from <https://www.heart.org/en/healthy-living/healthy-eating/eat-smart/nutrition-basics/healthy-post-play-snacks>  3. United States Department of Agriculture. (2024). FoodData Central. Retrieved from <https://fdc.nal.usda.gov/> |
| **Question 11** | **What are some good plant based protein sources?** |
| **Perplexity Answer 1** | Here are some of the best plant-based protein sources:  1. Soy products like tofu, tempeh, and edamame are among the richest sources of protein, with tofu containing about 10g of protein per 1⁄2 cup, edamame 8.5g per 1⁄2 cup, and tempeh 15g per 1⁄2 cup[1][3].  2. Legumes such as lentils, chickpeas, and beans are excellent sources of plant-based protein. For example, lentils contain 18g of protein per cooked cup[3][4].  3. Nuts and seeds like almonds, walnuts, chia seeds, flax seeds, hemp seeds, and pumpkin seeds are protein-packed. One ounce of nuts contains 5-7g of protein[2][4].  4. Whole grains like quinoa, wild rice, and oats provide protein. Quinoa is a complete protein, meaning it contains all nine essential amino acids[2][4].  5. Other good plant-based protein sources include spirulina, nutritional yeast, seitan (wheat gluten), and protein-rich vegetables like broccoli[3][5].  The key is to eat a variety of plant-based proteins throughout the day to ensure you get all the essential amino acids your body needs. Combining different protein sources, such as grains and legumes, can also create a complete protein[1][4].  Citations:  [1] https://www.onepeloton.com/blog/plant-based-protein-sources/  [2] https://www.healthline.com/nutrition/protein-for-vegans-vegetarians  [3] https://www.medicalnewstoday.com/articles/321474  [4] https://www.chhs.colostate.edu/krnc/monthly-blog/plant-based-protein-a-simple-guide-to-getting-enough/  [5] https://www.eatingwell.com/plant-based-foods-with-more-protein-than-an-egg-8424134 |
| **Perplexity Answer 2** | Here are some of the best plant-based protein sources:  - Tofu, tempeh, and edamame - Soy products are among the richest sources of protein in a plant-based diet, with firm tofu containing about 10g of protein per 1⁄2 cup, edamame 8.5g per 1⁄2 cup, and tempeh 15g per 1⁄2 cup[1][3].  - Lentils - Lentils are a type of legume that provide about 9g of protein per 1⁄2 cup when cooked[3].  - Chickpeas - Also known as garbanzo beans, chickpeas contain 7.3g of protein per 1⁄2 cup when cooked[3].  - Quinoa - This grain is a complete protein, meaning it contains all nine essential amino acids. Cooked quinoa provides 8g of protein per cup[3][4].  - Beans - Beans are an excellent source of plant-based protein. For example, black beans contain 7.6g of protein per 1⁄2 cup when cooked[3].  - Nuts and seeds - Almonds, chia seeds, hemp seeds, and peanuts are all good sources of plant protein. For example, 2 tablespoons of chia seeds provide 4g of protein[3][4].  - Nutritional yeast - This ingredient provides 8g of protein per 2 tablespoons and can be used to add a savory, ""cheesy"" flavor to dishes[2].  The key is to eat a variety of plant-based protein sources throughout the day to ensure you get all the essential amino acids your body needs[4]. Combining incomplete proteins, like rice and beans, can also create a complete protein source[3].  Citations:  [1] https://www.heart.org/en/healthy-living/healthy-eating/eat-smart/nutrition-basics/plant-based-protein-infographic  [2] https://www.onepeloton.com/blog/plant-based-protein-sources/  [3] https://www.medicalnewstoday.com/articles/321474  [4] https://www.chhs.colostate.edu/krnc/monthly-blog/plant-based-protein-a-simple-guide-to-getting-enough/  [5] https://www.healthline.com/nutrition/protein-for-vegans-vegetarians |
| **Perplexity Answer 3** | Here are some excellent plant-based protein sources:  - Tofu, tempeh, and edamame - Soy products are among the richest sources of plant-based protein, with firm tofu containing about 10g of protein per 1⁄2 cup, edamame 8.5g per 1⁄2 cup, and tempeh 15g per 1⁄2 cup[1][2].  - Lentils and chickpeas - Legumes like lentils and chickpeas are high in protein, with 9g and 7.5g per 1⁄2 cup respectively[2][3].  - Quinoa - This grain is a complete protein source, meaning it contains all nine essential amino acids. Cooked quinoa contains 8g of protein per cup[2][3].  - Chia and hemp seeds - Chia seeds provide 2g of protein per tablespoon while hemp seeds offer 5g per tablespoon. They are both complete protein sources[2][4].  - Beans and rice - While incomplete proteins on their own, when eaten together beans and rice provide a full complement of amino acids. A cup of the combination provides 7g of protein[2].  - Seitan - Made from wheat gluten, seitan is extremely high in protein, with 17g per 2-ounce serving, more than an egg[5].  - Peas - Green peas pack 8g of protein per 1-cup serving along with 9g of fiber[5].  Other good plant-based protein sources include nuts, seeds, whole grains like oats and wild rice, and soy milk[3][4]. Eating a variety of these foods throughout the day ensures you get enough protein and all the essential amino acids your body needs[3].  Citations:  [1] https://www.heart.org/en/healthy-living/healthy-eating/eat-smart/nutrition-basics/plant-based-protein-infographic  [2] https://www.medicalnewstoday.com/articles/321474  [3] https://www.chhs.colostate.edu/krnc/monthly-blog/plant-based-protein-a-simple-guide-to-getting-enough/  [4] https://www.healthline.com/nutrition/protein-for-vegans-vegetarians  [5] https://www.eatingwell.com/plant-based-foods-with-more-protein-than-an-egg-8424134 |
| **GPT4o Answer 1** | Plant-based protein sources are diverse and can be incorporated into various meals to ensure adequate protein intake. Here are some good options:  1. Legumes:  - Lentils: High in protein and fiber, lentils are versatile and can be used in soups, stews, and salads.  - Chickpeas: Also known as garbanzo beans, chickpeas can be used in hummus, salads, and stews.  - Black beans: Rich in protein and fiber, black beans are great in soups, salads, and as a side dish.  - Peas: Green peas are a good source of protein and can be added to various dishes.  2. Nuts and Seeds:  - Almonds: High in protein and healthy fats, almonds are great as a snack or added to meals.  - Chia seeds: These tiny seeds are packed with protein, fiber, and omega-3 fatty acids. They can be added to smoothies, oatmeal, or used in chia pudding.  - Hemp seeds: Rich in protein and healthy fats, hemp seeds can be sprinkled on salads, yogurt, or blended into smoothies.  - Pumpkin seeds: High in protein and magnesium, pumpkin seeds are a nutritious snack or salad topping.  3. Grains:  - Quinoa: A complete protein containing all nine essential amino acids, quinoa can be used as a base for salads, bowls, or as a side dish.  - Farro: An ancient grain with a nutty flavor and high protein content, farro is excellent in salads and soups.  - Amaranth: A gluten-free grain that is high in protein and can be used in porridge, soups, or as a side dish.  4. Soy Products:  - Tofu: A versatile soy product that can be grilled, stir-fried, or added to soups and salads.  - Tempeh: Fermented soybeans that are high in protein and have a nutty flavor, great for stir-fries and sandwiches.  - Edamame: Young soybeans that can be steamed and eaten as a snack or added to salads and stir-fries.  5. Vegetables:  - Spinach: While not extremely high in protein, spinach can contribute to overall protein intake and is rich in other nutrients.  - Broccoli: Contains protein and fiber, making it a nutritious addition to meals.  6. Plant-Based Protein Powders:  - Pea protein: Derived from yellow split peas, this protein powder can be added to smoothies or baked goods.  - Rice protein: Made from brown rice, it’s a good option for those with allergies to other protein sources.  Incorporating a variety of these plant-based protein sources into your diet can help ensure you get enough protein along with other essential nutrients. |
| **GPT4o Answer 2** | Plant-based protein sources are diverse and can easily provide the necessary protein for a balanced diet. Here are some of the best options:  Legumes  1. Lentils: High in protein and fiber, versatile for soups, stews, and salads.  2. Chickpeas (Garbanzo beans): Great for hummus, salads, and stews.  3. Black beans: Excellent in soups, salads, and Mexican dishes.  4. Kidney beans: Common in chili and soups.  Nuts and Seeds  5. Almonds: Good for snacking, almond butter, and baking.  6. Chia seeds: High in protein and omega-3 fatty acids, great in smoothies, puddings, and yogurt.  7. Hemp seeds: Complete protein, good for sprinkling on salads, cereals, and smoothies.  8. Pumpkin seeds: Great snack, can be added to salads and baking.  Whole Grains  9. Quinoa: A complete protein, used in salads, bowls, and as a rice substitute.  10. Farro: Nutty flavor, good for salads and as a side dish.  11. Oats: Excellent for breakfast, baking, and smoothies.  Soy Products  12. Tofu: Versatile, can be used in stir-fries, soups, and as a meat substitute.  13. Tempeh: Fermented soybean product, great for sandwiches, stir-fries, and salads.  14. Edamame: Young soybeans, good as a snack or in salads.  Vegetables  15. Spinach: Contains protein, good in salads, smoothies, and cooked dishes.  16. Broccoli: High in protein, can be steamed, roasted, or used in stir-fries.  Other Sources  17. Nutritional yeast: High in protein and B vitamins, good for adding a cheesy flavor to dishes.  18. Seitan: Made from wheat gluten, very high in protein, used as a meat substitute in various dishes.  19. Green peas: Versatile, can be used in soups, stews, and as a side dish.  20. Spirulina: A blue-green algae, high in protein, used in smoothies and as a supplement.  These options not only provide a good amount of protein but also come with various other nutrients and health benefits, making them excellent choices for a plant-based diet. |
| **GPT4o Answer 3** | Plant-based protein sources are diverse and can provide a balanced diet with all essential amino acids. Here are some of the best options:  1. Legumes: Beans, lentils, chickpeas, and peas are excellent sources of protein. They are also high in fiber and various essential nutrients.  2. Tofu and Tempeh: Made from soybeans, tofu and tempeh are versatile and can be used in a variety of dishes. Tempeh, in particular, is fermented, which adds beneficial probiotics to the diet.  3. Edamame: These are young soybeans and are a great snack or addition to meals, providing a good amount of protein and fiber.  4. Quinoa: A complete protein, quinoa contains all nine essential amino acids. It's also high in fiber, iron, and magnesium.  5. Nuts and Seeds: Almonds, walnuts, chia seeds, flaxseeds, and hemp seeds are protein-rich and provide healthy fats. Chia and flaxseeds are also good sources of omega-3 fatty acids.  6. Seitan: Made from wheat gluten, seitan is very high in protein and can be used as a meat substitute in many recipes.  7. Nutritional Yeast: Often used as a cheese substitute, nutritional yeast is high in protein and B-vitamins, including B12 if fortified.  8. Green Peas: Surprisingly high in protein, peas can be added to many dishes or enjoyed as a side.  9. Spirulina: This blue-green algae is incredibly nutrient-dense, providing a high amount of protein along with vitamins and minerals.  10. Oats and Oatmeal: Besides being a good source of protein, oats are rich in fiber and can help in maintaining a healthy digestive system.  11. Hemp Seeds: These seeds provide a complete protein source and are rich in healthy fats, including omega-3 and omega-6 fatty acids.  12. Chickpea Flour: Used in various dishes, chickpea flour is a high-protein alternative to regular flour.  13. Amaranth: Like quinoa, amaranth is a complete protein and provides numerous other nutrients such as iron and magnesium.  14. Soy Milk: An excellent alternative to dairy milk, soy milk provides a good amount of protein and is often fortified with calcium and vitamin D.  15. Vegetables: Certain vegetables like broccoli, spinach, and Brussels sprouts have higher protein content compared to others.  Including a variety of these sources in your diet can help ensure you get a balanced intake of protein and other essential nutrients. |
| **Llama 3 Answer 1** | There are many delicious and nutritious plant-based protein sources to explore. Here are some high-protein foods that are perfect for vegans and vegetarians:  1. Legumes:  * Lentils (18g of protein per 1 cup cooked)  * Chickpeas (15g of protein per 1 cup cooked)  * Black beans (15g of protein per 1 cup cooked)  * Kidney beans (15g of protein per 1 cup cooked)  * Soybeans (29g of protein per 1 cup cooked)  2. Nuts and Seeds:  * Almonds (6g of protein per 1 ounce)  * Chia seeds (5g of protein per 1 ounce)  * Hemp seeds (10g of protein per 1 ounce)  * Pumpkin seeds (7g of protein per 1 ounce)  * Sunflower seeds (6g of protein per 1 ounce)  3. Whole Grains:  * Quinoa (8g of protein per 1 cup cooked)  * Amaranth (9g of protein per 1 cup cooked)  * Teff (7g of protein per 1 cup cooked)  4. Soy Products:  * Tofu (20g of protein per 3 ounces)  * Tempeh (15g of protein per 3 ounces)  * Edamame (17g of protein per 1 cup cooked)  5. Meat Alternatives:  * Seitan (21g of protein per 3 ounces)  * Veggie burgers (varies by brand and type)  6. Mushrooms:  * Shiitake mushrooms (2g of protein per 1 cup)  * Portobello mushrooms (4g of protein per 1 cup)  7. Vegetables:  * Broccoli (4g of protein per 1 cup)  * Spinach (5g of protein per 1 cup cooked)  * Kale (2g of protein per 1 cup cooked)  8. Plant-Based Protein Powders:  * Pea protein powder  * Hemp protein powder  * Brown rice protein powder  * Soy protein powder  Remember, you can combine different plant-based protein sources to meet your daily protein needs. For example, having a bowl of lentil soup with whole grain bread and a side of steamed broccoli can provide a good amount of protein.  I hope this helps! |
| **Llama3 Answer 2** | There are many delicious and nutritious plant-based protein sources to choose from. Here are some high protein, plant-based options:  1. Legumes:  * Lentils (18g protein per 1 cup cooked)  * Chickpeas (15g protein per 1 cup cooked)  * Black beans (15g protein per 1 cup cooked)  * Kidney beans (15g protein per 1 cup cooked)  * Soybeans (29g protein per 1 cup cooked)  2. Nuts and Seeds:  * Almonds (6g protein per 1 ounce)  * Chia seeds (5g protein per 1 ounce)  * Hemp seeds (10g protein per 1 ounce)  * Pumpkin seeds (7g protein per 1 ounce)  * Sunflower seeds (6g protein per 1 ounce)  3. Whole Grains:  * Quinoa (8g protein per 1 cup cooked)  * Amaranth (9g protein per 1 cup cooked)  * Teff (7g protein per 1 cup cooked)  4. Soy Products:  * Tofu (20g protein per 3 ounces)  * Tempeh (15g protein per 3 ounces)  * Edamame (17g protein per 1 cup cooked)  5. Meat Alternatives:  * Seitan (21g protein per 3 ounces)  * Veggie burgers (varies by brand, but often around 10g protein per patty)  6. Mushrooms:  * Shiitake mushrooms (2g protein per 1 cup cooked)  * Portobello mushrooms (4g protein per 1 cup cooked)  7. Other Plant-Based Protein Sources:  * Spirulina (16g protein per 1 tablespoon)  * Vegan protein powder (varies by brand and type)  * Broccoli (2g protein per 1 cup cooked)  * Spinach (3g protein per 1 cup cooked)  Remember, you don't need to get all your protein from a single source. Aim to include a variety of protein-rich foods in your diet to ensure you're getting enough protein overall. |
| **Llama Answer 3** | There are many delicious and nutritious plant-based protein sources to choose from. Here are some high-protein plant-based foods:  1. Legumes:  * Lentils (18g of protein per 1 cup cooked)  * Chickpeas (15g of protein per 1 cup cooked)  * Black beans (15g of protein per 1 cup cooked)  * Kidney beans (15g of protein per 1 cup cooked)  * Soybeans (29g of protein per 1 cup cooked)  2. Nuts and Seeds:  * Almonds (6g of protein per 1 ounce)  * Chia seeds (5g of protein per 1 ounce)  * Hemp seeds (10g of protein per 1 ounce)  * Pumpkin seeds (7g of protein per 1 ounce)  * Sunflower seeds (6g of protein per 1 ounce)  3. Whole Grains:  * Quinoa (8g of protein per 1 cup cooked)  * Amaranth (9g of protein per 1 cup cooked)  * Teff (7g of protein per 1 cup cooked)  4. Soy Products:  * Tofu (20g of protein per 3 oz serving)  * Tempeh (15g of protein per 3 oz serving)  * Edamame (17g of protein per 1 cup cooked)  5. Meat Alternatives:  * Seitan (21g of protein per 3 oz serving)  * Veggie burgers (varies by brand, but can be up to 10g of protein per patty)  6. Mushrooms:  * Shiitake mushrooms (2g of protein per 1 cup cooked)  * Portobello mushrooms (4g of protein per 1 cup cooked)  7. Vegan Protein Powder:  * Pea protein powder (20-25g of protein per scoop)  * Hemp protein powder (15-20g of protein per scoop)  * Brown rice protein powder (15-20g of protein per scoop)  Remember, you don't need to get all your protein from a single source. Aim to include a variety of protein-rich foods in your diet to ensure you're getting enough protein overall. |
| **LLaMA3+RAG model Answer 1** | Plant-based protein sources are an essential component of a healthy diet, and fortunately, there are numerous options available. According to the American Heart Association, some excellent plant-based protein sources include:  1. Beans and legumes: These are some of the richest sources of plant-based protein, fiber, and various essential nutrients. Examples include kidney beans, black beans, chickpeas, lentils, and edamame.  2. Broccoli: While not as high in protein as other options, broccoli is a cruciferous vegetable that contains a significant amount of protein, along with vitamins, minerals, and antioxidants.  3. Chickpeas: These versatile legumes are not only high in protein but also rich in fiber, vitamins, and minerals. They can be used in a variety of dishes, from hummus to salads.  4. Edamame: These boiled soybeans are a tasty and protein-rich snack or addition to meals.  5. Lentils: Another type of legume, lentils are packed with protein, fiber, and essential nutrients like iron and potassium.  6. Nut butter: While high in calories, nut butters like peanut butter, almond butter, and cashew butter are good sources of protein and healthy fats.  7. Nuts and seeds: Almonds, walnuts, chia seeds, flaxseeds, and hemp seeds are all high in protein and healthy fats, making them great additions to oatmeal, yogurt, or salads.  8. Oats: While not as high in protein as other options, oats are a good source of protein and fiber, making them a nutritious breakfast choice.  9. Peas: Fresh or frozen, peas are a low-calorie, high-protein addition to meals.  10. Quinoa: This protein-rich grain is also a complete protein, meaning it contains all nine essential amino acids that the body can't produce on its own.  11. Sorghum: A type of ancient grain, sorghum is high in protein and fiber, making it a nutritious alternative to traditional grains.  12. Soymilk: A plant-based milk alternative, soymilk is a good source of protein and can be used in cereal, smoothies, or as a dairy substitute.  13. Spinach: While not as high in protein as other leafy greens like kale, spinach is still a good source of protein and packed with vitamins and minerals.  14. Tempeh: A fermented soybean product, tempeh is a good source of protein and probiotics, which support gut health.  15. Tofu: A versatile and protein-rich soybean product, tofu can be used in a variety of dishes, from stir-fries to curries.  16. Veggie patties: Many veggie patties are made with protein-rich ingredients like beans, lentils, or soy, making them a convenient and protein-packed meal option.  Incorporating these plant-based protein sources into your diet can help you meet your daily protein needs while also providing a range of essential nutrients and health benefits.  Sources:  American Heart Association. (2024). Plant-Based Protein Infographic. Retrieved from <https://www.heart.org/en/healthy-living/healthy-eating/eat-smart/nutrition-basics/plant-based-protein-infographic>  National Institutes of Health. (2020). Protein in Diet. Retrieved from <https://medlineplus.gov/ency/article/002467.htm> |
| **LLaMA3+RAG model Answer 2** | When it comes to plant-based protein sources, there are numerous options to choose from, and they're not only rich in protein but also packed with fiber, vitamins, minerals, and other essential nutrients. According to the American Heart Association, some excellent plant-based protein sources include:  1. Beans and Legumes: These include kidney beans, black beans, chickpeas, lentils, and more. They're not only high in protein but also rich in fiber, potassium, and folate.  2. Broccoli: This cruciferous vegetable is a good source of protein, vitamins C and K, and fiber.  3. Chickpeas: With about 15 grams of protein per 1 cup serving, chickpeas are an excellent source of protein, fiber, and various vitamins and minerals.  4. Edamame: These boiled soybeans are a tasty and protein-rich snack, with about 17 grams of protein per 1 cup serving.  5. Lentils: With about 18 grams of protein per 1 cup serving, lentils are a great source of protein, fiber, and iron.  6. Nut Butter: While high in calories, nut butters like peanut butter, almond butter, and cashew butter are good sources of protein and healthy fats.  7. Nuts and Seeds: Almonds, chia seeds, hemp seeds, and pumpkin seeds are all high in protein and healthy fats.  8. Oats: While not as high in protein as other options, oats are a good source of protein, fiber, and various vitamins and minerals.  9. Peas: Fresh or frozen, peas are a low-calorie, high-protein snack, with about 9 grams of protein per 1 cup serving.  10. Quinoa: This complete protein is a great source of protein, fiber, and various vitamins and minerals.  11. Sorghum: This ancient grain is a good source of protein, fiber, and antioxidants.  12. Soymilk: A plant-based milk alternative, soymilk is a good source of protein and calcium.  13. Spinach: While not as high in protein as other leafy greens, spinach is a good source of protein, iron, and vitamins A and K.  14. Tempeh: A fermented soybean product, tempeh is a good source of protein, probiotics, and vitamins.  15. Tofu: A versatile and protein-rich food, tofu can be used in a variety of dishes, from stir-fries to curries.  16. Veggie Patties: Many veggie patties are made from protein-rich ingredients like beans, lentils, and soy.  Incorporating these plant-based protein sources into your diet can help you meet your daily protein needs while also providing a range of other essential nutrients.  Sources:  American Heart Association. (2024). Plant-Based Protein Infographic. Retrieved from <https://www.heart.org/en/healthy-living/healthy-eating/eat-smart/nutrition-basics/plant-based-protein-infographic> |
| **LLaMA3+RAG model Answer 3** | According to the American Heart Association, some excellent plant-based protein sources include:  1. Beans and legumes: These are some of the highest protein-containing plant-based foods, including kidney beans, black beans, chickpeas, lentils, and more. They're also rich in fiber, vitamins, and minerals.  2. Broccoli: This cruciferous vegetable is not only a good source of protein but also packed with vitamins C and K, and fiber.  3. Chickpeas: With about 15g of protein per 1 cup serving, chickpeas are an excellent addition to salads, stews, and curries.  4. Edamame: These boiled soybeans are a tasty and protein-rich snack, with about 17g of protein per 1 cup serving.  5. Lentils: With about 18g of protein per 1 cup serving, lentils are a great addition to soups, stews, and curries.  6. Nut butter: While high in calories, nut butters like peanut butter, almond butter, and cashew butter are good sources of protein, with about 8g per 2 tbsp serving.  7. Nuts and seeds: Almonds, chia seeds, hemp seeds, and pumpkin seeds are all high in protein, with about 5-8g per 1/4 cup serving.  8. Oats: While not as high in protein as other options, oats are a good source of protein, especially when combined with nuts and seeds.  9. Peas: Fresh or frozen, peas are a tasty and protein-rich addition to meals, with about 9g of protein per 1 cup serving.  10. Quinoa: This complete protein is a great source of all nine essential amino acids, with about 8g of protein per 1 cup serving.  11. Sorghum: This ancient grain is a good source of protein, with about 10g per 1 cup serving.  12. Soymilk: A plant-based milk alternative, soymilk is a good source of protein, with about 8g per 1 cup serving.  13. Spinach: While not as high in protein as other leafy greens, spinach is still a good source of protein, with about 5g per 1 cup serving.  14. Tempeh: A fermented soybean product, tempeh is a good source of protein, with about 15g per 3 oz serving.  15. Tofu: A versatile and protein-rich soybean product, tofu has about 20g of protein per 3 oz serving.  16. Veggie patties: Many veggie patties are made with protein-rich ingredients like beans, lentils, and soy, making them a convenient and protein-rich option.  These plant-based protein sources offer a variety of options for individuals looking to incorporate more protein into their diet while following a plant-based lifestyle.  Sources:  American Heart Association. (2024). Plant-Based Protein Infographic. Retrieved from <https://www.heart.org/en/healthy-living/healthy-eating/eat-smart/nutrition-basics/plant-based-protein-infographic> |
| **Question 12** | **How many meals a day should I eat for a heart healthy diet?** |
| **Perplexity Answer 1** | Here are some tips for how many meals to eat per day on a heart-healthy diet:  - Aim for 3 meals per day, with 1-2 healthy snacks in between if needed[1][2]. Eating regular meals helps keep your blood sugar stable and prevents overeating later in the day.  - Portion control is key. The sample 1,500 calorie heart-healthy meal plan includes 3 meals and 2 snacks[2]. Adjust portions up or down based on your calorie needs.  - Breakfast is important. Eating a nutritious breakfast helps control hunger and cravings later in the day[1][2]. Examples include oatmeal with fruit, whole grain cereal with milk, or eggs with whole grain toast.  - Snacks can be part of a heart-healthy diet. Healthy snack ideas include fresh fruit, raw veggies with hummus, unsalted nuts, or low-fat yogurt[1][2]. Portion sizes for snacks should be around 100-200 calories.  - Stay hydrated by drinking water throughout the day. Avoid sugary beverages which can contribute excess calories and sugar[1].  The key is to eat a balanced diet with a variety of nutrient-dense foods like fruits, vegetables, whole grains, lean proteins and healthy fats. Portion control and eating at regular intervals can help maintain healthy blood sugar levels and prevent overeating. Consult with your doctor or a registered dietitian to determine the right meal frequency and portions for your individual needs.  Citations:  [1] https://www.mayoclinic.org/diseases-conditions/heart-disease/in-depth/heart-healthy-diet/art-20046702  [2] https://www.eatingwell.com/article/7884046/heart-healthy-diet-plan-for-beginners/  [3] https://www.eatingwell.com/article/289245/7-day-heart-healthy-meal-plan-1200-calories/  [4] https://www.tasteofhome.com/collection/heart-healthy-meal-plan/  [5] https://www.medicalnewstoday.com/articles/cardiac-diet |
| **Perplexity Answer 2** | For a heart-healthy diet, it's recommended to eat 3 meals per day with 1-2 healthy snacks[1][2][3]. The key is to focus on nutrient-dense foods like:  - Vegetables and fruits (2.5-3 cups of vegetables and 1.5-2 cups of fruits per day)[1]  - Whole grains (at least half of your grains should be whole grains)[1]  - Lean protein like chicken, fish, beans, lentils, and nuts[1][2]  - Low-fat or fat-free dairy products[1]  Portion control is also important - don't overload your plate and be mindful of serving sizes[3]. Limit high-fat, high-sodium, and high-sugar foods[2][3]. Drink plenty of water throughout the day[2].  By eating 3 balanced meals and 1-2 healthy snacks, you can get all the nutrients you need while keeping your heart healthy. The sample meal plans provided show how to structure a heart-healthy day with breakfast, lunch, dinner and snacks[2][4][5].  Citations:  [1] https://www.nhlbi.nih.gov/health/educational/healthdisp/pdf/tipsheets/Heart-Healthy-Eating-Plan.pdf  [2] https://www.mayoclinic.org/diseases-conditions/heart-disease/in-depth/heart-healthy-diet/art-20046702  [3] https://www.mayoclinic.org/diseases-conditions/heart-disease/in-depth/heart-healthy-diet/art-20047702  [4] https://www.eatingwell.com/article/7884046/heart-healthy-diet-plan-for-beginners/  [5] https://www.eatingwell.com/article/289245/7-day-heart-healthy-meal-plan-1200-calories/ |
| **Perplexity Answer 3** | Here are some key tips for how many meals to eat per day on a heart-healthy diet:  - Eat 3 meals per day, with 1-2 healthy snacks in between[1][2][3]. Eating smaller, more frequent meals helps keep your blood sugar stable and prevents overeating at meals.  - Space your meals out evenly throughout the day, such as breakfast, lunch, and dinner[2]. Avoid skipping meals, as this can lead to hunger and unhealthy snacking later.  - If you get hungry between meals, have a healthy snack like fresh fruit, vegetables with hummus, or a small handful of nuts[1][3]. Snacks should be around 150-200 calories.  - Drink plenty of water throughout the day to stay hydrated[2]. Avoid sugary beverages like soda, which provide excess calories and sugar.  - Pay attention to portion sizes at meals. Use a smaller plate to help control portions[1]. Filling half your plate with vegetables is a good rule of thumb for heart-healthy eating.  The key is to eat a balanced diet with a variety of nutrient-dense foods like fruits, vegetables, whole grains, lean proteins and healthy fats[1][2][3]. Eating regular meals and snacks helps keep your energy levels stable and prevents overeating.  Citations:  [1] https://www.nhlbi.nih.gov/health/educational/healthdisp/pdf/tipsheets/Heart-Healthy-Eating-Plan.pdf  [2] https://www.mayoclinic.org/diseases-conditions/heart-disease/in-depth/heart-healthy-diet/art-20046702  [3] https://www.eatingwell.com/article/7884046/heart-healthy-diet-plan-for-beginners/  [4] https://www.eatingwell.com/article/289245/7-day-heart-healthy-meal-plan-1200-calories/  [5] https://www.tasteofhome.com/collection/heart-healthy-meal-plan/ |
| **GPT4o Answer 1** | For a heart-healthy diet, it's generally recommended to focus more on the quality and balance of the meals rather than the exact number of meals per day. However, here are some guidelines that can help:  1. Number of Meals: Most people do well with three balanced meals a day (breakfast, lunch, and dinner) and 1-2 healthy snacks if needed. This helps to maintain stable blood sugar levels and avoid overeating.  2. Meal Composition:  - Fruits and Vegetables: Aim for at least five servings of fruits and vegetables a day. These are high in fiber, vitamins, and antioxidants.  - Whole Grains: Choose whole grains over refined grains. Whole grains like oats, brown rice, and whole wheat bread are better for heart health.  - Lean Proteins: Include sources of lean protein such as fish, poultry, beans, and legumes. Fish, particularly fatty fish like salmon, is rich in omega-3 fatty acids, which are good for the heart.  - Healthy Fats: Use healthy fats like olive oil, avocado, nuts, and seeds. Limit saturated fats and avoid trans fats.  - Low-fat Dairy: If you consume dairy, opt for low-fat or fat-free versions.  - Limit Salt and Sugar: Reduce the intake of salt and added sugars. High salt intake is linked to high blood pressure, a risk factor for heart disease.  3. Portion Control: Pay attention to portion sizes to avoid overeating. Eating larger portions can lead to weight gain, which increases the risk of heart disease.  4. Hydration: Drink plenty of water throughout the day. Limit sugary beverages and alcohol.  5. Regular Eating Schedule: Try to eat at regular intervals to prevent extreme hunger, which can lead to overeating. Skipping meals, particularly breakfast, can lead to unhealthy choices later in the day.  Overall, focusing on a balanced diet with nutrient-dense foods and maintaining a regular eating pattern will contribute significantly to heart health. Consulting with a healthcare provider or a nutritionist can provide personalized recommendations based on individual health needs |
| **GPT4o Answer 2** | For a heart-healthy diet, it's not just about the number of meals you eat per day but also the quality and composition of those meals. However, here are some general guidelines:  1. Regular Meal Patterns: Eating three balanced meals per day (breakfast, lunch, and dinner) with healthy snacks in between can help regulate blood sugar levels and prevent overeating.  2. Portion Control: Focus on portion control to avoid excessive calorie intake. Eating smaller, more frequent meals can help manage hunger and prevent large spikes in blood sugar levels.  3. Nutrient-Rich Foods:  - Fruits and Vegetables: Aim for at least 5 servings of fruits and vegetables per day. They are high in vitamins, minerals, and fiber.  - Whole Grains: Choose whole grains over refined grains. Whole grains include brown rice, oats, whole wheat, and quinoa.  - Lean Proteins: Include sources of lean protein such as fish, skinless poultry, beans, and legumes. Fish rich in omega-3 fatty acids, such as salmon and mackerel, are particularly beneficial.  - Healthy Fats: Opt for healthy fats found in nuts, seeds, avocados, and olive oil. Avoid trans fats and limit saturated fats.  4. Limit Sodium and Added Sugars: Reduce your intake of sodium and added sugars, which are linked to hypertension and heart disease.  5. Hydration: Drink plenty of water and avoid sugary drinks. Green tea and herbal teas can be good alternatives.  6. Avoid Processed Foods: Minimize consumption of processed and junk foods, which often contain unhealthy fats, sugars, and salt.  In summary, while three balanced meals a day is a good starting point, the focus should be on the quality of the food you eat. Incorporating nutrient-dense foods and maintaining a balanced diet with controlled portions is key to heart health. If you have specific health concerns or dietary needs, consulting with a healthcare provider or a registered dietitian can provide personalized advice. |
| **GPT4o Answer 3** | For a heart-healthy diet, the focus should be more on the quality and composition of the meals rather than the exact number of meals per day. However, general guidelines recommend:  Frequency of Meals  1. Three Balanced Meals: Eating three well-balanced meals (breakfast, lunch, and dinner) a day is a common recommendation. This can help maintain consistent energy levels and prevent overeating.  2. Healthy Snacks: Including one or two healthy snacks can be beneficial, especially if there are long gaps between meals. This can help manage hunger and prevent unhealthy eating  Key Components of a Heart-Healthy Diet  1. Fruits and Vegetables: Aim for at least 5 servings of fruits and vegetables per day. They are rich in vitamins, minerals, and fiber, and low in calories.  2. Whole Grains: Choose whole grains over refined grains. Options include whole wheat, brown rice, oats, quinoa, and barley.  3. Lean Proteins: Incorporate lean protein sources such as fish, skinless poultry, beans, legumes, and tofu. Fatty fish like salmon and mackerel are excellent for heart health due to their omega-3 fatty acids.  4. Healthy Fats: Use healthy fats like olive oil, avocado, nuts, and seeds. Limit saturated and trans fats.  5. Low-Fat Dairy: Choose low-fat or fat-free dairy products to reduce saturated fat intake.  6. Limit Sodium and Sugar: Reduce the intake of sodium (salt) and added sugars. Use herbs and spices to flavor foods instead of salt.  Meal Timing  - Breakfast: Start your day with a nutritious breakfast that includes whole grains, fruits, and lean proteins.  - Lunch and Dinner: Keep these meals balanced with a variety of vegetables, lean proteins, and whole grains.  - Snacks: Opt for heart-healthy snacks like a piece of fruit, a handful of nuts, or yogurt.  Additional Tips  - Portion Control: Pay attention to portion sizes to avoid overeating.  - Stay Hydrated: Drink plenty of water throughout the day.  - Moderation: Enjoy treats and indulgences in moderation rather than as regular parts of your diet.  Ultimately, the best meal frequency and diet plan will vary from person to person based on individual health needs, lifestyle, and preferences. Consulting with a healthcare provider or a registered dietitian can provide personalized guidance tailored to your specific situation. |
| **Llama 3 Answer 1** | For a heart-healthy diet, the American Heart Association (AHA) recommends eating a balanced diet that focuses on whole, unprocessed foods, and includes a variety of meals and snacks throughout the day. There is no one-size-fits-all answer to the number of meals you should eat, as it depends on individual factors such as age, gender, activity level, and overall health goals. However, here are some general guidelines:  1. Eat 3 main meals a day: Aim for three main meals, spaced evenly apart, to help regulate your appetite and energy levels. These meals should include a balance of protein, healthy fats, and complex carbohydrates.  2. Incorporate 2-3 snacks: In addition to your main meals, include 2-3 snacks in between to help manage hunger and prevent overeating. Choose nutrient-dense snacks like fruits, nuts, veggies, and whole grain crackers with avocado or peanut butter.  3. Listen to your body: Pay attention to your hunger and fullness cues. If you're hungry, eat. If you're not hungry, don't force yourself to eat. This will help you maintain a healthy weight and reduce the risk of heart disease.  4. Consider your lifestyle: If you have a busy schedule or engage in regular physical activity, you may need to eat more frequently to maintain energy levels. On the other hand, if you're less active, you may not need as many meals.  5. Focus on quality over quantity: Prioritize whole, unprocessed foods like fruits, vegetables, whole grains, lean proteins, and healthy fats. Limit your intake of added sugars, saturated fats, and refined carbohydrates.  Here's an example of what a heart-healthy eating pattern might look like:  * Breakfast: Oatmeal with fruit and nuts  * Mid-morning snack: Carrot sticks with hummus  * Lunch: Grilled chicken breast with quinoa and roasted vegetables  * Mid-afternoon snack: Apple slices with almond butter  * Dinner: Baked salmon with brown rice and steamed broccoli  * Evening snack (optional): Greek yogurt with berries and honey  Remember, the key to a heart-healthy diet is to focus on nutrient-dense foods, portion control, and mindful eating habits. Consult with a healthcare professional or a registered dietitian to create a personalized eating plan that meets your unique needs and health goals. |
| **Llama 3 Answer 2** | For a heart healthy diet, the American Heart Association (AHA) recommends eating a balanced diet that includes a variety of nutrient-dense foods from all food groups. The number of meals you should eat per day can vary depending on your individual needs and lifestyle, but here are some general guidelines:  1. Three main meals a day: Aim for three main meals per day, spaced evenly apart, to help control hunger and portion sizes. This can include breakfast, lunch, and dinner.  2. Incorporate healthy snacks: In addition to your three main meals, you can include one or two healthy snacks per day to help manage hunger and maintain energy levels. Choose snacks that are nutrient-dense, such as fruits, nuts, carrot sticks with hummus, or a small handful of trail mix.  3. Listen to your body: Pay attention to your hunger and fullness cues. If you're feeling hungry between meals, it's okay to have a healthy snack. But if you're not hungry, don't force yourself to eat.  4. Avoid excessive eating: Eating too frequently or in excess can lead to consuming more calories, sugar, salt, and unhealthy fats, which can increase your risk of heart disease.  Here are some tips to keep in mind:  * Eat a balanced breakfast to help jumpstart your day and prevent overeating later on.  * Choose whole, unprocessed foods like fruits, vegetables, whole grains, lean proteins, and healthy fats.  * Limit your intake of added sugars, saturated and trans fats, and sodium.  * Stay hydrated by drinking plenty of water throughout the day.  Remember, a heart healthy diet is not just about the number of meals you eat, but also the quality of those meals. Focus on making healthy choices and enjoying a variety of nutrient-dense foods. |
| **Llama 3 Answer 3** | For a heart healthy diet, the general recommendation is to eat 3-5 main meals and 2-3 snacks in between, spaced out every 3-4 hours. This can help:  1. Control hunger and portion sizes: Eating smaller, frequent meals can help regulate hunger and prevent overeating, which can lead to weight gain and other heart health issues.  2. Manage blood sugar and insulin levels: Spacing out meals can help maintain stable blood sugar and insulin levels, reducing the risk of developing insulin resistance and type 2 diabetes.  3. Boost metabolism: Eating frequently can help keep your metabolism going, which can aid in weight management and improve overall heart health.  4. Support healthy blood lipid profiles: Aiming for 3-5 main meals and 2-3 snacks can help maintain healthy levels of triglycerides, LDL (bad) cholesterol, and HDL (good) cholesterol.  Here's an example of what a heart healthy meal pattern might look like:  Main Meals (3-5)  1. Breakfast: Oatmeal with fruit and nuts, whole-grain toast with avocado and eggs  2. Lunch: Grilled chicken breast with quinoa, steamed vegetables, and a side salad  3. Dinner: Baked salmon with brown rice, roasted vegetables, and a side of mixed berries  4. (Optional) Late Dinner or Early Evening Snack: Greek yogurt with honey and almonds, or a small serving of whole-grain crackers with hummus  Snacks (2-3)  1. Mid-Morning Snack: Fresh fruit and a handful of nuts  2. Afternoon Snack: Carrot sticks with hummus or a small serving of trail mix  3. (Optional) Late Afternoon Snack: A small serving of whole-grain crackers with peanut butter or a piece of fresh fruit  Remember to focus on whole, unprocessed foods like fruits, vegetables, whole grains, lean proteins, and healthy fats. Limit your intake of added sugars, saturated and trans fats, and sodium.  Consult with a healthcare professional or a registered dietitian to determine the best meal frequency and pattern for your individual needs and health goals. |
| **LLaMA3+RAG model Answer 1** | The optimal number of meals per day for a heart-healthy diet is a topic of ongoing debate, and the answer is not a one-size-fits-all solution. According to the American Heart Association (AHA), there is no specific recommendation for the number of meals per day, but rather an emphasis on the overall calorie intake and distribution throughout the day.  The AHA suggests that eating more frequent, smaller meals can help balance out hunger and calorie intake, which can be beneficial for weight management. This approach can also help individuals learn to limit their intake at each meal and avoid overeating. However, it is essential to note that the total calorie intake remains the primary determinant of body weight, and excess calorie consumption, regardless of the number of meals, will contribute to weight gain.  The AHA also highlights the importance of not skipping meals, particularly breakfast, as those who forego breakfast and consume a large evening meal tend to have higher BMIs. Instead, it is recommended to focus on spreading calorie intake across the course of the day.  In terms of specific meal frequency, some studies suggest that eating 4-6 meals per day, including 2-3 main meals and 2-3 snacks, can be beneficial for weight management and overall health. However, this may vary depending on individual factors such as age, sex, body composition, and physical activity level.  Ultimately, the key to a heart-healthy diet is to focus on making smart food choices, controlling portion sizes, and maintaining a balanced calorie intake. It is also essential to combine a healthy diet with regular physical activity to achieve and maintain a healthy weight.  Sources:  American Heart Association. (2024). Is 3 Meals a Day the Only Way? Retrieved from <https://www.heart.org/en/healthy-living/healthy-eating/eat-smart/nutrition-basics/is-3-meals-a-day-the-only-way> |
| **LLaMA3+RAG model Answer 2** | The optimal number of meals per day for a heart-healthy diet is not a one-size-fits-all answer. According to the American Heart Association, the traditional approach of having three square meals a day may not be the only way to achieve and maintain a healthy weight. In fact, having more frequent, smaller meals throughout the day can help balance out hunger and calorie intake.  Research suggests that eating more frequent, smaller meals can help individuals learn how to limit their intake at each meal and avoid the ravenous appetite that often accompanies skipping meals or going too long without eating. This approach can also help spread out calorie intake across the course of the day, which is beneficial for overall health and weight management.  That being said, it's essential to remember that the total calorie intake ultimately determines body size. Excess calorie intake, regardless of whether it's spread out over several meals or consumed in one sitting, will still contribute to weight gain.  Instead of focusing on the number of meals, it's more important to prioritize spreading out calorie intake across the day and avoiding skipping meals. Skipping meals, particularly breakfast, can lead to higher BMIs, as individuals tend to consume larger evening meals to compensate for the lack of food earlier in the day.  A more effective approach might be to:  1. Set calorie targets: Determine your daily calorie needs and aim to subtract 500 calories per day to lose 1 pound per week.  2. Start a food diary: Track your meals, portions, and times of day to identify patterns and make adjustments.  3. Get moving: Regular exercise is crucial for overall health and can also help with weight management.  In summary, while there is no specific number of meals per day that is universally recommended for a heart-healthy diet, focusing on spreading out calorie intake across the day, avoiding skipping meals, and prioritizing overall calorie balance is a more effective approach to achieving and maintaining a healthy weight.  Sources:  American Heart Association. (2024). Is 3 Meals a Day the Only Way? Retrieved from <https://www.heart.org/en/healthy-living/healthy-eating/eat-smart/nutrition-basics/is-3-meals-a-day-the-only-way> |
| **LLaMA3+RAG model Answer 3** | The ideal number of meals per day for a heart-healthy diet is not strictly defined, but rather, it's about finding a balance that works for you. The American Heart Association suggests that having more frequent, smaller meals throughout the day can help balance out hunger and calories, rather than sticking to the traditional three square meals a day.  According to Dr. Linda Van Horn, a registered dietitian and professor at Northwestern University, eating more frequent, smaller meals can help individuals learn to limit their intake at each meal and avoid the ravenous appetite that comes with skipping meals or going too long without eating. This approach can also help spread out calorie intake across the course of the day, which is beneficial for weight management.  It's essential to note that the total calorie intake is what ultimately determines body size, and excess calorie intake, regardless of the frequency of meals, will contribute to weight gain. Therefore, it's crucial to set calorie targets, track food intake, and make smart choices when dining out or cooking at home.  The American Heart Association recommends starting with small steps, such as setting calorie targets, keeping a food diary, and getting regular exercise. By doing so, individuals can develop healthy eating habits and maintain a healthy weight.  In summary, while there is no one-size-fits-all answer to the question of how many meals a day to eat for a heart-healthy diet, the key takeaways are:  Aim for more frequent, smaller meals to balance out hunger and calories  Spread out calorie intake across the course of the day  Set calorie targets and track food intake  Make smart choices when dining out or cooking at home  Get regular exercise for overall health and weight management  Sources:  American Heart Association. (2024). Is 3 Meals a Day the Only Way? Retrieved from <https://www.heart.org/en/healthy-living/healthy-eating/eat-smart/nutrition-basics/is-3-meals-a-day-the-only-way> |
| **Question 13** | **What counts as a serving of fruits and vegetables?** |
| **Perplexity Answer 1** | A serving of fruits and vegetables can vary slightly depending on the source, but generally:  For fruits:  - 1 medium-sized whole fruit (like an apple, banana, orange, etc.) [1][3]  - 1/2 cup fresh, frozen, or canned fruit [1][3]  - 1/4 cup dried fruit [1][3]  - 1/4 cup 100% fruit juice [1][3]  For vegetables:  - 1 cup raw leafy greens (like spinach, lettuce, etc.) [1][3]  - 1/2 cup fresh, frozen, or canned vegetables [1][3]  - 1/2 cup 100% vegetable juice [3]  - 1/2 cup cooked beans or peas [5]  Some specific examples:  - 1 medium pear, peach, or orange [1]  - 16 grapes or 7 strawberries [1]  - 5-8 broccoli or cauliflower florets [1]  - 1/2 medium potato or sweet potato [1]  - 3 heaped tablespoons of cooked carrots, peas, or corn [4]  The recommended daily target is 4-5 servings of fruits and 4-5 servings of vegetables for a 2,000 calorie diet.[1][3][5] Serving sizes are based on providing proper nutrition while limiting calories, sugar, and sodium intake.[3]  Citations:  [1] https://hamptonmarket.com/what-to-know-about-fruit-and-vegetable-serving-sizes/  [2] https://www.cnet.com/health/nutrition/how-to-get-your-daily-servings-of-vegetables-and-fruit-a-visual-guide/  [3] https://www.heart.org/en/healthy-living/healthy-eating/add-color/fruits-and-vegetables-serving-sizes  [4] https://www.nhs.uk/live-well/eat-well/5-a-day/portion-sizes/  [5] https://www.mayoclinichealthsystem.org/hometown-health/speaking-of-health/123-approach-to-eating-fruits-and-vegetables |
| **Perplexity Answer 2** | A serving of fruits and vegetables can vary slightly depending on the source, but generally:  For fruits:  - 1 medium-sized whole fruit (like an apple, banana, orange, etc.) [1][3]  - 1/2 cup fresh, frozen, or canned fruit [1][3]  - 1/4 cup dried fruit [1][3]  - 1/4 cup 100% fruit juice [1][3]  For vegetables:  - 1 cup raw leafy greens (like spinach, lettuce, etc.) [1][3]  - 1/2 cup fresh, frozen, or canned vegetables [1][3]  - 1/2 cup 100% vegetable juice [3]  - 1/2 cup cooked beans or peas [5]  Some specific examples:  - 1 medium pear, peach, or orange [1]  - 16 grapes or 7 strawberries [1]  - 5-8 broccoli or cauliflower florets [1]  - 1/2 medium potato or sweet potato [1]  - 3 heaped tablespoons of cooked carrots, peas, or corn [4]  The recommended daily target is 4-5 servings of fruits and 4-5 servings of vegetables for a 2,000 calorie diet.[1][3][5] Serving sizes are based on a visual guide to make it easier to estimate proper portions.[2][4]  Citations:  [1] https://hamptonmarket.com/what-to-know-about-fruit-and-vegetable-serving-sizes/  [2] https://www.cnet.com/health/nutrition/how-to-get-your-daily-servings-of-vegetables-and-fruit-a-visual-guide/  [3] https://www.heart.org/en/healthy-living/healthy-eating/add-color/fruits-and-vegetables-serving-sizes  [4] https://www.nhs.uk/live-well/eat-well/5-a-day/portion-sizes/  [5] https://www.mayoclinichealthsystem.org/hometown-health/speaking-of-health/123-approach-to-eating-fruits-and-vegetables |
| **Perplexity Answer 3** | A serving of fruits and vegetables can vary slightly depending on the source, but generally:  For fruits:  - 1 medium-sized whole fruit (like an apple, banana, orange, etc.) [1][3]  - 1/2 cup fresh, frozen, or canned fruit [1][3]  - 1/4 cup dried fruit [1][3]  - 1/4 cup 100% fruit juice [1][3]  For vegetables:  - 1 cup raw leafy greens (like spinach, lettuce, etc.) [1][3]  - 1/2 cup fresh, frozen, or canned vegetables [1][3]  - 1/2 cup 100% vegetable juice [3]  - 1/2 cup cooked beans or peas [5]  Some specific examples of one serving:  - 1 medium apple or banana [1][3]  - 16 grapes or 7 strawberries [1]  - 1/2 cup broccoli or carrots [1][5]  - 1 small baked potato [5]  - 3 cups raw spinach [5]  The general recommended amount is 4-5 servings of fruits and vegetables per day for a 2,000 calorie diet.[1][3][4] Serving sizes can vary slightly by source, but the above guidelines provide a good reference for what counts as one serving.[1][3][5]  Citations:  [1] https://hamptonmarket.com/what-to-know-about-fruit-and-vegetable-serving-sizes/  [2] https://www.cnet.com/health/nutrition/how-to-get-your-daily-servings-of-vegetables-and-fruit-a-visual-guide/  [3] https://www.heart.org/en/healthy-living/healthy-eating/add-color/fruits-and-vegetables-serving-sizes  [4] https://www.nhs.uk/live-well/eat-well/5-a-day/portion-sizes/  [5] https://www.mayoclinichealthsystem.org/hometown-health/speaking-of-health/123-approach-to-eating-fruits-and-vegetables |
| **GPT4o Answer 1** | A serving of fruits and vegetables can vary based on the type of produce, but general guidelines can help you understand typical serving sizes:  Fruits  1. Fresh Fruit:  - 1 medium-sized fruit (e.g., apple, banana, orange)  - ½ cup of chopped, cooked, or canned fruit  - ¼ cup of dried fruit  - 1 cup of 100% fruit juice (though juice is often less preferred due to lower fiber content)  2. Berries and Smaller Fruits:  - 1 cup of berries (e.g., strawberries, blueberries, raspberries)  - 2 small fruits (e.g., kiwi, apricots, plums)  3. Melons and Pineapples:  - 1 cup of diced melon or pineapple  Vegetables  1. Raw Leafy Vegetables:  - 2 cups of raw leafy greens (e.g., spinach, lettuce, kale)  2. Cooked or Raw Non-leafy Vegetables:  - 1 cup of cooked or raw vegetables (e.g., broccoli, carrots, bell peppers)  - 1 cup of vegetable juice  3. Starchy Vegetables:  - 1 cup of cooked starchy vegetables (e.g., potatoes, corn, peas)  Mixed Dishes and General Guidelines  - When vegetables are part of a mixed dish (e.g., stew, casserole), a serving is typically 1 cup of the mixed dish, which will include about ½ cup of vegetables.  - A serving of salad is typically considered 2 cups, as it often includes a mix of various vegetables.  General Considerations  - Fiber and Nutrient Content: Whole fruits and vegetables are often better than juices, as they contain more fiber and nutrients.  - Variety: Aim for a variety of colors and types to ensure a broad intake of vitamins, minerals, and antioxidants.  These guidelines can help ensure you meet the daily recommended intake of fruits and vegetables for a balanced and nutritious diet. |
| **GPT4o Answer 2** | The definition of a serving of fruits and vegetables can vary depending on the type of produce and the guidelines you are following. Here are some general guidelines based on common recommendations:  Fruits  - Fresh, frozen, or canned fruit: 1 medium-sized fruit (such as an apple, banana, or orange), 1/2 cup of chopped, cooked, or canned fruit (in 100% juice), or 1/4 cup of dried fruit.  - Fruit juice: 1/2 cup (4 ounces) of 100% fruit juice.  Vegetables  - Raw leafy greens: 1 cup of raw leafy greens (such as spinach, lettuce, or kale) counts as one serving.  - Fresh, frozen, or canned vegetables: 1/2 cup of chopped, cooked, or canned vegetables.  - Vegetable juice: 1/2 cup (4 ounces) of 100% vegetable juice.  - Starchy vegetables: 1/2 cup of cooked starchy vegetables (such as potatoes, corn, or peas).  - Legumes: 1/2 cup of cooked beans, peas, or lentils.  Examples of Serving Sizes  Fruits  - 1 small apple  - 1 large banana  - 32 seedless grapes  - 1 medium grapefruit  - 1 large orange  - 1 small pear  - 1 small watermelon wedge  - 1/2 cup of fresh strawberries  Vegetables  - 1 cup of raw spinach  - 1 large bell pepper  - 1/2 cup of chopped carrots  - 1 cup of raw broccoli  - 1/2 cup of cooked green beans  - 1/2 cup of cooked peas  - 1 large tomato  - 1/2 cup of sliced cucumbers  General Recommendations  The USDA MyPlate guidelines suggest filling half your plate with fruits and vegetables at each meal. The specific number of servings recommended can vary based on age, sex, and level of physical activity, but a general goal is to consume at least 5 servings of fruits and vegetables per day.  It's also important to aim for a variety of fruits and vegetables to ensure you're getting a broad range of nutrients. |
| **GPT4o Answer 3** | A serving of fruits and vegetables can vary depending on the type of fruit or vegetable and the guidelines provided by different health organizations. Here are general guidelines for what counts as a serving:  Fruits:  1. Fresh Fruit:  - 1 medium piece of fruit (e.g., apple, banana, orange).  - ½ cup of chopped, cooked, or canned fruit.  - ¼ cup of dried fruit.  - ¾ cup of 100% fruit juice.  2. Berries and Smaller Fruits:  - 1 cup of berries or grapes.  - ½ large grapefruit or avocado.  Vegetables:  1. Leafy Greens:  - 1 cup of raw leafy vegetables (e.g., spinach, lettuce).  - ½ cup of cooked leafy greens.  2. Other Vegetables:  - ½ cup of chopped, cooked, or canned vegetables.  - 1 medium potato (about the size of a computer mouse).  3. Juice:  - ¾ cup of 100% vegetable juice.  Examples:  - Carrots: ½ cup raw or cooked.  - Tomatoes: 1 medium tomato or ½ cup cooked/canned.  - Broccoli: ½ cup cooked or raw.  - Peas: ½ cup cooked.  General Tips:  - Variety: Aim for a variety of colors and types to ensure a range of nutrients.  - Fresh, Frozen, or Canned: All forms count, but watch for added sugars or sodium in canned or processed versions.  - Daily Goals: Most guidelines recommend at least 5 servings of fruits and vegetables per day.  Portion Control:  - Using a standard measuring cup for portions can help.  - Visual cues like comparing a serving size to a common object (e.g., a baseball for a medium apple) can be useful.  These guidelines help ensure that you're getting enough fruits and vegetables in your diet for optimal health. |
| **Llama 3 Answer 1** | A serving of fruits and vegetables is a standardized amount that helps you measure your daily intake. Here are the general guidelines:  Fruits:  * 1 serving of fruit is:  + 1 medium-sized fruit (e.g., apple, banana, orange)  + 1/2 cup fresh, frozen, or canned fruit  + 4 ounces (1/2 cup) 100% fruit juice  + 1/4 cup dried fruit (e.g., raisins, apricots)  Examples of fruits and their serving sizes:  * Apple: 1 medium (about 5 ounces)  * Berries: 1/2 cup fresh or frozen  * Banana: 1 medium (about 6 ounces)  * Orange: 1 medium (about 5 ounces)  Vegetables:  * 1 serving of vegetables is:  + 1 cup raw or 1/2 cup cooked leafy greens (e.g., spinach, kale)  + 1 cup raw or 1/2 cup cooked non-leafy vegetables (e.g., broccoli, carrots)  + 1/2 cup cooked legumes (e.g., beans, lentils)  + 1/2 cup 100% vegetable juice  Examples of vegetables and their serving sizes:  * Carrots: 1 cup raw or 1/2 cup cooked  * Broccoli: 1 cup raw or 1/2 cup cooked  * Spinach: 1 cup fresh or 1/2 cup cooked  * Tomatoes: 1 cup cherry tomatoes or 1 medium tomato  Tips:  * Aim for a variety of colors on your plate to ensure you're getting a range of nutrients.  * Fresh, frozen, canned, and dried options all count towards your daily servings.  * Cooking and preparing fruits and vegetables can affect their serving sizes, so adjust accordingly.  * Pay attention to portion sizes and control the amount you eat to maintain a healthy diet.  Remember, the goal is to consume a minimum of 5 servings of fruits and vegetables daily, with 2.5 servings being vegetables. Enjoy your fruits and veggies! |
| **Llama 3 Answer 2** | A serving of fruits and vegetables is a standardized amount that helps you measure your daily intake. Here are the serving sizes and examples from the United States Department of Agriculture (USDA):  Fruits:  * 1 medium-sized fruit (e.g., apple, banana, orange)  * 1/2 cup fresh, frozen, or canned fruit (e.g., berries, sliced peaches)  * 4 ounces (1/2 cup) 100% fruit juice  * 1/4 cup dried fruit (e.g., raisins, apricots)  Examples of single servings:  * 1 small apple (about the size of a tennis ball)  * 1/2 cup grapes  * 1/2 cup sliced strawberries  * 4 ounces (1/2 cup) orange juice  Vegetables:  * 1 cup raw or 1/2 cup cooked leafy greens (e.g., spinach, kale)  * 1/2 cup cooked or 1 cup raw other vegetables (e.g., broccoli, carrots)  * 4 ounces (1/2 cup) 100% vegetable juice  Examples of single servings:  * 1 cup raw spinach leaves  * 1/2 cup cooked broccoli florets  * 1/2 cup sliced bell peppers  * 4 ounces (1/2 cup) tomato juice  Additional Tips:  * Fresh, frozen, canned, and dried fruits and vegetables all count towards your daily intake.  * Try to include a variety of colors on your plate to ensure you're getting a range of nutrients.  * Aim for 2-3 servings of fruits and 3-5 servings of vegetables per day.  * If you're not sure about serving sizes, use a measuring cup or the palm of your hand as a rough guide (1 serving is about the size of your palm).  Remember, the key is to aim for a balanced diet with a variety of fruits and vegetables to support your overall health and well-being! |
| **Llama 3 Answer 3** | A serving of fruits and vegetables is a standardized amount that is used to help guide daily intake recommendations. Here are the general guidelines:  Fruits:  * 1 serving of fruit is:  + 1 medium-sized fruit (e.g., apple, banana, orange)  + 1/2 cup fresh, frozen, or canned fruit  + 4 ounces (1/2 cup) 100% fruit juice  + 1/4 cup dried fruit  * Examples of 1 serving of fruit:  + 1 small apple (about the size of a tennis ball)  + 1/2 cup grapes  + 1/2 cup mixed berries  + 4 ounces (1/2 cup) orange juice  Vegetables:  * 1 serving of vegetables is:  + 1 cup raw or 1/2 cup cooked leafy greens (e.g., spinach, kale)  + 1/2 cup cooked or 1 cup raw non-leafy vegetables (e.g., broccoli, carrots)  + 1/2 cup 100% vegetable juice  + 1/4 cup cooked legumes (e.g., beans, lentils)  * Examples of 1 serving of vegetables:  + 1 cup raw spinach leaves  + 1/2 cup cooked broccoli florets  + 1/2 cup sliced carrots  + 1/2 cup cooked black beans  Special Cases:  * Tomatoes: 1 medium tomato or 1 cup cherry tomatoes count as 1 serving  * Potatoes: 1 medium-sized potato (about the size of a tennis ball) counts as 1 serving  * Corn: 1 cup cooked or 1 ear of corn counts as 1 serving  Daily Recommendations:  * The World Health Organization recommends a minimum of 400g (5 portions) of fruits and vegetables per day, with a variety of types and colors.  * The United States Department of Agriculture (USDA) recommends 2-3 cups of vegetables and 1.5-2 cups of fruits per day for adults.  Remember, these are general guidelines, and individual serving sizes may vary based on age, sex, body size, and physical activity level. It's always a good idea to consult with a healthcare professional or registered dietitian for personalized nutrition recommendations. |
| **LLaMA3+RAG model Answer 1** | According to the American Heart Association, a serving size of fruits and vegetables varies depending on the type and preparation method. Here's a breakdown of what counts as a serving:  Fruits:  One medium-sized fruit (e.g., apple, banana, orange) is equivalent to one serving.  Fresh, frozen, or canned fruit: ½ cup is considered one serving.  Dried fruit: ¼ cup is considered one serving.  Fruit juice: ¼ cup of 100% fruit juice (without added sugars or sodium) counts as one serving. However, it's essential to be mindful of the sugar content and opt for 100% juice or a mix of 100% juice and water instead of sweetened juice or juice drinks.  Vegetables:  Raw leafy vegetables: 1 cup is considered one serving.  Fresh, frozen, or canned vegetables: ½ cup is considered one serving.  Vegetable juice: ½ cup of 100% vegetable juice (without added sugars or sodium) counts as one serving.  It's recommended to aim for 4-5 servings of fruits and 4-5 servings of vegetables per day, based on a 2,000 calorie/day eating pattern.  Sources:  American Heart Association. (2024). Fruits and Vegetables Serving Sizes. Retrieved from <https://www.heart.org/en/healthy-living/healthy-eating/add-color/fruits-and-vegetables-serving-sizes:~:text=One%20serving%20of%20100%25%20juice,sweetened%20juice%20or%20juice%20drinks.> |
| **LLaMA3+RAG model Answer 2** | According to the American Heart Association, a serving of fruits and vegetables can vary depending on the type and preparation method. Here's a breakdown of what counts as a serving for each:  Fruits:  One medium-sized fruit (e.g., apple, banana, orange)  ½ cup of fresh, frozen, or canned fruit  ¼ cup of dried fruit  ¼ cup of fruit juice (100% juice, not sweetened juice or juice drinks)  Vegetables:  1 cup of raw leafy vegetables (e.g., spinach, kale, lettuce)  ½ cup of fresh, frozen, or canned vegetables  ½ cup of vegetable juice (100% juice, not sweetened juice or juice drinks)  It's essential to note that these serving sizes are based on a 2,000 calorie/day eating pattern, and the recommended daily goal is to consume 4-5 servings of each per day.  Remember, when it comes to fruit and vegetable juices, it's crucial to choose 100% juice without added sugars or sodium. You can also opt for 100% juice and water as a healthier alternative.  Sources:  American Heart Association. (2024). Fruits and Vegetables Serving Sizes. Retrieved from <https://www.heart.org/en/healthy-living/healthy-eating/add-color/fruits-and-vegetables-serving-sizes:~:text=One%20serving%20of%20100%25%20juice,sweetened%20juice%20or%20juice%20drinks>  American Heart Association. (2024). Healthy Eating Plate. Retrieved from <https://www.heart.org/en/healthy-living/healthy-eating/eat-smart/nutrition-basics/aha-diet-and-lifestyle-recommendations |
| **LLaMA3+RAG model Answer 3** | According to the American Heart Association, a serving of fruits and vegetables can be measured in various ways depending on the type and form of the fruit or vegetable. Here's a breakdown of what counts as a serving:  Fruits:  One medium-sized fruit (e.g., apple, banana, orange)  ½ cup of fresh, frozen, or canned fruit  ¼ cup of dried fruit  ¼ cup of 100% fruit juice (note: watch out for added sugars or sodium)  Vegetables:  1 cup of raw leafy vegetables (e.g., spinach, kale, lettuce)  ½ cup of fresh, frozen, or canned vegetables  ½ cup of vegetable juice (again, be mindful of added sugars or sodium)  It's essential to aim for 4-5 servings of each per day, based on a 2,000 calorie/day eating pattern. Remember, 100% fruit and vegetable juices can be part of a healthy dietary pattern, but it's crucial to choose unsweetened and low-sodium options.  Sources:  American Heart Association. (2024). Fruits and Vegetables Serving Sizes. Retrieved from <https://www.heart.org/en/healthy-living/healthy-eating/add-color/fruits-and-vegetables-serving-sizes:~:text=One%20serving%20of%20100%25%20juice,sweetened%20juice%20or%20juice%20drinks.> |
| **Question 14** | **Can I eat pizza on a heart healthy diet?** |
| **Perplexity Answer 1** | Yes, you can still enjoy pizza on a heart-healthy diet with some modifications. Here are some tips for making pizza more heart-friendly:  Choose Whole-Grain Crust  Opt for a whole-grain or thin crust to increase fiber intake and reduce refined carbs[1]. Avoid crusts made with hydrogenated oils.  Use Healthy Sauces  Go for fresh tomato sauce or no-salt-added canned tomato sauce, which are high in fiber, vitamins, and antioxidants like lycopene[1][3]. Avoid cream-based or sugary sauces. You can also try pesto or olive oil with garlic for healthy fats.  Go Light on Cheese  Cheese is high in saturated fat and sodium, so use it sparingly[1]. Choose low-fat options like part-skim mozzarella or feta. You can also try dairy-free cheese alternatives.  Load Up on Veggies  Pile on nutrient-rich veggie toppings like spinach, bell peppers, mushrooms, onions, and tomatoes for fiber, vitamins, and antioxidants[1].  Choose Lean Proteins  Avoid processed meats like pepperoni and sausage, which are high in saturated fat and sodium[1][3]. Instead, opt for lean proteins like grilled chicken, shrimp, or plant-based toppings like chickpeas or lentils.  Watch Portion Sizes  Stick to one or two slices of pizza with a side salad or veggies to control calories and sodium intake[2]. Overeating pizza can lead to weight gain and increased cholesterol levels.  By making these adjustments, you can enjoy pizza while still following a heart-healthy eating pattern[1][2][3]. Moderation and balance are key.  Citations:  [1] https://health.clevelandclinic.org/how-to-make-healthy-pizza  [2] https://www.eatingwell.com/article/8055628/is-pizza-healthy/  [3] https://www.healthline.com/nutrition/pizza-cholesterol  [4] https://timesofindia.indiatimes.com/life-style/health-fitness/diet/heres-what-happens-to-your-body-if-you-eat-pizza-every-week/photostory/100252263.cms  [5] https://www.webmd.com/heart-disease/ss/slideshow-foods-bad-heart |
| **Perplexity Answer 2** | Yes, you can eat pizza on a heart-healthy diet by making some simple modifications:  Crust  - Choose a whole-grain or thin crust to reduce refined carbs and increase fiber[9].  - Look for crusts without hydrogenated oils[9].  Sauce  - Use tomato-based sauces which are lower in fat and provide vitamins[9].  - Avoid cream-based sauces like Alfredo which are high in saturated fat[9].  Toppings  - Load up on vegetable toppings like mushrooms, peppers, onions, etc. for added nutrients[9].  - Use lean protein sources like grilled chicken or turkey pepperoni instead of high-fat meats[9].  - Go light on the cheese or use a low-fat variety[9].  Other Tips  - Make your own pizza at home so you can control the ingredients[9].  - When ordering out, choose thin-crust veggie pizzas and avoid stuffed crusts or meat-loaded toppings[9].  By choosing whole-grain crusts, tomato sauces, plenty of veggies, and lean proteins, pizza can absolutely fit into a heart-healthy diet in moderation[9]. The key is being mindful of portion sizes and making smart topping choices.  Citations:  [1] https://www.yelp.com/biz/pizzaiolo-oakland?adjust_creative=6zagVcKwffYZAA-51mlGNg  [2] https://www.yelp.com/biz/cholita-linda-oakland?adjust_creative=6zagVcKwffYZAA-51mlGNg  [3] https://www.yelp.com/biz/fourn%C3%A9e-bakery-berkeley?adjust_creative=6zagVcKwffYZAA-51mlGNg  [4] https://www.yelp.com/biz/nicks-pizza-oakland?adjust_creative=6zagVcKwffYZAA-51mlGNg  [5] https://www.yelp.com/biz/mariposa-baking-company-oakland?adjust_creative=6zagVcKwffYZAA-51mlGNg  [6] https://www.yelp.com/biz/casa-barotti-berkeley?adjust_creative=6zagVcKwffYZAA-51mlGNg  [7] https://www.yelp.com/biz/beautys-bagel-shop-oakland?adjust_creative=6zagVcKwffYZAA-51mlGNg  [8] https://www.yelp.com/biz/pizzeria-da-laura-berkeley?adjust_creative=6zagVcKwffYZAA-51mlGNg  [9] https://health.clevelandclinic.org/how-to-make-healthy-pizza  [10] https://www.eatingwell.com/article/8055628/is-pizza-healthy/  [11] https://www.webmd.com/heart-disease/ss/slideshow-foods-bad-heart  [12] https://www.healthline.com/nutrition/pizza-cholesterol  [13] https://www.mayoclinic.org/diseases-conditions/heart-disease/in-depth/heart-healthy-diet/art-20046702 |
| **Perplexity Answer 3** | Yes, you can eat pizza on a heart-healthy diet by making some simple modifications:  Crust  - Choose a whole-grain or thin crust to reduce refined carbs and increase fiber[9]  - Look for crusts without hydrogenated oils[9]  Sauce  - Use tomato-based sauces which are lower in fat and provide vitamins[9]  - Avoid cream-based sauces like Alfredo which are high in saturated fat[9]  Toppings  - Load up on vegetable toppings like mushrooms, peppers, onions, etc. for nutrients and fiber[9]  - Use lean protein toppings like grilled chicken or Canadian bacon instead of high-fat meats[9]  - Go light on the cheese or use a low-fat variety[9]  Other Tips  - Make your own pizza at home so you can control the ingredients[9]  - When ordering out, choose thin crust and ask for half the cheese[9]  - Add healthy fats like avocado or olive oil instead of fatty meats[9]  By following these tips, you can make pizza a part of a balanced, heart-healthy diet by increasing nutrients like fiber, vitamins and healthy fats while reducing saturated fat, sodium and refined carbs[9]. Moderation is key, but pizza can be enjoyed on a heart-healthy eating plan.  Citations:  [1] https://www.yelp.com/biz/pizzaiolo-oakland?adjust_creative=6zagVcKwffYZAA-51mlGNg  [2] https://www.yelp.com/biz/cholita-linda-oakland?adjust_creative=6zagVcKwffYZAA-51mlGNg  [3] https://www.yelp.com/biz/fourn%C3%A9e-bakery-berkeley?adjust_creative=6zagVcKwffYZAA-51mlGNg  [4] https://www.yelp.com/biz/nicks-pizza-oakland?adjust_creative=6zagVcKwffYZAA-51mlGNg  [5] https://www.yelp.com/biz/mariposa-baking-company-oakland?adjust_creative=6zagVcKwffYZAA-51mlGNg  [6] https://www.yelp.com/biz/casa-barotti-berkeley?adjust_creative=6zagVcKwffYZAA-51mlGNg  [7] https://www.yelp.com/biz/beautys-bagel-shop-oakland?adjust_creative=6zagVcKwffYZAA-51mlGNg  [8] https://www.yelp.com/biz/pizzeria-da-laura-berkeley?adjust_creative=6zagVcKwffYZAA-51mlGNg  [9] https://health.clevelandclinic.org/how-to-make-healthy-pizza  [10] https://www.eatingwell.com/article/8055628/is-pizza-healthy/  [11] https://www.webmd.com/heart-disease/ss/slideshow-foods-bad-heart  [12] https://www.healthline.com/nutrition/pizza-cholesterol  [13] https://blog.mercy.com/worst-foods-for-your-heart/ |
| **GPT4o Answer 1** | Yes, you can eat pizza on a heart-healthy diet, but with some modifications to make it more nutritious and aligned with heart-healthy guidelines. Here are some tips:  1. Choose Whole Grain Crust: Opt for a whole wheat or whole grain crust instead of the traditional white crust. Whole grains provide more fiber, which is beneficial for heart health.  2. Load Up on Veggies: Increase the amount of vegetables on your pizza. Vegetables like bell peppers, tomatoes, spinach, mushrooms, and onions add nutrients and fiber without adding unhealthy fats.  3. Limit Cheese: Cheese can be high in saturated fat and sodium. Use a moderate amount of low-fat cheese, or try using a strong-flavored cheese in smaller quantities so you need less of it.  4. Add Lean Proteins: Instead of processed meats like pepperoni and sausage, choose lean protein options like grilled chicken, turkey, or plant-based proteins like tofu.  5. Control Portions: Eat smaller portions of pizza and pair it with a large salad or a side of vegetables to make your meal more balanced and filling.  6. Mind the Sauce: Use a tomato sauce that is low in added sugars and sodium. You can even make your own sauce using fresh tomatoes and herbs.  7. Limit Extra Fats: Be cautious with additional toppings like extra cheese, creamy sauces, or high-fat meats. Choose healthier options like olives, artichokes, or fresh herbs.  By making these adjustments, you can enjoy pizza as part of a heart-healthy diet without compromising on flavor. |
| **GPT4o Answer 2** | Yes, you can eat pizza on a heart-healthy diet, but it's important to make mindful choices regarding ingredients and portions. Here are some tips to make pizza more heart-healthy:  Choose a Whole Grain Crust  - Opt for a whole wheat or whole grain crust instead of a refined white flour crust. Whole grains provide more fiber and nutrients, which are beneficial for heart health.  Go Light on the Cheese  - Use less cheese, and choose lower-fat cheese options. Part-skim mozzarella or other reduced-fat cheeses can help reduce saturated fat intake.  Load Up on Vegetables  - Add a variety of vegetables as toppings. Veggies like tomatoes, spinach, bell peppers, onions, mushrooms, and broccoli add flavor, fiber, and nutrients without adding unhealthy fats.  Choose Lean Proteins  - If you want to add protein, opt for lean options like grilled chicken, turkey, or plant-based proteins. Avoid high-fat meats like pepperoni, sausage, and bacon.  Limit Processed Meats  - Processed meats are often high in sodium and unhealthy fats, which can negatively impact heart health. It's best to minimize or avoid them.  Use a Healthy Sauce  - Choose a tomato sauce that is low in added sugars and sodium. You can also make your own sauce to control the ingredients.  Watch Portion Sizes  - Be mindful of portion sizes. Instead of eating multiple slices, enjoy one or two slices with a side salad or other vegetables to keep the meal balanced and satisfying.  Consider Homemade Pizza  - Making pizza at home gives you complete control over the ingredients, allowing you to make healthier choices.  Mind the Extras  - Avoid adding extra salt and use herbs and spices for flavor instead. Herbs like basil, oregano, and garlic can enhance the taste without adding sodium.  By following these guidelines, you can enjoy pizza while maintaining a heart-healthy diet. |
| **GPT4o Answer 3** | Yes, you can eat pizza on a heart-healthy diet, but it's important to make some mindful choices to keep it nutritious and supportive of heart health. Here are some tips to enjoy pizza while maintaining a heart-healthy diet:  1. Choose a Whole-Grain Crust: Whole grains provide more fiber and nutrients compared to refined grains. Look for or make a whole-grain or whole-wheat pizza crust.  2. Limit Cheese: Cheese can be high in saturated fat and sodium. Use a moderate amount of cheese, preferably low-fat or part-skim varieties. Y3u can also try using less cheese and adding flavor with herbs and spices.  3. Pile on the Veggies: Load your pizza with a variety of vegetables. Veggies add fiber, vitamins, and minerals while being low in calories. Options include tomatoes, bell peppers, onions, mushrooms, spinach, and broccoli.  4. Choose Lean Proteins: Instead of high-fat meats like pepperoni or sausage, opt for lean protein sources. Consider grilled chicken, turkey, or plant-based proteins like tofu or legumes.  5. Use a Healthy Sauce: Choose a tomato sauce with no added sugar and low sodium. You can also make your own sauce using fresh tomatoes and herbs.  6. Watch Portion Sizes: Be mindful of how much pizza you eat. Pair your pizza with a side salad to help fill you up and provide additional nutrients without overeating.  7. Limit Processed Ingredients: Avoid highly processed toppings and ingredients that can be high in unhealthy fats, sodium, and additives.  By making these adjustments, you can enjoy pizza as part of a heart-healthy diet without compromising on flavor or satisfaction. |
| **Llama 3 Answer 1** | The eternal question! While traditional pizza can be a nutritional nightmare, it's not necessarily off-limits on a heart-healthy diet. The key is to make some savvy choices and modifications to create a healthier pizza option. Here's how:  Heart-healthy pizza tips:  1. Crust: Opt for a whole-grain crust or a cauliflower crust, which are lower in refined carbohydrates and added fiber.  2. Sauce: Choose a tomato sauce with no added salt or sugar. You can also use a homemade sauce made with canned crushed tomatoes, olive oil, and herbs.  3. Cheese: Use a moderate amount of lower-fat cheese, such as part-skim mozzarella, reduced-fat cheddar, or feta. You can also try dairy-free alternatives like vegan mozzarella.  4. Toppings: Load up on heart-healthy toppings like:  * Vegetables: mushrooms, bell peppers, onions, olives, and tomatoes  * Lean protein: chicken, turkey pepperoni, or plant-based options like vegan sausage  * Herbs and spices: oregano, basil, garlic, and red pepper flakes  5. Portion control: Aim for a slice that's about 1/4 of the pizza (around 3-4 inches in diameter).  6. Limit the frequency: Enjoy pizza in moderation, as it's still a treat. Aim for no more than 1-2 times a week.  Healthier pizza options:  1. Mediterranean pizza: Top with feta cheese, Kalamata olives, artichoke hearts, and sun-dried tomatoes.  2. Veggie lovers: Load up on mushrooms, bell peppers, onions, and tomatoes, with a sprinkle of part-skim mozzarella.  3. Grilled chicken and veggie: Top with grilled chicken, roasted vegetables like zucchini and eggplant, and a drizzle of olive oil.  Remember: Even with healthier modifications, pizza is still a treat and should be consumed in moderation as part of a balanced diet.  So, go ahead and enjoy your heart-healthy pizza – just be mindful of the ingredients and portion sizes! |
| **Llama 3 Answer 2** | The eternal question! While pizza can be a challenging food to fit into a heart-healthy diet, it's not necessarily off-limits. The key is to make smart choices and modify your pizza habits to reduce the negative impact on your heart health. Here's how:  Why pizza can be a concern:  1. High sodium content: Pizza can be high in sodium, which can increase blood pressure and contribute to heart disease.  2. Saturated fat and cholesterol: The cheese and meat on pizza can be high in saturated fat and cholesterol, which can raise LDL (bad) cholesterol levels and increase heart disease risk.  3. Refined carbohydrates: The crust is typically made from refined flour, which can cause a spike in blood sugar and insulin levels, potentially leading to insulin resistance and heart disease.  4. Portion sizes: Large pizza portions can lead to consuming excess calories, which can contribute to weight gain and obesity, a major risk factor for heart disease.  How to make pizza a heart-healthy option:  1. Choose a healthier crust: Opt for a whole-grain or whole-wheat crust, which will provide more fiber and nutrients compared to refined flour.  2. Load up on veggies: Add plenty of vegetables like mushrooms, bell peppers, onions, and tomatoes, which are rich in fiber, vitamins, and minerals.  3. Select lean protein sources: Opt for lean protein sources like chicken, turkey, or plant-based options like beans or tofu instead of pepperoni or sausage.  4. Go easy on the cheese: Use moderate amounts of low-fat cheese or try dairy-free alternatives like vegan mozzarella.  5. Limit portion sizes: Aim for a slice that's around 1/4 of the pizza (about 3-4 inches in diameter).  6. Be mindful of toppings: Avoid or limit high-sodium, high-fat toppings like bacon, sausage, and extra cheese.  7. Make your own pizza: By preparing your own pizza at home, you can control the ingredients and portion sizes.  Heart-healthy pizza ideas:  1. Veggie delight: Load up on vegetables like mushrooms, bell peppers, onions, and tomatoes, and use a whole-grain crust.  2. Grilled chicken and avocado: Top a whole-grain crust with grilled chicken, avocado, and a sprinkle of low-fat cheese.  3. Mediterranean style: Add feta cheese, olives, artichoke hearts, and sun-dried tomatoes to a whole-grain crust.  4. Roasted vegetable and bean: Top a whole-grain crust with roasted vegetables, black beans, and a sprinkle of low-fat cheese.  Remember, even with healthier modifications, pizza should be consumed in moderation as part of a balanced diet. Enjoy your pizza, but don't overdo it! |
| **Llama 3 Answer 3** | The eternal question! While pizza can be a challenge to incorporate into a heart-healthy diet, it's not impossible. Here's the good news: you can enjoy pizza in moderation, as long as you make some thoughtful choices to make it a healthier option.  Here are some tips to help you enjoy pizza while keeping your heart health in mind:  1. Opt for a whole-grain crust: Choose a crust made with whole grains like whole wheat, oats, or barley. This will increase the fiber content and reduce the glycemic index of your pizza.  2. Load up on veggies: Add plenty of vegetables like mushrooms, bell peppers, onions, and tomatoes. These will not only add fiber, vitamins, and antioxidants but also reduce the overall calorie and fat content of your pizza.  3. Go easy on the cheese: While cheese can be delicious on pizza, it's high in saturated fat, sodium, and calories. Opt for a lighter layer of cheese or choose a lower-fat variety like part-skim mozzarella or reduced-fat cheddar.  4. Select lean protein sources: Instead of pepperoni or sausage, choose lean protein sources like chicken, turkey, or vegetarian options like beans or tofu.  5. Limit the meat: If you do choose to add meat, keep the portion sizes in check. Aim for 2-3 slices of meat per pizza.  6. Watch portion sizes: Even with healthier toppings, pizza can be high in calories. Aim for a slice that's 1/4 to 1/3 of the entire pizza.  7. Make your own: Cooking your own pizza at home allows you to control the ingredients and portion sizes. Try using a pre-made whole-grain crust or making your own dough from scratch.  8. Look for heart-healthy toppings: Some pizza places offer heart-healthy toppings like olive oil, garlic, or artichokes. Take advantage of these options to add flavor without compromising your diet.  Some heart-healthy pizza options might look like this:  * Veggie lovers' pizza with whole-grain crust, mushrooms, bell peppers, onions, and part-skim mozzarella  * Grilled chicken pizza with whole-grain crust, chicken breast, tomatoes, and a sprinkle of feta cheese  * Mediterranean pizza with whole-grain crust, artichoke hearts, olives, and a drizzle of olive oil  Remember, even with healthier options, pizza should be consumed in moderation as part of a balanced diet. Aim to limit your pizza intake to 1-2 times per week and pair it with regular exercise and a healthy lifestyle. |
| **LLaMA3+RAG model Answer 1** | According to the American Heart Association (AHA), a heart-healthy diet should be low in salt, added sugar, tropical oil, alcohol, and processed foods, and high in non-starchy vegetables, fruits, whole grains, and legumes. Pizza, in its traditional form, often contains high amounts of salt, added sugar, and processed meats, making it a less-than-ideal choice for a heart-healthy diet.  However, it's not necessary to completely eliminate pizza from your diet. Instead, consider making some heart-healthy modifications:  1. Choose a whole-grain crust or a cauliflower-based crust to increase the fiber content.  2. Load up on vegetables such as bell peppers, onions, mushrooms, and olives to increase the nutrient density.  3. Opt for lean protein sources like chicken, turkey, or plant-based options like beans or tofu.  4. Limit the amount of cheese and processed meats like pepperoni and sausage.  5. Consider using herbs and spices to add flavor instead of salt.  It's also important to keep in mind the frequency and portion size of pizza consumption. The AHA recommends limiting dietary sodium to less than 2,300 milligrams per day, and consuming no more than 1,500 milligrams per day if you're at risk for high blood pressure or heart disease. A single slice of pizza can easily exceed these recommendations, so it's essential to be mindful of your overall sodium intake.  In summary, while pizza can be a part of a heart-healthy diet, it's crucial to make conscious choices about the ingredients and preparation methods used. By making a few simple modifications and being mindful of portion sizes, you can enjoy pizza while still prioritizing your heart health.  Sources:  American Heart Association. (2023). Here's How 10 Popular Diets Scored for Heart Health. Retrieved from <https://www.heart.org/en/news/2023/04/27/heres-how-10-popular-diets-scored-for-heart-health>  American Heart Association. (2023). 2023 Presidential Advisory: Dietary Guidance to Improve Cardiovascular Health. Retrieved from <https://www.ahajournals.org/doi/full/10.1161/CIR.0000000000001146> |
| **LLaMA3+RAG model Answer 2** | According to the American Heart Association (AHA), a heart-healthy diet emphasizes whole, unprocessed foods and limits or avoids foods high in salt, added sugar, saturated fats, and refined carbohydrates. A traditional pizza, with its thick crust, high-sodium sauce, melted cheese, and various meats, can be improved to fir your nutritional needs.  However, by making some tweaks, you can create a more heart-healthy pizza option:  1. Crust: Opt for a whole-grain or whole-wheat crust, which will provide more fiber and nutrients compared to refined white flour.  2. Sauce: Choose a low-sodium tomato sauce or make your own using fresh tomatoes, garlic, and herbs.  3. Cheese: Select lower-fat cheese options, such as part-skim mozzarella or reduced-fat cheddar. Use them in moderation, as cheese can still be high in saturated fat and sodium.  4. Toppings: Load up on vegetables like mushrooms, bell peppers, onions, and olives, which are rich in fiber, vitamins, and antioxidants. Consider lean protein sources like chicken or turkey instead of pepperoni or sausage.  5. Portion control: Keep your pizza slice sizes in check to avoid overconsumption of calories, fat, and sodium.  Here's an example of a heart-healthy pizza option:  Whole-grain crust  Low-sodium tomato sauce  Part-skim mozzarella cheese (used in moderation)  Topped with mushrooms, bell peppers, onions, olives, and lean chicken breast  Remember, even with healthier modifications, pizza should be consumed in moderation as part of a balanced diet. The AHA recommends limiting your daily intake of saturated fat, sodium, and added sugars to promote heart health.  Sources:  American Heart Association. (2023). Here's How 10 Popular Diets Scored for Heart Health. Retrieved from <https://www.heart.org/en/news/2023/04/27/heres-how-10-popular-diets-scored-for-heart-health>  American Heart Association. (2023). 2023 Dietary Guidance to Improve Cardiovascular Health. Retrieved from <https://www.ahajournals.org/doi/full/10.1161/CIR.0000000000001146>  Academy of Nutrition and Dietetics. (2020). Eat Right: Pizza, a Heart-Healthy Option? Retrieved from <https://www.eatright.org/food/planning-and-prep/snack-and-meal-tips/pizza-a-heart-healthy-option> |
| **LLaMA3+RAG model Answer 3** | According to the American Heart Association (AHA), a heart-healthy diet should be low in salt, added sugar, tropical oil, and processed foods, and high in non-starchy vegetables, fruits, whole grains, and legumes. Proteins should mostly come from plant sources, such as legumes, beans, or nuts, along with fish or seafood, lean poultry, and low-fat or fat-free dairy products.  A traditional pizza, loaded with pepperoni, sausage, and melted mozzarella cheese, is high in saturated fats, sodium, and processed meats, making it a less-than-ideal choice for a heart-healthy diet. However, you can modify your pizza to make it a healthier option.  Here are some tips to make your pizza more heart-healthy:  1. Choose a whole-grain crust or a cauliflower crust to increase the fiber content.  2. Load up on vegetables like mushrooms, bell peppers, onions, and olives, which are rich in antioxidants and fiber.  3. Opt for lean protein sources like chicken, turkey, or plant-based options like beans or tofu.  4. Use herbs and spices to add flavor instead of salt and sugar.  5. Limit the amount of cheese or choose a lower-fat option like part-skim mozzarella or ricotta.  6. Avoid processed meats like pepperoni and sausage, and instead choose nitrate-free options or plant-based alternatives.  By making these modifications, you can enjoy a heart-healthy pizza that aligns with the AHA's dietary guidelines. Remember, even with healthier options, pizza should be consumed in moderation as part of a balanced diet.  Sources:  American Heart Association. (2023). Here's how 10 popular diets scored for heart health. Retrieved from <https://www.heart.org/en/news/2023/04/27/heres-how-10-popular-diets-scored-for-heart-health>  American Heart Association. (2023). 2023 AHA Dietary Guidance. Retrieved from <https://www.ahajournals.org/doi/full/10.1161/CIR.0000000000001146> |
| **Question 15** | **How do I incorporate canned and frozen fruits and vegetables into my daily servings?** |
| **Perplexity Answer 1** | Here are some simple ways to incorporate canned and frozen fruits and vegetables into your daily servings:  Breakfast  - Add frozen berries or sliced bananas to your cereal, oatmeal or yogurt[1][3]  - Blend frozen fruit into a smoothie with yogurt and milk[3]  - Mix canned or frozen fruit into pancake or waffle batter[4]  Lunch  - Add canned corn, peas, or diced tomatoes to a salad[1][3]  - Use canned beans or lentils to make a hearty vegetable soup[4]  - Top a baked potato with canned broccoli or frozen mixed vegetables[4]  Dinner  - Stir frozen spinach or kale into pasta sauce[1][3]  - Add canned diced tomatoes or frozen peppers to chili or stew[3]  - Serve frozen broccoli, cauliflower or Brussels sprouts as a side dish[4]  Snacks  - Dip apple slices or carrot sticks into peanut butter[4]  - Enjoy a piece of fresh fruit or a small handful of dried fruit[3]  - Snack on frozen grapes or berries[1]  Some tips:  - Canned and frozen produce are just as nutritious as fresh, often more affordable, and have a longer shelf life[1][2]  - Look for canned fruit packed in water or 100% juice, and canned vegetables with no added salt[1][5]  - Rinse canned vegetables to reduce sodium content[1]  - Keep a variety of frozen fruits and veggies on hand for easy additions to meals[2][4]  The key is to always have canned and frozen options available to easily add extra servings to your meals and snacks throughout the day. With a little creativity, you can boost your fruit and veggie intake without much extra effort.  Citations:  [1] https://www.statesmanjournal.com/story/sponsor-story/kaiser-permanente/2016/01/21/maximizing-canned-and-frozen-produce-winter/79136540/  [2] https://frozenadvantage.org/frozen-fruits-and-vegetables-supporting-produce-consumption/  [3] https://www.cspinet.org/cspi-news/simple-inexpensive-ways-eat-more-fruit-and-veggies  [4] https://www.mayoclinichealthsystem.org/hometown-health/speaking-of-health/123-approach-to-eating-fruits-and-vegetables  [5] https://www.nhs.uk/live-well/eat-well/5-a-day/5-a-day-what-counts/ |
| **Perplexity Answer 1** | Here are some simple ways to incorporate canned and frozen fruits and vegetables into your daily servings:  - Add thawed frozen spinach or broccoli to pasta dishes, casseroles, soups and stews[1][3]  - Toss frozen berries into your morning smoothie or oatmeal[3]  - Stir a drained can of corn or diced tomatoes into chili, rice dishes, or pasta sauce[3]  - Roast frozen vegetables like Brussels sprouts, cauliflower or carrots for a quick side dish[4]  - Top a baked potato with canned black beans and salsa[4]  - Dip raw veggies like baby carrots or sliced bell peppers into hummus for a healthy snack[3]  - Scatter canned pineapple chunks or mandarin oranges over a pizza before baking[3]  - Add frozen fruit like mango, pineapple or berries to plain Greek yogurt[3]  Canned and frozen produce are just as nutritious as fresh, and can be more affordable and convenient[1][2]. Look for options with no added salt, sugar or sauces. Rinsing canned veggies can also reduce sodium[1].  Aim for at least 1 serving of fruits or veggies at each meal and snack to easily reach the recommended 5-9 servings per day[4]. With some simple swaps and additions, canned and frozen produce can help you boost your intake of these important foods.  Citations:  [1] https://www.statesmanjournal.com/story/sponsor-story/kaiser-permanente/2016/01/21/maximizing-canned-and-frozen-produce-winter/79136540/  [2] https://frozenadvantage.org/frozen-fruits-and-vegetables-supporting-produce-consumption/  [3] https://www.cspinet.org/cspi-news/simple-inexpensive-ways-eat-more-fruit-and-veggies  [4] https://www.mayoclinichealthsystem.org/hometown-health/speaking-of-health/123-approach-to-eating-fruits-and-vegetables  [5] https://www.nhs.uk/live-well/eat-well/5-a-day/5-a-day-what-counts/ |
| **Perplexity Answer 1** | Here are some simple ways to incorporate canned and frozen fruits and vegetables into your daily servings:  - Add frozen spinach or kale to your morning smoothie[1][3]. The greens blend right in with the fruit and yogurt.  - Toss a diced zucchini or some frozen broccoli into your pasta sauce[3]. The vegetables will bulk up the sauce and add extra nutrition.  - Keep a bag of frozen mixed vegetables on hand to quickly add to soups, stews, rice dishes, or as a side dish[1][3]. Frozen veggies are picked at peak ripeness so they retain nutrients.  - Drain and rinse canned beans, corn, or diced tomatoes to add to chili, tacos, or burritos[3]. Rinsing canned veggies reduces sodium.  - Top your cereal, oatmeal or yogurt with frozen berries or sliced peaches[4]. The fruit will thaw quickly and add sweetness.  - Stir a can of pineapple chunks into your favorite muffin or pancake batter[3]. The fruit adds moisture and flavor.  - Keep single-serve cups of applesauce or canned fruit in 100% juice on hand for an easy snack[3]. Look for varieties with no added sugar.  The key is to always have canned and frozen fruits and veggies stocked in your kitchen. They are nutritionally equivalent to fresh produce, often more affordable, and have a longer shelf life to reduce food waste[2]. With a little creativity, it's easy to add extra servings to your meals and snacks throughout the day.  Citations:  [1] https://www.statesmanjournal.com/story/sponsor-story/kaiser-permanente/2016/01/21/maximizing-canned-and-frozen-produce-winter/79136540/  [2] https://frozenadvantage.org/frozen-fruits-and-vegetables-supporting-produce-consumption/  [3] https://www.cspinet.org/cspi-news/simple-inexpensive-ways-eat-more-fruit-and-veggies  [4] https://www.mayoclinichealthsystem.org/hometown-health/speaking-of-health/123-approach-to-eating-fruits-and-vegetables  [5] https://www.nhs.uk/live-well/eat-well/5-a-day/5-a-day-what-counts/ |
| **GPT4o Answer 1** | Incorporating canned and frozen fruits and vegetables into your daily servings can be easy and convenient. Here are some practical tips and ideas to help you include these nutritious options in your meals:  Canned Fruits and Vegetables  1. Breakfast:  - Smoothies: Blend canned fruits (like peaches, pineapples, or pears) with yogurt or milk for a quick smoothie.  - Toppings: Add canned fruit to your oatmeal, cereal, or yogurt.  - Fruit Salad: Combine different types of canned fruits for a quick fruit salad.  2. Lunch:  - Salads: Add canned vegetables (like corn, beans, or beets) to your salads.  - Sandwiches and Wraps: Use canned vegetables like olives, artichoke hearts, or roasted red peppers as sandwich or wrap fillings.  - Soups: Use canned vegetables to prepare quick and easy soups.  3. Dinner:  - Side Dishes: Serve canned vegetables (like green beans, carrots, or peas) as side dishes.  - Casseroles and Stews: Incorporate canned vegetables into casseroles and stews for added nutrition and convenience.  - Stir-fries: Use canned vegetables in stir-fries with your choice of protein and sauce.  4. Snacks:  - Fruit Cups: Enjoy canned fruit cups as a healthy snack option.  - Dips: Blend canned beans to make healthy dips like hummus or bean dip.  Frozen Fruits and Vegetables  1. Breakfast:  - Smoothies: Use frozen fruits (like berries, mangoes, or bananas) in smoothies for a refreshing and nutritious start to your day.  - Pancake Toppings: Heat frozen fruits and use them as a topping for pancakes or waffles.  2. Lunch:  - Salads: Thaw and add frozen vegetables (like peas or corn) to salads for a quick addition.  - Wraps and Sandwiches: Include thawed frozen vegetables in wraps or sandwiches.  3. Dinner:  - Stir-fries: Use frozen vegetables in stir-fries for a quick and easy dinner option.  - Pasta Dishes: Add frozen vegetables to pasta dishes for added flavor and nutrients.  - Soups and Stews: Incorporate frozen vegetables into soups and stews.  4. Snacks:  - Frozen Fruit Snacks: Enjoy frozen fruits like grapes, blueberries, or mangoes straight from the freezer as a refreshing snack.  - Yogurt or Cottage Cheese: Mix frozen fruits into yogurt or cottage cheese.  General Tips  - Rinse Canned Vegetables: If you are concerned about sodium content, rinse canned vegetables before use to reduce sodium levels.  - Choose No Added Sugar or Low Sodium Options: Opt for canned fruits in juice rather than syrup and low-sodium or no-salt-added canned vegetables.  - Portion Control: Be mindful of portion sizes to ensure you are meeting your daily fruit and vegetable intake without overconsuming.  By creatively incorporating canned and frozen fruits and vegetables into your meals and snacks, you can easily increase your daily servings and enjoy the convenience they offer. |
| **GPT4o Answer 2** | Incorporating canned and frozen fruits and vegetables into your daily servings is a convenient and nutritious way to meet your dietary needs. Here are some tips and ideas on how to do it effectively:  Breakfast  1. Smoothies: Use frozen fruits like berries, mango, or peaches in your morning smoothie. Add some spinach or kale for an extra boost.  2. Oatmeal: Mix in canned fruits such as peaches, pears, or pineapple into your oatmeal for added sweetness and nutrients.  3. Yogurt: Top your yogurt with canned or frozen fruits like blueberries, strawberries, or mixed fruit.  Lunch  1. Salads: Add canned beans (like black beans or chickpeas) and corn to your salads. Frozen peas or edamame can also be tossed into your salad for extra protein and fiber.  2. Wraps and Sandwiches: Include canned vegetables like roasted red peppers or artichoke hearts in your wraps and sandwiches.  3. Soups: Enhance your soups with frozen vegetables like peas, corn, green beans, or mixed vegetables.  Dinner  1. Stir-Fries: Use a mix of frozen vegetables like broccoli, bell peppers, and snap peas in your stir-fries.  2. Casseroles: Incorporate canned vegetables like tomatoes, green beans, or mushrooms in casseroles and baked dishes.  3. Side Dishes: Serve canned or frozen vegetables as side dishes. For example, canned carrots or green beans can be heated and seasoned for a quick side.  Snacks  1. Fruit Cups: Keep single-serving canned fruit cups (in juice, not syrup) on hand for a quick snack.  2. Frozen Fruit Snacks: Enjoy frozen grapes, berries, or mango pieces as a refreshing snack.  3. Veggie Dips: Dip frozen or canned vegetables like baby corn, carrots, or peas into hummus or a yogurt-based dip.  Desserts  1. Fruit Sorbet: Blend frozen fruits with a bit of juice or yogurt to make a quick and healthy sorbet.  2. Fruit Compotes: Use canned fruits to make a compote, which can be served over ice cream, yogurt, or pancakes.  Tips for Using Canned and Frozen Produce  - Rinse Canned Vegetables: Rinse canned vegetables to reduce sodium content.  - Check Labels: Look for canned fruits packed in juice rather than syrup to avoid added sugars.  - Proper Storage: Store frozen fruits and vegetables properly to maintain their quality. Keep them in a freezer at 0°F (-18°C) or lower.  - Portion Control: Measure out servings to ensure you’re getting the right amount of fruits and vegetables as per dietary guidelines.  By incorporating these ideas into your meals and snacks, you can easily increase your daily intake of fruits and vegetables using convenient canned and frozen options. |
| **GPT4o Answer 3** | Incorporating canned and frozen fruits and vegetables into your daily servings can be an easy and convenient way to ensure you're getting enough produce. Here are some practical tips:  Breakfast  1. Smoothies: Add frozen fruits like berries, mango, or peaches to your smoothies. They blend well and create a thick, cold texture.  2. Oatmeal or Cereal Toppings: Top your oatmeal or cereal with canned or frozen fruits. Just thaw the frozen fruits before adding them.  3. Egg Dishes: Include canned or frozen vegetables like spinach, bell peppers, or mushrooms in omelets or scrambled eggs.  Snacks  1. Yogurt Mix: Mix thawed frozen fruits or drained canned fruits with yogurt for a quick snack.  2. Fruit Salad: Create a fruit salad using a mix of canned and thawed frozen fruits.  Lunch  1. Salads: Add drained canned vegetables like corn, peas, or beans to your salads.  2. Sandwiches and Wraps: Include canned vegetables like roasted red peppers or artichoke hearts in your sandwiches and wraps.  3. Soups: Use canned or frozen vegetables in soups. They are already prepped and cook quickly.  Dinner  1. Stir-Fries: Use a mix of frozen vegetables in your stir-fries. They cook quickly and retain their nutrients.  2. Casseroles and Bakes: Add canned or frozen vegetables to casseroles, bakes, or pasta dishes.  3. Side Dishes: Serve canned or frozen vegetables as side dishes. Simply heat and season to taste.  Tips for Using Canned and Frozen Produce  1. Rinse Canned Vegetables: Rinse canned vegetables to remove excess sodium.  2. No Added Sugars: Choose canned fruits packed in water or natural juice, not syrup, to avoid added sugars.  3. Proper Thawing: Thaw frozen fruits and vegetables in the refrigerator or under cold running water if you plan to use them soon.  4. Season and Flavor: Enhance the flavor of canned and frozen produce with herbs, spices, or a splash of lemon juice.  Examples of Incorporating Them Daily  1. Breakfast Smoothie: Blend spinach (frozen), berries (frozen), and a banana with yogurt and milk.  2. Mid-Morning Snack: Have a small bowl of fruit salad with canned pineapple, mandarin oranges, and thawed strawberries.  3. Lunch Salad: Toss together a salad with lettuce, canned chickpeas, frozen corn (thawed), and cherry tomatoes.  4. Afternoon Snack: Enjoy a yogurt parfait with layers of yogurt, thawed frozen berries, and a drizzle of honey.  5. Dinner: Make a stir-fry with frozen mixed vegetables, tofu or chicken, and your favorite sauce.  By integrating canned and frozen fruits and vegetables into your meals and snacks, you can easily meet your daily servings and enjoy a variety of flavors and nutrients. |
| **Llama 3 Answer 1** | Incorporating canned and frozen fruits and vegetables into your daily diet can be a convenient and affordable way to meet your daily serving goals. Here are some tips to help you do so:  Canned Fruits and Vegetables:  1. Choose low-sodium options: Opt for canned fruits and vegetables that are low in sodium or rinse them with water to remove excess salt.  2. Drain and rinse: Drain the liquid from canned fruits and vegetables and rinse them with water to remove added sugars or salt.  3. Use in recipes: Add canned fruits and vegetables to soups, stews, salads, and casseroles to increase their nutritional value.  4. Make a fruit salad: Mix canned fruits like mandarin oranges, pineapple, and fruit cocktail with fresh fruits like berries, bananas, or apples.  5. Add to oatmeal or yogurt: Mix canned fruits with oatmeal or yogurt for a quick and easy breakfast or snack.  Frozen Fruits and Vegetables:  1. Freeze them yourself: Freeze fresh fruits and vegetables at their peak ripeness to preserve their nutrients and flavor.  2. Thaw frozen fruits: Thaw frozen fruits like berries, cherries, or peaches and use them in smoothies, salads, or as a topping for oatmeal or yogurt.  3. Steam frozen vegetables: Steam frozen vegetables like broccoli, cauliflower, or green beans to preserve their nutrients.  4. Add to soups and stews: Add frozen vegetables to soups and stews for added nutrition and flavor.  5. Make a frozen fruit popsicle: Blend frozen fruits with a little water and freeze them in popsicle molds for a healthy and refreshing snack.  Tips for Both Canned and Frozen:  1. Check expiration dates: Make sure to check the expiration dates of canned and frozen fruits and vegetables before consuming them.  2. Store properly: Store canned fruits and vegetables in a cool, dry place, and frozen fruits and vegetables in airtight containers or freezer bags.  3. Vary your choices: Mix and match different types of canned and frozen fruits and vegetables to ensure you're getting a variety of nutrients.  4. Use them in place of fresh: Use canned or frozen fruits and vegetables when fresh options are not available or out of season.  5. Consult with a registered dietitian: If you have specific dietary needs or concerns, consult with a registered dietitian for personalized advice on incorporating canned and frozen fruits and vegetables into your daily diet.  By following these tips, you can easily incorporate canned and frozen fruits and vegetables into your daily diet, increasing your consumption of essential nutrients and meeting your daily serving goals. |
| **Llama 3 Answer 2** | Incorporating canned and frozen fruits and vegetables into your daily servings can be a convenient and affordable way to meet your daily nutrient needs. Here are some tips to help you do so:  Canned Fruits and Vegetables:  1. Choose low-sodium options: Opt for canned fruits and vegetables that are labeled "low sodium" or "no salt added" to reduce your sodium intake.  2. Rinse with water: Drain and rinse canned fruits and vegetables with water to remove excess sodium and added sugars.  3. Use in cooking: Canned tomatoes, beans, and corn are great additions to soups, stews, and stir-fries.  4. Add to salads: Canned fruits like mandarin oranges or pineapple can be a tasty addition to green salads or fruit salads.  5. Make a fruit salad: Mix canned fruits like peaches, pears, or fruit cocktail with fresh fruits for a quick and easy snack.  Frozen Fruits and Vegetables:  1. Choose flash-frozen options: Flash-frozen fruits and vegetables are frozen soon after harvesting, which helps preserve their nutrient content.  2. Use in smoothies: Frozen fruits like berries, bananas, or mango make great additions to smoothies.  3. Steam or roast: Frozen vegetables like broccoli, cauliflower, or green beans can be steamed or roasted as a side dish.  4. Add to soups and stews: Frozen vegetables like peas, carrots, or corn can be added to soups and stews for added nutrition.  5. Make a veggie mix: Mix frozen vegetables like peas, carrots, and corn for a quick and easy stir-fry or sauté.  General Tips:  1. Check expiration dates: Make sure to check the expiration dates of canned and frozen fruits and vegetables before consuming them.  2. Store properly: Store canned goods in a cool, dry place, and frozen fruits and vegetables in airtight containers or freezer bags.  3. Variety is key: Incorporate a variety of canned and frozen fruits and vegetables into your diet to ensure you're getting a range of nutrients.  4. Combine with fresh options: Mix canned and frozen fruits and vegetables with fresh options to increase nutrient density and flavor.  5. Consult with a registered dietitian: If you have specific dietary needs or restrictions, consult with a registered dietitian for personalized guidance on incorporating canned and frozen fruits and vegetables into your diet.  By following these tips, you can easily incorporate canned and frozen fruits and vegetables into your daily servings, ensuring you're meeting your nutrient needs while saving time and money. |
| **Llama 3 Answer 3** | Incorporating canned and frozen fruits and vegetables into your daily servings can be a convenient and nutritious way to meet your daily recommended intake. Here are some tips to help you do so:  Canned Fruits and Vegetables:  1. Choose low-sodium options: Opt for canned fruits and vegetables that are labeled "low sodium" or "no salt added" to reduce your sodium intake.  2. Rinse with water: Rinse canned beans and vegetables with water to remove excess sodium.  3. Use in soups and stews: Add canned vegetables like tomatoes, beans, and corn to soups and stews for added nutrition and flavor.  4. Make a salad: Mix canned fruits like mandarin oranges or pineapple with fresh greens and nuts for a quick and easy salad.  5. Add to oatmeal or yogurt: Mix canned fruits like peaches or pears with oatmeal or yogurt for a healthy breakfast or snack.  Frozen Fruits and Vegetables:  1. Thaw and use in smoothies: Thaw frozen fruits like berries, bananas, or mangoes and blend into smoothies for a quick and nutritious snack.  2. Roast or sauté: Roast or sauté frozen vegetables like broccoli, cauliflower, or Brussels sprouts with olive oil and seasonings for a healthy side dish.  3. Add to soups and stews: Add frozen vegetables like peas, carrots, or green beans to soups and stews for added nutrition.  4. Make a stir-fry: Stir-fry frozen vegetables like bell peppers, onions, and mushrooms with your favorite protein and seasonings for a quick and easy dinner.  5. Use in baked goods: Use frozen fruits like blueberries or raspberries in muffins, cakes, or bread recipes for added flavor and nutrition.  General Tips:  1. Check expiration dates: Always check the expiration dates of canned and frozen fruits and vegetables to ensure they are safe to consume.  2. Store properly: Store canned goods in a cool, dry place, and frozen fruits and vegetables in airtight containers or freezer bags.  3. Variety is key: Mix and match different types of canned and frozen fruits and vegetables to ensure you're getting a wide range of nutrients.  4. Consult with a registered dietitian: If you have specific dietary needs or restrictions, consult with a registered dietitian for personalized advice on incorporating canned and frozen fruits and vegetables into your diet.  By following these tips, you can easily incorporate canned and frozen fruits and vegetables into your daily servings and reap the nutritional benefits they offer. |
| **LLaMA3+RAG model Answer 1** | Incorporating canned and frozen fruits and vegetables into your daily servings can be a convenient and economical way to meet the recommended 4½ cups of daily intake. Here are some tips to help you do so:  Canned Fruits and Vegetables:  1. Check the sodium content: When selecting canned fruits and vegetables, opt for products with low sodium (<140 mg), reduced sodium (25% less sodium than regular canned produce), or no salt added (<5mg).  2. Rinse and drain: Rinse and drain canned foods before eating to reduce sodium intake.  3. Choose wisely: Select fruits canned in water, their own juice, or light syrup to avoid added sugars.  4. Get creative: Use canned fruits and vegetables in a variety of dishes, such as:  Vegetable soups: Add drained and rinsed canned vegetables to low-sodium chicken broth.  Bean dips: Blend drained and rinsed beans with olive oil, tahini, lemon juice, and garlic powder.  Healthy desserts: Top canned fruit with fat-free/low-fat, no-sugar-added yogurt.  Breakfast toppings: Add canned fruit to whole-grain cereal.  Frozen Fruits and Vegetables:  1. Check the sodium content: Compare sodium content on the Nutrition Facts label and choose products with the lowest amount.  2. Opt for 100% fruits: Select frozen fruits that have not been sweetened with added sugars.  3. Get creative: Use frozen fruits and vegetables in a variety of dishes, such as:  Pasta dishes: Add frozen vegetables to boiling water at the end of cooking time.  Smoothies: Blend frozen fruit with fat-free/low-fat milk or yogurt.  Baked goods: Fold frozen berries into oatmeal or baked goods.  Tips for Incorporating Both Canned and Frozen Fruits and Vegetables:  1. Stock up: Keep a supply of canned and frozen fruits and vegetables in your pantry and freezer to ensure you always have a healthy option available.  2. Plan ahead: Incorporate canned and frozen fruits and vegetables into your meal planning and grocery shopping.  3. Experiment with recipes: Try new recipes that incorporate canned and frozen fruits and vegetables to keep your diet interesting and varied.  By following these tips, you can easily incorporate canned and frozen fruits and vegetables into your daily servings, ensuring you meet the recommended daily intake and maintain a healthy diet.  Sources:  American Heart Association. (2024). Fresh, Frozen or Canned Fruits and Vegetables: All Can Be Healthy Choices. Retrieved from <https://www.heart.org/en/healthy-living/healthy-eating/add-color/fresh-frozen-or-canned-fruits-and-vegetables-all-can-be-healthy-choices> |
| **LLaMA3+RAG model Answer 2** | Incorporating canned and frozen fruits and vegetables into your daily servings can be a convenient and nutritious way to meet the recommended 4½ cups of each daily. Here are some tips to help you do so:  Canned Fruits and Vegetables:  1. Check the sodium content: When choosing canned fruits and vegetables, opt for products with low sodium (<140 mg), reduced sodium (25% less sodium than the regular canned produce), or no salt added (<5mg).  2. Rinse and drain: Rinse and drain canned foods before eating to reduce sodium intake.  3. Choose products with no added sugar: Select fruit that's canned in water, its own juice, or light syrup.  4. Get creative with recipes: Use canned fruits and vegetables in a variety of dishes, such as:  Vegetable soup with fat-free, low-sodium chicken broth and drained and rinsed canned vegetables.  Healthy bean dip with drained and rinsed beans, olive oil, tahini, lemon juice, and garlic powder.  Canned fruit topped with fat-free/low-fat, no-sugar-added yogurt for a healthy dessert.  Canned fruit on whole-grain cereal for breakfast.  Frozen Fruit and Vegetables:  1. Check the sodium content: Compare the sodium content on the Nutrition Facts label and choose the product with the lowest amount.  2. Choose 100% fruits without added sugars: Opt for frozen fruits that have been frozen without added sugars.  3. Get creative with recipes: Use frozen fruits and vegetables in a variety of dishes, such as:  Adding frozen veggies to boiling pasta water for added flavor, texture, and nutrition.  Blending frozen fruit with fat-free/low-fat milk or fat-free/low-fat no-added-sugar yogurt.  Gently folding frozen berries into baked goods and oatmeal.  Tips for Incorporating Canned and Frozen Fruits and Vegetables into Your Daily Servings:  1. Aim for variety: Include a mix of canned, frozen, and fresh fruits and vegetables in your diet to ensure you're getting a range of nutrients.  2. Plan ahead: Stock up on canned and frozen fruits and vegetables to have them on hand for quick and easy meals.  3. Experiment with new recipes: Try new recipes that incorporate canned and frozen fruits and vegetables to keep your diet interesting and varied.  4. Make it convenient: Keep canned and frozen fruits and vegetables in easily accessible locations, such as the pantry or freezer, to encourage frequent use.  By following these tips, you can easily incorporate canned and frozen fruits and vegetables into your daily servings and reap the nutritional benefits they provide.  Sources:  American Heart Association. (2024). Fresh, Frozen or Canned Fruits and Vegetables: All Can Be Healthy Choices. Retrieved from <https://www.heart.org/en/healthy-living/healthy-eating/add-color/fresh-frozen-or-canned-fruits-and-vegetables-all-can-be-healthy-choices> |
| **LLaMA3+RAG model Answer 3** | Incorporating canned and frozen fruits and vegetables into your daily servings can be a convenient and nutritious way to meet the recommended 4½ cups of each daily. Here are some tips to help you do so:  Canned Fruits and Vegetables:  1. Check the sodium content: When choosing canned fruits and vegetables, check the Nutrition Facts label and opt for products with low sodium (<140 mg), reduced sodium (25% less sodium than the regular canned produce), or no salt added (<5mg).  2. Rinse and drain: Rinse and drain canned fruits and vegetables before consuming to reduce sodium intake.  3. Choose wisely: Select fruits canned in water, its own juice, or light syrup to avoid added sugars.  4. Get creative: Use canned fruits and vegetables in a variety of dishes, such as:  Adding drained and rinsed cans of no-salt-added or reduced-sodium corn, tomatoes, and pinto beans to fat-free, low-sodium chicken broth for a quick and filling vegetable soup.  Making a healthy bean dip by blending drained and rinsed beans with olive oil, tahini, lemon juice, and garlic powder.  Serving canned fruit topped with fat-free/low-fat, no-sugar-added yogurt for a healthy dessert.  Topping whole-grain cereal with canned fruit for a nutritious breakfast.  Frozen Fruits and Vegetables:  1. Check the sodium content: Compare the sodium content on the Nutrition Facts label and choose products with the lowest amount. Be aware that vegetables frozen with added sauces and seasonings usually contain excess sodium and calories.  2. Choose 100% fruits: Opt for 100% fruits that have been frozen without added sugars.  3. Get creative: Use frozen fruits and vegetables in a variety of dishes, such as:  Adding frozen veggies to boiling water for pasta to add flavor, texture, and nutrition.  Blending frozen fruit with fat-free/low-fat milk or fat-free/low-fat no-added-sugar yogurt.  Gently folding frozen berries into baked goods and oatmeal.  Tips for Incorporating Canned and Frozen Fruits and Vegetables into Your Daily Servings:  1. Aim for variety: Incorporate a mix of canned, frozen, and fresh fruits and vegetables into your daily servings to ensure you're getting a range of nutrients.  2. Plan ahead: Stock up on canned and frozen fruits and vegetables to have them on hand for quick and easy meal prep.  3. Experiment with recipes: Try new recipes that incorporate canned and frozen fruits and vegetables to keep your diet interesting and varied.  4. Make it convenient: Keep canned and frozen fruits and vegetables in easily accessible locations, such as the pantry or freezer, to encourage healthy snacking and meal prep.  By following these tips, you can easily incorporate canned and frozen fruits and vegetables into your daily servings and reap the nutritional benefits they provide.  Sources:  American Heart Association. (2024). Fresh, Frozen or Canned Fruits and Vegetables: All Can Be Healthy Choices. Retrieved from <https://www.heart.org/en/healthy-living/healthy-eating/add-color/fresh-frozen-or-canned-fruits-and-vegetables-all-can-be-healthy-choices> |
| **Question 16** | **Which foods should I avoid on a heart healthy diet?** |
| **Perplexity Answer 1** | Here are the main foods to avoid on a heart-healthy diet:  1. Saturated and trans fats: Found in fried foods, baked goods, snack foods, and fatty meats. These unhealthy fats raise LDL (bad) cholesterol levels[9].  2. Added sugars: Found in sodas, sweets, and many processed foods. Added sugars provide calories without nutrients and can contribute to weight gain and inflammation[9].  3. Sodium: Found in processed, canned, and restaurant foods. Too much sodium can raise blood pressure. Aim for less than 2,300 mg per day[9].  4. Processed meats: Bacon, sausage, deli meats, and hot dogs are high in saturated fat, sodium, and preservatives that may increase heart disease risk[9].  5. Refined carbs: Found in white bread, pastries, and other foods made with white flour. Refined carbs spike blood sugar and provide calories without nutrients[9].  6. Alcohol: Heavy drinking can raise blood pressure and triglycerides. Moderate drinking (up to 1 drink per day for women, 2 for men) may be okay for some people[9].  Instead, focus on eating plenty of fruits, vegetables, whole grains, lean proteins, and healthy fats like olive oil and nuts. Staying hydrated with water is also important. Following a Mediterranean-style diet is one of the best ways to eat for heart health[9].  Citations:  [1] https://www.yelp.com/biz/bakesale-betty-oakland?adjust_creative=6zagVcKwffYZAA-51mlGNg  [2] https://www.yelp.com/biz/smokin-woods-bbq-oakland-9?adjust_creative=6zagVcKwffYZAA-51mlGNg  [3] https://www.yelp.com/biz/homeroom-oakland?adjust_creative=6zagVcKwffYZAA-51mlGNg  [4] https://www.yelp.com/biz/fob-kitchen-oakland?adjust_creative=6zagVcKwffYZAA-51mlGNg  [5] https://www.yelp.com/biz/clove-and-hoof-oakland-2?adjust_creative=6zagVcKwffYZAA-51mlGNg  [6] https://www.yelp.com/biz/barcote-ethiopian-restaurant-oakland?adjust_creative=6zagVcKwffYZAA-51mlGNg  [7] https://www.yelp.com/biz/north-light-oakland?adjust_creative=6zagVcKwffYZAA-51mlGNg  [8] https://www.yelp.com/biz/branch-line-bar-emeryville-17?adjust_creative=6zagVcKwffYZAA-51mlGNg  [9] https://riversedgehealth.org/10-foods-for-a-heart-healthy-cardiac-diet-and-6-foods-to-avoid/  [10] https://www.unitypoint.org/news-and-articles/foods-to-love-foods-to-avoid-for-a-heart-healthy-diet-infographic  [11] https://health.ucdavis.edu/blog/good-food/im-a-cardiovascular-dietitian-here-are-7-things-i-eat-and-avoid-for-heart-health/2023/02  [12] https://www.health.harvard.edu/heart-health/heart-healthy-foods-what-to-eat-and-what-to-avoid  [13] https://www.mayoclinic.org/diseases-conditions/heart-disease/in-depth/heart-healthy-diet/art-20047702 |
| **Perplexity Answer 2** | To avoid on a heart-healthy diet, limit or eliminate the following foods:  - Red and processed meats like beef, pork, and deli meats[1][3]  - Fried foods[2]  - Baked goods high in saturated and trans fats like cakes, cookies, pie crust, and crackers[2][4]  - Sugary foods and drinks like soda[1][4]  - White breads, pasta, and refined carbohydrates[1][4]  - High-sodium foods like microwave popcorn and canned soups[2][4]  - Tropical oils like coconut and palm oil, as well as butter and margarine[1][4]  - Alcohol, especially in excess[1][4]  Instead, focus on eating a diet rich in:  - Fruits and vegetables of all colors[1][2][4]  - Whole grains like oats, quinoa, and brown rice[1][2][3]  - Lean proteins like fish, poultry, beans, lentils, and low-fat dairy[1][2][3]  - Healthy fats from olive oil, nuts, seeds, and avocados[3][4]  By limiting saturated and trans fats, sodium, added sugars, and processed foods while emphasizing whole, minimally processed foods, you can significantly reduce your risk of heart disease. Consult with a registered dietitian for personalized dietary guidance.  Citations:  [1] https://riversedgehealth.org/10-foods-for-a-heart-healthy-cardiac-diet-and-6-foods-to-avoid/  [2] https://www.unitypoint.org/news-and-articles/foods-to-love-foods-to-avoid-for-a-heart-healthy-diet-infographic  [3] https://health.ucdavis.edu/blog/good-food/im-a-cardiovascular-dietitian-here-are-7-things-i-eat-and-avoid-for-heart-health/2023/02  [4] https://www.health.harvard.edu/heart-health/heart-healthy-foods-what-to-eat-and-what-to-avoid  [5] https://health.gov/myhealthfinder/health-conditions/heart-health/heart-healthy-foods-shopping-list |
| **Perplexity Answer 3** | To avoid on a heart-healthy diet, limit or eliminate the following foods:  - Red and processed meats like beef, pork, and deli meats, as they increase heart disease risk[1][3]  - Fried foods, which are high in unhealthy fats[2][4]  - Baked goods like cakes, cookies, pie crust, and crackers, which often contain trans fats and refined carbs[1][2][4]  - Sugary foods and drinks like soda, which can raise heart disease and diabetes risk[1][4]  - Foods high in saturated and trans fats like butter, margarine, shortening, and tropical oils[2][4]  - White breads, pastas, and refined carbohydrates, which lack fiber and nutrients[1][4]  - Salty foods, as excess sodium can raise blood pressure[1][4]  - Alcohol, which should be limited to no more than one drink per day for women and two for men[1][4]  Instead, focus on eating plenty of fruits, vegetables, whole grains, lean proteins, nuts, seeds, and healthy oils like olive oil. Choosing minimally processed foods and limiting sodium, added sugars, and unhealthy fats is key for a heart-healthy diet[3][4][5].  Citations:  [1] https://riversedgehealth.org/10-foods-for-a-heart-healthy-cardiac-diet-and-6-foods-to-avoid/  [2] https://www.unitypoint.org/news-and-articles/foods-to-love-foods-to-avoid-for-a-heart-healthy-diet-infographic  [3] https://health.ucdavis.edu/blog/good-food/im-a-cardiovascular-dietitian-here-are-7-things-i-eat-and-avoid-for-heart-health/2023/02  [4] https://www.health.harvard.edu/heart-health/heart-healthy-foods-what-to-eat-and-what-to-avoid  [5] https://www.mayoclinic.org/diseases-conditions/heart-disease/in-depth/heart-healthy-diet/art-20047702 |
| **GPT4o Answer 1** | To maintain a heart-healthy diet, it's important to avoid or limit certain foods that can negatively impact your cardiovascular health. Here are some key foods to avoid:  1. Trans Fats:  - Found in: Margarine, fried foods, baked goods (such as cookies, cakes, and pies), and some processed snacks.  - Reason: Trans fats increase bad cholesterol (LDL) and decrease good cholesterol (HDL), raising the risk of heart disease.  2. Saturated Fats:  - Found in: Fatty cuts of meat, poultry skin, full-fat dairy products (butter, cheese, cream), and tropical oils (coconut oil, palm oil).  - Reason: Saturated fats can raise cholesterol levels, leading to artery clogging.  3. Salt (Sodium):  - Found in: Processed and packaged foods (canned soups, deli meats, frozen meals), restaurant and fast food, and salty snacks (chips, pretzels).  - Reason: Excessive sodium intake can raise blood pressure, increasing the risk of heart disease and stroke.  4. Added Sugars:  - Found in: Sugary beverages (sodas, energy drinks), sweets (candy, cookies, pastries), and sweetened cereals.  - Reason: High sugar intake can lead to weight gain, increased blood pressure, and higher triglyceride levels, all of which are risk factors for heart disease.  5. Refined Carbohydrates:  - Found in: White bread, white rice, pastries, and many breakfast cereals.  - Reason: These can cause spikes in blood sugar levels and contribute to insulin resistance and weight gain.  6. Processed Meats:  - Found in: Sausages, hot dogs, bacon, and deli meats.  - Reason: These are often high in sodium, saturated fats, and preservatives, all of which can contribute to heart disease.  7. High-Fat Dairy Products:  - Found in: Whole milk, butter, full-fat yogurt, and cheese.  - Reason: High-fat dairy products contain saturated fats that can raise cholesterol levels.  8. Alcohol:  - Reason: While moderate alcohol consumption might have some heart benefits, excessive drinking can lead to high blood pressure, heart failure, and stroke.  Tips for a Heart-Healthy Diet:  - Focus on Whole Foods: Emphasize fruits, vegetables, whole grains, lean proteins, and healthy fats (such as those from nuts, seeds, avocados, and fish).  - Read Labels: Be mindful of the ingredients and nutritional content of packaged foods, especially regarding trans fats, sodium, and added sugars.  - Cook at Home: Preparing meals at home allows you to control the ingredients and cooking methods, reducing the intake of unhealthy fats, sodium, and sugars.  - Healthy Fats: Use olive oil or canola oil instead of butter or lard.  By avoiding these foods and focusing on a balanced diet rich in nutrients, you can significantly improve your heart health and reduce the risk of cardiovascular diseases. |
| **GPT4o Answer 2** | On a heart-healthy diet, it’s important to avoid or limit certain foods that can contribute to heart disease. Here are some key categories of foods to avoid:  1. Trans Fats: These are often found in:  - Partially hydrogenated oils  - Some margarines  - Commercially baked goods (cookies, cakes, pies)  - Fried fast foods  2. Saturated Fats: Common sources include:  - Fatty cuts of meat (beef, lamb, pork)  - Poultry skin  - Full-fat dairy products (butter, cream, cheese)  - Lard and cream-based sauces  3. Added Sugars: Found in:  - Sugary drinks (sodas, energy drinks)  - Sweets (candies, chocolates)  - Desserts (cakes, pastries, ice creams)  - Some cereals and snack bars  4. Refined Carbohydrates: Such as:  - White bread and pasta  - Pastries and other baked goods made with white flour  - Sugary cereals  5. Excessive Sodium: High-sodium foods include:  - Processed meats (bacon, sausages, deli meats)  - Canned soups and vegetables (unless labeled low-sodium)  - Salty snacks (potato chips, pretzels)  - Some frozen dinners  6. Processed Foods: These often contain unhealthy fats, sugars, and sodium, including:  - Packaged snacks and baked goods  - Ready-to-eat meals  - Instant noodles and soups  7. Alcohol: Excessive alcohol consumption can lead to high blood pressure and heart failure. Moderation is key:  - Men: No more than two drinks per day  - Women: No more than one drink per day  In addition to avoiding these foods, focusing on a diet rich in fruits, vegetables, whole grains, lean proteins, and healthy fats (like those from nuts, seeds, and olive oil) can promote heart health. |
| **GPT4o Answer 3** | On a heart-healthy diet, it is important to avoid or limit certain foods that can negatively impact cardiovascular health. Here are some key foods to avoid or reduce:  1. Trans Fats and Hydrogenated Oils:  - Found in: Processed snacks, baked goods (cakes, cookies, pies), margarines, and fried foods.  - Reason: Increase bad cholesterol (LDL) and decrease good cholesterol (HDL), leading to heart disease.  2. Saturated Fats:  - Found in: Red meat, full-fat dairy products (butter, cheese, cream), coconut oil, palm oil.  - Reason: Can raise total cholesterol levels and increase the risk of heart disease.  3. Sugary Foods and Beverages:  - Found in: Sodas, sweetened juices, candies, desserts, and many processed foods.  - Reason: Contribute to obesity, type 2 diabetes, and heart disease.  4. Excessive Sodium:  - Found in: Processed foods, canned soups, fast foods, deli meats, and snacks like chips.  - Reason: High sodium intake can lead to high blood pressure, a risk factor for heart disease.  5. Refined Carbohydrates and Processed Grains:  - Found in: White bread, white rice, pastries, and many processed snacks.  - Reason: Can spike blood sugar levels and contribute to obesity and type 2 diabetes.  6. High-Cholesterol Foods:  - Found in: Organ meats (liver), shellfish, and egg yolks (in large amounts).  - Reason: While dietary cholesterol has less impact on blood cholesterol than once thought, it is still advisable to consume these foods in moderation, especially if you have high cholesterol.  7. Alcohol (in excess):  - Found in: Beer, wine, and spirits.  - Reason: Excessive alcohol intake can lead to high blood pressure, heart failure, and even stroke. Moderate consumption is key.  8. Processed Meats:  - Found in: Bacon, sausage, hot dogs, and deli meats.  - Reason: High in sodium, saturated fats, and preservatives that can contribute to heart disease.  9. Commercially Baked Goods:  - Found in: Doughnuts, cakes, pastries, and cookies.  - Reason: Often high in trans fats, sugars, and refined carbs, which are detrimental to heart health.  Instead, focus on consuming a variety of whole, nutrient-dense foods such as fruits, vegetables, whole grains, lean proteins (like fish and poultry), nuts, seeds, and healthy fats (such as those from avocados, olive oil, and fatty fish). These foods can help support cardiovascular health and reduce the risk of heart disease. |
| **Llama 3 Answer 1** | On a heart-healthy diet, it's essential to limit or avoid foods that can increase your risk of heart disease, high blood pressure, and high cholesterol. Here are some foods to limit or avoid:  1. Saturated and Trans Fats:  * Red meat (opt for lean cuts or choose plant-based alternatives)  * Full-fat dairy products (choose low-fat or fat-free options)  * Processed meats (e.g., hot dogs, sausages, bacon)  * Fried foods and baked goods (e.g., donuts, pastries)  * Partially hydrogenated oils (e.g., partially hydrogenated soybean oil)  2. Refined Carbohydrates:  * White bread and sugary baked goods  * Refined grains (e.g., white rice, pasta)  * Sugary drinks (e.g., soda, sports drinks, sweet tea)  3. High-Sodium Foods:  * Processed and packaged foods (e.g., canned soups, frozen meals)  * Cured meats (e.g., bacon, ham)  * Soy sauce and other high-sodium condiments  4. Added Sugars:  * Sugary snacks (e.g., candy, cookies)  * Fruit juices and sweetened beverages  * Refined grains with added sugars (e.g., sweetened cereals)  5. Foods High in Cholesterol:  * Egg yolks (limit to 4 per week)  * Organ meats (e.g., liver, kidney)  * High-cholesterol dairy products (e.g., cream, whole milk)  6. Processed and Packaged Foods:  * Frozen meals and TV dinners  * Canned goods with added salt and preservatives  * Boxed mixes and instant soups  7. Other Foods to Limit:  * Foods high in omega-6 fatty acids (e.g., corn oil, soybean oil)  * Foods with artificial sweeteners (e.g., aspartame, sucralose)  Remember, a heart-healthy diet is not just about avoiding certain foods, but also about incorporating nutrient-dense foods that can help lower your risk of heart disease. Focus on whole, unprocessed foods like:  * Fresh fruits and vegetables  * Whole grains (e.g., brown rice, quinoa, whole wheat)  * Lean protein sources (e.g., poultry, fish, legumes)  * Healthy fats (e.g., avocado, nuts, olive oil)  * Low-fat dairy products and plant-based alternatives  By making informed food choices, you can help protect your heart health and reduce your risk of heart disease. |
| **Llama 3 Answer 2** | On a heart-healthy diet, it's essential to limit or avoid foods that can increase your risk of heart disease, high blood pressure, and stroke. Here are some foods to limit or avoid:  1. Saturated and Trans Fats:  * Red meat (especially processed meats like bacon, sausage, and deli meats)  * Full-fat dairy products (cheese, whole milk, cream)  * Fried foods (french fries, fried chicken, doughnuts)  * Processed snacks (chips, crackers, cookies)  * Partially hydrogenated oils (trans fats)  2. Sodium-Rich Foods:  * Processed and packaged foods (canned soups, frozen meals, condiments)  * Salted snack foods (pretzels, popcorn, chips)  * Cured meats (hot dogs, ham, bacon)  * Sauces and condiments (soy sauce, teriyaki sauce, ketchup)  3. Added Sugars:  * Sugary drinks (soda, sports drinks, sweet tea, and coffee)  * Refined grains (white bread, sugary cereals, baked goods)  * Candy, cookies, cakes, and pastries  4. Refined Carbohydrates:  * White rice, pasta, and bread  * Sugary cereals and energy bars  5. Processed Meats:  * Hot dogs, sausages, bacon, and other processed meats  6. High-Cholesterol Foods:  * Egg yolks (limit to 4 per week)  * Organ meats (liver, kidney)  * High-cholesterol dairy products (cream, cheese, whole milk)  7. Foods High in Advanced Glycation End (AGE) Products:  * Fried foods, grilled meats, and processed meats  * Foods cooked at high temperatures (e.g., seared meat, roasted coffee)  Remember, moderation is key. It's not necessary to completely eliminate these foods, but try to limit your consumption and focus on whole, nutrient-dense foods like:  * Fresh fruits and vegetables  * Whole grains (brown rice, quinoa, whole-grain bread)  * Lean protein sources (poultry, fish, beans, lentils)  * Healthy fats (avocado, nuts, olive oil)  * Low-fat dairy products (skim milk, Greek yogurt)  By making these dietary changes, you'll be well on your way to a heart-healthy diet! |
| **Llama 3 Answer 3** | On a heart-healthy diet, it's essential to limit or avoid foods that can increase your risk of heart disease, high blood pressure, and high cholesterol. Here are some foods to limit or avoid:  1. Saturated and trans fats:  * Red meat (beef, pork, lamb)  * Full-fat dairy products (cheese, whole milk, cream)  * Processed meats (hot dogs, sausages, bacon)  * Fried foods (french fries, fried chicken, doughnuts)  * Baked goods (pastries, cakes, cookies)  * Partially hydrogenated oils (trans fats)  2. Refined carbohydrates:  * White bread and baked goods  * Sugary drinks (soda, sports drinks, sweet tea)  * Refined grains (white rice, pasta)  3. High-sodium foods:  * Processed meats (canned goods, sausages)  * Canned soups and broths  * Frozen meals (TV dinners, pizza)  * Sauces and condiments (soy sauce, teriyaki sauce)  4. Added sugars:  * Sugary drinks (soda, sports drinks, sweet tea)  * Refined grains (white bread, pasta)  * Baked goods (cakes, cookies, pastries)  * Candy and sweets  5. Foods high in cholesterol:  * Egg yolks (limit to 4 per week)  * Organ meats (liver, kidney)  * Shellfish (shrimp, lobster, crab)  6. Processed and packaged foods:  * Frozen meals (TV dinners, pizza)  * Canned goods (vegetables, meats)  * Boxed snacks (chips, crackers)  7. Other foods to limit:  * Caffeine (excessive consumption can increase heart rate and blood pressure)  * Foods high in advanced glycation end (AGE) products (e.g., grilled, fried, or processed meats)  Remember, a heart-healthy diet focuses on whole, unprocessed foods, such as:  * Fresh fruits and vegetables  * Whole grains (brown rice, quinoa, whole wheat)  * Lean protein sources (poultry, fish, legumes)  * Low-fat dairy products (skim milk, low-fat cheese)  * Healthy fats (avocado, nuts, olive oil)  By making informed food choices, you can help reduce your risk of heart disease and maintain a healthy heart. |
| **LLaMA3+RAG model Answer 1** | To maintain a heart-healthy diet, it's essential to avoid or limit certain foods that can increase the risk of heart disease. According to the American Heart Association, here are the foods you should avoid or limit:  1. Trans fat and partially hydrogenated oils: Found in commercially baked and fried foods, these substances can raise your risk of heart disease.  2. Whole milk and full-fat dairy products: Whole milk, full-fat cheeses, yogurt, and ice cream are high in saturated fats, which can increase your LDL-C (bad) cholesterol levels.  3. Milk substitutes with coconut oil, palm oil, or palm kernel oil: These oils are high in saturated fats, which can raise your LDL-C cholesterol levels and increase your risk of heart disease.  4. Processed foods: Limit your consumption of highly-processed items like:  Chips  Cookies  Cakes  Sugar cereals  Fast foods  Snack foods  5. High-sodium foods: Limit your daily sodium intake to no more than 2,300 milligrams, and ideally no more than 1,500 milligrams if you have high blood pressure. Avoid or limit foods that are high in sodium, such as:  Sandwiches  Burgers  Tacos  Rice, pasta, and grain dishes  Pizza  Meat, poultry, and seafood dishes  Soups  6. Foods high in added sugar: Be mindful of foods with high amounts of added sugar, such as:  Baked goods  Sweetened beverages  Candy  7. Foods high in saturated fats: Limit your daily saturated fat intake to less than 7% of your daily calories. Avoid or limit foods that are high in saturated fats, such as:  Red meat  Full-fat dairy products  Processed meats  Fried foods  To avoid unhealthy foods, follow these tips:  1. Limit processed foods and choose whole, fresh foods instead.  2. Read food labels and avoid items high in saturated fats, sodium, and added sugars.  3. Plan ahead and prepare healthy snacks and meals at home.  4. Choose lean meats, fish, seafood, and plant-based proteins.  5. Don't skip meals, as this can lead to unhealthy snacking.  By avoiding or limiting these foods and following these tips, you can maintain a heart-healthy diet and reduce your risk of heart disease.  Sources:  1. American Heart Association. (2024). How to Eat Healthy Without Dieting. Retrieved from <https://www.heart.org/en/healthy-living/healthy-eating/eat-smart/nutrition-basics/how-to-eat-healthy-without-dieting>  2. American Heart Association. (2024). Dairy Products, Milk, Yogurt, and Cheese. Retrieved from <https://www.heart.org/en/healthy-living/healthy-eating/eat-smart/nutrition-basics/dairy-products-milk-yogurt-and-cheese>  3. American Heart Association. (2024). Unhealthy Foods. Retrieved from <https://www.heart.org/en/healthy-living/go-red-get-fit/unhealthy-foods> |
| **LLaMA3+RAG model Answer 2** | To maintain a heart-healthy diet, it's essential to avoid certain foods that can increase the risk of heart disease and other cardiovascular conditions. Based on the American Heart Association's guidelines, here are the foods to limit or avoid:  1. Trans fat and partially hydrogenated oils: Found in commercially baked and fried foods, these substances can increase the risk of heart disease by raising LDL (bad) cholesterol levels.  2. Whole milk and full-fat dairy products: Whole milk, full-fat cheeses, yogurt, and ice cream are high in saturated fats, which can raise LDL cholesterol levels and increase the risk of heart disease.  3. Milk substitutes with coconut oil, palm oil, or palm kernel oil: These oils are high in saturated fats, which can raise LDL cholesterol levels and increase the risk of heart disease.  4. Highly-processed foods: Foods like chips, cookies, cakes, and sugar cereals are low in nutrients and high in empty calories, unhealthy fats, sodium, and sugar.  5. Foods high in sodium: Limit sodium intake to no more than 2,300 milligrams per day, and ideally no more than 1,500 milligrams daily for people with high blood pressure. Foods to limit or avoid include:  Sandwiches  Burgers  Tacos  Rice, pasta, and grain dishes  Pizza  Meat, poultry, and seafood dishes  Soups  6. Foods high in added sugar: Be mindful of foods with high sugar content, such as baked goods, sweetened beverages, and candy.  7. Foods high in saturated fats: Limit saturated fat intake to less than 7% of daily calories. Foods to limit or avoid include:  Red meat  Full-fat dairy products  Processed meats  Fried foods  Baked goods  To avoid unhealthy foods, follow these tips:  Limit processed foods  Read food labels carefully  Plan ahead and prepare healthy snacks and meals at home using whole, fresh foods  Choose lean meats, fish, seafood, and plant-based proteins  Don't skip meals to avoid snacking on unhealthy foods  By avoiding these foods and following the American Heart Association's guidelines, you can reduce your risk of heart disease and maintain a healthy diet.  Sources:  1. American Heart Association. (2024). How to Eat Healthy Without Dieting. Retrieved from <https://www.heart.org/en/healthy-living/healthy-eating/eat-smart/nutrition-basics/how-to-eat-healthy-without-dieting>  2. American Heart Association. (2024). Dairy Products, Milk, Yogurt, and Cheese. Retrieved from <https://www.heart.org/en/healthy-living/healthy-eating/eat-smart/nutrition-basics/dairy-products-milk-yogurt-and-cheese>  3. American Heart Association. (2024). Unhealthy Foods. Retrieved from <https://www.heart.org/en/healthy-living/go-red-get-fit/unhealthy-foods> |
| **LLaMA3+RAG model Answer 3** | To maintain a heart-healthy diet, it's essential to limit or avoid certain foods that can increase the risk of heart disease. Based on the American Heart Association's guidelines, here are some foods to avoid or limit:  1. Trans fat and partially hydrogenated oils: Found in commercially baked and fried foods, these unhealthy fats can increase the risk of heart disease.  2. Whole milk and full-fat dairy products: Whole milk, full-fat cheeses, yogurt, and ice cream are high in saturated fats, which can raise LDL-C (bad) cholesterol levels, increasing the risk of heart disease.  3. Milk substitutes with coconut oil, palm oil, or palm kernel oil: These oils are high in saturated fats, which can raise LDL-C cholesterol levels and increase the risk of heart disease.  4. Highly-processed foods: Foods like chips, cookies, cakes, and sugar cereals are low in nutrients and high in empty calories, unhealthy fats, sodium, and sugar.  5. Foods high in sodium: Limit foods that are high in sodium, such as sandwiches, burgers, tacos, rice, pasta and grain dishes, pizza, meat, poultry and seafood dishes, and soups. Aim to consume no more than 2,300 milligrams of sodium per day, with an ideal limit of no more than 1,500 milligrams daily for people with high blood pressure.  6. Foods high in added sugar: Limit foods and beverages with added sugars, such as sugary drinks, baked goods, and candy.  7. Foods high in saturated fats: Limit foods high in saturated fats, such as red meat, full-fat dairy products, and processed meats. Aim to keep saturated fat consumption to less than 7% of daily calories.  To avoid these unhealthy foods, follow these tips:  Limit processed foods and opt for whole, fresh foods like fruits and vegetables.  Read food labels and choose products low in saturated fats, sodium, and added sugars.  Plan ahead and prepare healthy snacks and meals at home.  Choose lean meats, fish, seafood, and plant-based proteins.  Don't skip meals to avoid snacking on unhealthy foods.  By avoiding or limiting these foods and following a heart-healthy diet, you can reduce your risk of heart disease and maintain optimal heart health.  Sources:  American Heart Association. (2024). How to Eat Healthy Without Dieting. Retrieved from <https://www.heart.org/en/healthy-living/healthy-eating/eat-smart/nutrition-basics/how-to-eat-healthy-without-dieting>  American Heart Association. (2024). Dairy Products, Milk, Yogurt, and Cheese. Retrieved from <https://www.heart.org/en/healthy-living/healthy-eating/eat-smart/nutrition-basics/dairy-products-milk-yogurt-and-cheese>  American Heart Association. (2024). Unhealthy Foods. Retrieved from <https://www.heart.org/en/healthy-living/go-red-get-fit/unhealthy-foods> |
| **Question 17** | **What diet should I follow to lower cholesterol?** |
| **Perplexity Answer 1** | To lower your cholesterol through diet, you should:  1. Choose healthier fats. Limit saturated fat to less than 7% of your daily calories. Avoid trans fats. Instead, use olive, canola and other vegetable oils[1][4].  2. Eat plenty of soluble fiber. Good sources include oats, barley, beans, lentils, apples, bananas, oranges, pears, and prunes[1][2][3]. Aim for 20-35 grams of fiber per day, with at least 5-10 grams from soluble fiber[2].  3. Eat more fruits, vegetables, and whole grains. These foods are rich in soluble fiber and plant sterols that can help lower LDL cholesterol[1][3]. Vegetables like eggplant and okra are especially high in soluble fiber[2].  4. Choose lean protein, especially plant-based proteins. Eat more fish, poultry, beans, lentils, nuts and seeds. Limit red meat to 1-3 times per week[3][5].  5. Limit cholesterol from egg yolks, organ meats, shrimp and whole milk dairy. Aim for less than 200mg per day[1].  6. Eat 2 servings of fatty fish per week for heart-healthy omega-3 fats. Good choices include salmon, tuna and mackerel[1].  7. Limit salt to no more than 2,300mg per day. Avoid processed foods and choose low-sodium options[1].  8. If you drink alcohol, limit to no more than 1 drink per day for women and 2 for men. Alcohol is high in calories and can raise triglycerides[1].  Following a Mediterranean-style diet that emphasizes these foods is an effective way to lower LDL cholesterol and improve heart health[4]. Meeting with a dietitian can provide personalized guidance on the best diet for you.  Citations:  [1] https://medlineplus.gov/howtolowercholesterolwithdiet.html  [2] https://www.health.harvard.edu/heart-health/11-foods-that-lower-cholesterol  [3] https://www.betterhealth.vic.gov.au/health/conditionsandtreatments/cholesterol-healthy-eating-tips  [4] https://my.clevelandclinic.org/health/articles/16867-cholesterol--nutrition-tlc  [5] https://www.heart.org/en/health-topics/cholesterol/prevention-and-treatment-of-high-cholesterol-hyperlipidemia/cooking-to-lower-cholesterol |
| **Perplexity Answer 2** | To lower your cholesterol through diet, you should:  - Choose healthier fats. Limit saturated fat to less than 7% of your daily calories and avoid trans fat. Instead, use healthy unsaturated oils like olive, canola, safflower or sunflower oil[1][4].  - Eat plenty of soluble fiber from foods like oats, barley, beans, eggplant, okra, apples, bananas, oranges, pears and prunes. Soluble fiber helps prevent cholesterol absorption[1][2][3].  - Eat 2 servings of fish high in omega-3 fatty acids like salmon, tuna or mackerel each week. Omega-3s may help raise HDL (good) cholesterol[1].  - Include a variety of fruits, vegetables, whole grains, legumes, nuts and seeds in your diet. These foods are high in fiber and plant sterols which can help lower LDL (bad) cholesterol[2][3].  - Limit foods high in dietary cholesterol like organ meats, egg yolks, shrimp and whole milk dairy products to less than 200mg per day[1][4].  - Limit alcohol to no more than 2 drinks per day for men and 1 drink per day for women[1].  - Maintain a healthy weight and be physically active[3].  The Mediterranean diet, which emphasizes plant-based foods, healthy fats, and limits red and processed meats, is a heart-healthy eating pattern that can help lower cholesterol[4][5]. Cooking at home allows you to control ingredients and portion sizes[4].  Citations:  [1] https://medlineplus.gov/howtolowercholesterolwithdiet.html  [2] https://www.health.harvard.edu/heart-health/11-foods-that-lower-cholesterol  [3] https://www.betterhealth.vic.gov.au/health/conditionsandtreatments/cholesterol-healthy-eating-tips  [4] https://my.clevelandclinic.org/health/articles/16867-cholesterol--nutrition-tlc  [5] https://www.heart.org/en/health-topics/cholesterol/prevention-and-treatment-of-high-cholesterol-hyperlipidemia/cooking-to-lower-cholesterol |
| **Perplexity Answer 3** | To lower your cholesterol through diet, you should:  - Choose healthier fats. Limit saturated fat to less than 7% of your daily calories and avoid trans fat. Instead, use olive oil, canola oil, nuts, and seeds which contain healthy unsaturated fats[1][2].  - Eat plenty of soluble fiber from foods like oats, barley, beans, eggplant, okra, and berries. Soluble fiber binds to cholesterol and removes it from the body[1][2].  - Eat more fruits, vegetables, and whole grains. A diet rich in these provides important cholesterol-lowering compounds[1][3].  - Choose lean protein sources like fish, poultry, legumes, and soy. Limit red meat and processed meats[4][5].  - Limit foods high in cholesterol like organ meats, egg yolks, and full-fat dairy[1][4].  - Limit alcohol, which can raise triglycerides and LDL cholesterol[1][3].  The Mediterranean diet, which emphasizes plant-based foods, healthy fats, and lean proteins, is an excellent eating pattern for lowering cholesterol and heart disease risk[4]. Portion control is also important, as excess calories can lead to weight gain which negatively impacts cholesterol levels[3].  Citations:  [1] https://medlineplus.gov/howtolowercholesterolwithdiet.html  [2] https://www.health.harvard.edu/heart-health/11-foods-that-lower-cholesterol  [3] https://www.betterhealth.vic.gov.au/health/conditionsandtreatments/cholesterol-healthy-eating-tips  [4] https://my.clevelandclinic.org/health/articles/16867-cholesterol--nutrition-tlc  [5] https://www.heart.org/en/health-topics/cholesterol/prevention-and-treatment-of-high-cholesterol-hyperlipidemia/cooking-to-lower-cholesterol |
| **GPT4o Answer 1** | To lower cholesterol, you should follow a heart-healthy diet that focuses on reducing intake of saturated and trans fats, while increasing fiber and healthy fats. Here are some dietary guidelines to help you lower your cholesterol:  Foods to Include:  1. Fruits and Vegetables:  - Aim for at least five servings per day. They are rich in fiber and antioxidants.  - Examples: Apples, oranges, bananas, berries, leafy greens, and cruciferous vegetables like broccoli and Brussels sprouts.  2. Whole Grains:  - Choose whole grains over refined grains. They are high in fiber, which can help lower cholesterol.  - Examples: Oats, brown rice, whole wheat, quinoa, barley, and whole grain bread and pasta.  3. Healthy Fats:  - Focus on monounsaturated and polyunsaturated fats. These fats can help reduce LDL (bad) cholesterol.  - Examples: Olive oil, avocados, nuts, seeds, and fatty fish like salmon, mackerel, and sardines.  4. Legumes:  - Beans, lentils, and chickpeas are good sources of plant-based protein and fiber.  - Examples: Black beans, kidney beans, lentils, and chickpeas.  5. Fiber-Rich Foods:  - Soluble fiber can help reduce the absorption of cholesterol into your bloodstream.  - Examples: Oats, beans, flaxseeds, psyllium, and fruits like apples and pears.  Foods to Limit or Avoid:  1. Saturated Fats:  - Found in red meat, full-fat dairy products, butter, and tropical oils like coconut and palm oil.  - Choose lean cuts of meat and low-fat or fat-free dairy products.  2. Trans Fats:  - Often found in fried foods, baked goods, and processed snacks.  - Avoid foods with ""partially hydrogenated oils"" on the label.  3. Cholesterol-Rich Foods:  - Limit intake of high-cholesterol foods like organ meats, shellfish, and egg yolks.  4. Refined Carbohydrates and Sugary Foods:  - Reduce consumption of white bread, pasta, pastries, and sugary beverages.  - Choose whole grain options and limit added sugars.  Additional Tips:  - Eat More Plant-Based Meals:  - Consider incorporating more plant-based meals into your diet to reduce intake of animal fats.  - Examples: Vegetable stir-fry, lentil soup, or bean-based chili.  - Use Healthy Cooking Methods:  - Opt for baking, steaming, grilling, or broiling instead of frying.  - Stay Hydrated:  - Drink plenty of water and limit sugary drinks and alcohol.  - Exercise Regularly:  - Combine a healthy diet with regular physical activity. Aim for at least 30 minutes of moderate exercise most days of the week.  - Read Food Labels:  - Check nutrition labels for saturated fat, trans fat, and cholesterol content.  Consult with a healthcare professional or a registered dietitian for personalized advice tailored to your specific health needs and conditions. |
| **GPT4o Answer 2** | To lower cholesterol, following a heart-healthy diet is key. Here are some guidelines you can follow:  1. Reduce Saturated Fats  - Sources: Red meat, full-fat dairy products, fried foods, and commercially baked products.  - Alternatives: Lean meats, low-fat dairy, and plant-based oils (olive oil, canola oil).  2. Eliminate Trans Fats  - Sources: Partially hydrogenated oils, many margarines, store-bought cookies, crackers, and cakes.  - Tip: Check food labels and avoid anything that contains partially hydrogenated oils.  3. Increase Soluble Fiber Intake  - Sources: Oats, barley, fruits (like apples, oranges, and pears), vegetables, legumes (like beans and lentils).  - Effect: Helps reduce the absorption of cholesterol into your bloodstream.  4. Eat More Omega-3 Fatty Acids  - Sources: Fatty fish (salmon, mackerel, sardines), flaxseeds, chia seeds, walnuts.  - Benefits: They don’t affect LDL cholesterol but have heart health benefits, including reducing blood pressure.  5. Include Plant Sterols and Stanols  - Sources: Fortified foods (certain margarines, orange juice, and yogurt drinks), nuts, seeds.  - Benefits: They help block the absorption of cholesterol.  6. Consume More Fruits and Vegetables  - Recommendation: Aim for at least five servings a day.  - Benefits: They are high in dietary fiber, antioxidants, and help lower cholesterol.  7. Opt for Whole Grains  - Sources: Whole wheat, brown rice, oats, quinoa, barley.  - Benefits: They are rich in fiber and help in lowering cholesterol levels.  8. Limit Dietary Cholesterol  - Sources: Animal products such as meat, poultry, dairy, and egg yolks.  - Recommendation: Keep your dietary cholesterol intake below 200 mg per day.  9. Moderate Alcohol Intake  - Guideline: Up to one drink a day for women and two drinks a day for men.  - Note: Excess alcohol can raise cholesterol and triglyceride levels.  10. Maintain a Healthy Weight  - Strategy: Combine diet with regular physical activity.  - Benefits: Weight loss can help lower cholesterol levels and improve heart health.  Sample Diet Plan  Breakfast  - Oatmeal with fresh berries and a sprinkle of flaxseeds  - A glass of orange juice fortified with plant sterols  Lunch  - Grilled salmon salad with mixed greens, cherry tomatoes, cucumbers, and a vinaigrette dressing  - A serving of whole grain bread  Snack  - An apple or a handful of almonds  Dinner  - Quinoa and vegetable stir-fry with tofu  - Steamed broccoli  Dessert  - A bowl of fresh fruit or a small serving of low-fat yogurt  Incorporate these guidelines into your daily eating habits to effectively manage and lower your cholesterol levels. Additionally, regular physical activity and avoiding smoking can significantly enhance the benefits of a cholesterol-lowering diet. If you have specific health conditions or dietary restrictions, it's best to consult with a healthcare provider or a registered dietitian for personalized advice. |
| **GPT4o Answer 3** | To lower cholesterol, a heart-healthy diet is recommended. Here are key dietary guidelines to help you achieve this:  1. Increase Fiber Intake:  - Eat more fruits, vegetables, whole grains, and legumes.  - Soluble fiber, found in foods like oats, barley, beans, lentils, fruits, and vegetables, can help reduce cholesterol absorption.  2. Choose Healthy Fats:  - Replace saturated fats (found in red meat, butter, cheese, and other full-fat dairy products) with healthier fats such as monounsaturated and polyunsaturated fats (found in olive oil, avocados, nuts, and fatty fish).  - Avoid trans fats, often found in fried and commercially baked products.  3. Eat More Omega-3 Fatty Acids:  - Include fatty fish in your diet at least twice a week. Good options include salmon, mackerel, sardines, and albacore tuna.  - Plant sources include flaxseeds, chia seeds, and walnuts.  4. Limit Dietary Cholesterol:  - Reduce intake of high-cholesterol foods such as organ meats, shellfish, and full-fat dairy products.  5. Increase Plant Sterols and Stanols:  - Consume foods fortified with sterols and stanols, such as certain margarines, orange juice, and yogurt drinks. These can help block the absorption of cholesterol.  6. Reduce Sodium Intake:  - Limit salt and high-sodium foods. Aim for less than 2,300 milligrams of sodium per day, and ideally reduce it to 1,500 milligrams for better heart health.  7. Limit Sugar and Refined Carbohydrates:  - Cut back on sugary beverages, sweets, and refined grains. Opt for whole grains and low-glycemic index foods instead.  8. Drink Alcohol in Moderation:  - If you drink alcohol, do so in moderation. That means up to one drink per day for women and up to two drinks per day for men.  9. Maintain a Healthy Weight:  - Being overweight or obese can raise your cholesterol levels. Losing even a small amount of weight can help.  10. Stay Hydrated:  - Drink plenty of water throughout the day.  Sample Diet Plan  Breakfast:  - Oatmeal topped with fresh berries and a handful of nuts.  - A glass of orange juice fortified with plant sterols.  Mid-Morning Snack:  - An apple or a pear.  Lunch:  - Grilled salmon salad with mixed greens, cherry tomatoes, cucumber, and olive oil vinaigrette.  - A whole grain roll.  Afternoon Snack:  - Carrot sticks with hummus.  Dinner:  - Quinoa and black bean stuffed bell peppers.  - Steamed broccoli and a side salad with avocado.  Evening Snack:  - A handful of walnuts or a small bowl of mixed berries.  Additional Tips  - Cooking methods: Opt for grilling, baking, steaming, and broiling over frying.  - Read food labels: Look for low-saturated fat, low-sodium, and no trans fat options.  Always consult with a healthcare professional or a registered dietitian before making significant changes to your diet, especially if you have underlying health conditions. |
| **Llama3 Answer 1** | Lowering cholesterol levels can be achieved through a combination of dietary changes, lifestyle modifications, and if necessary, medication. Here's a comprehensive guide to help you get started with a cholesterol-lowering diet:  Dietary Recommendations:  1. Increase soluble fiber intake: Soluble fiber helps lower LDL (bad) cholesterol. Aim for 25-30 grams of soluble fiber per day from foods like:  * Oatmeal, oat bran, and barley  * Fruits (especially apples, bananas, and berries)  * Vegetables (especially carrots, Brussels sprouts, and broccoli)  * Legumes (such as beans, lentils, and peas)  2. Eat more plant-based protein: Plant-based protein sources tend to be lower in saturated fat and cholesterol than animal-based sources. Include:  * Legumes (mentioned above)  * Nuts and seeds (almonds, walnuts, chia seeds, and flaxseeds)  * Whole grains (quinoa, brown rice, and whole wheat)  3. Choose healthy fats: Focus on unsaturated fats, which can help lower LDL cholesterol and increase HDL (good) cholesterol:  * Avocados  * Fatty fish (salmon, tuna, and mackerel)  * Nuts and seeds (mentioned above)  * Olive oil  4. Limit or avoid:  * Saturated and trans fats: Found in red meat, full-fat dairy products, processed meats, and fried foods.  * Refined carbohydrates: Limit sugary drinks, white bread, and sweets.  * High-sodium foods: Consume no more than 2,300 milligrams of sodium per day.  5. Incorporate cholesterol-lowering foods: Certain foods have been shown to have a positive impact on cholesterol levels:  * Tea (especially green tea)  * Dark chocolate (70% cocoa or higher)  * Fermented foods (like kimchi, sauerkraut, and yogurt)  Additional Tips:  1. Maintain a healthy weight: Excess weight, particularly around the waistline, can contribute to high cholesterol.  2. Exercise regularly: Aim for at least 150 minutes of moderate-intensity aerobic exercise or 75 minutes of vigorous-intensity aerobic exercise per week.  3. Limit dietary cholesterol: Consume no more than 300 milligrams of dietary cholesterol per day. Foods high in cholesterol include egg yolks, organ meats, and high-cholesterol dairy products.  4. Stay hydrated: Drink plenty of water and limit sugary drinks.  5. Consider consulting a registered dietitian or healthcare professional: They can help you develop a personalized diet plan based on your individual needs and health goals.  Sample Meal Ideas:  Breakfast:  * Oatmeal with banana, almond butter, and a splash of low-fat milk  * Avocado toast on whole-grain bread with scrambled eggs and a side of berries  Lunch:  * Grilled chicken breast with roasted vegetables and quinoa  * Lentil soup with whole-grain bread and a side salad  Dinner:  * Baked salmon with brown rice, steamed broccoli, and a side of sautéed spinach  * Grilled turkey burger on a whole-grain bun with avocado, lettuce, and tomato  Remember, a comprehensive approach to lowering cholesterol involves a combination of dietary changes, regular exercise, and, if necessary, medication. Consult with your healthcare provider before making any significant changes to your diet or exercise routine. |
| **Llama3 Answer 2** | A diet to lower cholesterol typically involves making healthy food choices and limiting certain nutrients that can raise cholesterol levels. Here are some general dietary recommendations to help lower cholesterol:  Increase consumption of:  1. Soluble fiber: Foods high in soluble fiber, such as oats, barley, fruits (especially apples, berries, and pears), vegetables (like carrots, brussels sprouts, and broccoli), and legumes (like beans, lentils, and peas), can help lower LDL (bad) cholesterol.  2. Plant-based protein: Choose plant-based protein sources like beans, lentils, tofu, tempeh, and seitan, which are low in saturated fat and cholesterol.  3. Fatty fish: Fatty fish like salmon, mackerel, and sardines are rich in omega-3 fatty acids, which can help lower triglycerides and improve overall heart health.  4. Avocados: Rich in monounsaturated fats, avocados can help lower LDL cholesterol and increase HDL (good) cholesterol.  5. Nuts and seeds: Almonds, walnuts, flaxseeds, and chia seeds are rich in healthy fats, fiber, and antioxidants, which can help lower cholesterol.  6. Foods fortified with plant sterols and stanols: These natural substances, found in fortified foods like margarine and spreads, can help lower cholesterol.  Limit or avoid:  1. Saturated and trans fats: Limit foods high in saturated fats, like:  * Red meat (beef, pork, lamb)  * Full-fat dairy products (cheese, milk, cream)  * Processed meats (hot dogs, sausages, bacon)  * Fried foods and baked goods  * Partially hydrogenated oils (trans fats)  2. Cholesterol-rich foods: Limit or avoid foods high in cholesterol, such as:  * Egg yolks  * Organ meats (l!ver, kidney)  * High-cholesterol dairy products (cream, cheese)  3. Refined carbohydrates: Limit or avoid refined carbohydrates, such as:  * White bread  * Sugary drinks  * Refined grains (white rice, pasta)  Dietary patterns:  1. Mediterranean Diet: Emphasizes whole grains, fruits, vegetables, lean protein, and healthy fats, such as those found in olive oil.  2. Portfolio Diet: A plant-based diet that combines soluble fiber, soy protein, and plant sterols to help lower cholesterol.  3. DASH Diet: Focuses on whole grains, fruits, vegetables, lean protein, and low-fat dairy products to help lower blood pressure and cholesterol.  Additional tips:  1. Maintain a healthy weight: Excess weight can increase cholesterol levels, so aim for a healthy weight through a combination of diet and exercise.  2. Limit dietary cholesterol: Consume no more than 300 milligrams of dietary cholesterol per day.  3. Choose healthy oils: Use heart-healthy oils like olive, canola, or avocado oil for cooking and dressings.  4. Stay hydrated: Drink plenty of water and limit sugary drinks.  5. Consult a healthcare professional: Work with a registered dietitian, nutritionist, or healthcare provider to develop a personalized diet plan tailored to your specific needs and health goals.  Remember, a healthy diet is just one aspect of managing cholesterol levels. Regular exercise, stress management, and, if necessary, medication can also play important roles in maintaining healthy cholesterol levels. |
| **Llama3 Answer 3** | Lowering cholesterol levels can be achieved through a combination of dietary changes, lifestyle modifications, and, if necessary, medication. Here's a comprehensive guide to help you get started:  Dietary Recommendations:  1. Increase Soluble Fiber Intake: Foods high in soluble fiber can help lower LDL (bad) cholesterol. Aim for 25-30 grams of soluble fiber per day from sources like:  * Oats  * Barley  * Fruits (especially apples, berries, and pears)  * Vegetables (like broccoli, carrots, and brussels sprouts)  * Legumes (such as beans, lentils, and peas)  2. Choose Healthy Fats: Focus on unsaturated fats, which can help lower LDL cholesterol and increase HDL (good) cholesterol:  * Avocados  * Nuts (almonds, walnuts, and pecans)  * Fatty fish (like salmon, mackerel, and sardines)  * Olive oil  3. Select Lean Protein Sources: Opt for lean protein sources, which can help lower saturated fat intake:  * Poultry (chicken, turkey)  * Fish (especially fatty fish)  * Legumes  * Low-fat dairy products  4. Limit Saturated and Trans Fats: Restrict or avoid foods high in saturated and trans fats, which can raise LDL cholesterol:  * Red meat  * Full-fat dairy products  * Processed meats (like hot dogs, sausages, and bacon)  * Fried foods  * Baked goods and sweets  5. Watch Cholesterol-Rich Foods: Limit or avoid foods high in cholesterol:  * Egg yolks  * Organ meats (like liver and kidney)  * Shellfish (like shrimp and lobster)  Additional Tips:  1. Maintain a Healthy Weight: Excess weight can contribute to high cholesterol, so aim for a healthy weight through a combination of diet and exercise.  2. Limit Dietary Cholesterol: Consume no more than 300 milligrams of dietary cholesterol per day.  3. Incorporate Plant-Based Meals: Plant-based diets tend to be lower in saturated fat and higher in fiber, which can help lower cholesterol.  4. Stay Hydrated: Drink plenty of water and limit sugary drinks.  5. Limit Sodium Intake: Excessive sodium consumption can increase blood pressure, which can contribute to high cholesterol. Aim for no more than 2,300 milligrams of sodium per day.  Sample Meal Ideas:  * Oatmeal with banana, almond butter, and low-fat milk  * Grilled chicken breast with roasted vegetables and quinoa  * Lentil soup with whole-grain bread and a side salad  * Baked salmon with brown rice and steamed broccoli  Remember:  * Always consult with your healthcare provider or a registered dietitian before making significant changes to your diet.  * Combine dietary changes with regular exercise, stress management, and, if necessary, medication to effectively lower cholesterol levels.  By following these guidelines, you'll be well on your way to reducing your cholesterol levels and improving your overall heart health. |
| **LLaMA3+RAG model Answer 1** | To lower cholesterol, it is recommended to follow a diet that replaces saturated fats with unsaturated fats as part of a healthy eating pattern. This can be achieved by incorporating the following foods into your diet:  1. Fatty fish: Eat at least two 3.5- to 4-ounce servings of non-fried fatty fish per week, such as albacore tuna, herring, lake trout, mackerel, sardines, and salmon. These fish are rich in omega-3 fatty acids, which can help lower bad cholesterol and triglyceride levels.  2. Nuts and seeds: Munch on a small handful (about 1 ounce) of unsalted nuts and seeds daily, such as almonds, hazelnuts, peanuts, pecans, pistachios, pumpkin seeds, sunflower seeds, and walnuts. These foods are rich in good fats, energy, protein, and fiber.  3. Avocado: Incorporate avocado into your diet by snacking, cooking, and baking with it. Avocado is a rich source of healthy fats, fiber, and essential vitamins and minerals.  4. Healthy oils: Use nontropical vegetable oils that are lower in saturated fat, such as avocado oil, canola oil, corn oil, grapeseed oil, olive oil, peanut oil, safflower oil, sesame oil, soybean oil, and sunflower oil.  In addition to incorporating these foods into your diet, it is also important to limit or avoid foods that are high in saturated and trans fats, such as:  Red meat and full-fat dairy products  Processed and packaged foods, such as baked goods, snacks, and fried foods  Partially hydrogenated oils, such as partially hydrogenated soybean oil and partially hydrogenated corn oil  By following this dietary pattern, you can help lower your cholesterol levels and reduce your risk of heart disease.  Sources:  American Heart Association. (2024). 4 Ways to Get Good Fats Infographic. Retrieved from <https://www.heart.org/en/healthy-living/healthy-eating/eat-smart/fats/4-ways-to-get-good-fats-infographic> |
| **LLaMA3+RAG model Answer 2** | To lower cholesterol, it is essential to focus on a diet that replaces saturated fats with unsaturated fats. The American Heart Association recommends incorporating the following foods into your diet to achieve this goal:  1. Fatty Fish: Eat at least two 3.5- to 4-ounce servings of non-fried fatty fish per week, such as albacore tuna, herring, lake trout, mackerel, sardines, and salmon. These fish are rich in omega-3 fatty acids, which can help lower bad cholesterol and triglyceride levels.  2. Unsalted Nuts and Seeds: Munch on a small handful (about 1 ounce) of unsalted nuts and seeds daily, including almonds, hazelnuts, peanuts, pecans, pistachios, pumpkin seeds, sunflower seeds, and walnuts. These snacks provide good fats, energy, protein, and fiber.  3. Avocado: Incorporate avocado into your diet by snacking, cooking, and baking with it. Avocados are a rich source of healthy fats, fiber, and essential vitamins and minerals.  4. Nontropical Vegetable Oils: Use nontropical vegetable oils that are lower in saturated fat, such as avocado oil, canola oil, corn oil, grapeseed oil, olive oil, peanut oil, safflower oil, sesame oil, soybean oil, and sunflower oil. These oils can help replace saturated fats in your diet.  In addition to incorporating these foods into your diet, it is also essential to limit or avoid foods that are high in saturated and trans fats, such as:  Red meat  Full-fat dairy products  Processed meats  Fried foods  Baked goods  Partially hydrogenated oils  By following this dietary pattern, you can help lower your cholesterol levels and reduce your risk of heart disease.  Sources:  American Heart Association. (2024). 4 Ways to Get Good Fats Infographic. Retrieved from <https://www.heart.org/en/healthy-living/healthy-eating/eat-smart/fats/4-ways-to-get-good-fats-infographic> |
| **LLaMA3+RAG model Answer 3** | To lower cholesterol, it's essential to follow a diet that focuses on replacing saturated fats with unsaturated fats as part of a healthy eating pattern. Here's a comprehensive dietary plan to help you achieve this goal:  Incorporate Fatty Fish: Eat at least two 3.5- to 4-ounce servings of non-fried fatty fish per week. Fatty fish like albacore tuna, herring, lake trout, mackerel, sardines, and salmon are rich in essential omega-3 fatty acids, which can help lower bad cholesterol and triglyceride levels.  Nuts and Seeds: Munch on a small handful (about 1 ounce) of unsalted nuts and seeds daily to get a boost of good fats, energy, protein, and fiber. Almonds, hazelnuts, peanuts, pecans, pistachios, pumpkin seeds, sunflower seeds, and walnuts are all excellent choices.  Avocado: Snack, cook, and bake with avocado to add healthy fats, fiber, and essential vitamins and minerals to your diet. Avocados are a rich source of monounsaturated fats, which can help lower bad cholesterol levels.  Healthy Oils: Use non-tropical vegetable oils that are lower in saturated fat. Opt for avocado oil, canola oil, corn oil, grapeseed oil, olive oil, peanut oil, safflower oil, sesame oil, soybean oil, and sunflower oil for cooking, baking, and making salad dressings.  Additional Tips:  Limit or avoid saturated and trans fats, often found in processed and fried foods, red meat, full-fat dairy products, and partially hydrogenated oils.  Choose lean protein sources like poultry, fish, and plant-based options like beans, lentils, and tofu.  Incorporate soluble fiber-rich foods like oats, barley, nuts, seeds, and fruits (especially apples, berries, and pears) into your diet to help lower cholesterol levels.  Limit dietary cholesterol by avoiding or limiting foods high in cholesterol, such as egg yolks, organ meats, and high-cholesterol dairy products.  By following this dietary plan, you'll be well on your way to reducing your cholesterol levels and promoting overall heart health.  Sources:  American Heart Association. (2024). 4 Ways to Get Good Fats Infographic. Retrieved from <https://www.heart.org/en/healthy-living/healthy-eating/eat-smart/fats/4-ways-to-get-good-fats-infographic>  American Heart Association. (2017). Healthy Eating Plate. Retrieved from <https://www.heart.org/en/healthy-living/healthy-eating/eat-smart/nutrition-basics/healthy-eating-plate> |
| **Question 18** | **Can I have dairy on a heart healthy diet?** |
| **Perplexity Answer 1** | Yes, dairy can be part of a heart-healthy diet. While dairy products contain saturated fat, which was previously thought to increase heart disease risk, newer research suggests that dairy consumption, including full-fat varieties, is not associated with an increased risk of heart disease or stroke[1][3][5].  In fact, consuming at least two servings of dairy per day, such as milk, yogurt, or cheese, has been linked to a lower risk of heart disease, stroke, and heart-related death[4]. Fermented dairy foods like yogurt and cheese may even help reduce weight gain, body fat, and lower the risk of cardiovascular disease[5].  The American Heart Association, the Dietary Guidelines for Americans, and the DASH Diet all recommend including dairy as part of a heart-healthy diet, along with fruits, vegetables, whole grains, lean protein, and healthy oils[1]. To reap the heart-health benefits of dairy, aim for at least 3 servings each day[1].  When choosing dairy products, a variety of options, including whole and reduced-fat varieties, can be part of a balanced, heart-smart diet[1][2]. Focus on plain, unsweetened dairy and pair it with other healthy foods like fresh fruit, whole grain crackers, or salads[2].  Citations:  [1] https://www.americandairy.com/health-wellness/benefits-of-dairy/heart-health-and-dairy/  [2] https://www.health.harvard.edu/heart-health/whats-the-deal-with-dairy-and-heart-health  [3] https://www.webmd.com/diet/news/20230711/whole-fat-dairy-ok-for-a-heart-healthy-diet  [4] https://www.health.harvard.edu/heart-health/two-daily-servings-of-dairy-good-for-your-heart  [5] https://mcpress.mayoclinic.org/dairy-health/full-fat-dairy-foods-and-cardiovascular-disease-is-there-a-connection/ |
| **Perplexity Answer 2** | Yes, dairy can be part of a heart-healthy diet, including full-fat dairy products in moderation[1][2][3][4][5]. Here are the key points:  - Dairy foods like milk, yogurt, and cheese are good sources of protein, calcium, potassium, and other nutrients important for heart health[2][4].  - Consuming at least 2-3 servings of dairy per day is linked to a lower risk of heart disease, stroke, and death compared to not eating dairy[4].  - While dairy contains saturated fat which can raise LDL cholesterol, newer research suggests full-fat dairy may not negatively impact heart disease risk as previously thought[1][3][5].  - Replacing dairy fat with refined carbs and sugars is worse for heart health than including moderate amounts of full-fat dairy[1].  - The American Heart Association, DASH diet, and Dietary Guidelines recommend dairy as part of a heart-healthy diet[2].  - Experts advise focusing on increasing protective foods like dairy, fruits, vegetables, nuts, legumes and fish rather than restricting full-fat dairy[3].  So in moderation, both low-fat and full-fat dairy can be part of an overall nutritious diet to support heart health. The key is to enjoy dairy with other healthy foods and limit saturated fat from other sources[1][2][3][4][5].  Citations:  [1] https://www.health.harvard.edu/heart-health/whats-the-deal-with-dairy-and-heart-health  [2] https://www.americandairy.com/health-wellness/benefits-of-dairy/heart-health-and-dairy/  [3] https://www.webmd.com/diet/news/20230711/whole-fat-dairy-ok-for-a-heart-healthy-diet  [4] https://www.health.harvard.edu/heart-health/two-daily-servings-of-dairy-good-for-your-heart  [5] https://mcpress.mayoclinic.org/dairy-health/full-fat-dairy-foods-and-cardiovascular-disease-is-there-a-connection/ |
| **Perplexity Answer 3** | Yes, dairy can be part of a heart-healthy diet, including full-fat dairy products in moderation[1][2][3][4][5]. Here are the key points:  - Dairy foods like milk, yogurt, and cheese are good sources of essential nutrients for heart health, including calcium, potassium, vitamin D, and protein[2][4].  - Consuming at least 2-3 servings of dairy per day is linked to a lower risk of heart disease, stroke, and mortality compared to little or no dairy intake[4][5].  - While dairy products contain saturated fat, which can raise LDL cholesterol, newer research suggests full-fat dairy may not have the negative effects on heart health that were previously assumed[1][3][5].  - Replacing dairy fat with refined carbs or sugary foods is worse for heart health than enjoying dairy in moderation as part of an overall healthy diet[1].  - The American Heart Association, DASH diet, and Dietary Guidelines for Americans all recommend dairy as part of a heart-healthy eating pattern[2].  The take-home message is to enjoy a variety of dairy products, including full-fat options in moderation, as part of an overall diet rich in fruits, vegetables, whole grains, lean protein, and healthy fats to support heart health[3][5].  Citations:  [1] https://www.health.harvard.edu/heart-health/whats-the-deal-with-dairy-and-heart-health  [2] https://www.americandairy.com/health-wellness/benefits-of-dairy/heart-health-and-dairy/  [3] https://www.webmd.com/diet/news/20230711/whole-fat-dairy-ok-for-a-heart-healthy-diet  [4] https://www.health.harvard.edu/heart-health/two-daily-servings-of-dairy-good-for-your-heart  [5] https://mcpress.mayoclinic.org/dairy-health/full-fat-dairy-foods-and-cardiovascular-disease-is-there-a-connection/ |
| **GPT4o Answer 1** | Yes, you can have dairy on a heart-healthy diet, but it's important to choose the right types and consume them in moderation. Here are some guidelines for incorporating dairy into a heart-healthy diet:  1. Choose Low-Fat or Fat-Free Options: Opt for low-fat or fat-free versions of milk, yogurt, and cheese. These options have less saturated fat, which is better for heart health.  2. Watch Portions: Dairy products can be high in calories and fat, so it's important to consume them in moderation. Stick to recommended serving sizes.  3. Avoid High-Fat Dairy: Limit or avoid full-fat dairy products, as they are higher in saturated fats, which can raise cholesterol levels.  4. Select Fermented Dairy Products: Yogurt and kefir can be good choices because they contain probiotics, which can be beneficial for gut health. Just be sure to choose low-fat or fat-free versions without added sugars.  5. Limit Added Sugars: Choose plain dairy products or those with no added sugars. Flavored yogurts, for example, can have a lot of added sugars, which are not heart-healthy.  6. Consider Plant-Based Alternatives: If you're looking to reduce your dairy intake, consider plant-based alternatives such as almond milk, soy milk, or oat milk. Choose versions that are fortified with calcium and vitamin D and do not contain added sugars.  7. Monitor Sodium Intake: Some dairy products, like cheese, can be high in sodium. Choose lower-sodium options when available and be mindful of your overall sodium intake.  Remember, a heart-healthy diet is about balance and variety. Including dairy in moderation, along with plenty of fruits, vegetables, whole grains, lean proteins, and healthy fats, can help you maintain a heart-healthy diet. |
| **GPT4o Answer 2** | Yes, you can include dairy in a heart-healthy diet, but it's important to choose the right types and amounts. Here are some guidelines to follow:  1. Choose Low-Fat or Fat-Free Options: Opt for low-fat or fat-free milk, yogurt, and cheese. These options contain less saturated fat, which is better for heart health.  2. Watch Portion Sizes: Even low-fat and fat-free dairy products can be high in calories, so it's important to consume them in moderation.  3. Consider Alternatives: Plant-based milk alternatives like almond, soy, or oat milk can be good options, especially if they are fortified with calcium and vitamin D. Make sure to choose unsweetened versions to avoid added sugars.  4. Limit High-Fat Dairy: Full-fat dairy products contain more saturated fat, which can raise LDL cholesterol levels. If you choose to consume these, do so sparingly.  5. Check Labels for Added Sugars: Some flavored yogurts and milk can have added sugars. Opt for plain versions and add your own fruit or a small amount of honey for sweetness.  6. Incorporate Fermented Dairy: Yogurt and kefir are fermented dairy products that can be beneficial for gut health, which is linked to overall heart health. Choose varieties with live active cultures.  7. Balance Your Diet: Make sure your diet is balanced with plenty of fruits, vegetables, whole grains, lean proteins, and healthy fats. Dairy should be just one component of a varied and nutritious diet.  Remember to consult with a healthcare provider or a dietitian to tailor these recommendations to your specific health needs and dietary preferences. |
| **GPT4o Answer 3** | Yes, you can have dairy on a heart-healthy diet, but it's important to choose the right types and consume them in moderation. Here are some guidelines:  1. Choose Low-Fat or Fat-Free Options: Opt for low-fat or fat-free milk, yogurt, and cheese instead of full-fat versions. This helps reduce your intake of saturated fat, which can contribute to heart disease.  2. Limit Portion Sizes: Even low-fat dairy products should be consumed in moderation. Pay attention to serving sizes to avoid excessive calorie intake.  3. Watch for Added Sugars: Some dairy products, like flavored yogurts, can be high in added sugars. Choose plain versions and add your own fruit for sweetness.  4. Include Dairy as Part of a Balanced Diet: Incorporate dairy into a diet rich in fruits, vegetables, whole grains, lean proteins, and healthy fats.  5. Consider Alternative Dairy Options: If you are lactose intolerant or prefer non-dairy options, look for fortified plant-based milk alternatives like almond, soy, or oat milk. Ensure they are fortified with calcium and vitamin D.  Examples of Heart-Healthy Dairy Choices  - Skim or 1% Milk: These have less saturated fat compared to whole milk.  - Greek Yogurt: Opt for non-fat or low-fat versions. It’s also higher in protein.  - Low-Fat Cottage Cheese: A good source of protein with less fat.  - Part-Skim Cheese: Look for versions like part-skim mozzarella or reduced-fat cheddar.  Benefits of Dairy in a Heart-Healthy Diet  - Calcium: Essential for maintaining strong bones and teeth.  - Vitamin D: Helps with calcium absorption and has other health benefits.  - Protein: Important for muscle repair and overall health.  Remember, individual dietary needs can vary, so it’s always a good idea to consult with a healthcare provider or a registered dietitian for personalized advice |
| **Llama 3 Answer 1** | Dairy can be a part of a heart-healthy diet, but it depends on the type and amount of dairy you consume.  The good news:  1. Calcium and potassium: Dairy products are rich in calcium, potassium, and other essential nutrients that can help lower blood pressure and support heart health.  2. Protein: Dairy is a good source of protein, which can help with weight management and satiety.  3. Fatty acid profile: Some dairy products, like cheese and whole milk, contain conjugated linoleic acid (CLA), a potentially beneficial fatty acid.  The not-so-good news:  1. Saturated fat: Whole dairy products, like cheese, whole milk, and cream, are high in saturated fat, which can increase cholesterol levels and heart disease risk when consumed excessively.  2. Cholesterol: Dairy products contain cholesterol, which can also contribute to high cholesterol levels and heart disease risk.  3. Added sugars: Some dairy products, like flavored yogurts and milk, can contain significant amounts of added sugars, which can increase heart disease risk.  Heart-healthy dairy choices:  1. Low-fat or fat-free dairy: Choose low-fat or fat-free milk, yogurt, and cheese to reduce saturated fat and cholesterol intake.  2. Cultured dairy: Fermented dairy products, like yogurt and kefir, contain probiotics, which may help support heart health.  3. Grass-fed or pasture-raised dairy: These dairy products may be higher in beneficial fatty acids, like omega-3s, and conjugated linoleic acid (CLA).  4. Limited portion sizes: Consume dairy products in moderation, as part of a balanced diet, to minimize excessive calorie, saturated fat, and cholesterol intake.  Tips for incorporating dairy into a heart-healthy diet:  1. Start with small amounts: If you're new to dairy or have concerns about saturated fat intake, start with small amounts (e.g., 1/2 cup of milk or 1 ounce of cheese per day).  2. Choose dairy as a substitute: Use dairy products as a substitute for less healthy options, like processed meats or refined carbohydrates.  3. Balance with other nutrient-dense foods: Make sure your diet is well-rounded and includes plenty of fruits, vegetables, whole grains, lean protein sources, and healthy fats.  In summary, dairy can be a part of a heart-healthy diet when chosen wisely and consumed in moderation. Opt for low-fat or fat-free, cultured, and grass-fed or pasture-raised dairy products, and be mindful of portion sizes to minimize excessive saturated fat and cholesterol intake. |
| **Llama 3 Answer 2** | Dairy can be a part of a heart-healthy diet, but it's essential to choose the right types and amounts. Here's what you need to know:  The good news:  1. Calcium and potassium: Dairy products are rich in calcium, potassium, and other minerals that help lower blood pressure and support heart health.  2. Protein: Dairy provides high-quality protein, which can help with weight management and overall health.  3. Some dairy products may help with cholesterol: Fermented dairy products like yogurt and cheese contain probiotics, which may help improve cholesterol levels and reduce blood pressure.  The not-so-good news:  1. Saturated fat: Whole milk, cheese, and butter are high in saturated fat, which can increase cholesterol levels and heart disease risk.  2. Added sugars: Flavored yogurts and dairy desserts can be high in added sugars, which are detrimental to heart health.  3. High-sodium content: Some dairy products, like processed cheese and butter, can be high in sodium, which can increase blood pressure.  Heart-healthy dairy choices:  1. Fat-free or low-fat dairy: Choose fat-free or low-fat milk, cheese, and yogurt to reduce saturated fat intake.  2. Unsweetened and unflavored dairy: Opt for unsweetened and unflavored yogurt and milk to avoid added sugars.  3. Fermented dairy: Enjoy fermented dairy products like yogurt, kefir, and cheese, which contain probiotics that support heart health.  4. Calcium-fortified plant-based dairy alternatives: If you're lactose intolerant or prefer plant-based options, choose calcium-fortified alternatives like soy milk, almond milk, or oat milk.  Portion control is key:  1. Limit daily dairy intake: Aim for 1-2 cups of dairy products per day, including milk, cheese, and yogurt.  2. Watch serving sizes: Be mindful of portion sizes, especially with high-calorie dairy products like cheese and butter.  In summary, dairy can be a part of a heart-healthy diet when you choose low-fat, unsweetened, and fermented options, and consume them in moderation. Always prioritize a balanced diet rich in fruits, vegetables, whole grains, lean protein, and healthy fats. |
| **Llama 3 Answer 3** | The age-old question about dairy and heart health:  The answer is a resounding "maybe." It depends on the type of dairy, the amount consumed, and individual factors such as lactose tolerance and overall diet quality.  Here's the scoop:  The good:  1. Calcium and protein: Dairy products are excellent sources of calcium, protein, and other essential nutrients, which are important for bone health and overall nutrition.  2. Potential heart benefits: Some studies suggest that moderate dairy consumption (1-2 servings per day) may be associated with a lower risk of cardiovascular disease, particularly when part of a balanced diet.  The not-so-good:  1. Saturated fat: Whole dairy products, like cheese and full-fat milk, contain saturated fat, which can increase cholesterol levels and heart disease risk when consumed excessively.  2. Cholesterol: Dairy products can be high in cholesterol, which may be a concern for individuals with high cholesterol levels or heart disease.  3. Added sugars: Flavored milk, yogurt, and cheese products can be high in added sugars, which are detrimental to heart health.  The heart-healthy dairy options:  1. Fat-free or low-fat dairy: Choose fat-free or low-fat milk, cheese, and yogurt to reduce saturated fat intake.  2. Cultured dairy: Fermented dairy products like yogurt and kefir contain probiotics, which may help lower blood pressure and improve cardiovascular health.  3. High-calcium, low-fat dairy: Select dairy products that are high in calcium and low in fat, such as part-skim mozzarella cheese or reduced-fat cottage cheese.  Tips for incorporating dairy into a heart-healthy diet:  1. Consume in moderation: Limit daily dairy intake to 1-2 servings (1 serving = 1 cup milk or yogurt, 1.5 ounces cheese).  2. Choose heart-healthy options: Opt for fat-free or low-fat dairy products, and select cultured or high-calcium, low-fat options.  3. Balance with other nutrients: Ensure your diet is well-rounded with plenty of fruits, vegetables, whole grains, lean protein, and healthy fats.  4. Consider individual needs: If you're lactose intolerant, have high cholesterol, or are at risk for heart disease, consult with a healthcare professional or registered dietitian for personalized guidance.  In summary, dairy can be part of a heart-healthy diet when consumed in moderation, chosen wisely, and balanced with other nutrient-dense foods. |
| **LLaMA3+RAG model Answer 1** | The American Heart Association (AHA) recommends choosing low-fat or fat-free dairy products to minimize your intake of saturated fats, cholesterol, and calories.  According to the AHA, adults should aim for two to three servings of fat-free or low-fat dairy products daily, while children should have two to two and a half servings a day. Teenagers and older adults should aim for three servings each day. A serving size is typically one cup of milk or yogurt, 1.5 ounces of cheese, or 2 ounces of processed cheese.  When selecting dairy products, opt for:  Fat-free milk, 1% low-fat milk, or fortified fat-free milk powder  Plain fat-free or low-fat yogurt, frozen fat-free or low-fat yogurt, or drinks made with fat-free or 1% fat milk and cocoa  Low-fat cheeses (dry-curd or low-fat, cottage cheese, low-fat natural cheeses, or processed cheeses made with fat-free or low-fat milk)  Fat-free or low-fat ice cream (no more than 3 grams of fat per 1/2 cup serving)  Avoid or limit:  Whole milk and full-fat dairy products (cheeses, yogurt, and ice cream)  Milk substitutes containing coconut oil, palm oil, or palm kernel oil, which are high in saturated fats  It's also important to note that plant-based milk alternatives can be a part of a heart-healthy diet, but choose products that are fortified with vitamins A and D, such as oat, soy, or almond milk.  Remember, a heart-healthy diet is about balance and variety. Dairy products can be a nutritious part of your diet when chosen wisely and consumed in moderation.  Sources:  American Heart Association. (2024). How Do I Follow a Healthy Diet? Retrieved from <https://www.heart.org/-/media/files/health-topics/answers-by-heart/how-do-i-follow-a-healthy-diet.pdf>  American Heart Association. (2024). Dairy Products, Milk, Yogurt, and Cheese. Retrieved from <https://www.heart.org/en/healthy-living/healthy-eating/eat-smart/nutrition-basics/dairy-products-milk-yogurt-and-cheese> |
| **LLaMA3+RAG model Answer 2** | The American Heart Association (AHA) recommends opting for low-fat or fat-free dairy products to reap the nutritional benefits while reducing the risk of heart disease.  According to the AHA, adults and children aged 2 and older should consume milk that is low in dairy fats, such as fortified fat-free milk, 1% and 2% low-fat milk. These options provide more nutrients and fewer calories compared to whole milk and 2% fat milk.  The AHA suggests the following dairy products as part of a heart-healthy diet:  Fat-free milk  1% low-fat milk  Fat-free or low-fat dry milk powder  Evaporated fat-free milk  Buttermilk made from fat-free or 1% fat milk  Plain fat-free or low-fat yogurt  Frozen fat-free or low-fat yogurt  Drinks made with fat-free or 1% fat milk and cocoa (or other low-fat drink powders)  Low-fat cheeses (dry-curd or low-fat, cottage cheese, low-fat natural cheeses or processed cheeses made with fat-free or low-fat milk)  It's also important to be mindful of the recommended daily servings of dairy products:  For adults: 2-3 servings of fat-free or low-fat dairy products daily  For children: 2-2.5 servings a day  For teenagers and older adults: 3 servings each day  On the other hand, it's recommended to avoid or limit the following dairy products:  Whole milk  Full-fat cheeses, yogurt, and ice cream  Milk substitutes that contain coconut oil, palm oil, or palm kernel oil, which are high in saturated fats  To make the transition to a heart-healthy dairy routine, you can start by gradually switching from whole-milk products to lower-fat options. For example, try 2% low-fat milk first, then move to 1% low-fat milk, and eventually to fat-free milk.  Remember, a heart-healthy diet is all about balance and variety. By making informed choices and incorporating low-fat or fat-free dairy products into your diet, you can enjoy the nutritional benefits of dairy while reducing your risk of heart disease.  Sources:  American Heart Association. (2024). How Do I Follow a Healthy Diet? Retrieved from <https://www.heart.org/-/media/files/health-topics/answers-by-heart/how-do-i-follow-a-healthy-diet.pdf>  American Heart Association. (2024). Dairy Products, Milk, Yogurt, and Cheese. Retrieved from <https://www.heart.org/en/healthy-living/healthy-eating/eat-smart/nutrition-basics/dairy-products-milk-yogurt-and-cheese> |
| **LLaMA3+RAG model Answer 3** | The American Heart Association (AHA) recommends consuming fat-free or low-fat dairy products as part of a healthy eating pattern.  According to the AHA, adults and children aged 2 and older should opt for milk that is low in dairy fats, such as fortified fat-free milk, 1% and 2% low-fat milk. These options provide more nutrients and are lower in fat, saturated fat, cholesterol, and calories compared to whole milk.  The AHA suggests consuming two to three servings of fat-free or low-fat dairy products daily for adults, and two to two and a half servings for children. For teenagers and older adults, three servings per day are recommended.  When choosing dairy products, opt for:  Fat-free milk  1% low-fat milk  Fat-free or low-fat dry milk powder  Evaporated fat-free milk  Buttermilk made from fat-free or 1% fat milk  Plain fat-free or low-fat yogurt  Frozen fat-free or low-fat yogurt  Drinks made with fat-free or 1% fat milk and cocoa (or other low-fat drink powders)  Low-fat cheeses (dry-curd or low-fat, cottage cheese, low-fat natural cheeses or processed cheeses made with fat-free or low-fat milk)  On the other hand, it's recommended to avoid or limit:  Whole milk  Full-fat cheeses, yogurt, and ice cream  Milk substitutes that contain coconut oil, palm oil, or palm kernel oil, which are high in saturated fats  Additionally, if you're transitioning from whole-milk products to fat-free or low-fat options, you can do so gradually to ease the change in taste and texture.  It's also important to note that plant-based milk alternatives can be a healthy option, but choose products that have been fortified with vitamins A and D, such as oat, soy, or almond milk.  In summary, dairy can be a part of a heart-healthy diet when chosen wisely and consumed in moderation.  Sources:  American Heart Association. (2024). How Do I Follow a Healthy Diet? Retrieved from <https://www.heart.org/-/media/files/health-topics/answers-by-heart/how-do-i-follow-a-healthy-diet.pdf>  American Heart Association. (2024). Dairy Products, Milk, Yogurt, and Cheese. Retrieved from <https://www.heart.org/en/healthy-living/healthy-eating/eat-smart/nutrition-basics/dairy-products-milk-yogurt-and-cheese> |
| **Question 19** | **What are some heart healthy breakfast ideas?** |
| **Perplexity Answer 1** | Here are some heart-healthy breakfast ideas:  Oatmeal  Oatmeal is a great breakfast choice, as it is high in fiber and associated with lowering cholesterol and aiding weight control[4]. For a heart-healthy oatmeal, use rolled oats (not instant), cook with water instead of milk or butter, and top with berries, ground flax seed, and a few walnuts[4]. You can also make overnight oats with chia seeds soaked in non-dairy milk, dried or frozen fruit, and nuts or seeds[4].  Smoothies and Shakes  Protein-based shakes made with pea protein, unsweetened soy milk, and fruit like berries or banana are a good option[4]. For a smoothie, blend whole vegetables and fruits to get the most fiber, such as tomatoes, celery, carrots, ginger, chia seeds, and apple[4].  Avocado Toast  Top a slice of 100% whole grain bread with a thin layer of avocado and vegetables like onions or tomatoes[4]. Avocados can help lower cholesterol, but consume in moderation due to the high calorie content[4].  Fruit, Whole Grain Crackers and Cheese  For a quick on-the-go breakfast, pair fruit, whole grain crackers, and a piece of cheese[1].  Homemade Muffins  Make a batch of healthy muffins like Vegan Sweet Potato Date Muffins and keep them in the freezer for an easy breakfast[5].  Whole Grain Pancakes  Whole Wheat Pancakes made with whole wheat flour and buttermilk are filling yet light[3]. Serve with hot chocolate for a delicious and nutritious breakfast.  Eggs  While the cholesterol in eggs is still debated, they can be part of a heart-healthy breakfast in moderation[4]. Try a Curry Scramble with sliced peppers[3] or Italian Cloud Eggs with whipped Italian-seasoned egg whites[3].  The key is to focus on fiber-rich whole grains, fruits, vegetables, lean proteins, and healthy fats like those found in avocados, nuts and seeds. Limit processed meats, sugary cereals, and high-sodium foods.  Citations:  [1] https://www.heartandstroke.ca/articles/20-fast-and-healthy-breakfasts  [2] https://www.signos.com/blog/heart-healthy-breakfast  [3] https://www.tasteofhome.com/collection/heart-healthy-breakfasts/  [4] https://www.today.com/health/diet-fitness/what-cardiologists-eat-for-breakfast-rcna89695  [5] https://www.epicurious.com/recipes-menus/heart-healthy-breakfast |
| **Perplexity Answer 2** | Here are some heart-healthy breakfast ideas:  Oatmeal  Oatmeal is a great choice for a heart-healthy breakfast. It is high in fiber, vitamins, and minerals, and studies show it can help lower cholesterol and aid in weight control[4]. For a nutritious oatmeal breakfast, use rolled oats (not instant) and top with berries, ground flax seed, and a few walnuts[4]. You can also make overnight oats with chia seeds soaked in non-dairy milk, dried or frozen fruit, and nuts or seeds[4].  Smoothies and Shakes  Smoothies and shakes can be a quick and easy way to get a heart-healthy breakfast. For a protein-based shake, blend pea protein, unsweetened soy milk, and berries or a banana[4]. For a smoothie packed with fiber, blend whole vegetables and fruits like tomatoes, celery, carrots, ginger, and an orange[4].  Avocado Toast  Top a slice of 100% whole grain bread with a thin layer of avocado and vegetables like onions or tomatoes for a heart-healthy avocado toast[4]. Avocados can help lower cholesterol, but be mindful of portion sizes as the calories can add up quickly[4].  Muffins and Baked Goods  For a heartier breakfast, try vegan sweet potato date muffins or a breakfast amaranth bowl with walnuts and honey[5]. These baked goods are designed to be heart-healthy with fiber-rich ingredients and limited saturated fat and sodium[5].  Other heart-healthy breakfast ideas include:  - Fruit, whole-grain crackers, and a piece of cheese[1]  - A homemade muffin from the freezer with a cheese string and clementine[1]  - Overnight oats with chia seeds, dried or frozen fruit, and nuts or seeds[2]  - Whole grain cereals and breads[2]  - Eggs (in moderation)[4]  The key is to focus on fiber-rich whole grains, fruits, vegetables, lean proteins, and healthy fats while limiting processed meats, sugary foods, and high-sodium items. Discuss any dietary changes with your healthcare provider to ensure they are right for you.  Citations:  [1] https://www.heartandstroke.ca/articles/20-fast-and-healthy-breakfasts  [2] https://www.signos.com/blog/heart-healthy-breakfast  [3] https://www.tasteofhome.com/collection/heart-healthy-breakfasts/  [4] https://www.today.com/health/diet-fitness/what-cardiologists-eat-for-breakfast-rcna89695  [5] https://www.epicurious.com/recipes-menus/heart-healthy-breakfast |
| **Perplexity Answer 3** | Here are some heart-healthy breakfast ideas:  Oatmeal  Oatmeal is a great breakfast choice, as it is high in fiber and can help lower cholesterol. Top it with berries, nuts, and seeds for added nutrients[1][2][4]. For convenience, make overnight oats with chia seeds, non-dairy milk, and fruit[4].  Avocado Toast  Toast a slice of 100% whole grain bread and top it with a thin layer of avocado and vegetables like onions or tomatoes. Avocados are high in heart-healthy monounsaturated fats[4].  Smoothies and Shakes  Blend together a protein-based shake with pea protein, unsweetened soy milk, and fruit[4]. For a nutrient-dense smoothie, blend whole vegetables and fruits like tomatoes, celery, carrots, ginger, and citrus fruits[4].  Fruit and Nuts  For a quick breakfast on-the-go, pair fruit like berries or citrus with whole grain crackers and a piece of cheese, or have a homemade muffin with cheese and clementines[1]. Nuts like walnuts provide heart-healthy fats and fiber[2].  Eggs  While eggs are a source of cholesterol, research shows they may not significantly raise heart disease risk for most people. Eggs can be part of a heart-healthy breakfast in moderation[4].  Whole Grain Pancakes and French Toast  Make pancakes and French toast with whole wheat flour for added fiber. Top with fruit like berries or bananas for extra nutrients[3].  The key is to focus on fruits, vegetables, whole grains, lean proteins, and healthy fats. Limit processed meats like bacon and sugary foods. With a little preparation, it's easy to enjoy a delicious and heart-healthy breakfast.  Citations:  [1] https://www.heartandstroke.ca/articles/20-fast-and-healthy-breakfasts  [2] https://www.signos.com/blog/heart-healthy-breakfast  [3] https://www.tasteofhome.com/collection/heart-healthy-breakfasts/  [4] https://www.today.com/health/diet-fitness/what-cardiologists-eat-for-breakfast-rcna89695  [5] https://www.epicurious.com/recipes-menus/heart-healthy-breakfast |
| **GPT4o Answer 1** | Eating a heart-healthy breakfast can help you start your day with the right nutrients to support cardiovascular health. Here are some nutritious and delicious options:  Oatmeal  - Ingredients: Whole oats, water or milk (low-fat or plant-based), fresh fruit, nuts, and seeds.  - Benefits: Oats are high in soluble fiber, which can help lower cholesterol levels.  Greek Yogurt with Berries  - Ingredients: Non-fat or low-fat Greek yogurt, mixed berries (blueberries, strawberries, raspberries), and a drizzle of honey.  - Benefits: Greek yogurt provides protein and probiotics, while berries are rich in antioxidants and fiber.  Avocado Toast  - Ingredients: Whole-grain bread, ripe avocado, a squeeze of lemon juice, and a sprinkle of chia or flax seeds.  - Benefits: Avocados are rich in healthy fats and fiber, and whole-grain bread adds complex carbohydrates.  Smoothie Bowl  - Ingredients: Spinach or kale, frozen berries, a banana, unsweetened almond milk, and a scoop of flaxseed or chia seeds.  - Benefits: Green leafy vegetables and berries provide essential vitamins and antioxidants, and seeds add omega-3 fatty acids.  Egg White Omelet  - Ingredients: Egg whites, spinach, tomatoes, bell peppers, onions, and a sprinkle of low-fat cheese.  - Benefits: Egg whites are a good source of protein without the cholesterol found in yolks, and vegetables add fiber and nutrients.  Chia Pudding  - Ingredients: Chia seeds, almond milk, a touch of vanilla extract, and fresh fruit toppings.  - Benefits: Chia seeds are rich in omega-3 fatty acids, fiber, and protein.  Whole-Grain Cereal  - Ingredients: Whole-grain cereal (like bran flakes or steel-cut oats), skim milk or a plant-based alternative, and fresh fruit.  - Benefits: Whole grains provide fiber and essential nutrients, and pairing them with fruit adds vitamins and antioxidants.  Nut Butter and Banana on Whole-Grain Bread  - Ingredients: Whole-grain bread, natural almond or peanut butter, and banana slices.  - Benefits: Nut butters provide healthy fats and protein, and bananas offer potassium and fiber.  Quinoa Breakfast Bowl  - Ingredients: Cooked quinoa, fresh berries or chopped apple, a handful of nuts or seeds, and a drizzle of honey or maple syrup.  - Benefits: Quinoa is a complete protein and high in fiber, making it a great base for a heart-healthy breakfast.  Cottage Cheese with Fruit  - Ingredients: Low-fat cottage cheese, pineapple chunks or other fresh fruit, and a sprinkle of cinnamon.  - Benefits: Cottage cheese is high in protein and low in fat, and fruit adds vitamins and fiber.  Additional Tips:  - Limit added sugars and saturated fats. Opt for natural sweeteners like fruit and use healthy fats from sources like nuts, seeds, and avocados.  - Incorporate a variety of colorful fruits and vegetables. These provide essential vitamins, minerals, and antioxidants that support heart health.  - Stay hydrated. Drink water or herbal teas to keep your body hydrated throughout the day.  Incorporating these heart-healthy breakfast ideas can help you maintain good cardiovascular health and provide sustained energy throughout the morning. |
| **GPT4o Answer 2** | Here are some heart-healthy breakfast ideas that are both nutritious and delicious:  1. Oatmeal: Top with fresh berries, a handful of nuts, and a drizzle of honey. Oatmeal is rich in soluble fiber, which can help lower cholesterol levels.  2. Greek Yogurt with Fruit and Nuts: Greek yogurt is high in protein, and adding fresh fruits like blueberries or strawberries along with nuts like almonds or walnuts can provide healthy fats and antioxidants.  3. Whole Grain Toast with Avocado: Mash an avocado and spread it on whole grain toast. Sprinkle with a pinch of salt, pepper, and a squeeze of lemon juice. Avocados are high in monounsaturated fats, which are good for heart health.  4. Smoothie Bowl: Blend spinach, banana, berries, and a splash of almond milk or yogurt. Top with chia seeds, flaxseeds, and a few slices of banana or berries. Smoothies are a great way to pack in a lot of nutrients.  5. Chia Pudding: Mix chia seeds with almond milk and let it sit overnight. In the morning, top with fresh fruit and a sprinkle of nuts or seeds. Chia seeds are rich in omega-3 fatty acids, which are good for heart health.  6. Egg White Omelet: Make an omelet with egg whites and fill it with vegetables like spinach, tomatoes, and bell peppers. Add a slice of whole grain toast on the side. Egg whites are a low-fat source of protein.  7. Quinoa Breakfast Bowl: Cook quinoa and mix it with almond milk, a bit of cinnamon, and a handful of berries or sliced apple. Quinoa is a complete protein and a good source of fiber.  8. Cottage Cheese with Fruit: Cottage cheese is high in protein and pairs well with fruits like peaches, pineapples, or berries. Add a sprinkle of flaxseeds for extra fiber and omega-3s.  9. Banana Pancakes: Make pancakes using mashed bananas and oats. Serve with a dollop of Greek yogurt and fresh berries. These pancakes are high in fiber and potassium.  10. Whole Grain Muffins: Bake muffins using whole grain flour and add fruits like blueberries or raspberries. These can be made ahead of time and are a convenient option for a busy morning.  These breakfast ideas focus on whole foods, rich in fiber, healthy fats, and lean proteins, all of which are beneficial for maintaining heart health. |
| **GPT4o Answer 3** | Here are some heart-healthy breakfast ideas that are nutritious and delicious:  1. Oatmeal:  - Toppings: Fresh fruits (berries, bananas, apples), nuts (almonds, walnuts), seeds (chia, flax), and a drizzle of honey or maple syrup.  - Benefits: Oats are high in fiber, which can help lower cholesterol levels and keep your heart healthy.  2. Greek Yogurt with Fresh Fruit:  - Toppings: Berries, a sprinkle of granola, and a drizzle of honey.  - Benefits: Greek yogurt is high in protein and probiotics, which are good for gut health and overall well-being.  3. Smoothie Bowl:  - Ingredients: Blend spinach, kale, or other leafy greens with a banana, berries, and a splash of almond milk. Top with nuts, seeds, and a few pieces of fresh fruit.  - Benefits: Packed with vitamins, minerals, and antioxidants, which are all great for heart health.  4. Whole Grain Toast with Avocado and Egg:  - Toppings: Mashed avocado, a poached or boiled egg, a sprinkle of chili flakes, and a squeeze of lemon juice.  - Benefits: Avocado is rich in healthy fats and eggs provide protein and essential nutrients.  5. Chia Pudding:  - Ingredients: Mix chia seeds with almond milk and let it sit overnight. In the morning, top with fresh fruit and nuts.  - Benefits: Chia seeds are high in omega-3 fatty acids, fiber, and antioxidants.  6. Smoothies:  - Ingredients: Blend a combination of fruits (like berries, bananas, apples), vegetables (like spinach, kale), and a protein source (like Greek yogurt or protein powder).  - Benefits: Easy to digest and packed with nutrients that support heart health.  7. Quinoa Breakfast Bowl:  - Ingredients: Cook quinoa and top with mixed berries, a handful of nuts, and a drizzle of almond or peanut butter.  - Benefits: Quinoa is a complete protein and high in fiber, which helps in maintaining healthy cholesterol levels.  8. Nut Butter on Whole Grain Bread:  - Toppings: Add a layer of natural almond or peanut butter and top with sliced bananas or strawberries.  - Benefits: Nut butters are rich in healthy fats and protein, while whole grain bread provides fiber.  9. Vegetable Omelette:  - Ingredients: Eggs or egg whites mixed with a variety of vegetables like spinach, tomatoes, bell peppers, and onions.  - Benefits: High in protein and vitamins, and using vegetables adds fiber and antioxidants.  10. Cottage Cheese with Fresh Fruit:  - Toppings: Add peaches, pineapple, or berries to cottage cheese.  - Benefits: Cottage cheese is high in protein and calcium, and adding fruit increases fiber and vitamin intake.  These breakfast options are designed to be low in unhealthy fats and sugars while being rich in nutrients that support heart health. |
| **Llama 3 Answer 1** | Starting your day with a heart-healthy breakfast can help boost your overall cardiovascular well-being. Here are some delicious and nutritious breakfast ideas to get you going:  1. Oatmeal with Fresh Fruits and Nuts: Steel-cut oats or rolled oats cooked with low-fat milk or water, topped with fresh fruits like berries, bananas, or apples, and chopped nuts like almonds or walnuts.  2. Avocado Toast: Whole-grain toast topped with mashed avocado, a fried egg, and a sprinkle of salt and pepper. The healthy fats in avocado support heart health.  3. Greek Yogurt Parfait: Greek yogurt layered with fresh berries, granola, and a sprinkle of chia seeds. The protein and calcium in yogurt help lower blood pressure.  4. Smoothie Bowl: A blend of frozen berries, banana, spinach, and almond milk topped with sliced almonds, chia seeds, and shredded coconut. The antioxidants and fiber in berries support heart health.  5. Whole-Grain Waffles with Fresh Fruits and Yogurt: Whole-grain waffles topped with fresh fruits like strawberries or blueberries, and a dollop of Greek yogurt.  6. Egg White Omelette with Spinach and Mushrooms: A protein-rich omelette made with egg whites, spinach, and mushrooms, cooked in a heart-healthy oil like olive or avocado oil.  7. Chia Seed Pudding with Banana and Honey: A bowl of chia seeds soaked in almond milk, topped with sliced banana and a drizzle of honey. Chia seeds are rich in omega-3 fatty acids and fiber.  8. Green Smoothie: A blend of spinach, avocado, banana, and almond milk, packed with antioxidants and healthy fats.  9. Cottage Cheese with Fresh Fruits and Cinnamon: Cottage cheese mixed with fresh fruits like berries or sliced peaches, and a sprinkle of cinnamon. The protein and potassium in cottage cheese support heart health.  10. Whole-Grain Cereal with Almond Milk and Sliced Banana: A bowl of whole-grain cereal like oat bran or bran flakes, served with almond milk and sliced banana.  Remember to choose whole grains, fruits, and lean protein sources, and limit your intake of added sugars, saturated fats, and sodium. |
| **Llama 3 Answer 2** | Starting your day with a heart-healthy breakfast can help set you up for a nutritious and energized day. Here are some delicious and healthy breakfast ideas that can support heart health:  1. Oatmeal with Fresh Fruits and Nuts: Steel-cut oats or rolled oats cooked with low-fat milk or water, topped with fresh fruits and chopped nuts (e.g., walnuts, almonds) provide a boost of fiber, antioxidants, and healthy fats.  2. Avocado Toast: Whole-grain toast topped with mashed avocado, salt, and pepper, and a sliced egg or a sprinkle of red pepper flakes for added protein.  3. Greek Yogurt Parfait: Greek yogurt layered with fresh berries, granola, and a sprinkle of chia seeds provides protein, calcium, and fiber.  4. Smoothie Bowl: A blend of frozen berries, banana, spinach, and almond milk topped with sliced almonds, chia seeds, and shredded coconut.  5. Whole-Grain Waffles with Fresh Fruits and Yogurt: Whole-grain waffles made with heart-healthy oils (e.g., canola, olive) and topped with fresh fruits and a dollop of Greek yogurt.  6. Egg White Omelette with Spinach and Mushrooms: A protein-rich omelette made with egg whites, spinach, and mushrooms, cooked with heart-healthy oils.  7. Chia Seed Pudding: Mix chia seeds with almond milk and let it sit overnight. Top with fresh fruits and a drizzle of honey for a nutritious and filling breakfast.  8. Cottage Cheese with Fresh Fruits and Cinnamon: Cottage cheese is a great source of protein and calcium. Mix it with fresh fruits and a sprinkle of cinnamon for a delicious and healthy breakfast.  9. Green Smoothie: Blend spinach, avocado, banana, and almond milk for a nutrient-packed smoothie that's rich in antioxidants and healthy fats.  10. Whole-Grain Cereal with Almond Milk and Sliced Banana: A bowl of whole-grain cereal with almond milk and sliced banana provides a good source of fiber, vitamins, and minerals.  Remember to choose whole, unprocessed foods whenever possible, and limit your intake of added sugars, saturated fats, and sodium. Also, be mindful of your portion sizes and calorie needs to maintain a healthy weight and support overall heart health. |
| **Llama 3 Answer 3** | Starting your day with a heart-healthy breakfast can help boost your energy, support your overall well-being, and even reduce your risk of heart disease. Here are some delicious and nutritious heart-healthy breakfast ideas:  1. Oatmeal with Fresh Fruits and Nuts: Steel-cut oats or rolled oats cooked with low-fat milk or water, topped with fresh fruits like berries, banana, or sliced apples, and a sprinkle of chopped nuts like almonds or walnuts.  2. Avocado Toast: Whole-grain toast topped with mashed avocado, a sliced egg, and a sprinkle of salt and pepper. Add some cherry tomatoes or spinach for extra nutrition.  3. Greek Yogurt Parfait: Greek yogurt layered with fresh berries, granola, and a sprinkle of chia seeds. You can also add some sliced banana or honey for extra flavor.  4. Smoothie Bowl: A bowl made with a blend of frozen berries, banana, spinach, and almond milk, topped with sliced fruits, nuts, and seeds like chia, flax, or hemp.  5. Whole-Grain Waffles with Fresh Fruits and Yogurt: Make whole-grain waffles and top them with fresh fruits like strawberries, blueberries, or bananas, and a dollop of low-fat yogurt.  6. Veggie Scramble: Scrambled eggs with sautéed spinach, bell peppers, and onions, served with whole-grain toast or a whole-grain English muffin.  7. Chia Seed Pudding: Mix chia seeds with almond milk and let it sit overnight. Top with fresh fruits and a drizzle of honey for a nutritious and filling breakfast.  8. Heart-Healthy Pancakes: Make pancakes with whole-grain flour, eggs, and low-fat milk. Top with fresh fruits, a drizzle of honey, and a sprinkle of chopped nuts.  9. Breakfast Burrito: Scrambled eggs, black beans, and avocado wrapped in a whole-grain tortilla.  10. Cottage Cheese and Fresh Fruits: Mix cottage cheese with fruit like berries or sliced peaches, and a sprinkle of cinnamon for a high-protein breakfast.  Remember to choose whole grains, lean proteins, and a variety of fruits and vegetables to keep your breakfast heart-healthy. Limit your intake of added sugars, saturated fats, and sodium. |
| **LLaMA3+RAG model Answer 1** | Heart-healthy breakfast ideas are essential for a nutritious start to the day. According to the American Heart Association, incorporating breakfast into your daily routine can help lower the risk of heart disease and stroke. Here are some delicious and healthy breakfast ideas to get you started:  1. Homemade Muffins: Make your own muffins using whole wheat flour, fruits, nuts, and vegetables. Freeze them and grab one on-the-go. Try the Cranberry Muffin recipe or experiment with different add-ins like shredded carrots, dried cranberries, and chopped nuts.  2. Instant Oatmeal: Prepare instant oatmeal with hot water from a coffee maker and add your choice of nuts, fruits, and spices. Look for unsweetened packets and flavor with cinnamon, applesauce, or frozen fruit.  3. DIY Smoothies: Blend together your favorite frozen fruits, low-fat milk or yogurt, and 100% fruit juice. Add some ice cubes for a refreshing crunch. Try the Basic Berry Smoothie recipe or experiment with different fruits and vegetables.  4. Whole-Grain Toast with Nut Butter and Fruit: Spread whole-grain toast with nut butter or low-fat cottage cheese and top with sliced fruit. Cut into halves for a mini-sandwich.  5. Yogurt Parfaits: Fill small mason jars with plain low-fat yogurt, frozen fruit, nuts, rolled oats, and a sprinkle of cinnamon. Prepare over the weekend for a few days' worth of healthy breakfasts.  6. Overnight Sorgum: Try this gluten-free alternative to overnight oats. Mix together sorgum, low-fat milk, and your choice of nuts, seeds, and fruits. Refrigerate overnight and enjoy in the morning.  7. Hard-Boiled Eggs: Boil eggs on the weekend and keep them in the fridge for a quick protein-packed breakfast on-the-go. Add some whole grain toast, avocado, and tomato for extra nutrition.  Remember to always check the nutrition facts label when choosing prepackaged breakfast foods to avoid sneaky salt and added sugars.  Sources:  American Heart Association. (2024). Breakfast Eating Healthy on the Run. Retrieved from <https://www.heart.org/en/healthy-living/healthy-eating/cooking-skills/meal-planning/breakfast-eating-healthy-on-the-run>  American Heart Association. (2024). Heart-Check Certified Recipes. Retrieved from <https://www.heart.org/en/healthy-living/company-collaboration/heart-check-certification/heart-check-certified-recipes> |
| **LLaMA3+RAG model Answer 2** | Here are some heart-healthy breakfast ideas According to the American Heart Association:  1. Homemade Muffins: Made with healthy ingredients like fruit, nuts, and whole wheat flour, these muffins can be frozen and grabbed on-the-go. Try the Make Your Own Whole-Wheat Muffins recipe provided, which uses unsweetened applesauce, nonfat milk, and canola oil.  2. Instant Oatmeal: A quick and easy whole grain option that can be made with hot water from a coffee maker. Look for flavors low in added sugars and consider adding nuts, fruit, or spices for extra flavor and nutrition.  3. DIY Smoothies: Blend together frozen fruit, low-fat milk or yogurt, and 100% fruit juice for a quick and nutritious breakfast. Try the Basic Berry Smoothie Recipe provided, which uses frozen berries, banana, and nonfat vanilla yogurt.  4. Overnight Sorgum: A gluten-free alternative to overnight oats, made with sorgum, milk, and flavorings of your choice.  5. Breakfast Hacks: Make ahead and freeze breakfast items like muffins, smoothies, and yogurt parfaits to grab-and-go in the morning.  6. Hard-Boiled Eggs: Boil eggs on the weekend and keep them in the fridge for a quick protein-packed breakfast on-the-go. Add some whole grain toast, avocado, and tomato for extra nutrition.  7. Whole Grain Toast with Nut Butter and Fruit: Spread whole grain toast with nut butter or low-fat cottage cheese and top with sliced fruit for a satisfying breakfast.  8. Yogurt Parfaits: Layer plain low-fat or fat-free yogurt, frozen fruit, nuts, and rolled oats in small mason jars for a healthy and convenient breakfast.  Remember to always choose whole grain products, limit added sugars and salt, and opt for unsweetened and low-fat ingredients whenever possible.  Sources:  American Heart Association. (2024). Breakfast Eating Healthy on the Run. Retrieved from <https://www.heart.org/en/healthy-living/healthy-eating/cooking-skills/meal-planning/breakfast-eating-healthy-on-the-run>  American Heart Association. (2024). Heart-Check Certified Recipes. Retrieved from <https://www.heart.org/en/healthy-living/company-collaboration/heart-check-certification/heart-check-certified-recipes> |
| **LLaMA3+RAG model Answer 3** | According to the American Heart Association, a heart-healthy breakfast is essential to kick-start your day and even lower your risk of heart disease and stroke. Here are some delicious and easy-to-make breakfast ideas that are perfect for a busy morning:  1. Homemade Muffins: Make your own muffins using healthy ingredients like fruits, nuts, and whole wheat flour. Freeze them and grab one on-the-go. Try the AHA's recipe for whole-wheat muffins with add-ins like shredded carrot, sunflower seeds, dried cranberries, or fresh blueberries.  2. Instant Oatmeal: Prepare instant oatmeal with hot water from your coffee maker. Choose flavors low in added sugars and add your own toppings like walnuts, applesauce, cinnamon, and frozen fruit.  3. DIY Smoothie: Blend together your favorite frozen fruits, low-fat milk or yogurt, and 100% fruit juice. Add some fresh veggies like spinach or kale for an extra nutritional boost. Try the AHA's Basic Berry Smoothie recipe or experiment with different fruits and ingredients.  4. Breakfast Hacks: Make ahead of time and grab-and-go with these ideas:  Pack a homemade smoothie in a thermos.  Keep a stash of hard-boiled eggs in the fridge and serve on whole grain toast with avocado and tomato.  Spread whole-grain waffles or toast with nut butter or low-fat cottage cheese and sliced fruit.  Prepare yogurt parfaits in small mason jars with plain low-fat yogurt, frozen fruit, nuts, and rolled oats.  5. Watch Out for Sneaky Ingredients: When choosing prepackaged breakfast foods, be mindful of added sugars and salt. Check the nutrition facts label and opt for whole-grain products with dietary fiber and other beneficial nutrients.  Remember, a healthy breakfast can help you feel full and energized until lunchtime, and it's an essential habit to develop for a heart-healthy lifestyle.  Sources:  American Heart Association. (2024). Breakfast Eating Healthy on the Run. Retrieved from <https://www.heart.org/en/healthy-living/healthy-eating/cooking-skills/meal-planning/breakfast-eating-healthy-on-the-run>  American Heart Association. (2024). Heart-Check Certified Recipes. Retrieved from <https://www.heart.org/en/healthy-living/company-collaboration/heart-check-certification/heart-check-certified-recipes> |
| **Question 20** | **How much added sugar is okay to consume in a heart healthy diet?** |
| **Perplexity Answer 1** | The American Heart Association (AHA) recommends limiting added sugar intake to no more than:  - 6 teaspoons (25 grams or 100 calories) per day for most adult women[4][5]  - 9 teaspoons (36 grams or 150 calories) per day for most adult men[4][5]  The AHA also suggests a lower daily limit of added sugars for children ages 2-18 to less than 6 teaspoons or 24 grams per day[5].  Consuming too much added sugar is linked to an increased risk of heart disease and cardiovascular mortality[1][2][3]. High sugar diets have been associated with higher blood pressure, insulin resistance, weight gain, and inflammation - all of which are risk factors for heart disease[1][2].  The average American consumes about 17 teaspoons of added sugar per day, which is more than 2-3 times the recommended amount[4]. The top sources of added sugar in the U.S. diet are sugar-sweetened beverages, desserts, sweet snacks, and processed foods[4][5].  To reduce added sugar intake, read nutrition labels, choose whole foods over processed foods, limit sugary drinks, and satisfy sweet cravings with fresh fruits[1][4]. Paying attention to added sugar consumption can help minimize excessive intake and lower the risk of heart disease in the long run[1][2][4].  Citations:  [1] https://www.scripps.org/news_items/7679-how-does-sugar-affect-your-heart-health  [2] https://www.health.harvard.edu/heart-health/the-sweet-danger-of-sugar  [3] https://pubmed.ncbi.nlm.nih.gov/24493081/  [4] https://www.heart.org/en/healthy-living/healthy-eating/eat-smart/sugar/how-much-sugar-is-too-much  [5] https://www.hsph.harvard.edu/nutritionsource/carbohydrates/added-sugar-in-the-diet/ |
| **Perplexity Answer 2** | The American Heart Association (AHA) recommends limiting added sugar intake to no more than 6 teaspoons (25 grams or 100 calories) per day for most adult women and no more than 9 teaspoons (36 grams or 150 calories) per day for most adult men[4][5]. Children ages 2-18 should limit added sugars to less than 6 teaspoons (24 grams) per day[5].  Regularly consuming high amounts of added sugar can lead to insulin resistance, which is associated with an increased risk of heart disease[1]. High sugar diets have been linked to higher blood pressure, a significant risk factor for heart disease and stroke[1]. Excess added sugar consumption can also contribute to weight gain, obesity, type 2 diabetes, and fatty liver disease, all of which increase cardiovascular disease risk[1][2].  A study published in JAMA Internal Medicine found that people who got 17% to 21% of their calories from added sugar had a 38% higher risk of dying from cardiovascular disease compared to those who consumed 8% of their calories as added sugar[2]. Most U.S. adults consume more added sugar than is recommended for a healthy diet[3].  To reduce added sugar intake, limit sugary beverages, desserts, and sweet snacks[4][5]. Read food labels and look for products with little or no added sugar[1][4]. Satisfy sweet cravings with whole fruits instead of candy or other sweets[1]. Making an effort to consume whole, unprocessed foods can significantly benefit heart health in the long run[1].  Citations:  [1] https://www.scripps.org/news_items/7679-how-does-sugar-affect-your-heart-health  [2] https://www.health.harvard.edu/heart-health/the-sweet-danger-of-sugar  [3] https://pubmed.ncbi.nlm.nih.gov/24493081/  [4] https://www.heart.org/en/healthy-living/healthy-eating/eat-smart/sugar/how-much-sugar-is-too-much  [5] https://www.hsph.harvard.edu/nutritionsource/carbohydrates/added-sugar-in-the-diet/ |
| **Perplexity Answer 3** | The American Heart Association (AHA) recommends limiting added sugar intake to no more than 6 teaspoons (25 grams or 100 calories) per day for most adult women and no more than 9 teaspoons (36 grams or 150 calories) per day for most adult men[4][5]. For children ages 2-18, the AHA recommends limiting added sugars to less than 6 teaspoons (24 grams) per day[5].  Consuming too much added sugar is linked to an increased risk of heart disease[1][2][3]. High sugar diets have been associated with higher blood pressure, insulin resistance, chronic inflammation, weight gain, fatty liver disease, and type 2 diabetes - all of which are risk factors for heart disease[1][2].  The average American consumes about 17 teaspoons (270 calories) of added sugar per day, which is over 2-3 times the recommended limit[4][5]. The top sources of added sugar in the U.S. diet are sugar-sweetened beverages, desserts, sweet snacks, and processed foods[4][5].  To reduce added sugar intake, read nutrition labels, choose whole foods over processed foods, limit sugary drinks, and satisfy sweet cravings with fresh fruits[1][4]. Consuming natural sugars found in whole fruits and vegetables is okay, as they contain beneficial nutrients and fiber[2][5].  Citations:  [1] https://www.scripps.org/news_items/7679-how-does-sugar-affect-your-heart-health  [2] https://www.health.harvard.edu/heart-health/the-sweet-danger-of-sugar  [3] https://pubmed.ncbi.nlm.nih.gov/24493081/  [4] https://www.heart.org/en/healthy-living/healthy-eating/eat-smart/sugar/how-much-sugar-is-too-much  [5] https://www.hsph.harvard.edu/nutritionsource/carbohydrates/added-sugar-in-the-diet/ |
| **GPT4o Answer 1** | In a heart-healthy diet, it's important to limit added sugar intake to reduce the risk of cardiovascular diseases. The American Heart Association (AHA) provides the following guidelines for added sugar consumption:  1. For Men: No more than 9 teaspoons (36 grams or 150 calories) of added sugar per day.  2. For Women: No more than 6 teaspoons (25 grams or 100 calories) of added sugar per day.  These guidelines are designed to help maintain heart health by preventing weight gain, lowering blood pressure, and reducing the risk of chronic conditions such as heart disease and type 2 diabetes. Reducing added sugar intake can also help improve overall diet quality, as it encourages the consumption of more nutrient-dense foods. |
| **GPT4o Answer 2** | In a heart-healthy diet, it's important to limit the intake of added sugars to reduce the risk of cardiovascular disease. The American Heart Association (AHA) provides specific guidelines for added sugar consumption:  - For women: No more than 6 teaspoons (25 grams or about 100 calories) of added sugar per day.  - For men: No more than 9 teaspoons (36 grams or about 150 calories) of added sugar per day.  These limits are recommended to help maintain a healthy weight, reduce the risk of heart disease, and promote overall health. It's important to note that these recommendations pertain specifically to added sugars, which are sugars added to foods during processing or preparation, and not naturally occurring sugars found in fruits and dairy products.  To adhere to these guidelines, it's beneficial to:  - Read food labels to check for added sugars in ingredients.  - Reduce consumption of sugary beverages, sweets, and processed foods.  - Opt for natural sweeteners like fruits or small amounts of honey when needed.  - Focus on a balanced diet rich in whole grains, fruits, vegetables, lean proteins, and healthy fats.  By keeping added sugar intake within these limits, you can support a heart-healthy diet and reduce the risk of developing cardiovascular diseases. |
| **GPT4o Answer 3** | The American Heart Association (AHA) provides guidelines on the amount of added sugar that can be consumed in a heart-healthy diet. These guidelines are:  - For women: No more than 6 teaspoons (25 grams or 100 calories) of added sugar per day.  - For men: No more than 9 teaspoons (36 grams or 150 calories) of added sugar per day.  These limits are based on reducing the risk of cardiovascular diseases and maintaining overall health. It's important to note that added sugars are those that are added to foods during processing or preparation, as well as those added at the table, and do not include naturally occurring sugars found in fruits, vegetables, and dairy products.  Maintaining these limits helps to control calorie intake and can reduce the risk of obesity, type 2 diabetes, and heart disease. It's advisable to check food labels and be mindful of the sugar content in foods and beverages to stay within these recommendations. |
| **Llama 3 Answer 1** | The American Heart Association (AHA) recommends that women consume no more than 25 grams (6 teaspoons) of added sugars per day, and men consume no more than 36 grams (9 teaspoons) per day. This is based on a 2,000 calorie diet, and the daily limit may be lower or higher depending on individual calorie needs.  Added sugars are sugars that are added to foods and beverages during processing or preparation, as opposed to naturally occurring sugars found in whole foods like fruits, vegetables, and dairy products. Consuming high amounts of added sugars has been linked to an increased risk of heart disease, type 2 diabetes, and certain cancers.  Here are some examples of the amount of added sugar in common foods and beverages:  * 12 oz can of soda: 39 grams (9.8 teaspoons)  * 1 cup of sweetened yogurt: 30-40 grams (7.5-10 teaspoons)  * 1 tablespoon of ketchup: 4 grams (! teaspoon)  * 1 cup of granola: 10-15 grams (2.5-3.75 teaspoons)  * 1 cup of flavored coffee creamer: 5-10 grams (1.25-2.5 teaspoons)  To reduce added sugar intake, the AHA recommends:  1. Reading food labels: Check the ingredient list for added sugars, which can be hidden under many names, such as high fructose corn syrup, honey, maple syrup, and agave nectar.  2. Choosing whole foods: Focus on whole, unprocessed foods like fruits, vegetables, whole grains, lean proteins, and healthy fats.  3. Limiting sugary drinks: Avoid sugary drinks like soda, sports drinks, and sweet tea, and opt for water, unsweetened tea, and black coffee instead.  4. Cooking at home: Prepare meals at home using fresh ingredients to control the amount of added sugar in your food.  5. Reducing portion sizes: Even healthy foods can become unhealthy if consumed in excess, so be mindful of portion sizes to reduce overall sugar intake.  Remember, it's not just about cutting out added sugars, but also about focusing on a balanced diet that includes a variety of whole, nutrient-dense foods. |
| **Llama 3 Answer 2** | The American Heart Association (AHA) recommends that women consume no more than 25 grams (6 teaspoons) of added sugars per day, and men consume no more than 36 grams (9 teaspoons) per day, as part of a heart-healthy diet.  Added sugars are sugars that are added to foods and beverages during processing or preparation, as opposed to naturally occurring sugars found in whole foods like fruits, vegetables, and dairy products. Consuming high amounts of added sugars has been linked to an increased risk of heart disease, type 2 diabetes, and certain cancers.  Here are some examples of the amount of added sugar in common foods and beverages:  * 1 can of soda: 39 grams (10 teaspoons)  * 1 cup of sweetened yogurt: 30-40 grams (7-10 teaspoons)  * 1 tablespoon of ketchup: 4 grams (1 teaspoon)  * 1 cup of granola: 10-15 grams (2-3 teaspoons)  * 1 energy drink: 20-30 grams (4-6 teaspoons)  * 1 cup of fruit juice: 20-30 grams (4-6 teaspoons)  To follow the AHA's recommendation, focus on whole, unprocessed foods like fruits, vegetables, whole grains, lean proteins, and healthy fats. Limit your intake of sugary drinks, baked goods, candy, and other sweets. Also, be mindful of hidden sources of added sugar in foods like sauces, condiments, and processed snacks.  Here are some tips to help you reduce your added sugar intake:  1. Read food labels: Check the ingredient list for added sugars, which can be listed under many names, including sugar, high-fructose corn syrup, honey, maple syrup, and agave nectar.  2. Choose whole foods: Focus on whole, unprocessed foods like fruits, vegetables, whole grains, lean proteins, and healthy fats.  3. Limit sugary drinks: Avoid sugary drinks like soda, sports drinks, and sweet tea, and opt for water, unsweetened tea, or seltzer water instead.  4. Cook at home: Preparing meals at home allows you to control the amount of added sugar that goes into your food.  5. Be mindful of portion sizes: Even healthy foods can become unhealthy if consumed in excess. Be mindful of your portion sizes and control the amount of added sugar you consume.  Remember, it's all about balance and moderation. If you're trying to reduce your added sugar intake, start by making small changes and gradually work your way towards the AHA's recommended daily limits. |
| **Llama 3 Answer 3** | Consuming too much added sugar has been linked to an increased risk of heart disease, so it's essential to limit your intake in a heart-healthy diet. Here are the guidelines:  American Heart Association (AHA) Recommendations:  * Women: No more than 25 grams (6 teaspoons) of added sugar per day.  * Men: No more than 36 grams (9 teaspoons) of added sugar per day.  What is "added sugar"?  Added sugar refers to sugars that are added to foods during processing or preparation, as opposed to naturally occurring sugars found in whole foods like fruits, vegetables, and dairy products. Examples of added sugars include:  * Table sugar (sucrose)  * High-fructose corn syrup  * Honey  * Maple syrup  * Agave nectar  * Fruit juice concentrates  * Cane sugar  * Maltose  * Dextrose  * Fructose  * Glucose  Why limit added sugar intake?  Consuming high amounts of added sugar has been linked to:  1. Weight gain and obesity: Excess sugar calories can lead to weight gain, which increases the risk of heart disease.  2. Insulin resistance and type 2 diabetes: Regularly consuming high amounts of added sugar can lead to insulin resistance, a precursor to type 2 diabetes.  3. Increased triglycerides and blood pressure: High sugar intake has been linked to higher triglyceride levels and blood pressure, both risk factors for heart disease.  4. Inflammation and oxidative stress: Added sugar consumption can lead to chronic inflammation and oxidative stress, which can damage blood vessels and increase heart disease risk.  Tips to limit added sugar in your diet:  1. Read food labels: Check the ingredient list for added sugars.  2. Choose whole foods: Focus on whole, unprocessed foods like fruits, vegetables, whole grains, lean proteins, and healthy fats.  3. Limit sugary drinks: Avoid sugary drinks like soda, sports drinks, and sweet tea or coffee.  4. Cook at home: Prepare meals at home using fresh ingredients to control the amount of added sugar.  5. Be mindful of hidden sources: Added sugar can be found in seemingly healthy foods like yogurt, granola, and energy bars.  By limiting your added sugar intake and focusing on a balanced diet rich in whole foods, you can help protect your heart health and reduce your risk of chronic diseases. |
| **LLaMA3+RAG model Answer 1** | According to the American Heart Association (AHA), a heart-healthy diet should limit added sugars to no more than 6% of daily calories. This translates to:  For women: no more than 100 calories per day, equivalent to about 6 teaspoons of sugar.  For men: no more than 150 calories per day, equivalent to about 9 teaspoons of sugar.  It's essential to note that these recommendations apply to all added sugars, regardless of the type, including high fructose corn syrup, honey, maple syrup, coconut sugar, and others. The AHA emphasizes that the body reacts similarly to all types of added sugars, and excessive consumption can lead to negative health effects.  The key difference lies in how quickly the sugars are absorbed by the body. Natural sugars, like those found in fruits and vegetables, are accompanied by fiber, which slows down digestion and absorption. In contrast, added sugars in foods and beverages, such as soda, are absorbed rapidly, causing a sudden spike in calorie intake.  If you're looking for a low-calorie sweetener option, plant-based sweeteners like stevia or monk fruit may be a good choice. These sweeteners are considered ""generally recognized as safe"" by the FDA, based on published research.  In summary, to maintain a heart-healthy diet, it's crucial to limit added sugars to the recommended daily amounts and be mindful of the sources of sugar in your diet. Opt for natural sources, like fruits and vegetables, and choose low-calorie sweeteners when needed.  Sources:  American Heart Association. (2024). Added Sugars. Retrieved from <https://www.heart.org/en/healthy-living/healthy-eating/eat-smart/sugar/added-sugars>  American Heart Association. (2024). How Much Sugar is Too Much? Retrieved from <https://www.heart.org/en/healthy-living/healthy-eating/eat-smart/sugar/how-much-sugar-is-too-much>  Food and Drug Administration. (2024). Generally Recognized as Safe (GRAS). Retrieved from <https://www.fda.gov/Food/IngredientsPackagingLabeling/GRAS/> |
| **LLaMA3+RAG model Answer 2** | According to the American Heart Association (AHA), a heart-healthy diet should limit added sugars to no more than 6% of daily calories. This translates to:  For most American women: no more than 100 calories per day, or about 6 teaspoons of sugar.  For men: no more than 150 calories per day, or about 9 teaspoons of sugar.  It's essential to note that the AHA recommendations focus on all added sugars, without singling out a specific type, such as high fructose corn syrup. This means that it's not just about the type of sugar, but the overall amount consumed.  Consuming high amounts of added sugars can lead to a rapid increase in calorie intake, which can have negative effects on the body. When sugar is consumed quickly, it can cause a rapid spike in blood sugar levels, followed by a crash, leaving you feeling hungry and craving more sugar. This can lead to a vicious cycle of overconsumption, contributing to weight gain, insulin resistance, and an increased risk of heart disease.  On the other hand, natural sources of sugar, such as fruits and vegetables, are accompanied by fiber, which slows down the digestion and absorption of sugar, reducing the negative effects on the body.  If you're looking for alternative sweeteners, plant-based options like stevia or monk fruit may be a good choice, as they are ""generally recognized as safe"" by the FDA and contain zero calories.  Sources:  American Heart Association. (2024). Added Sugars. Retrieved from <https://www.heart.org/en/healthy-living/healthy-eating/eat-smart/sugar/added-sugars>  American Heart Association. (2024). How Much Sugar is Too Much? Retrieved from <https://www.heart.org/en/healthy-living/healthy-eating/eat-smart/sugar/how-much-sugar-is-too-much>  Food and Drug Administration. (2024). Generally Recognized as Safe (GRAS). Retrieved from <https://www.fda.gov/Food/IngredientsPackagingLabeling/GRAS/> |
| **LLaMA3+RAG model Answer 3** | According to the American Heart Association (AHA), a heart-healthy diet should limit added sugars to no more than 6% of daily calories. This translates to a maximum of 100 calories (approximately 6 teaspoons) per day for most American women and 150 calories (approximately 9 teaspoons) per day for most American men.  It's essential to note that the AHA recommendations focus on all added sugars, without distinguishing between types such as high fructose corn syrup. This means that it's not just about avoiding one specific type of sugar, but rather limiting overall added sugar intake.  Consuming high amounts of added sugars can lead to a rapid spike in blood sugar levels, which can be detrimental to overall health. This is because the body absorbs added sugars quickly, unlike natural sugars found in whole foods like fruits, which are accompanied by fiber and absorbed more slowly.  To make smart choices, it's crucial to be aware of the amount of added sugars in foods and beverages, particularly sugary drinks like soda, which can contain as much as 10 teaspoons of sugar per 12-ounce can. Instead, opt for plant-based sweeteners like stevia or monk fruit, which are ""generally recognized as safe"" by the FDA and contain zero calories.  In summary, to maintain a heart-healthy diet, it's recommended to limit added sugars to no more than 6% of daily calories, which translates to approximately 6-9 teaspoons per day, depending on gender. Be mindful of food labels, choose whole foods whenever possible, and opt for low-calorie sweeteners when needed.  Sources:  American Heart Association. (2024). Added Sugars. Retrieved from <https://www.heart.org/en/healthy-living/healthy-eating/eat-smart/sugar/added-sugars>  American Heart Association. (2024). How Much Sugar is Too Much? Retrieved from <https://www.heart.org/en/healthy-living/healthy-eating/eat-smart/sugar/how-much-sugar-is-too-much>  Food and Drug Administration. (2024). Generally Recognized as Safe (GRAS). Retrieved from <https://www.fda.gov/Food/IngredientsPackagingLabeling/GRAS> |
| **Question 21** | **What are healthy fats to incorporate in a heart healthy diet?** |
| **Perplexity Answer 1** | Here are some healthy fats to incorporate into a heart-healthy diet:  - Monounsaturated fats like olive oil, peanut oil, canola oil, avocados, nuts and seeds, and peanut butter. These fats can help lower LDL (""bad"") cholesterol and raise HDL (""good"") cholesterol[1][2][3].  - Polyunsaturated fats like soybean oil, corn oil, safflower oil, soft tub margarines, walnuts, flax seed, sunflower seeds. These fats can also help lower LDL cholesterol[1][2][3].  - Omega-3 fatty acids found in fatty fish like salmon, mackerel, tuna, sardines, and herring. Omega-3s help improve heart health by reducing plaque buildup in arteries and decreasing triglycerides[2][3][5].  - Avocados, olives, nuts and seeds are all good sources of healthy fats[3][4].  To get more of these healthy fats:  - Cook with olive oil, canola oil or other nontropical vegetable oils lower in saturated fat[3][5]  - Have fish at least twice a week[1]  - Snack on a 1-ounce serving of nuts[2]  - Add ground flax seed to foods like yogurt, cereal and smoothies[1]  - Use avocado or nut butters instead of butter or mayo[2][4]  The key is to replace saturated and trans fats with these healthier unsaturated fats to help improve cholesterol levels and reduce heart disease risk[3][4][5].  Citations:  [1] https://www.pardeehospital.org/app/files/public/d9443cc7-7a33-4f38-8038-4437816f1082/cardiosmart-heart-healty-diet.pdf  [2] https://www.in.gov/spd/files/HHE-HeartHealthyFats.pdf  [3] https://www.heartfoundation.org.au/healthy-living/healthy-eating/fats-oils-and-heart-health  [4] https://health.gov/myhealthfinder/health-conditions/heart-health/heart-healthy-foods-shopping-list  [5] https://www.heart.org/en/healthy-living/healthy-eating/eat-smart/fats/4-ways-to-get-good-fats-infographic |
| **Perplexity Answer 2** | Here are some healthy fats to incorporate into a heart-healthy diet:  Monounsaturated Fats  - Olive oil, canola oil, peanut oil[1][3][4][5]  - Avocados[3][4]  - Nuts and seeds like almonds, cashews, peanuts[3][4]  - Olives and peanut butter[3][4]  Polyunsaturated Fats  - Soybean oil, corn oil, safflower oil[1][3][4][5]  - Walnuts, flax seeds, sunflower seeds[2][3]  - Fatty fish like salmon, mackerel, tuna, sardines, herring[2][3]  Omega-3 Fatty Acids  - Fatty fish like salmon, mackerel, tuna, sardines, herring[2][3]  - Walnuts[2][3]  - Ground flaxseed and flaxseed oil[1][2]  To get more of these healthy fats:  - Cook with olive oil or canola oil instead of butter[1][4]  - Have fish twice a week[1]  - Add ground flaxseed to cereals, soups, and smoothies[1]  - Snack on a 1-ounce serving of nuts[2]  - Use avocado instead of mayonnaise or butter on sandwiches[2]  Replacing saturated and trans fats with these healthier unsaturated fats can help improve cholesterol levels and lower heart disease risk[3][5]. Focus on getting a balance of fats from whole food sources as part of an overall heart-healthy eating pattern[3].  Citations:  [1] https://www.pardeehospital.org/app/files/public/d9443cc7-7a33-4f38-8038-4437816f1082/cardiosmart-heart-healty-diet.pdf  [2] https://www.in.gov/spd/files/HHE-HeartHealthyFats.pdf  [3] https://www.heartfoundation.org.au/healthy-living/healthy-eating/fats-oils-and-heart-health  [4] https://health.gov/myhealthfinder/health-conditions/heart-health/heart-healthy-foods-shopping-list  [5] https://www.heart.org/en/healthy-living/healthy-eating/eat-smart/fats/4-ways-to-get-good-fats-infographic |
| **Perplexity Answer 3** | Here are some healthy fats to incorporate into a heart-healthy diet:  Monounsaturated Fats  - Olive oil, canola oil, peanut oil[1][3][4][5]  - Avocados[3][4][5]  - Nuts and seeds like almonds, cashews, peanuts[3][4][5]  - Peanut butter[1][4]  Polyunsaturated Fats  - Soybean oil, corn oil, safflower oil[1][3][4][5]  - Walnuts[1][3][4]  - Flaxseeds and flaxseed oil[1][3]  - Sunflower seeds[3]  Omega-3 Fatty Acids  - Fatty fish like salmon, mackerel, tuna, sardines, herring[1][3][4]  - Walnuts[1][3]  - Ground flaxseed[1][3]  - Flaxseed oil[3]  To get more of these healthy fats:  - Cook with olive oil or canola oil instead of butter[4][5]  - Have fish like salmon twice a week[1]  - Add ground flaxseed to cereals, soups, and smoothies[1]  - Snack on a 1-ounce serving of nuts[4]  - Use avocado instead of mayo or butter on sandwiches[4]  Replacing saturated and trans fats with these healthier unsaturated fats can help improve cholesterol levels and lower heart disease risk[3][5]. Focus on getting a balance of fats from whole foods as part of an overall heart-healthy eating pattern[3].  Citations:  [1] https://www.pardeehospital.org/app/files/public/d9443cc7-7a33-4f38-8038-4437816f1082/cardiosmart-heart-healty-diet.pdf  [2] https://www.in.gov/spd/files/HHE-HeartHealthyFats.pdf  [3] https://www.heartfoundation.org.au/healthy-living/healthy-eating/fats-oils-and-heart-health  [4] https://health.gov/myhealthfinder/health-conditions/heart-health/heart-healthy-foods-shopping-list  [5] https://www.heart.org/en/healthy-living/healthy-eating/eat-smart/fats/4-ways-to-get-good-fats-infographic |
| **GPT4o Answer 1** | Incorporating healthy fats into a heart-healthy diet can help reduce the risk of cardiovascular disease and improve overall health. Here are some sources of healthy fats:  1. Monounsaturated Fats:  - Olive Oil: Rich in monounsaturated fats, it can help lower bad cholesterol levels.  - Avocados: High in heart-healthy monounsaturated fats and fiber.  - Nuts and Seeds: Almonds, cashews, peanuts, and sesame seeds are excellent sources.  2. Polyunsaturated Fats:  - Fatty Fish: Salmon, mackerel, sardines, and trout are high in omega-3 fatty acids, which reduce inflammation and the risk of heart disease.  - Walnuts: Contain alpha-linolenic acid (ALA), a type of omega-3 fatty acid.  - Flaxseeds and Chia Seeds: Both are rich in omega-3 fatty acids and fiber.  3. Omega-3 Fatty Acids:  - Fish Oil: Supplements can be an alternative if fatty fish is not regularly consumed.  - Algae Oil: A plant-based source of omega-3s, suitable for vegetarians and vegans.  4. Other Healthy Fat Sources:  - Soybeans and Tofu: Provide polyunsaturated fats and are versatile in many recipes.  - Dark Chocolate: Contains healthy fats and antioxidants, but should be consumed in moderation.  - Eggs: Provide a good balance of saturated and unsaturated fats along with protein.  Tips for Including Healthy Fats:  - Cooking: Use olive oil or avocado oil for cooking and salad dressings.  - Snacking: Choose nuts and seeds as snacks instead of processed snacks.  - Meals: Incorporate fatty fish into meals at least twice a week.  - Substitution: Replace butter and margarine with healthier fat options like nut butters or avocado.  By incorporating these healthy fats into your diet, you can support heart health and overall wellness. Remember to balance fat intake with a variety of nutrient-dense foods, including plenty of fruits, vegetables, whole grains, and lean proteins. |
| **GPT4o Answer 2** | Incorporating healthy fats into a heart-healthy diet is essential for maintaining overall cardiovascular health. Here are some sources of healthy fats:  1. Olive Oil: Rich in monounsaturated fats, olive oil can help reduce bad cholesterol levels and is a staple in the Mediterranean diet.  2. Avocados: Avocados are high in monounsaturated fats and provide potassium, fiber, and vitamins.  3. Nuts: Almonds, walnuts, pecans, and pistachios are excellent sources of healthy fats, protein, and fiber. Walnuts, in particular, are rich in omega-3 fatty acids.  4. Seeds: Chia seeds, flaxseeds, and hemp seeds are good sources of omega-3 fatty acids, fiber, and protein.  5. Fatty Fish: Salmon, mackerel, sardines, and trout are rich in omega-3 fatty acids, which are beneficial for heart health.  6. Dark Chocolate: In moderation, dark chocolate (with at least 70% cocoa) can provide healthy fats and antioxidants.  7. Coconut Oil: While it is high in saturated fats, some studies suggest that it can be part of a healthy diet when used in moderation and balanced with other healthy fats.  8. Nut Butters: Peanut butter, almond butter, and other nut butters are good sources of healthy fats, as long as they don’t contain added sugars or hydrogenated oils.  9. Tofu and Soy Products: These are good sources of polyunsaturated fats and can be a heart-healthy alternative to animal products.  10. Eggs: Eggs, particularly the yolks, contain healthy fats and important nutrients like choline.  When incorporating these fats into your diet, it's important to focus on balance and moderation, while also considering overall dietary patterns that emphasize whole, minimally processed foods. |
| **GPT4o Answer 3** | Incorporating healthy fats into a heart-healthy diet is essential for maintaining cardiovascular health. Here are some of the best sources of healthy fats:  1. Monounsaturated Fats  - Olive Oil: Extra virgin olive oil is a great source of monounsaturated fats.  - Avocados: These are high in monounsaturated fats and also provide fiber.  - Nuts: Almonds, cashews, and peanuts are good sources.  - Seeds: Sesame seeds and pumpkin seeds are beneficial.  2. Polyunsaturated Fats  - Fatty Fish: Salmon, mackerel, sardines, and trout are rich in omega-3 fatty acids.  - Walnuts: These are an excellent source of omega-3 fatty acids.  - Flaxseeds: Ground flaxseeds and flaxseed oil provide omega-3s.  - Chia Seeds: High in omega-3 fatty acids, fiber, and protein.  - Soybeans and Tofu: These are good plant-based sources of polyunsaturated fats.  3. Omega-3 Fatty Acids  - Fish Oil: Fish oil supplements can be considered if dietary intake is insufficient.  - Algal Oil: A plant-based omega-3 supplement derived from algae, suitable for vegetarians and vegans.  4. Omega-6 Fatty Acids  - Vegetable Oils: Sunflower, safflower, and soybean oils are rich in omega-6 fatty acids.  - Nuts and Seeds: Many nuts and seeds also provide omega-6 fatty acids.  5. Other Sources of Healthy Fats  - Dark Chocolate: Contains a small amount of healthy fats along with antioxidants.  - Coconut Oil: In moderation, coconut oil can be a good source of healthy fats, though it should not replace other healthier fat sources.  Tips for Incorporation  - Cooking: Use olive oil for cooking and dressings.  - Snacking: Include a handful of nuts as a snack.  - Meals: Add avocados to salads, sandwiches, and smoothies.  - Supplements: Consider fish oil or algal oil supplements if you're not consuming enough omega-3-rich foods.  Incorporating these sources of healthy fats can help reduce bad cholesterol levels, increase good cholesterol levels, and lower the risk of heart disease. Always aim for balance and moderation, and consult with a healthcare professional before making significant changes to your diet. |
| **Llama 3 Answer 1** | Healthy fats are an essential part of a heart-healthy diet, as they can help lower cholesterol levels, improve blood lipid profiles, and reduce the risk of heart disease. Here are some healthy fats to incorporate into your diet:  1. Monounsaturated Fats (MUFAs):  * Avocados: rich in oleic acid, which can help lower LDL (bad) cholesterol.  * Olive oil: use it as a finishing oil for cooking, dressings, and marinades.  * Nuts and seeds: almonds, cashews, pumpkin seeds, and sesame seeds are all good sources.  2. Polyunsaturated Fats (PUFAs):  * Fatty fish: salmon, tuna, mackerel, and sardines are rich in omega-3 fatty acids, which can help reduce inflammation and improve heart health.  * Walnuts: a rich source of alpha-linolenic acid (ALA), an omega-3 fatty acid.  * Chia seeds: a good source of ALA and fiber.  3. Omega-3 Fatty Acids:  * Flaxseeds: rich in ALA, which can help lower triglycerides and blood pressure.  * Hemp seeds: a good source of ALA and gamma-linolenic acid (GLA), an omega-6 fatty acid.  4. Medium-Chain Triglycerides (MCTs):  * Coconut oil: contains lauric acid, a medium-chain fatty acid with antimicrobial properties.  * Full-fat dairy: choose grass-fed, full-fat dairy products like milk, cheese, and yogurt.  5. Other Healthy Fats:  * Ghee: a type of clarified butter that's rich in fat-soluble vitamins and conjugated linoleic acid (CLA), which may help improve heart health.  * Dark chocolate: contains flavonoids and healthy fats like oleic acid and stearic acid. Opt for dark chocolate with at least 70% cocoa solids.  Remember to consume these healthy fats in moderation, as they are still high in calories. The American Heart Association recommends that adults get 20-35% of their daily calories from fat, with an emphasis on unsaturated fats.  Tips for incorporating healthy fats into your diet:  1. Use olive oil or avocado oil for cooking and dressings.  2. Snack on nuts and seeds, like almonds, cashews, and pumpkin seeds.  3. Add fatty fish to your meals 2-3 times a week.  4. Use coconut oil or ghee for sautéing or baking.  5. Top your oatmeal or yogurt with chia seeds, flaxseeds, or hemp seeds.  6. Enjoy dark chocolate in moderation as a sweet treat.  By incorporating these healthy fats into your diet, you'll be well on your way to a heart-healthy lifestyle! |
| **Llama 3 Answer 2** | Healthy fats are an essential part of a heart-healthy diet, and they can provide numerous benefits when consumed in moderation. Here are some healthy fats to incorporate into your diet:  1. Monounsaturated Fats (MUFAs):  * Avocados: Rich in potassium, vitamins, and minerals, avocados can help lower LDL (bad) cholesterol and improve heart health.  * Olive oil: Use it for cooking, dressings, or as a finishing oil for dishes. It's rich in antioxidants and can help lower total cholesterol and LDL cholesterol.  * Nuts and seeds: Almonds, cashews, pumpkin seeds, and sesame seeds are all good sources of MUFAs.  2. Polyunsaturated Fats (PUFAs):  * Fatty fish: Fatty fish like salmon, sardines, and mackerel are rich in omega-3 PUFAs, which can help lower triglycerides and blood pressure.  * Walnuts: These nuts are a rich source of alpha-linolenic acid (ALA), a type of omega-3 fatty acid.  * Chia seeds: These tiny seeds are a good source of ALA and can help lower blood pressure and triglycerides.  3. Omega-3 Fatty Acids:  * Flaxseeds: Rich in ALA, flaxseeds can help lower inflammation and improve heart health.  * Hemp seeds: These seeds are a good source of ALA and gamma-linolenic acid (GLA), an omega-6 fatty acid.  4. Saturated Fats (in moderation):  * Coconut oil: While saturated fats are generally considered less healthy, coconut oil contains medium-chain triglycerides (MCTs) that may have benefits for heart health. Use it in moderation.  * Full-fat dairy: Choose low-fat or fat-free dairy products most of the time, but occasional consumption of full-fat dairy products like cheese and whole milk can provide benefits.  5. Other Healthy Fats:  * Ghee: Clarified butter from grass-fed cows, ghee is a healthy alternative to regular butter.  * Dark chocolate: Containing flavonoids and healthy fats, dark chocolate can help improve heart health and lower blood pressure. Choose dark chocolate with at least 70% cocoa solids.  Remember to consume these healthy fats in moderation, as excessive intake can lead to weight gain and other health issues. The American Heart Association recommends that adults limit their daily intake of saturated fats to no more than 5-6% of total daily calories.  Here are some general guidelines for incorporating healthy fats into your diet:  * Nuts and seeds: 1 ounce (28g) per day  * Fatty fish: 2 servings (about 8 ounces or 225g) per week  * Avocado: 1/2 to 1 medium avocado per day  * Olive oil: 1-2 tablespoons (15-30mL) per day  * Coconut oil: 1-2 tablespoons (15-30mL) per day (in moderation)  By incorporating these healthy fats into your diet, you can help improve your heart health and overall well-being. |
| **Llama 3 Answer 3** | Healthy fats are an essential part of a heart-healthy diet. They can help lower cholesterol levels, reduce inflammation, and provide sustained energy. Here are some healthy fats to incorporate into your diet:  1. Monounsaturated Fats (MUFAs):  * Avocados: Rich in vitamins, minerals, and antioxidants, avocados are a great source of MUFAs.  * Nuts and seeds: Almonds, cashews, pumpkin seeds, and sesame seeds are all high in MUFAs.  * Olive oil: Use it as a finishing oil for cooking, dressings, and marinades.  2. Polyunsaturated Fats (PUFAs):  * Fatty fish: Fatty fish like salmon, tuna, and mackerel are rich in omega-3 PUFAs, which reduce inflammation and improve heart health.  * Walnuts: These nuts are a rich source of alpha-linolenic acid (ALA), a type of omega-3 PUFA.  * Chia seeds: These tiny seeds are packed with ALA and fiber.  3. Saturated Fats (SFAs) in moderation:  * Coconut oil: While saturated, coconut oil contains medium-chain triglycerides (MCTs) that have potential health benefits.  * Full-fat dairy: Choose full-fat dairy products like cheese, yogurt, and milk from grass-fed cows.  * Grass-fed beef: Opt for lean cuts and consume in moderation.  4. Omega-6 Fatty Acids:  * Nuts and seeds: While omega-6s can be pro-inflammatory in excess, moderate amounts from nuts and seeds like sunflower seeds, pumpkin seeds, and flaxseeds are beneficial.  * Vegetable oils: Use moderate amounts of oils like grapeseed, safflower, and sunflower oil for cooking and dressings.  Incorporate these healthy fats into your diet by:  * Adding nuts and seeds to oatmeal, yogurt, or salads  * Using olive oil, avocado oil, or grapeseed oil for cooking and dressings  * Eating fatty fish 2-3 times a week  * Snacking on avocado slices or guacamole  * Adding chia seeds or flaxseeds to smoothies or oatmeal  * Choosing full-fat dairy products and grass-fed beef in moderation  Remember to always consume these healthy fats in moderation as part of a balanced diet. Excessive fat consumption, even from healthy sources, can lead to weight gain and other health issues. |
| **LLaMA3+RAG model Answer 1** | Healthy fats are an essential component of a heart-healthy diet, and incorporating the right types of fats can have numerous benefits for cardiovascular health. According to the American Heart Association (AHA), healthy fats can be found in various food sources, including:  1. Nontropical vegetable oils: These oils are rich in unsaturated fats, which can help lower total cholesterol and LDL (""bad"") cholesterol levels. Examples of nontropical vegetable oils include olive oil, canola oil, and avocado oil.  2. Nuts and seeds: Nuts and seeds are rich in healthy fats, protein, and fiber. Almonds, walnuts, chia seeds, and flaxseeds are all excellent sources of healthy fats.  3. Fatty fish: Fatty fish like salmon, tuna, and mackerel are rich in omega-3 fatty acids, which have been shown to reduce inflammation and improve heart health.  4. Avocados: Avocados are a rich source of monounsaturated fats, which can help lower LDL cholesterol levels and improve heart health.  5. Fat-free or low-fat dairy products: While the AHA recommends limiting dairy fats, fat-free or low-fat dairy products like milk, yogurt, and cheese can be a good source of healthy fats. Look for products with no more than 3 grams of fat per ounce and no more than 1 gram of saturated fat per ounce.  Incorporating these healthy fats into your diet can have numerous benefits for heart health, including:  Lowering total cholesterol and LDL cholesterol levels  Reducing inflammation and improving blood vessel function  Improving blood lipid profiles  Reducing the risk of heart disease and stroke  Remember to always consume healthy fats in moderation as part of a balanced diet. The AHA recommends limiting daily fat intake to 20-35% of total daily calories, with an emphasis on unsaturated fats.  Sources:  American Heart Association. (2024). How Do I Follow a Healthy Diet? Retrieved from <https://www.heart.org/-/media/files/health-topics/answers-by-heart/how-do-i-follow-a-healthy-diet.pdf>  American Heart Association. (2024). Dairy Products, Milk, Yogurt, and Cheese. Retrieved from <https://www.heart.org/en/healthy-living/healthy-eating/eat-smart/nutrition-basics/dairy-products-milk-yogurt-and-cheese> |
| **LLaMA3+RAG model Answer 2** | Healthy fats are an essential component of a heart-healthy diet, and incorporating the right types can have numerous benefits for overall health. According to the American Heart Association (AHA), healthy fats can be found in various food sources, including:  1. Nontropical vegetable oils: These oils are rich in healthy fats and can be used for cooking, dressing salads, or making sauces. Examples include olive oil, canola oil, and avocado oil.  2. Nuts and seeds: Almonds, walnuts, chia seeds, flaxseeds, and pumpkin seeds are all rich in healthy fats and can be added to meals or snacks.  3. Fatty fish: Fatty fish like salmon, tuna, and mackerel are high in omega-3 fatty acids, which are beneficial for heart health.  4. Avocados: Avocados are a rich source of monounsaturated fats, which can help lower cholesterol levels and improve heart health.  5. Fat-free or low-fat dairy products: While the AHA recommends limiting dairy fats, fat-free or low-fat dairy products like milk, yogurt, and cheese can be a good source of healthy fats.  When choosing healthy fats, it's essential to be mindful of the amount consumed, as even healthy fats are high in calories. The AHA recommends limiting daily intake of saturated and trans fats, which can increase the risk of heart disease.  Incorporating healthy fats into your diet can be as simple as:  Adding nuts or seeds to your oatmeal or yogurt  Using avocado oil or olive oil for cooking  Incorporating fatty fish into your meals 2-3 times a week  Snacking on avocado slices or guacamole  Choosing fat-free or low-fat dairy products  Remember to always consume healthy fats in moderation and as part of a balanced diet that emphasizes whole, nutrient-dense foods.  Sources:  1. American Heart Association. (2024). How Do I Follow a Healthy Diet? Retrieved from <https://www.heart.org/-/media/files/health-topics/answers-by-heart/how-do-i-follow-a-healthy-diet.pdf>  2. American Heart Association. (2024). Dairy Products, Milk, Yogurt, and Cheese. Retrieved from <https://www.heart.org/en/healthy-living/healthy-eating/eat-smart/nutrition-basics/dairy-products-milk-yogurt-and-cheese>  3. American Heart Association. (2024). Healthy Eating Plate. Retrieved from <https://www.heart.org/en/healthy-living/healthy-eating/eat-smart/nutrition-basics/healthy-eating-plate> |
| **LLaMA3+RAG model Answer 3** | Healthy fats are an essential component of a heart-healthy diet, and incorporating the right types of fats can have a significant impact on cardiovascular health. According to the American Heart Association (AHA), healthy fats to incorporate into your diet include:  1. Nontropical vegetable oils: These oils are rich in unsaturated fats, which can help lower total cholesterol and LDL (""bad"") cholesterol levels. Examples of nontropical vegetable oils include olive oil, canola oil, and grapeseed oil.  2. Nuts and seeds: Nuts and seeds are rich in healthy fats, protein, and fiber. Almonds, walnuts, chia seeds, and flaxseeds are all great options to incorporate into your diet.  3. Fatty fish: Fatty fish like salmon, tuna, and mackerel are rich in omega-3 fatty acids, which have been shown to reduce inflammation and improve heart health.  4. Avocados: Avocados are a rich source of monounsaturated fats, which can help lower cholesterol levels and improve heart health.  5. Fat-free or low-fat dairy products: While the AHA recommends limiting dairy fat intake, fat-free or low-fat dairy products like milk, yogurt, and cheese can still be a healthy source of fat in moderation.  It's essential to remember to consume these healthy fats in moderation, as excessive fat intake can still contribute to weight gain and other health problems. The AHA recommends limiting daily fat intake to 20-35% of total daily calories.  In contrast, it's recommended to limit or avoid unhealthy fats, such as:  1. Saturated fats: Found in whole milk, full-fat cheeses, and processed meats, saturated fats can raise LDL cholesterol levels and increase the risk of heart disease.  2. Trans fats: Found in processed foods and baked goods, trans fats can increase the risk of heart disease by raising LDL cholesterol levels and lowering HDL (""good"") cholesterol levels  By incorporating healthy fats into your diet and limiting unhealthy fats, you can help reduce your risk of heart disease and maintain a healthy cardiovascular system.  Sources:  1. American Heart Association. (2024). How Do I Follow a Healthy Diet? Retrieved from <https://www.heart.org/-/media/files/health-topics/answers-by-heart/how-do-i-follow-a-healthy-diet.pdf>  2. American Heart Association. (2024). Dairy Products, Milk, Yogurt, and Cheese. Retrieved from <https://www.heart.org/en/healthy-living/healthy-eating/eat-smart/nutrition-basics/dairy-products-milk-yogurt-and-cheese>  3. National Institutes of Health. (2020). Omega-3 Fatty Acids. Retrieved from <https://ods.od.nih.gov/factsheets/Omega3FattyAcids-HealthProfessional/> |
| **Question 22** | **How many calories should I be eating per day to support a heart healthy diet?** |
| **Perplexity Answer 1** | To determine the appropriate number of calories to eat per day for a heart-healthy diet, several factors need to be considered, such as your age, gender, and activity level. However, some general guidelines can be provided:  - The American Heart Association recommends using up at least as many calories as you take in to maintain a healthy weight[3].  - Nutrition and calorie information on food labels is typically based on a 2,000 calorie per day diet, but you may need fewer or more calories depending on your individual needs[3].  - One effective way to begin a heart-healthy diet is by creating a daily meal plan aiming for a balanced diet rich in lean protein, vegetables, fruits, and whole grains, while limiting high-fat foods, sodium-laden items, and added sugars[4].  - Example heart-healthy meal plans provided by Mayo Clinic contain around 1,600-1,700 calories per day[4].  So in summary, while the exact number of calories will vary, a general target for a heart-healthy diet is to consume enough calories to maintain a healthy weight, while emphasizing nutrient-dense foods like fruits, vegetables, whole grains and lean proteins. Consulting with a healthcare professional can help determine your specific calorie needs.  Citations:  [1] https://www.eatingwell.com/article/289245/7-day-heart-healthy-meal-plan-1200-calories/  [2] https://www.eatingwell.com/article/7884046/heart-healthy-diet-plan-for-beginners/  [3] https://www.heart.org/en/healthy-living/healthy-eating/eat-smart/nutrition-basics/aha-diet-and-lifestyle-recommendations  [4] https://www.mayoclinic.org/diseases-conditions/heart-disease/in-depth/heart-healthy-diet/art-20046702  [5] https://www.mayoclinic.org/diseases-conditions/heart-disease/in-depth/heart-healthy-diet/art-20047702 |
| **Perplexity Answer 2** | To determine the number of calories you should be eating per day for a heart-healthy diet, consider the following:  - The American Heart Association recommends starting by knowing how many calories you should be eating to maintain your weight, which is typically based on a 2,000 calorie per day diet. However, you may need fewer or more calories depending on factors like age, gender, and physical activity level[2].  - The Mayo Clinic provides two sample 1-day heart-healthy meal plans - one with 1,688 calories and one with 1,621 calories[3]. This suggests a range of 1,600-1,700 calories per day can be appropriate for a heart-healthy diet.  - For weight loss, the Mayo Clinic recommends controlling portion sizes and eating more low-calorie, nutrient-rich foods like fruits and vegetables while limiting high-calorie, high-sodium processed foods[4].  - Limiting added sugars to less than 10% of daily calories is also recommended. For a 2,000 calorie diet, that's about 50 grams of added sugar[4].  In summary, while calorie needs vary individually, a heart-healthy diet typically falls in the range of 1,600-2,000 calories per day, with an emphasis on nutrient-dense whole foods and limited added sugars and processed foods. Consulting with a registered dietitian can help determine your specific calorie and nutrient needs.  Citations:  [1] https://www.eatingwell.com/article/289245/7-day-heart-healthy-meal-plan-1200-calories/  [2] https://www.heart.org/en/healthy-living/healthy-eating/eat-smart/nutrition-basics/aha-diet-and-lifestyle-recommendations  [3] https://www.mayoclinic.org/diseases-conditions/heart-disease/in-depth/heart-healthy-diet/art-20046702  [4] https://www.mayoclinic.org/diseases-conditions/heart-disease/in-depth/heart-healthy-diet/art-20047702  [5] https://www.mountsinai.org/health-library/report/heart-healthy-diet |
| **Perplexity Answer 3** | To determine the appropriate number of calories to consume for a heart-healthy diet, several factors need to be considered, such as age, gender, and physical activity level[2]. The American Heart Association recommends starting by knowing how many calories you should be eating and drinking to maintain your weight[2].  Nutrition and calorie information on food labels is typically based on a 2,000 calorie per day diet[2]. However, you may need fewer or more calories depending on your individual needs[2].  The Mayo Clinic provides two sample menus for heart-healthy eating, which provide around 1,600-1,700 calories per day[3]. These menus are intended as examples and inspiration for creating your own heart-healthy eating plan[3].  It's important to note that the optimal calorie intake can vary widely from person to person. The key is to aim for an overall healthy dietary pattern that emphasizes fruits, vegetables, whole grains, lean protein, and healthy fats, while minimizing added sugars, sodium, and saturated fat[2][4][5]. Portion control is also crucial for maintaining a healthy calorie balance[5].  If you are unsure of your specific calorie needs, it's best to consult with a healthcare professional or registered dietitian to determine the right calorie target for you based on your individual health status, goals, and lifestyle.  Citations:  [1] https://www.eatingwell.com/article/289245/7-day-heart-healthy-meal-plan-1200-calories/  [2] https://www.heart.org/en/healthy-living/healthy-eating/eat-smart/nutrition-basics/aha-diet-and-lifestyle-recommendations  [3] https://www.mayoclinic.org/diseases-conditions/heart-disease/in-depth/heart-healthy-diet/art-20046702  [4] https://www.nhlbi.nih.gov/health/educational/healthdisp/pdf/tipsheets/Heart-Healthy-Eating-Plan.pdf  [5] https://www.mayoclinic.org/diseases-conditions/heart-disease/in-depth/heart-healthy-diet/art-20047702 |
| **GPT4o Answer 1** | The number of calories you should consume each day to support a heart-healthy diet depends on various factors including your age, gender, weight, height, and activity level. However, general guidelines can help you understand the average calorie needs:  Average Calorie Needs:  1. Men:  - Sedentary: 2,000-2,400 calories per day  - Moderately active: 2,200-2,800 calories per day  - Active: 2,400-3,000 calories per day  2. Women:  - Sedentary: 1,600-2,000 calories per day  - Moderately active: 1,800-2,200 calories per day  - Active: 2,000-2,400 calories per day  Heart-Healthy Diet Recommendations:  To support heart health, consider focusing on the following dietary principles:  - Balance Calories: Ensure you are not consuming more calories than you burn to maintain a healthy weight.  - Eat Nutrient-Dense Foods: Focus on foods that provide vitamins, minerals, fiber, and other nutrients without too many calories. These include fruits, vegetables, whole grains, lean proteins, and healthy fats.  - Limit Saturated and Trans Fats: Choose lean meats, low-fat dairy products, and healthy oils like olive oil. Avoid trans fats found in some processed and fried foods.  - Choose Whole Grains: Whole grains provide more nutrients and fiber than refined grains. Opt for whole wheat bread, brown rice, quinoa, and oats.  - Eat Plenty of Fruits and Vegetables: Aim for a variety of colors and types to ensure a broad range of nutrients.  - Reduce Sodium: Too much sodium can increase blood pressure. Try to limit processed foods and choose low-sodium options.  - Moderate Sugar Intake: Limit sugary beverages and snacks. Opt for natural sources of sweetness like fruits.  Personalized Recommendations:  To get a more accurate estimation tailored to your specific needs, consider using a calorie calculator that factors in your personal details, or consult with a healthcare provider or a registered dietitian. They can provide individualized guidance based on your health status and goals.  Would you like help calculating your specific daily calorie needs? If so, please provide your age, gender, weight, height, and activity level. |
| **GPT4o Answer 2** | The number of calories you should consume daily to support a heart-healthy diet depends on several factors including your age, sex, weight, height, and activity level. Here are some general guidelines based on the Dietary Guidelines for Americans 2020-2025:  1. Adult Women:  - Sedentary (little or no physical activity): 1,800-2,000 calories per day  - Moderately active (moderate physical activity such as walking 1.5 to 3 miles per day): 2,000-2,200 calories per day  - Active (physical activity equivalent to walking more than 3 miles per day): 2,200-2,400 calories per day  2. Adult Men:  - Sedentary: 2,200-2,400 calories per day  - Moderately active: 2,400-2,800 calories per day  - Active: 2,800-3,000 calories per day  Components of a Heart-Healthy Diet  In addition to calorie intake, the quality of the diet is crucial for heart health. Here are some key components:  1. Fruits and Vegetables: Aim for a variety of colors and types, at least 4.5 cups per day.  2. Whole Grains: At least half of your grain intake should be whole grains. Aim for three 1-ounce servings per day.  3. Protein Sources: Prefer lean protein sources such as poultry, fish, legumes, nuts, and seeds. Fish should be consumed at least twice a week.  4. Dairy: Choose low-fat or fat-free options.  5. Fats: Focus on healthy fats such as those from olive oil, avocados, and nuts. Limit saturated fats and avoid trans fats.  6. Sodium: Limit sodium intake to less than 2,300 mg per day, ideally 1,500 mg for better heart health.  7. Added Sugars: Keep added sugars to a minimum, ideally no more than 10% of your daily calorie intake.  Personalized Recommendations  To get a more accurate estimate tailored to your specific needs, consider using a calorie calculator that takes into account your personal details and activity level. Additionally, consulting with a healthcare provider or a registered dietitian can provide personalized recommendations that align with your health goals.  Would you like help with calculating your specific calorie needs or further information on heart-healthy foods? |
| **GPT4o Answer 3** | The number of calories you should eat per day to support a heart-healthy diet depends on several factors, including your age, gender, weight, height, and level of physical activity. Additionally, individual health conditions and specific dietary needs play a role. However, general guidelines can help you determine an appropriate calorie range for maintaining a heart-healthy lifestyle.  General Caloric Intake Guidelines  1. Adult Women:  - Sedentary: 1,800 to 2,000 calories per day  - Moderately Active: 2,000 to 2,200 calories per day  - Active: 2,200 to 2,400 calories per day  2. Adult Men:  - Sedentary: 2,200 to 2,400 calories per day  - Moderately Active: 2,400 to 2,800 calories per day  - Active: 2,800 to 3,000 calories per day  Key Components of a Heart-Healthy Diet  To support heart health, focus on the following dietary components:  1. Fruits and Vegetables: Aim for a variety of colors and types to ensure a range of nutrients.  2. Whole Grains: Choose whole grain products like brown rice, whole wheat bread, and oatmeal.  3. Lean Proteins: Opt for lean meats, poultry, fish, beans, and legumes.  4. Healthy Fats: Incorporate sources of unsaturated fats such as avocados, nuts, seeds, and olive oil. Limit saturated and trans fats.  5. Low-Fat Dairy: Choose low-fat or fat-free dairy products.  6. Limit Added Sugars and Sodium: Reduce intake of sugary drinks, sweets, and processed foods high in salt.  Example Meal Plan  Here's an example of how you might structure your meals throughout the day:  - Breakfast:  - Oatmeal with fresh berries and a handful of nuts  - A glass of low-fat milk or a dairy alternative  - Lunch:  - Grilled chicken salad with a variety of vegetables and a vinaigrette dressing  - A slice of whole-grain bread  - Dinner:  - Baked salmon with a side of quinoa and steamed broccoli  - A small side salad with mixed greens  - Snacks:  - Fresh fruit (like an apple or a banana)  - A small handful of almonds or walnuts  - Carrot sticks with hummus  Personalized Recommendations  For more personalized recommendations, consider consulting with a registered dietitian or a healthcare provider. They can provide a tailored plan based on your specific health needs and goals. Additionally, using tools like the Dietary Guidelines for Americans and resources from organizations such as the American Heart Association can provide further guidance on maintaining a heart-healthy diet. |
| **Llama 3 Answer 1** | A heart-healthy diet focuses on consuming nutrient-dense foods that help maintain a healthy weight, blood pressure, cholesterol levels, and blood lipid profiles. The ideal daily calorie intake varies based on factors such as age, sex, weight, height, and physical activity level. Here are some general guidelines:  Estimated Daily Calorie Needs for a Heart-Healthy Diet:  * Sedentary (little or no exercise):  + Women: 1,600-2,000 calories/day  + Men: 2,000-2,400 calories/day  * Lightly active (light exercise/sports 1-3 days/week):  + Women: 1,800-2,200 calories/day  + Men: 2,200-2,800 calories/day  * Moderately active (moderate exercise/sports 3-5 days/week):  + Women: 2,000-2,400 calories/day  + Men: 2,400-3,000 calories/day  * Very active (hard exercise/sports 6-7 days a week):  + Women: 2,200-2,800 calories/day  + Men: 2,800-3,500 calories/day  * Extremely active (very hard exercise/sports & physical job or 2x training):  + Women: 2,400-3,000 calories/day  + Men: 3,000-4,000 calories/day  Additional Tips:  1. Focus on nutrient-dense foods: Prioritize whole, unprocessed foods like fruits, vegetables, whole grains, lean proteins, and healthy fats. These foods provide essential vitamins, minerals, and antioxidants that support heart health.  2. Watch portion sizes: Even healthy foods can lead to weight gain and negatively impact heart health if consumed in excess.  3. Limit added sugars, saturated and trans fats, and sodium: Try to limit or avoid foods high in these nutrients, as they can increase the risk of heart disease.  4. Stay hydrated: Drink plenty of water and limit sugary drinks.  5. Consult with a healthcare professional or registered dietitian: They can help you determine your individual calorie needs and create a personalized heart-healthy diet plan.  Remember, a heart-healthy diet is just one aspect of maintaining overall cardiovascular well-being. Regular physical activity, stress management, and not smoking are also crucial for a healthy heart. |
| **Llama 3 Answer 2** | The recommended daily calorie intake to support a heart-healthy diet varies based on several factors, including your age, gender, weight, height, and activity level. Here are some general guidelines from the American Heart Association (AHA):  For adults:  * Sedentary (little or no exercise): 1,600-2,000 calories/day  * Lightly active (light exercise/sports 1-3 days/week): 1,800-2,200 calories/day  * Moderately active (moderate exercise/sports 3-5 days/week): 2,000-2,400 calories/day  * Very active (hard exercise/sports 6-7 days a week): 2,200-2,800 calories/day  * Extremely active (very hard exercise/sports & physical job or 2x training): 2,400-3,000 calories/day  For older adults (65 years and older):  * Sedentary: 1,400-1,800 calories/day  * Lightly active: 1,600-2,000 calories/day  * Moderately active: 1,800-2,200 calories/day  Additional considerations:  * If you're trying to lose weight, you may need to reduce your daily calorie intake by 500-1000 calories to promote weight loss while still supporting a heart-healthy diet.  * If you have a medical condition, such as diabetes, high blood pressure, or high cholesterol, your healthcare provider may recommend a specific calorie intake based on your individual needs.  Heart-healthy diet principles:  While calorie intake is important, it's equally essential to focus on the quality of your diet. A heart-healthy diet should emphasize:  1. Fruits and vegetables: Aim for at least 5 servings a day.  2. Whole grains: Choose whole, unprocessed grains like brown rice, quinoa, whole wheat bread, and whole grain pasta.  3. Lean protein sources: Include low-fat dairy products, poultry, fish, beans, lentils, and nuts.  4. Healthy fats: Nuts, seeds, avocados, and olive oil are good sources of healthy fats.  5. Limited added sugars: Aim to limit added sugars to no more than 25 grams (6 teaspoons) per day.  6. Limited saturated and trans fats: Choose lean protein sources, and limit intake of processed meats and full-fat dairy products.  7. Low sodium intake: Aim for no more than 2,300 milligrams of sodium per day.  Remember to consult with a healthcare professional or a registered dietitian to determine the best calorie intake and diet plan for your individual needs and health goals. |
| **Llama 3 Answer 3** | The ideal daily calorie intake for a heart-healthy diet varies depending on several factors, including your age, sex, weight, height, and physical activity level. Here are some general guidelines from the American Heart Association (AHA) and the Dietary Guidelines for Americans 2020:  Recommended Daily Calorie Intake:  * Sedentary women (little or no exercise): 1,600-2,000 calories/day  * Sedentary men (little or no exercise): 1,900-2,400 calories/day  * Active women (light exercise/sports 1-3 days/week): 1,800-2,200 calories/day  * Active men (light exercise/sports 1-3 days/week): 2,000-2,400 calories/day  * Very active women (heavy exercise/sports 4-6 days/week): 2,000-2,400 calories/day  * Very active men (heavy exercise/sports 4-6 days/week): 2,200-2,800 calories/day  * Extremely active women (very heavy exercise/sports & physical job or 2x training): 2,400-2,800 calories/day  * Extremely active men (very heavy exercise/sports & physical job or 2x training): 2,800-3,000 calories/day  Additional Considerations:  * If you're trying to lose weight, you may need to reduce your daily calorie intake by 500-1000 calories to promote weight loss while still meeting your nutritional needs.  * If you have a medical condition, such as diabetes, high blood pressure, or high cholesterol, your healthcare provider may recommend a specific calorie intake based on your individual needs.  Heart-Healthy Diet Principles:  While calorie intake is important, it's equally crucial to focus on the quality of your diet. A heart-healthy diet should emphasize:  1. Fruits and vegetables: Aim for at least 5 servings a day.  2. Whole grains: Choose whole, unprocessed grains like brown rice, quinoa, whole wheat bread, and whole grain pasta.  3. Lean protein sources: Include poultry, fish, beans, lentils, and low-fat dairy products.  4. Healthy fats: Nuts, seeds, avocados, and olive oil are good sources of healthy fats.  5. Limited added sugars: Aim to limit added sugars to no more than 25 grams (6 teaspoons) per day for women and 36 grams (9 teaspoons) per day for men.  6. Limited saturated and trans fats: Choose lean protein sources and healthy fats, and limit processed meats, full-fat dairy products, and fried foods.  Remember to consult with a healthcare professional or a registered dietitian to determine the best calorie intake and dietary plan for your individual needs and health goals. |
| **LLaMA3+RAG model Answer 1** | To support a heart-healthy diet, the ideal daily calorie intake varies depending on individual factors such as age, sex, weight, height, and activity level. The American Heart Association (AHA) does not provide a specific daily calorie recommendation in the provided context. However, it does offer guidance on setting calorie targets for weight loss.  According to the AHA, to lose 1 pound per week, most people need to subtract about 500 calories per day from their diet. This implies that the daily calorie intake for weight loss would be the individual's maintenance calories minus 500.  To determine your daily calorie needs, you can use the following estimates based on the Dietary Guidelines for Americans 2020:  Sedentary women: 1,600-2,000 calories/day  Sedentary men: 1,900-2,400 calories/day  Active women: 1,800-2,200 calories/day  Active men: 2,000-2,400 calories/day  Keep in mind that these are general estimates, and your individual calorie needs may vary. It's essential to consult with a healthcare professional or registered dietitian to determine your specific calorie needs for a heart-healthy diet.  In addition to calorie intake, it's crucial to focus on nutrient-dense eating, including plenty of fruits, vegetables, whole grains, lean proteins, and healthy fats. The AHA recommends spreading calorie intake across the day, avoiding skipping meals, and making smart choices when dining out or cooking at home.  Sources:  American Heart Association. (2024). Is 3 Meals a Day the Only Way? Retrieved from <https://www.heart.org/en/healthy-living/healthy-eating/eat-smart/nutrition-basics/is-3-meals-a-day-the-only-way>  Dietary Guidelines for Americans 2020. (2020). U.S. Department of Agriculture and U.S. Department of Health and Human Services. Retrieved from <https://health.gov/dietaryguidelines/2020/resources/Dietary-Guidelines-for-Americans-2020.pdf>  Academy of Nutrition and Dietetics. (2024). How Many Calories Do I Need? Retrieved from <https://www.andjrnl.org/article/S2212-2672(20)30223-5/> |
| **LLaMA3+RAG model Answer 2** | To support a heart-healthy diet, the ideal daily calorie intake varies depending on several factors, including age, sex, weight, height, and physical activity level. According to the American Heart Association (AHA), the recommended daily calorie intake for adults is:  For women:  + Sedentary (little or no exercise): 1,600-2,000 calories/day  + Lightly active (light exercise/sports 1-3 days/week): 1,800-2,200 calories/day  + Moderately active (moderate exercise/sports 3-5 days/week): 2,000-2,400 calories/day  + Very active (hard exercise/sports 6-7 days a week): 2,200-2,800 calories/day  + Extremely active (very hard exercise/sports & physical job or 2x training): 2,400-3,000 calories/day  For men:  + Sedentary (little or no exercise): 1,900-2,400 calories/day  + Lightly active (light exercise/sports 1-3 days/week): 2,000-2,400 calories/day  + Moderately active (moderate exercise/sports 3-5 days/week): 2,200-2,800 calories/day  + Very active (hard exercise/sports 6-7 days a week): 2,400-3,000 calories/day  + Extremely active (very hard exercise/sports & physical job or 2x training): 2,800-3,500 calories/day  It's essential to note that these are general guidelines, and individual calorie needs may vary. For example, if you're trying to lose weight, you may need to subtract 500 calories per day from your diet, as mentioned in the provided context.  To maintain a healthy weight, it's crucial to focus on the total calorie intake rather than the frequency of meals. Eating more frequent, smaller meals throughout the day can help balance hunger and calories, but it's still important to monitor overall calorie intake to avoid weight gain.  In addition to calorie intake, a heart-healthy diet should emphasize whole, unprocessed foods, such as fruits, vegetables, whole grains, lean proteins, and healthy fats. It's also important to limit sugary drinks, saturated and trans fats, and sodium.  Sources:  American Heart Association. (2022). Is 3 Meals a Day the Only Way? Retrieved from <https://www.heart.org/en/healthy-living/healthy-eating/eat-smart/nutrition-basics/is-3-meals-a-day-the-only-way>  American Heart Association. (2022). Healthy Eating Plate. Retrieved from <https:// www.heart.org/en/healthy-living/healthy-eating/eat-smart/nutrition-basics/healthy-eating-plate>  National Institutes of Health. (2022). Dietary Guidelines for Americans 2020. Retrieved from <https://health.gov/our-work/food-nutrition/2020-dietary-guidelines> |
| **LLaMA3+RAG model Answer 3** | To support a heart-healthy diet, the ideal daily calorie intake varies depending on factors such as age, sex, weight, height, and physical activity level. According to the American Heart Association, there is no one-size-fits-all answer to the question of how many calories you should eat per day. However, here are some general guidelines:  For women, the recommended daily calorie intake is:  + 1,600-2,000 calories per day for sedentary women (little or no exercise)  + 1,800-2,200 calories per day for lightly active women (light exercise/sports 1-3 days/week)  + 2,000-2,400 calories per day for moderately active women (moderate exercise/sports 3-5 days/week)  + 2,200-2,800 calories per day for very active women (hard exercise/sports 6-7 days a week)  For men, the recommended daily calorie intake is:  + 2,000-2,400 calories per day for sedentary men (little or no exercise)  + 2,200-2,800 calories per day for lightly active men (light exercise/sports 1-3 days/week)  + 2,400-3,000 calories per day for moderately active men (moderate exercise/sports 3-5 days/week)  + 2,800-3,500 calories per day for very active men (hard exercise/sports 6-7 days a week)  It's also important to note that if you need to lose weight, you should aim to subtract about 500 calories per day from your diet to lose 1 pound per week. This can be achieved through a combination of reducing your daily calorie intake and increasing your physical activity level.  In addition to the total calorie intake, it's also important to focus on the quality of your diet, including eating a variety of fruits, vegetables, whole grains, lean protein sources, and healthy fats. It's also important to limit your intake of sugary drinks, saturated and trans fats, and sodium.  Sources:  American Heart Association. (2024). Is 3 Meals a Day the Only Way? Retrieved from <https://www.heart.org/en/healthy-living/healthy-eating/eat-smart/nutrition-basics/is-3-meals-a-day-the-only-way>  American Heart Association. (2024). Healthy Eating Plate. Retrieved from <https://www.heart.org/en/healthy-living/healthy-eating/eat-smart/nutrition-basics/healthy-eating-plate>  Academy of Nutrition and Dietetics. (2024). How Many Calories Do I Need? Retrieved from <https://www.andjrnl.org/article/S2212-2672(19)30335-6/>  National Institutes of Health. (2024). Dietary Guidelines for Americans 2020. Retrieved from <https://health.gov/our-work/food-nutrition/2020-dietary-guidelines> |
| **Question 23** | **Which diet is best to prevent heart disease?** |
| **Perplexity Answer 1** | To prevent heart disease, the best diet emphasizes:  - Plenty of fruits and vegetables, aiming for a variety of colors[1][2][4][5]  - Whole grains instead of refined grains[1][2][4]  - Lean proteins like fish, poultry, legumes, nuts and seeds[1][2][3][4][5]  - Low-fat or fat-free dairy products[1][4]  - Healthy unsaturated fats from plant oils, avocados, nuts and seeds[2][4][5]  Specific recommendations include:  - Eating oily fish like salmon at least once per week for heart-healthy omega-3 fatty acids[2][4]  - Snacking on a handful of nuts, especially walnuts and almonds, most days of the week[3][4]  - Choosing lean, unprocessed meats and limiting red meat to 1-3 times per week[4]  - Eating legumes like beans, lentils and soybeans regularly[3][4]  - Limiting sodium, added sugars, saturated fats, and processed foods[1][2][4]  The DASH diet and Mediterranean diet are two well-studied eating patterns that incorporate these heart-healthy foods and have been shown to reduce heart disease risk factors like high blood pressure and cholesterol[2][4]. Maintaining a healthy weight by balancing calories from food with physical activity is also important for heart health[2].  Citations:  [1] https://www.mayoclinic.org/diseases-conditions/heart-disease/in-depth/heart-healthy-diet/art-20047702  [2] https://www.health.harvard.edu/heart-health/heart-healthy-foods-what-to-eat-and-what-to-avoid  [3] https://health.ucdavis.edu/blog/good-food/im-a-cardiovascular-dietitian-here-are-7-things-i-eat-and-avoid-for-heart-health/2023/02  [4] https://www.betterhealth.vic.gov.au/health/conditionsandtreatments/heart-disease-and-food  [5] https://www.healthline.com/nutrition/heart-healthy-foods |
| **Perplexity Answer 2** | To prevent heart disease, the best diet emphasizes:  - Plenty of fruits and vegetables, aiming for a variety of colors[1][2][4][5]  - Whole grains instead of refined grains[1][2][4]  - Lean proteins like fish, poultry, legumes, nuts and seeds[1][2][3][4]  - Low-fat or fat-free dairy products[1][4]  - Healthy unsaturated fats from plant oils, avocados, nuts and seeds[2][4][5]  Specific recommendations include:  - Eating oily fish like salmon at least once per week for heart-healthy omega-3 fatty acids[2][4]  - Snacking on a handful of nuts, especially walnuts and almonds, most days of the week[3][4]  - Choosing minimally processed foods and limiting fried, fast, and processed foods[1][4]  - Reducing sodium intake by avoiding packaged/processed foods and limiting salt[1][4]  The DASH diet, which emphasizes fruits, vegetables, whole grains, lean proteins and low-fat dairy, has been shown to significantly boost cardiovascular health[2]. Portion control is also important, as overeating can lead to obesity, a major risk factor for heart disease[1].  By following these dietary guidelines and making heart-healthy food choices, you can greatly reduce your risk of developing heart disease. Combining a nutritious diet with regular exercise, maintaining a healthy weight, and avoiding smoking provides the best protection for your heart.  Citations:  [1] https://www.mayoclinic.org/diseases-conditions/heart-disease/in-depth/heart-healthy-diet/art-20047702  [2] https://www.health.harvard.edu/heart-health/heart-healthy-foods-what-to-eat-and-what-to-avoid  [3] https://health.ucdavis.edu/blog/good-food/im-a-cardiovascular-dietitian-here-are-7-things-i-eat-and-avoid-for-heart-health/2023/02  [4] https://www.betterhealth.vic.gov.au/health/conditionsandtreatments/heart-disease-and-food  [5] https://www.healthline.com/nutrition/heart-healthy-foods |
| **Perplexity Answer 3** | To prevent heart disease, the best diet emphasizes:  - Plenty of fruits and vegetables, aiming for a variety of colors[1][2][4][5]  - Whole grains instead of refined grains[1][2][4]  - Lean proteins like fish, poultry, legumes, nuts and seeds[1][2][3][4]  - Low-fat or fat-free dairy products[1][4]  - Healthy unsaturated fats from plant oils, avocados, nuts and seeds[2][4][5]  Specific recommendations include:  - Eating oily fish like salmon at least once per week for heart-healthy omega-3 fatty acids[2][4]  - Snacking on a handful of nuts, especially walnuts and almonds, most days of the week[3][4]  - Choosing minimally processed foods and limiting fried, fast, and processed foods[1][4]  - Reducing sodium intake by avoiding packaged foods, limiting fast foods, and using herbs and spices instead of salt[1][4]  The DASH diet, which is low in sodium and rich in fruits, vegetables, whole grains and lean proteins, has been shown to significantly reduce blood pressure and heart disease risk[2].  Controlling portion sizes, maintaining a healthy weight, and limiting alcohol are also important for heart health[1][4]. Overall, a diet high in plant foods, lean proteins, and healthy fats while minimizing processed foods and sodium is the best approach to prevent heart disease.  Citations:  [1] https://www.mayoclinic.org/diseases-conditions/heart-disease/in-depth/heart-healthy-diet/art-20047702  [2] https://www.health.harvard.edu/heart-health/heart-healthy-foods-what-to-eat-and-what-to-avoid  [3] https://health.ucdavis.edu/blog/good-food/im-a-cardiovascular-dietitian-here-are-7-things-i-eat-and-avoid-for-heart-health/2023/02  [4] https://www.betterhealth.vic.gov.au/health/conditionsandtreatments/heart-disease-and-food  [5] https://www.healthline.com/nutrition/heart-healthy-foods |
| **GPT4o Answer 1** | Preventing heart disease through diet involves focusing on foods and eating patterns that reduce risk factors such as high cholesterol, high blood pressure, inflammation, and excess weight. Here are some of the best dietary approaches to prevent heart disease:  1. Mediterranean Diet  The Mediterranean diet emphasizes:  - Fruits and vegetables: Aim for a variety of colorful fruits and vegetables.  - Whole grains: Include whole wheat, oats, barley, and other whole grains.  - Healthy fats: Use olive oil as the primary fat, and include nuts and seeds.  - Fish and seafood: Consume fish, especially fatty fish like salmon, mackerel, and sardines, at least twice a week.  - Lean proteins: Include poultry, eggs, and legumes, while limiting red meat.  - Herbs and spices: Use herbs and spices instead of salt to flavor foods.  2. DASH Diet (Dietary Approaches to Stop Hypertension)  The DASH diet focuses on:  - Fruits and vegetables: Aim for 4-5 servings of each per day.  - Whole grains: Include 6-8 servings per day.  - Low-fat dairy: Consume 2-3 servings of low-fat or fat-free dairy products.  - Lean proteins: Include fish, poultry, and legumes, while limiting red meat and full-fat dairy.  - Nuts, seeds, and legumes: Include 4-5 servings per week.  - Limited sodium: Aim to reduce sodium intake to less than 2,300 mg per day, and ideally to 1,500 mg per day.  3. Plant-Based Diets  Plant-based diets emphasize:  - Fruits and vegetables: Prioritize a wide variety of fruits and vegetables.  - Whole grains: Focus on whole grains such as brown rice, quinoa, and whole wheat.  - Legumes: Include beans, lentils, and peas as primary protein sources.  - Nuts and seeds: Incorporate nuts and seeds for healthy fats and proteins.  - Minimal animal products: Limit or eliminate meat, dairy, and eggs.  4. Low-Carbohydrate Diets (When focusing on healthy fats)  Low-carb diets can be heart-healthy if they emphasize:  - Non-starchy vegetables: Prioritize leafy greens, broccoli, cauliflower, and other non-starchy vegetables.  - Healthy fats: Include sources such as avocados, olive oil, nuts, and fatty fish.  - Lean proteins: Focus on fish, poultry, and plant-based proteins.  - Limited refined carbs: Avoid sugars, refined grains, and highly processed foods.  Key Nutrients for Heart Health:  - Fiber: Found in fruits, vegetables, whole grains, and legumes.  - Omega-3 fatty acids: Found in fatty fish, flaxseeds, and walnuts.  - Antioxidants: Found in fruits, vegetables, nuts, and seeds.  - Potassium: Found in fruits, vegetables, dairy, and fish.  - Magnesium: Found in whole grains, nuts, and leafy green vegetables.  General Tips:  - Limit added sugars: Reduce consumption of sugary beverages, snacks, and desserts.  - Limit saturated and trans fats: Avoid fried foods, fast foods, and processed snacks.  - Watch portion sizes: Be mindful of portion sizes to maintain a healthy weight.  - Stay hydrated: Drink plenty of water and limit sugary and high-calorie drinks.  Combining these dietary practices with regular physical activity, maintaining a healthy weight, not smoking, and managing stress can significantly reduce the risk of heart disease. It's also important to consult with a healthcare provider or a registered dietitian to tailor dietary choices to individual health needs and conditions. |
| **GPT4o Answer 2** | Preventing heart disease through diet involves consuming foods that support cardiovascular health. Here are some of the most recommended diets for heart disease prevention:  Mediterranean Diet  The Mediterranean diet is highly praised for its heart-healthy benefits. Key components include:  - Fruits and Vegetables: Rich in vitamins, minerals, and fiber.  - Whole Grains: Such as brown rice, whole wheat, and oats.  - Healthy Fats: Primarily from olive oil, nuts, and seeds.  - Fish: Especially fatty fish like salmon and mackerel, which are high in omega-3 fatty acids.  - Legumes: Beans, lentils, and chickpeas.  - Moderate Dairy: Mainly low-fat or fat-free options.  - Limited Red Meat: Mostly lean cuts, with more emphasis on poultry and fish.  - Red Wine: In moderation.  DASH Diet (Dietary Approaches to Stop Hypertension)  The DASH diet is designed to combat high blood pressure, a major risk factor for heart disease. Its principles include:  - Fruits and Vegetables: Emphasizing variety and quantity.  - Whole Grains: Providing essential nutrients and fiber.  - Low-fat Dairy: Including milk, yogurt, and cheese.  - Lean Proteins: Such as poultry, fish, and legumes.  - Nuts and Seeds: For healthy fats and protein.  - Limited Sodium: Reducing salt intake to lower blood pressure.  - Reduced Red Meat and Sweets: Focusing on healthier protein sources and limiting added sugars.  Plant-Based Diet  A plant-based diet can significantly benefit heart health by emphasizing:  - Fruits and Vegetables: Abundant and varied.  - Whole Grains: Such as quinoa, barley, and brown rice.  - Legumes: Beans, lentils, and peas for protein and fiber.  - Nuts and Seeds: For healthy fats and protein.  - Minimal Processed Foods: Avoiding processed and refined foods.  - Limited Animal Products: If included, they should be lean and in small amounts.  Key Dietary Recommendations  Regardless of the specific diet, certain common principles help in preventing heart disease:  - Increase Fiber Intake: Aim for at least 25-30 grams of fiber per day.  - Reduce Saturated and Trans Fats: Choose healthier fats like those in olive oil, nuts, and avocados.  - Limit Added Sugars: Minimize consumption of sugary drinks and snacks.  - Control Portion Sizes: Maintain a healthy weight to reduce heart disease risk.  - Stay Hydrated: Drink plenty of water and limit sugary beverages.  Foods to Include  - Leafy Greens: Spinach, kale, and Swiss chard.  - Berries: Strawberries, blueberries, and raspberries.  - Nuts and Seeds: Almonds, walnuts, chia seeds, and flaxseeds.  - Whole Grains: Oats, quinoa, and whole-wheat products.  - Healthy Fats: Olive oil, avocado, and fatty fish.  Foods to Avoid  - Processed Meats: Sausages, hot dogs, and deli meats.  - Sugary Beverages: Sodas, fruit drinks, and energy drinks.  - Refined Carbohydrates: White bread, pastries, and sugary cereals.  - Trans Fats: Found in many processed and fried foods.  - Excessive Salt: Found in canned soups, snacks, and fast food.  Incorporating these dietary guidelines can significantly reduce the risk of heart disease. Always consult with a healthcare provider or a dietitian to tailor dietary choices to individual health needs and conditions. |
| **GPT4o Answer 3** | Preventing heart disease through diet involves choosing foods that promote heart health and avoiding those that can contribute to heart disease. Here are some dietary patterns and specific dietary choices that are widely recommended by health experts for heart disease prevention:  1. Mediterranean Diet  - Key Features:  - High consumption of fruits, vegetables, whole grains, nuts, and seeds.  - Healthy fats, primarily from olive oil.  - Moderate consumption of fish and poultry.  - Limited red meat and sweets.  - Moderate wine consumption, typically with meals.  2. DASH Diet (Dietary Approaches to Stop Hypertension)  - Key Features:  - Emphasizes vegetables, fruits, whole grains, and lean proteins.  - Low in saturated fat, cholesterol, and total fat.  - Focus on low-fat dairy products, fish, poultry, beans, and nuts.  - Reduced intake of sodium, sweets, and red meats.  3. Plant-Based Diets  - Key Features:  - High in fruits, vegetables, whole grains, legumes, nuts, and seeds.  - Avoids or minimizes animal products, including red meat, poultry, fish, and dairy.  - Low in saturated fats and cholesterol.  4. Whole Food, Plant-Based Diet  - Key Features:  - Similar to a plant-based diet but focuses more on minimally processed foods.  - Avoids processed foods, oils, and refined sugars.  - High in fiber, antioxidants, and beneficial plant compounds.  5. Flexitarian Diet  - Key Features:  - Primarily vegetarian with occasional inclusion of meat and animal products.  - Emphasizes plant-based foods most of the time.  - Focus on whole grains, legumes, and a variety of fruits and vegetables.  Specific Dietary Recommendations:  1. Increase Fiber Intake:  - Aim for whole grains, fruits, vegetables, and legumes.  - High fiber intake is associated with lower cholesterol levels and reduced heart disease risk.  2. Healthy Fats:  - Choose unsaturated fats like those found in olive oil, avocados, and nuts.  - Omega-3 fatty acids from fatty fish (e.g., salmon, mackerel) are beneficial.  3. Reduce Saturated and Trans Fats:  - Limit consumption of red meat, full-fat dairy products, and processed foods.  - Avoid trans fats found in many fried and commercially baked products.  4. Increase Antioxidant-Rich Foods:  - Fruits and vegetables high in antioxidants (e.g., berries, leafy greens) can protect against heart disease.  5. Limit Sodium Intake:  - High sodium intake is linked to hypertension, a risk factor for heart disease.  - Use herbs and spices instead of salt to flavor foods.  6. Moderate Alcohol Consumption:  - If consumed, limit alcohol to moderate levels (one drink per day for women and up to two for men).  Conclusion:  The best diet to prevent heart disease typically emphasizes plant-based foods, healthy fats, and whole grains while minimizing processed foods, red meat, and added sugars. Both the Mediterranean and DASH diets are highly regarded and supported by extensive research for their heart-health benefits. However, individual dietary preferences and health conditions should guide the choice of diet. Consulting with a healthcare provider or a registered dietitian can help tailor dietary choices to individual needs. |
| **Llama 3 Answer 1** | The best diet to prevent heart disease is a comprehensive eating plan that focuses on whole, nutrient-dense foods and minimizes processed and unhealthy ones. Here are some general principles and specific diets that can help:  General Principles:  1. Emphasize whole, unprocessed foods: Focus on fruits, vegetables, whole grains, lean proteins, and healthy fats.  2. Limit saturated and trans fats: Opt for healthier fats like avocado, nuts, and olive oil.  3. Choose lean protein sources: Include fatty fish, poultry, beans, lentils, and low-fat dairy products.  4. Consume omega-3 fatty acids: Find them in fatty fish, walnuts, flaxseeds, and chia seeds.  5. Stay hydrated: Drink plenty of water and limit sugary drinks.  6. Limit sodium and added sugars: Aim for no more than 2,300 milligrams of sodium and 25 grams of added sugars per day.  7. Maintain a healthy weight: Aim for a body mass index (BMI) between 18.5 and 24.9.  Specific Diets:  1. Mediterranean Diet: Emphasizes whole grains, fruits, vegetables, lean proteins, and healthy fats, such as the Mediterranean diet, which has been shown to reduce heart disease risk by up to 30%.  2. DASH Diet (Dietary Approaches to Stop Hypertension): Focuses on whole grains, fruits, vegetables, lean proteins, and low-fat dairy products, with an emphasis on reducing sodium intake.  3. Plant-Based Diet: A well-planned plant-based diet that includes a variety of fruits, vegetables, whole grains, and legumes can help lower heart disease risk.  4. American Heart Association Diet: Recommends a balanced diet that includes a variety of fruits, vegetables, whole grains, lean proteins, and healthy fats, with an emphasis on reducing saturated and trans fats.  Foods to Include:  1. Leafy greens: Spinach, kale, collard greens, and other leafy greens are rich in vitamins, minerals, and antioxidants.  2. Berries: Blueberries, strawberries, raspberries, and other berries are high in antioxidants and fiber.  3. Nuts and seeds: Almonds, walnuts, chia seeds, and flaxseeds are rich in healthy fats and fiber.  4. Fatty fish: Fatty fish like salmon, tuna, and mackerel are high in omega-3 fatty acids.  5. Avocados: Rich in healthy fats, fiber, and various vitamins and minerals.  6. Olive oil: A healthy source of monounsaturated fats.  7. Whole grains: Brown rice, quinoa, whole wheat bread, and whole grain pasta provide fiber, vitamins, and minerals.  Foods to Limit or Avoid:  1. Processed meats: Hot dogs, sausages, bacon, and other processed meats are high in sodium and preservatives.  2. Refined carbohydrates: White bread, sugary drinks, and sweets can lead to insulin resistance and inflammation.  3. Saturated and trans fats: Limit foods high in saturated fats, such as butter, lard, and partially hydrogenated oils.  4. High-sodium foods: Limit foods with high sodium content, such as canned soups, processed snacks, and frozen meals.  Remember, a heart-healthy diet is just one aspect of maintaining overall cardiovascular health. Regular physical activity, stress management, and not smoking are also essential for reducing heart disease risk. Consult with a healthcare professional or registered dietitian to create a personalized plan that suits your needs. |
| **Llama 3 Answer 2** | Preventing heart disease requires a comprehensive approach that includes a healthy diet, regular exercise, stress management, and not smoking. When it comes to diet, there is no single "best" diet, but rather a pattern of eating that emphasizes whole, nutrient-dense foods and limits unhealthy ones. Here are some evidence-based dietary recommendations to help prevent heart disease:  1. Mediterranean Diet: This diet is rich in fruits, vegetables, whole grains, lean protein, and healthy fats, such as those found in olive oil. It has been consistently shown to reduce the risk of heart disease.  2. DASH Diet (Dietary Approaches to Stop Hypertension): This diet focuses on whole grains, fruits, vegetables, lean protein, and low-fat dairy. It's designed to help manage blood pressure and cholesterol levels.  3. Plant-Based Diet: A diet rich in fruits, vegetables, whole grains, and legumes can help reduce the risk of heart disease by lowering blood pressure, cholesterol, and inflammation.  4. Portfolio Diet: This diet combines elements of the Mediterranean and DASH diets, with an emphasis on soluble fiber, plant sterols, and stanols to help lower cholesterol.  Key dietary components to focus on:  1. Fruits and Vegetables: Aim for at least 5 servings a day, with a variety of colors to ensure a range of antioxidants and fiber.  2. Whole Grains: Choose whole, unprocessed grains like brown rice, quinoa, whole wheat bread, and oats.  3. Lean Protein: Include sources like poultry, fish, beans, lentils, and low-fat dairy products.  4. Healthy Fats: Nuts, seeds, avocados, and olive oil are all good sources of healthy fats.  5. Low-Fat Dairy: Choose low-fat or fat-free dairy products to reduce saturated fat intake.  6. Omega-3 Fatty Acids: Fatty fish like salmon, tuna, and mackerel are rich in omega-3s, which can help lower triglycerides and blood pressure.  7. Limit or Avoid:  * Saturated and trans fats (found in processed meats, full-fat dairy, and fried foods)  * Refined carbohydrates (white bread, sugary drinks, and sweets)  * Sodium (limit to less than 2,300 milligrams per day)  * Added sugars (limit to less than 10% of daily calories)  Remember, a healthy diet is just one aspect of preventing heart disease. Regular physical activity, stress management, and not smoking are also crucial for overall cardiovascular health. Consult with a healthcare professional or a registered dietitian to create a personalized plan that suits your needs and lifestyle. |
| **Llama 3 Answer 3** | While there's no single "best" diet to prevent heart disease, a well-planned eating pattern that emphasizes whole, nutrient-dense foods can help reduce the risk of heart disease. Here are some dietary approaches that have been shown to be beneficial:  1. Mediterranean Diet: This diet focuses on whole grains, fruits, vegetables, lean protein, and healthy fats, such as those found in olive oil. It's rich in fiber, vitamins, and minerals, and has been shown to lower cardiovascular disease risk by 30% [1].  2. DASH Diet (Dietary Approaches to Stop Hypertension): This diet is designed to lower blood pressure and cholesterol levels by emphasizing whole grains, fruits, vegetables, lean protein, and low-fat dairy. It's also rich in potassium, calcium, and fiber [2].  3. Plant-Based Diet: A diet rich in fruits, vegetables, whole grains, and legumes, with minimal or no animal products, can help lower blood pressure, cholesterol, and body mass index (BMI). Plant-based diets tend to be high in fiber, vitamins, and minerals [3].  4. Portfolio Diet: This diet combines elements of the Mediterranean and DASH diets, with an emphasis on soluble fiber, plant sterols, and stanols. It's been shown to lower LDL (bad) cholesterol by 13% [4].  Common characteristics of these diets include:  * High consumption of:  + Fruits and vegetables (at least 5 servings a day)  + Whole grains (such as brown rice, quinoa, and whole-wheat bread)  + Lean protein sources (like poultry, fish, and legumes)  + Healthy fats (like olive oil, avocado, and nuts)  * Low consumption of:  + Saturated and trans fats (found in red meat, full-fat dairy, and processed snacks)  + Refined carbohydrates (like white bread, sugary drinks, and sweets)  + Sodium (limit to less than 2,300 milligrams per day)  Additionally, consider the following tips to prevent heart disease:  * Maintain a healthy weight: Excess weight, particularly around the waist, increases the risk of heart disease.  * Limit dietary cholesterol: Consume less than 300 milligrams of dietary cholesterol per day.  * Choose healthy fats: Opt for unsaturated fats, like those found in nuts, seeds, and avocados, instead of saturated and trans fats.  * Stay hydrated: Drink plenty of water and limit sugary drinks.  * Limit processed and packaged foods: These foods tend to be high in sodium, added sugars, and unhealthy fats.  Remember, a healthy diet is just one aspect of preventing heart disease. Regular physical activity, stress management, and not smoking are also crucial for maintaining heart health.  Consult with a healthcare professional or a registered dietitian to determine the best diet for your individual needs and health goals.  References:  [1] Estruch, R., et al. (2013). Primary prevention of cardiovascular disease with a Mediterranean diet. New England Journal of Medicine, 368(14), 1279-1290.  [2] Sacks, F. M., et al. (2001). Effects of blood pressure reduction on cardiovascular disease: Results of the DASH-sodium trial. New England Journal of Medicine, 344(1), 3-10.  [3] Sabaté, J., et al. (2014). Vegetarian diets and cardiovascular risk factors: A systematic review and meta-analysis. Annals of Nutrition and Metabolism, 64(2-3), 32-40.  [4] Jenkins, D. J., et al. (2010). The portfolio diet: A practical approach to reducing cardiovascular disease risk. Journal of Clinical Lipidology, 4(3), 192-199. |
| **LLaMA3+RAG model Answer 1** | According to the American Heart Association, the best diet to prevent heart disease is a heart-healthy dietary pattern that includes:  1. Healthy sources of proteins, mostly from plant sources;  2. Regularly eating fish and seafood (at least 2 servings per week, with a serving size of 3 ounces cooked or about ¾ cup of flaked fish);  3. Substituting nonfat and low-fat dairy products in place of full-fat versions;  4. Choosing lean and unprocessed meat or poultry, if consumed.  This dietary pattern is recommended because it has been consistently associated with a lower risk of cardiovascular disease. Fish, in particular, is a good source of protein and omega-3 fatty acids, which have been shown to reduce the risk of heart disease and stroke.  The American Heart Association recommends eating a variety of fish to minimize the potential risks associated with environmental pollutants. It is also important to check the FDA website for the latest advisories on fish consumption, especially for women who are pregnant, planning to become pregnant, or nursing, as well as parents or caregivers of young children.  In addition to incorporating fish and seafood into the diet, it is also important to choose healthier cooking methods, such as grilling, steaming, barbequing, poaching, baking, or pan-frying, and to use healthier oils like olive oil, canola oil, sunflower oil, or peanut oil.  Overall, a heart-healthy dietary pattern that includes regular consumption of fish and seafood, along with other healthy food choices, can help to minimize the risk of heart disease and stroke.  Sources:  American Heart Association. (2024). Fish and Omega-3 Fatty Acids. Retrieved from <https://www.heart.org/en/healthy-living/healthy-eating/eat-smart/fats/fish-and-omega-3-fatty-acids>  Heart Foundation. (2024). Not Eating Enough Fish? Retrieved from <https://www.heartfoundation.org.au/blog/not-eating-enough-fish>  U.S. Food and Drug Administration. (2024). Fish: What Pregnant Women and Parents Should Know. Retrieved from <https://www.fda.gov/food/consumers/fish-and-pregnancy> |
| **LLaMA3+RAG model Answer 2** | The best diet to prevent heart disease is a dietary pattern that emphasizes healthy sources of proteins, mostly from plant sources, and regularly includes fish and seafood. This diet should also substitute nonfat and low-fat dairy products in place of full-fat versions, and for those who eat meat or poultry, choosing lean and unprocessed options.  The American Heart Association recommends eating at least 2 servings of fish (particularly fatty fish) per week, with a serving size of 3 ounces cooked or about ¾ cup of flaked fish. Fatty fish like anchovies, herring, mackerel, black cod, salmon, sardines, bluefin tuna, whitefish, striped bass, and cobia are high in omega-3 fatty acids, which can reduce the risk of heart disease and stroke.  In addition to fish, a heart-healthy diet should also include a variety of fruits, vegetables, whole grains, and healthy fats, such as those found in nuts, seeds, and avocados. It's also important to limit intake of saturated and trans fats, sodium, and added sugars.  Some tips to incorporate more fish and seafood into your diet include:  Adding canned tuna or salmon to salads, sandwiches, or eating as a snack  Using seafood marinara mixes in stir-fries and pasta  Baking or steaming fish parcels for a quick and easy dinner  Trying different seasonings and herbs, such as garlic, ginger, and lemon, to add flavor to fish dishes  Incorporating frozen seafood into meal planning to make it more convenient and affordable  Remember, a consistent, heart-healthy eating pattern is key to reducing the risk of heart disease and stroke.  Sources:  American Heart Association. (2024). Fish and Omega-3 Fatty Acids. Retrieved from <https://www.heart.org/en/healthy-living/healthy-eating/eat-smart/fats/fish-and-omega-3-fatty-acids>  American Heart Association. (2024). Eating Fish for Heart Health. Retrieved from <https://www.heartfoundation.org.au/blog/not-eating-enough-fish>  U.S. Food and Drug Administration. (2024). Fish: What Pregnant Women and Parents Should Know. Retrieved from <https://www.fda.gov/food/consumers/fish-what-pregnant-women-and-parents-should-know> |
| **LLaMA3+RAG model Answer 3** | According to the American Heart Association, the best diet to prevent heart disease is a dietary pattern that includes:  1. Healthy sources of proteins, mostly from plant sources  2. Regularly eating fish and seafood (at least 2 servings per week, with a serving size of 3 ounces cooked or about ¾ cup of flaked fish)  3. Substituting nonfat and low-fat dairy products in place of full-fat versions  4. Choosing lean and unprocessed meat or poultry for those who eat meat or poultry  This dietary pattern is recommended because it can help reduce the risk of heart disease and stroke. Fish and seafood are particularly beneficial due to their high levels of omega-3 fatty acids, which are good for heart health.  Incorporating fish and seafood into your diet can be easy and convenient. You can add canned tuna or salmon to salads, sandwiches, or eat them as a snack. You can also try seafood marinara mixes in stir-fries and pasta, or bake or steam fish parcels for a quick and tasty dinner. Remember to choose healthier cooking methods such as grilling, steaming, barbequing, poaching, baking, or pan-frying, and use healthier oils like olive oil, canola oil, sunflower oil, or peanut oil.  It's also important to note that while fish and seafood are beneficial for heart health, it's essential to choose a variety of fish to minimize any potentially adverse effects due to environmental pollutants. Pregnant women, babies, and young children should check the FDA website for the latest advisories on fish consumption.  Sources:  American Heart Association. (2024). Fish and Omega-3 Fatty Acids. Retrieved from <https://www.heart.org/en/healthy-living/healthy-eating/eat-smart/fats/fish-and-omega-3-fatty-acids>  Heart Foundation. (2024). Not Eating Enough Fish. Retrieved from <https://www.heartfoundation.org.au/blog/not-eating-enough-fish>  U.S. Food and Drug Administration. (2024). Fish: What Pregnant Women and Parents Should Know. Retrieved from <https://www.fda.gov/food/consumers/fish-what-pregnant-women-and-parents-should-know> |
| **Question 24** | **Can I drink alcohol on a heart healthy diet?** |
| **Perplexity Answer 1** | No, drinking alcohol is not recommended as part of a heart-healthy diet. While some studies have suggested a possible link between moderate alcohol consumption and a lower risk of heart disease, the evidence is not conclusive[1][3][4]. The American Heart Association does not recommend drinking alcohol to gain potential health benefits[4].  Excessive alcohol intake is clearly linked to several cardiovascular health risks, including high blood pressure, heart failure, stroke, and irregular heart rhythms[1][3][4]. Even moderate drinking can raise triglyceride levels and contribute to obesity, which are risk factors for heart disease[3][5].  If you do choose to drink alcohol, it's important to do so in moderation, defined as no more than one drink per day for women and one to two drinks per day for men[1][3][4][5]. However, the healthiest option is to avoid alcohol altogether and focus on proven ways to improve heart health, such as:  - Eating a diet rich in fruits, vegetables, whole grains, lean proteins and healthy fats  - Maintaining a healthy weight  - Getting regular physical activity  - Managing stress  - Quitting smoking  - Controlling conditions like high blood pressure and diabetes  In summary, while the occasional alcoholic drink in moderation is unlikely to be harmful for most people, it does not provide any heart health benefits and is not recommended as part of a heart-healthy lifestyle. The best approach is to avoid alcohol and prioritize other lifestyle factors that are proven to support cardiovascular health.  Citations:  [1] https://www.hopkinsmedicine.org/health/wellness-and-prevention/alcohol-and-heart-health-separating-fact-from-fiction  [2] https://www.heartfoundation.org.au/blog/alcohol-and-heart-health  [3] https://www.munsonhealthcare.org/blog/is-alcohol-heart-healthy  [4] https://www.heart.org/en/healthy-living/healthy-eating/eat-smart/nutrition-basics/alcohol-and-heart-health  [5] https://www.healthline.com/health/heart-disease/diet-sugar-caffeine-calcium-alcohol |
| **Perplexity Answer 2** | No, drinking alcohol is not recommended as part of a heart-healthy diet. While some studies have suggested that moderate alcohol consumption may provide some cardiovascular benefits, such as raising HDL (""good"") cholesterol levels, the evidence is not conclusive[1][3][4].  The American Heart Association does not recommend drinking alcohol to gain potential health benefits[4]. Excessive alcohol intake is linked to numerous health risks, including high blood pressure, heart failure, stroke, and cardiomyopathy[1][3][4]. Alcohol is also high in calories and can contribute to weight gain[1][4].  If you do choose to drink alcohol, it's important to do so in moderation. Moderate drinking is defined as no more than one drink per day for women and one to two drinks per day for men[1][3][4][5]. A drink is considered 12 ounces of regular beer, 4-5 ounces of wine, or 1.5 ounces of distilled spirits[3][4].  The healthiest approach is to focus on other lifestyle factors that are proven to benefit heart health, such as eating a diet rich in fruits, vegetables, whole grains, lean proteins and healthy fats, maintaining a healthy weight, getting regular physical activity, and avoiding smoking[4].  Citations:  [1] https://www.hopkinsmedicine.org/health/wellness-and-prevention/alcohol-and-heart-health-separating-fact-from-fiction  [2] https://www.heartfoundation.org.au/blog/alcohol-and-heart-health  [3] https://www.munsonhealthcare.org/blog/is-alcohol-heart-healthy  [4] https://www.heart.org/en/healthy-living/healthy-eating/eat-smart/nutrition-basics/alcohol-and-heart-health  [5] https://www.healthline.com/health/heart-disease/diet-sugar-caffeine-calcium-alcohol |
| **Perplexity Answer 3** | No, drinking alcohol is not recommended as part of a heart-healthy diet. While some studies have suggested that moderate alcohol consumption may provide some cardiovascular benefits, such as raising HDL (""good"") cholesterol levels, the evidence is not conclusive[1][3][4].  The American Heart Association does not recommend drinking alcohol to gain potential health benefits[4]. Excessive alcohol intake is linked to numerous health risks, including high blood pressure, heart failure, stroke, and cardiomyopathy[1][3][4]. Alcohol is also high in calories and can contribute to weight gain[1][4].  If you do choose to drink alcohol, it's important to do so in moderation. Moderate drinking is defined as no more than one drink per day for women and one to two drinks per day for men[1][3][4][5]. A drink is considered 12 ounces of regular beer, 4-5 ounces of wine, or 1.5 ounces of distilled spirits[3][4].  The healthiest approach is to focus on other lifestyle factors that are proven to benefit heart health, such as eating a diet rich in fruits, vegetables, whole grains, lean proteins and healthy fats, maintaining a healthy weight, getting regular physical activity, and avoiding smoking[4].  Citations:  [1] https://www.hopkinsmedicine.org/health/wellness-and-prevention/alcohol-and-heart-health-separating-fact-from-fiction  [2] https://www.heartfoundation.org.au/blog/alcohol-and-heart-health  [3] https://www.munsonhealthcare.org/blog/is-alcohol-heart-healthy  [4] https://www.heart.org/en/healthy-living/healthy-eating/eat-smart/nutrition-basics/alcohol-and-heart-health  [5] https://www.healthline.com/health/heart-disease/diet-sugar-caffeine-calcium-alcohol |
| **GPT4o Answer 1** | Drinking alcohol while following a heart-healthy diet can be a nuanced topic. Here are some key points to consider:  1. Moderation is Key: For those who choose to drink, moderation is essential. The American Heart Association (AHA) recommends no more than one drink per day for women and no more than two drinks per day for men. One drink is typically defined as:  - 12 ounces of beer  - 5 ounces of wine  - 1.5 ounces of distilled spirits  2. Type of Alcohol: Some studies suggest that certain types of alcohol, like red wine, might have heart benefits due to the presence of antioxidants such as resveratrol. However, these potential benefits do not outweigh the risks of excessive alcohol consumption.  3. Potential Benefits: Moderate alcohol consumption has been associated with a reduced risk of developing heart disease. It may help increase good cholesterol (HDL) and have a protective effect on the cardiovascular system.  4. Risks: Excessive alcohol consumption can lead to high blood pressure, heart failure, and even stroke. It can also contribute to weight gain, liver disease, and certain types of cancer. Moreover, alcohol can interact negatively with medications, including those for heart conditions.  5. Individual Factors: It's important to consider individual health conditions and risks. Those with certain health conditions, including certain types of heart disease, liver disease, or a history of alcoholism, should avoid alcohol entirely.  6. Dietary Balance: Ensuring a balanced diet that includes plenty of fruits, vegetables, whole grains, lean proteins, and healthy fats is crucial. Alcohol should not replace these nutritious foods and should be considered an occasional addition rather than a dietary staple.  If you have specific health conditions or are taking medications, it's best to consult with your healthcare provider to determine if and how much alcohol is safe for you to consume. |
| **GPT4o Answer 2** | When following a heart-healthy diet, moderate alcohol consumption can be included, but it’s important to understand the guidelines and potential impacts. Here are some key points to consider:  1. Moderation is Key:  - For women, moderate consumption means up to one drink per day.  - For men, it means up to two drinks per day.  2. Type of Alcohol:  - Some studies suggest that red wine, in particular, may have heart-healthy benefits due to its content of antioxidants like resveratrol. However, the evidence is not strong enough to recommend drinking wine specifically for heart health.  3. Potential Benefits:  - Moderate alcohol consumption has been linked to a reduced risk of developing heart disease. It may help increase good cholesterol (HDL) and reduce the risk of blood clots.  4. Risks of Alcohol:  - Excessive alcohol consumption can lead to high blood pressure, heart failure, and even stroke.  - It can also contribute to weight gain, which is a risk factor for heart disease.  - Some individuals should avoid alcohol entirely, including those with a history of alcoholism, liver disease, or certain heart conditions.  5. Lifestyle Considerations:  - It’s important to balance alcohol intake with other aspects of a heart-healthy lifestyle, including a diet rich in fruits, vegetables, whole grains, lean proteins, and healthy fats.  - Regular physical activity and avoiding smoking are also crucial for heart health.  Before making any changes to your diet or alcohol consumption, it’s always a good idea to discuss with your healthcare provider to tailor advice to your specific health needs and conditions. |
| **GPT4o Answer 3** | Yes, you can drink alcohol on a heart-healthy diet, but it's important to do so in moderation. Here are some guidelines:  1. Moderation is Key: For most people, moderate alcohol consumption is defined as up to one drink per day for women and up to two drinks per day for men.  2. Type of Alcohol: Red wine is often suggested for heart health because it contains antioxidants like resveratrol, which may help protect the lining of blood vessels in your heart. However, these benefits can also be obtained from other sources, and excessive alcohol consumption negates these benefits.  3. Overall Diet: Alcohol should be just one part of a balanced, heart-healthy diet that includes plenty of fruits, vegetables, whole grains, lean proteins, and healthy fats.  4. Health Conditions and Medications: If you have certain health conditions or are taking medications, alcohol may not be advisable. Always consult with your healthcare provider.  5. Risks of Excessive Drinking: Drinking too much alcohol can increase your risk of high blood pressure, heart failure, stroke, and other cardiovascular issues.  6. Calories: Alcohol can add extra calories to your diet, which may lead to weight gain, another risk factor for heart disease.  Moderate alcohol consumption may have some benefits for heart health, but it is not essential. Focus on a comprehensive approach to heart health that includes diet, exercise, and regular check-ups with your healthcare provider. |
| **Llama 3 Answer 1** | The eternal question! While a heart healthy diet is essential for maintaining cardiovascular well-being, moderate alcohol consumption can be a part of it, but with some caveats.  The good news:  Moderate alcohol consumption, defined as one drink per day for women and two drinks per day for men, may have some potential heart health benefits:  1. Cardiovascular disease risk reduction: Moderate alcohol consumption has been linked to a lower risk of cardiovascular disease, including heart attacks, strokes, and peripheral artery disease.  2. Increased HDL cholesterol: Alcohol can raise levels of high-density lipoprotein (HDL) cholesterol, the "good" cholesterol that helps remove excess cholesterol from the bloodstream.  3. Blood clot prevention: Moderate alcohol consumption may help prevent the formation of blood clots, which can lead to heart attacks and strokes.  The not-so-good news:  Excessive or heavy drinking can negate any potential heart health benefits and even increase cardiovascular risk factors:  1. Increased blood pressure: Excessive alcohol consumption can lead to high blood pressure, a major risk factor for heart disease.  2. Triglyceride increase: Heavy drinking can raise triglyceride levels, which can contribute to heart disease.  3. Weight gain and obesity: Alcoholic beverages are often high in calories, leading to weight gain and obesity, both of which increase cardiovascular risk.  4. Dehydration and electrolyte imbalance: Excessive drinking can cause dehydration and electrolyte imbalances, which can lead to heart rhythm disturbances and other cardiovascular problems.  Additional considerations:  1. Individual tolerance: Be aware of your personal tolerance to alcohol and its effects on your body. If you experience negative reactions, such as flushing, rapid heartbeat, or nausea, consider reducing or avoiding alcohol altogether.  2. Underlying health conditions: If you have certain health conditions, such as high blood pressure, heart failure, or liver disease, your doctor may advise against drinking or recommend stricter limits.  3. Medication interactions: Avoid drinking if you're taking medications that interact with alcohol, such as blood thinners, antidepressants, or medications for high blood pressure or heart conditions.  In summary:  If you choose to drink alcohol as part of a heart healthy diet, remember to:  * Consume in moderation (one drink per day for women, two drinks per day for men).  * Choose heart-healthy drinks, such as red wine, which is rich in antioxidants and polyphenols.  * Be mindful of your overall calorie intake and avoid excessive drinking, which can lead to weight gain and other cardiovascular risk factors.  * Consult with your healthcare provider if you have any concerns or underlying health conditions.  Remember, a heart healthy diet is just one aspect of maintaining overall cardiovascular well-being. Regular exercise, stress management, and not smoking are also essential for a healthy heart. |
| **Llama 3 Answer 2** | The age-old question: can I still enjoy a glass of wine or beer on a heart healthy diet?  The answer is... it's complicated. Moderate alcohol consumption may have some benefits for heart health, but it's essential to understand the risks and limitations.  The good news:  1. Moderate drinking may lower cardiovascular risk: Studies suggest that moderate alcohol consumption (1 drink per day for women, 2 drinks per day for men) may:  * Increase HDL (good) cholesterol  * Reduce inflammation  * Improve blood flow and lower blood pressure  * Decrease the risk of heart disease, stroke, and cardiovascular mortality  2. Antioxidants and polyphenols: Red wine, in particular, contains antioxidants and polyphenols, which may help protect against heart disease by reducing oxidative stress and improving vascular function.  The bad news:  1. Excessive drinking can harm the heart: Drinking more than the moderate amount can lead to:  * Increased blood pressure  * Cardiomyopathy (heart muscle damage)  * Arrhythmias (abnormal heart rhythms)  * Stroke and heart failure  2. Other health risks: Excessive alcohol consumption can also increase the risk of:  * Cancer (e.g., breast, colon, liver)  * Liver disease  * Pancreatitis  * Accidents and injuries  3. Interactions with medications: Alcohol can interact with certain medications, such as blood thinners, and exacerbate conditions like high blood pressure, diabetes, and atrial fibrillation.  4. Individual variability: People's bodies respond differently to alcohol, and some may be more susceptible to its negative effects.  Guidelines for heart healthy drinking:  1. Know your limits: Stick to moderate drinking: 1 drink per day for women, 2 drinks per day for men.  2. Choose wisely: Red wine, in moderation, may be a better choice than other types of alcohol due to its antioxidant content.  3. Be mindful of calories: Alcoholic drinks can be high in calories, which can contribute to weight gain and negatively impact heart health.  4. Consult your doctor: If you have a history of heart disease, high blood pressure, or other health conditions, consult with your doctor about safe alcohol consumption.  5. Don't start drinking for heart health: If you don't already drink, it's not recommended to start for heart health benefits alone. A heart healthy diet and lifestyle are more important for overall cardiovascular well-being.  In summary, moderate alcohol consumption may have some benefits for heart health, but it's crucial to be aware of the risks and limitations. Always prioritize a balanced diet, regular exercise, and a healthy lifestyle to support your heart health. |
| **Llama 3 Answer 3** | The age-old question: can I still enjoy a drink (or two) while keeping my heart healthy?  The answer is yes, but with some caveats. Moderate alcohol consumption, defined as one drink per day for women and two drinks per day for men, may be beneficial for heart health in some ways. However, excessive drinking can have negative effects on the heart and overall health.  Here are some points to consider:  Potential benefits:  1. Cardiovascular disease risk reduction: Moderate alcohol consumption may lower the risk of heart disease, including heart attacks, strokes, and peripheral artery disease.  2. Improved blood flow: Alcohol can help relax blood vessels, improving blood flow and reducing blood pressure.  3. Increased HDL cholesterol: Moderate drinking may increase high-density lipoprotein (HDL) cholesterol, the "good" cholesterol that helps remove excess cholesterol from the bloodstream.  Potential risks:  1. Excessive drinking: Consuming more than the recommended daily limit can increase the risk of heart disease, high blood pressure, and stroke.  2. Blood pressure increase: Regular heavy drinking can lead to high blood pressure, a major risk factor for heart disease.  3. Weight gain: Alcoholic beverages can be high in calories, contributing to weight gain and obesity, which increases the risk of heart disease.  4. Interference with medications: Alcohol can interact with certain medications, such as blood thinners, and decrease their effectiveness.  5. Other health risks: Excessive drinking is linked to an increased risk of various cancers, liver disease, and pancreatitis.  Guidelines for heart-healthy drinking:  1. Know your limits: Stick to the recommended daily limit: one drink per day for women and two drinks per day for men.  2. Choose wisely: Opt for red wine, which may have additional antioxidant benefits. However, it's essential to remember that the potential benefits of red wine are still being researched and debated.  3. Avoid binge drinking: Limit your drinking to special occasions and avoid consuming multiple drinks in one sitting.  4. Monitor your health: If you have high blood pressure, heart failure, or other heart conditions, consult with your doctor about safe drinking levels.  5. Stay hydrated: Drink water or other non-alcoholic beverages in between alcoholic drinks to avoid dehydration.  Remember, a heart-healthy diet is not just about what you drink, but also about maintaining a balanced diet rich in fruits, vegetables, whole grains, lean protein, and healthy fats. If you have concerns about your heart health or alcohol consumption, consult with your healthcare provider or a registered dietitian for personalized guidance. |
| **LLaMA3+RAG model Answer 1** | Moderate alcohol consumption, defined as an average of one to two drinks per day for men and one drink per day for women, is generally considered acceptable on a heart healthy diet. However, it's essential to keep in mind that drinking too much alcohol can increase the risk of various health problems, including high blood pressure, obesity, stroke, breast cancer, liver disease, depression, suicide, accidents, alcohol abuse, and alcoholism.  The American Heart Association (AHA) recommends moderation, and if you don't drink already, it's best not to start. If you do drink, it's crucial to talk with your doctor about the benefits and risks of consuming alcohol in moderation. Some individuals, such as pregnant or breastfeeding women, people under 21, and those with certain health conditions, should avoid drinking altogether.  Drinking alcohol can have both positive and negative effects on the body. On the one hand, moderate drinking may lead to a small increase in HDL (good) cholesterol. On the other hand, excessive drinking can raise triglyceride levels, increase the risk of heart attack and stroke, and lead to high blood pressure, cardiomyopathy, cardiac arrhythmia, and even death from alcohol poisoning.  It's also important to note that the potential health benefits associated with moderate drinking, such as a reduced risk of heart disease, may be due to other factors, such as a healthier diet and lifestyle, rather than the alcohol itself. Additionally, components like flavonoids and antioxidants found in red wine can also be obtained from other foods, such as grapes, red grape juice, or blueberries.  In conclusion, if you enjoy drinking alcohol, it's essential to do so in moderation and be aware of the potential risks. The AHA does not recommend drinking wine or any other form of alcohol to gain potential health benefits. Instead, focus on maintaining a healthy diet, staying physically active, and managing risk factors like high blood pressure, high cholesterol, and obesity.  Sources:  American Heart Association. (2024). Alcohol and Heart Health. Retrieved from <https://www.heart.org/en/healthy-living/healthy-eating/eat-smart/nutrition-basics/alcohol-and-heart-health>  American Heart Association. (1995). Moderate alcohol consumption and cardiovascular disease. Circulation, 94(11), 3023-3028. doi: 10.1161/01.CIR.94.11.3023 |
| **LLaMA3+RAG model Answer 2** | According to the American Heart Association (AHA), moderate alcohol consumption can be a part of a heart-healthy diet, but it's essential to keep moderation in mind to protect your health. Moderate alcohol consumption is defined as an average of one to two drinks per day for men and one drink per day for women. A drink is equivalent to one 12-ounce regular beer, 5 ounces of wine, or 1.5 ounces of 80-proof spirits.  Drinking too much alcohol can increase the risk of various health problems, including high blood pressure, obesity, stroke, breast cancer, liver disease, depression, suicide, accidents, alcohol abuse, and alcoholism. The AHA recommends that if you don't drink already, don't start. If you do drink, talk with your doctor about the benefits and risks of consuming alcohol in moderation.  It's also important to note that some people should not drink at all, including women who are pregnant or trying to get pregnant, people under age 21, and people with certain health conditions.  While some studies suggest that moderate drinking may have health benefits, such as a small increase in HDL (good) cholesterol, the AHA does not recommend drinking wine or any other form of alcohol to gain potential health benefits. Instead, it's recommended to focus on a healthy diet, regular physical activity, and other lifestyle changes to lower cholesterol, control high blood pressure, manage weight, and reduce the risk of cardiovascular disease.  If you drink every day, but not excessively, it's still important to be mindful of your consumption and consider cutting back if you find yourself drinking more and more over time. Binge drinking, in particular, can increase the risk of atrial fibrillation, an irregular or quivering heartbeat that can lead to blood clots, stroke, and heart failure.  In summary, while moderate alcohol consumption may be a part of a heart-healthy diet, it's crucial to prioritize moderation and be aware of the potential risks associated with excessive drinking.  Sources:  American Heart Association. (2024). Alcohol and Heart Health. Retrieved from <https://www.heart.org/en/healthy-living/healthy-eating/eat-smart/nutrition-basics/alcohol-and-heart-health>  American Heart Association. (1995). Moderate alcohol consumption and cardiovascular disease. Circulation, 94(11), 3023-3028. doi: 10.1161/01.CIR.94.11.3023 |
| **LLaMA3+RAG model Answer 3** | Drinking alcohol can be a part of a heart-healthy diet, but it's essential to do so in moderation. The American Heart Association (AHA) recommends that men limit their daily alcohol intake to one to two drinks, and women limit it to one drink per day. A drink is defined as 12 ounces of regular beer, 5 ounces of wine, or 1.5 ounces of 80-proof spirits.  Drinking too much alcohol can increase the risk of various health problems, including high blood pressure, obesity, stroke, breast cancer, liver disease, depression, suicide, accidents, alcohol abuse, and alcoholism. Excessive drinking can also raise triglyceride levels, lead to high blood pressure, cardiomyopathy, cardiac arrhythmia, and even death from alcohol poisoning.  While some studies suggest that moderate drinking may have health benefits, such as increasing HDL (good) cholesterol, the AHA does not recommend drinking wine or any other form of alcohol to gain potential health benefits. Instead, it's essential to focus on a healthy diet, regular physical activity, and managing other risk factors for heart disease.  It's also important to note that binge drinking, which is defined as having five or more drinks in two hours for men or four or more drinks for women, can increase the risk of atrial fibrillation, an irregular or quivering heartbeat that can lead to blood clots, stroke, and heart failure.  If you don't drink already, it's recommended not to start. If you do drink, it's essential to talk with your doctor about the benefits and risks of consuming alcohol in moderation. Some people, such as pregnant women, people under 21, and those with certain health conditions, should not drink at all.  In summary, moderate alcohol consumption can be a part of a heart-healthy diet, but it's crucial to be aware of the potential risks and to prioritize a healthy lifestyle, including a balanced diet, regular physical activity, and managing other risk factors for heart disease.  Sources:  American Heart Association. (2024). Alcohol and Heart Health. Retrieved from <https://www.heart.org/en/healthy-living/healthy-eating/eat-smart/nutrition-basics/alcohol-and-heart-health>  American Heart Association. (1995). Moderate alcohol consumption and cardiovascular disease. Circulation, 94(11), 3023-3028. doi: 10.1161/01.CIR.94.11.3023 |
| **Question 25** | **Should I follow a low carb diet to lower cholesterol?** |
| **Perplexity Answer 1** | A low-carb diet can be an effective way to improve cholesterol levels for many people, but the effects vary from person to person:  - On average, low-carb diets tend to improve cholesterol by lowering triglycerides and increasing HDL (good) cholesterol[1][3]. One meta-analysis found low-carb diets reduced total LDL and small LDL particles while increasing LDL particle size[3].  - However, for 5-25% of people, very low-carb diets can significantly increase LDL (bad) cholesterol levels, sometimes by 200% or more[3][4]. The reasons for this are still debated.  - The quality of fats and proteins on a low-carb diet matters. Diets high in vegetable fats and proteins are linked to a 30% lower heart disease risk compared to high-carb, low-fat diets. But diets high in animal fats and proteins do not show the same benefits[5].  - Cholesterol levels are just one factor in heart disease risk. Other important factors include blood pressure, inflammation, insulin resistance, and family history[4].  - For most people, the benefits of low-carb diets for weight loss, diabetes, and metabolic health likely outweigh any potential cholesterol concerns[4].  So in summary, a low-carb diet may help improve cholesterol for many, but a minority of people may see an undesirable rise in LDL. Focusing on healthy fats and proteins is important. If you have a strong family history of heart disease, it's wise to monitor your cholesterol closely when starting a low-carb diet. Discuss your individual situation with your doctor to determine the best approach for you.  Citations:  [1] https://corporate.dukehealth.org/news/study-shows-low-carb-diet-improves-cholesterol  [2] https://www.healthline.com/nutrition/low-carb-diets-and-cholesterol  [3] https://www.dietdoctor.com/low-carb/cholesterol-basics  [4] https://www.youtube.com/watch?v=rroy4ioG2fk  [5] https://www.hsph.harvard.edu/nutritionsource/carbohydrates/low-carbohydrate-diets/ |
| **Perplexity Answer 2** | A low-carb diet can be an effective way to improve cholesterol levels for many people, but the effects vary from person to person:  - On average, low-carb diets tend to improve cholesterol by lowering triglycerides and increasing HDL (good) cholesterol[1][3]. One meta-analysis found low-carb diets reduced total LDL and small LDL particles while increasing LDL particle size[3].  - However, a subset of people (estimated 5-25%) experience a significant increase in LDL (bad) cholesterol on a very low-carb or ketogenic diet[2][3]. The reasons for this are not fully understood.  - The quality of fats and proteins on a low-carb diet matters. Diets high in vegetable fats and proteins are linked to lower heart disease risk, while diets high in animal fats/proteins do not show the same benefits[5].  - For those with a family history of heart disease or other significant risk factors, it may be prudent to monitor cholesterol closely if starting a low-carb diet[2]. Simple adjustments like increasing fiber, olive oil, and fatty fish can help mitigate any LDL increases[2].  - Overall, low-carb diets have clear benefits for many heart disease risk factors like obesity, diabetes, and metabolic syndrome[2]. But the relationship between LDL cholesterol and heart disease risk is complex and not fully resolved, especially in the context of a healthy low-carb diet[3][4].  The decision to follow a low-carb diet should be made in consultation with your doctor, considering your individual health profile and preferences. For most, the benefits likely outweigh the risks, but monitoring cholesterol is advisable, especially for those with pre-existing heart disease or high risk.  Citations:  [1] https://corporate.dukehealth.org/news/study-shows-low-carb-diet-improves-cholesterol  [2] https://www.healthline.com/nutrition/low-carb-diets-and-cholesterol  [3] https://www.dietdoctor.com/low-carb/cholesterol-basics  [4] https://www.youtube.com/watch?v=rroy4ioG2fk  [5] https://www.hsph.harvard.edu/nutritionsource/carbohydrates/low-carbohydrate-diets/ |
| **Perplexity Answer 3** | A low-carb diet can be an effective way to improve cholesterol levels for many people, but the effects vary from person to person:  - On average, low-carb diets tend to improve cholesterol by lowering triglycerides and increasing HDL (good) cholesterol[1][3]. One meta-analysis found low-carb diets reduced total LDL and small LDL particles while increasing LDL particle size[3].  - However, a subset of people (estimated 5-25%) experience a significant increase in LDL (bad) cholesterol on a very low-carb or ketogenic diet[2][3]. The reasons for this are not fully understood.  - The quality of fats and proteins on a low-carb diet matters. Diets high in vegetable fats and proteins are linked to lower heart disease risk, while diets high in animal fats/proteins do not show the same benefits[5].  - Cholesterol levels are just one factor in heart disease risk. Other important factors include blood pressure, inflammation, insulin resistance, and family history[4].  - For those with high LDL on low-carb, adjustments like increasing fiber, plant sterols, and exercise may help lower levels[2]. But statin drugs are not always necessary if other risk factors are low.  In summary, a low-carb diet can be a good option to improve cholesterol for many, but individual responses vary. Consulting with your doctor to monitor cholesterol and other heart disease risk factors is recommended when making dietary changes. The overall quality of the diet is important for heart health.  Citations:  [1] https://corporate.dukehealth.org/news/study-shows-low-carb-diet-improves-cholesterol  [2] https://www.healthline.com/nutrition/low-carb-diets-and-cholesterol  [3] https://www.dietdoctor.com/low-carb/cholesterol-basics  [4] https://www.youtube.com/watch?v=rroy4ioG2fk  [5] https://www.hsph.harvard.edu/nutritionsource/carbohydrates/low-carbohydrate-diets/ |
| **GPT4o Answer 1** | A low-carb diet can be effective for some individuals in lowering cholesterol levels, but it depends on various factors such as your overall health, specific cholesterol profile, and how your body responds to dietary changes. Here are a few points to consider:  1. Impact on Lipid Profile: Studies have shown that low-carb diets can improve lipid profiles by increasing HDL (good cholesterol) and reducing triglycerides. However, the impact on LDL (bad cholesterol) can vary, with some people experiencing reductions and others seeing increases.  2. Weight Loss: Low-carb diets often lead to weight loss, which can positively affect cholesterol levels. Weight loss alone can help reduce LDL cholesterol and increase HDL cholesterol.  3. Types of Fats: The quality of fats consumed in a low-carb diet matters. Diets high in healthy fats, such as those from fish, nuts, seeds, and olive oil, can be beneficial. Conversely, diets high in saturated and trans fats can negatively impact cholesterol levels.  4. Individual Variability: People respond differently to dietary changes. Some may see significant improvements in cholesterol levels on a low-carb diet, while others may not see as much benefit or may even experience an increase in LDL cholesterol.  5. Other Health Considerations: It's important to consider other health factors such as diabetes, hypertension, and overall cardiovascular health when deciding on a diet.  6. Balanced Diet: Ensure that your diet remains balanced, with sufficient fiber intake from vegetables, fruits, and whole grains, which can also help lower cholesterol.  Before making any significant dietary changes, it's advisable to consult with a healthcare professional or a registered dietitian. They can help tailor a diet plan that meets your specific health needs and monitor your cholesterol levels to ensure the diet is having the desired effect. |
| **GPT4o Answer 2** | Following a low-carb diet can be an effective strategy for some individuals to lower cholesterol levels, particularly LDL cholesterol (the ""bad"" cholesterol) and triglycerides. However, it's important to approach this dietary change thoughtfully and consider the following points:  Benefits of a Low-Carb Diet:  1. Reduces Triglycerides: Low-carb diets often lead to a significant reduction in triglycerides, which are a type of fat in the blood associated with heart disease.  2. Increases HDL Cholesterol: These diets can help increase HDL (the ""good"" cholesterol), which helps remove LDL cholesterol from the bloodstream.  3. Improves Insulin Sensitivity: Improved insulin sensitivity can lead to better overall metabolic health, which is beneficial for heart health.  4. Weight Loss: Low-carb diets can promote weight loss, which can further help reduce cholesterol levels and improve heart health.  Considerations:  1. Type of Fats Consumed: Focus on healthy fats, such as those from fish, nuts, seeds, and avocados, rather than saturated and trans fats. Consuming unhealthy fats can negate the benefits.  2. Balance and Nutrient Intake: Ensure you are still getting adequate nutrients from vegetables, fruits, and whole grains. Completely eliminating these can lead to nutrient deficiencies.  3. Sustainability: Choose a diet that you can maintain long-term. Extreme low-carb diets may be hard to sustain over time and might not be necessary to achieve your cholesterol-lowering goals.  4. Individual Differences: Everyone’s body responds differently to dietary changes. Monitoring your cholesterol levels and overall health through regular check-ups is important.  Consulting a Healthcare Provider:  Before making significant changes to your diet, it's best to consult with a healthcare provider or a dietitian. They can provide personalized advice based on your current health status, cholesterol levels, and overall dietary needs. They can also monitor your progress and make adjustments as necessary.  Alternative Dietary Approaches:  Other dietary approaches, such as the Mediterranean diet or a diet rich in fiber and plant sterols, have also been shown to be effective in lowering cholesterol. These diets emphasize whole foods, healthy fats, and plant-based foods, which can be beneficial for heart health.  Summary:  A low-carb diet can help lower cholesterol for some individuals, but it's crucial to approach it in a balanced way, focusing on healthy fats and nutrient-dense foods. Consulting a healthcare provider can help tailor the approach to your specific needs and ensure it's safe and effective for you. |
| **GPT4o Answer 3** | Following a low-carb diet can potentially help lower cholesterol levels, but the effectiveness can vary from person to person. Here are some key points to consider:  Potential Benefits of a Low-Carb Diet on Cholesterol  1. Weight Loss: Low-carb diets often lead to weight loss, which can improve cholesterol levels.  2. Reduction in Triglycerides: Many people experience a significant drop in triglycerides, a type of fat in the blood, when they reduce carbohydrate intake.  3. Increase in HDL Cholesterol: Low-carb diets can increase high-density lipoprotein (HDL), the ""good"" cholesterol.  4. Changes in LDL Cholesterol: The impact on low-density lipoprotein (LDL), the ""bad"" cholesterol, can vary. Some people may see a reduction, while others might experience an increase. However, the LDL particles may become larger and less likely to cause arterial plaque.  Important Considerations  1. Type of Fats Consumed: Emphasize healthy fats from sources like avocados, nuts, seeds, and olive oil. Avoid trans fats and limit saturated fats from red meat and full-fat dairy.  2. Quality of Carbohydrates: Focus on high-fiber, low-glycemic-index carbs if you include them in your diet. Vegetables, legumes, and whole grains can be good choices.  3. Individual Variation: Genetic factors can influence how your cholesterol levels respond to dietary changes. It might be helpful to consult with a healthcare provider or a dietitian for personalized advice.  Alternative Dietary Approaches  - Mediterranean Diet: Emphasizes fruits, vegetables, whole grains, nuts, and healthy fats, which has been shown to improve heart health and cholesterol levels.  - DASH Diet: Focuses on reducing sodium and eating a variety of foods rich in nutrients that help lower blood pressure, which can also positively affect cholesterol levels.  Steps to Take  1. Monitor Your Cholesterol: Regular blood tests can help track changes in your cholesterol levels.  2. Consult with a Professional: A healthcare provider can offer guidance based on your health status and goals.  3. Adopt a Balanced Approach: Focus on overall dietary patterns rather than just one aspect. A balanced diet, regular exercise, and other lifestyle changes can collectively improve cholesterol levels.  Would you like more detailed information on specific low-carb diet plans or other dietary approaches to managing cholesterol |
| **Llama 3 Answer 1** | The eternal debate about low-carb diets and cholesterol!  While a low-carb diet may help with weight loss and improve certain health markers, its impact on cholesterol levels is more complex. Here's a balanced look at the evidence:  The good:  1. Weight loss: Low-carb diets can lead to weight loss, which is often associated with improved cholesterol profiles.  2. Improved triglycerides: Low-carb diets tend to lower triglyceride levels, which is a type of fat found in the blood. Elevated triglycerides are a risk factor for heart disease.  3. Increased HDL: Some studies suggest that low-carb diets may increase HDL (good) cholesterol levels.  The not-so-good:  1. Increased LDL: Low-carb diets, particularly those high in saturated and trans fats, may increase LDL (bad) cholesterol levels. This is a concern, as high LDL levels are a major risk factor for heart disease.  2. Limited evidence: The evidence for low-carb diets improving overall cholesterol profiles is mixed and often limited to specific populations, such as those with type 2 diabetes or obesity.  3. Potential negative effects on gut health: A low-carb diet may lead to a reduction in beneficial fiber intake, which can negatively impact gut health and overall cardiovascular health.  The American Heart Association's stance:  The American Heart Association (AHA) recommends a heart-healthy diet that is rich in fruits, vegetables, whole grains, lean protein sources, and healthy fats. They do not specifically recommend a low-carb diet for cholesterol management.  Who may benefit from a low-carb diet:  1. Individuals with insulin resistance or type 2 diabetes: Low-carb diets may help improve blood sugar control and insulin sensitivity.  2. Those with high triglycerides: Reducing carbohydrate intake may help lower triglyceride levels.  In conclusion:  While a low-carb diet may have some benefits, it is not the most effective or sustainable approach for lowering cholesterol levels. A more balanced diet that focuses on whole, nutrient-dense foods, such as:  * Fruits and vegetables  * Whole grains (like oats, barley, and quinoa)  * Lean protein sources (like poultry, fish, and legumes)  * Healthy fats (like avocado, nuts, and olive oil)  can help improve overall cardiovascular health and cholesterol profiles.  Before making significant changes to your diet, consult with a healthcare professional or registered dietitian to determine the best approach for your individual needs and health goals. |
| **Llama 3 Answer 2** | While a low-carb diet may help with weight loss and improve certain health markers, its effect on cholesterol levels is more nuanced. Here's a balanced look at the relationship between low-carb diets and cholesterol:  Theories behind a low-carb diet and cholesterol:  1. Reducing triglycerides: Low-carb diets often lead to a decrease in triglycerides, which are a type of fat found in the blood. Elevated triglycerides are associated with an increased risk of heart disease. By reducing triglycerides, a low-carb diet might help lower cholesterol levels.  2. Increasing HDL (good) cholesterol: Some studies suggest that low-carb diets may increase HDL (high-density lipoprotein) cholesterol, the "good" cholesterol that helps remove excess cholesterol from the bloodstream.  3. Decreasing small, dense LDL (bad) cholesterol: A low-carb diet may lead to a decrease in small, dense LDL cholesterol particles, which are associated with an increased risk of heart disease.  However, there are some concerns:  1. Increasing LDL (bad) cholesterol: Some low-carb diets, particularly those high in saturated and trans fats, may actually increase LDL cholesterol levels, which can increase the risk of heart disease.  2. Potential negative impact on cardiovascular health: A 2019 study published in The Lancet found that low-carb diets were associated with a higher risk of cardiovascular disease, including heart attacks, strokes, and cardiovascular deaths.  3. Lack of fiber and nutrients: Low-carb diets often restrict whole, unprocessed foods like fruits, vegetables, whole grains, and legumes, which are rich in fiber, vitamins, and minerals. This can lead to nutrient deficiencies and constipation.  Who may benefit from a low-carb diet for cholesterol management:  1. Individuals with insulin resistance or type 2 diabetes: A low-carb diet may help improve insulin sensitivity and blood sugar control, which can, in turn, lower cholesterol levels.  2. Those with high triglycerides: Reducing triglycerides through a low-carb diet may be beneficial for individuals with elevated levels.  In conclusion:  While a low-carb diet may have some benefits for cholesterol management, it's essential to approach this diet with caution and consideration. To minimize potential negative effects, focus on:  1. Whole, unprocessed foods: Emphasize whole, nutrient-dense foods like vegetables, fruits, lean proteins, and healthy fats.  2. Balanced macronutrient distribution: Ensure your diet includes a balanced mix of carbohydrates, protein, and healthy fats.  3. Monitor your cholesterol levels: Regularly check your cholesterol levels to ensure the diet is having a positive effect.  4. Consult a healthcare professional: Work with a registered dietitian, nutritionist, or healthcare provider to create a personalized diet plan that suits your needs and health goals.  Remember, a low-carb diet is not a one-size-fits-all solution for cholesterol management. Always prioritize a balanced, whole-food-based approach to support overall heart health |
| **Llama 3 Answer 3** | While a low-carb diet can be beneficial for weight loss and improving certain health markers, its impact on cholesterol levels is more nuanced. Let's dive into the details.  The relationship between carbs and cholesterol:  Carbohydrates, especially refined and high-glycemic ones, can raise triglycerides (a type of fat) and lower HDL (good) cholesterol. This is because carbs are broken down into glucose, which can stimulate the liver to produce more triglycerides. High triglycerides can contribute to high cholesterol.  Low-carb diets and cholesterol:  Some studies suggest that low-carb diets can lead to:  1. Increased HDL (good) cholesterol: By reducing carb intake, you may see an increase in HDL cholesterol, which can help remove excess cholesterol from the bloodstream.  2. Decreased triglycerides: Lowering carb consumption can lead to a decrease in triglycerides, which can contribute to high cholesterol.  3. Improved LDL (bad) cholesterol particle size: Low-carb diets may lead to an increase in larger, fluffier LDL particles, which are considered less harmful than smaller, denser particles.  However, it's essential to note that:  1. Not all low-carb diets are created equal: Some low-carb diets may be high in saturated fats, which can increase LDL cholesterol and negatively impact heart health.  2. Individual results may vary: People's responses to low-carb diets can differ significantly, and some may not experience improvements in cholesterol levels.  Other factors to consider:  1. Saturated and trans fats: Focus on reducing or avoiding saturated and trans fats, which can increase LDL cholesterol and negatively impact heart health.  2. Fiber intake: A high-fiber diet can help lower cholesterol levels by binding to bile acids and removing them from the body, which can help reduce cholesterol production.  3. Omega-3 fatty acids: Include sources of omega-3 fatty acids, such as fatty fish, nuts, and seeds, which can help improve heart health.  4. Weight loss: If you're overweight or obese, weight loss can help improve cholesterol levels, regardless of the diet you follow.  In conclusion:  While a low-carb diet may have some benefits for cholesterol levels, it's not a guarantee, and individual results may vary. A more effective approach might be to focus on a balanced diet that:  1. Emphasizes whole, minimally processed foods  2. Includes healthy fats, such as avocados, nuts, and olive oil  3. Is rich in fiber, omega-3 fatty acids, and antioxidants  4. Limits or avoids saturated and trans fats, added sugars, and refined carbohydrates  Consult with a healthcare professional or a registered dietitian to determine the best dietary approach for your individual needs and health goals. |
| **LLaMA3+RAG model Answer 1** | According to the American Heart Association, it is not recommended to follow a low-carb diet solely for the purpose of lowering cholesterol. While low-carb diets may lead to short-term weight loss, they are highly restrictive and difficult to sustain in the long term. Moreover, they often restrict consumption of fruits, grains, and legumes, which are essential for a heart-healthy diet.  The AHA's dietary guidance emphasizes the importance of consuming a balanced diet that is rich in non-starchy vegetables, fruits, whole grains, and legumes. These food groups provide essential nutrients, fiber, and antioxidants that help maintain healthy cholesterol levels and overall cardiovascular health.  In contrast, low-carb diets tend to be high in fat, particularly saturated fats, which can increase cholesterol levels and cardiovascular disease risk. The AHA's report suggests that low-carb diets align poorly with heart-healthy eating guidelines and have not been shown to be more effective for weight loss than less restrictive diets over the long term.  Instead, the AHA recommends following a heart-healthy eating pattern, such as the DASH diet, which is low in salt, added sugar, tropical oil, alcohol, and processed foods, and high in non-starchy vegetables, fruits, whole grains, and legumes. This eating pattern has been shown to be effective in reducing cholesterol levels and cardiovascular disease risk.  In conclusion, while a low-carb diet may lead to short-term weight loss, it is not a recommended approach for lowering cholesterol levels or promoting overall cardiovascular health. A balanced diet that emphasizes whole, nutrient-dense foods and is tailored to an individual's cultural and lifestyle needs is a more sustainable and effective approach for achieving and maintaining heart health.  Sources:  American Heart Association. (2023). Here's How 10 Popular Diets Scored for Heart Health. Retrieved from <https://www.heart.org/en/news/2023/04/27/heres-how-10-popular-diets-scored-for-heart-health>  American Heart Association. (2023). 2023 American Heart Association Dietary Guidance. Retrieved from <https://www.ahajournals.org/doi/full/10.1161/CIR.0000000000001146> |
| **LLaMA3+RAG model Answer 2** | According to the American Heart Association (AHA), it is not recommended to follow a low-carb diet solely for the purpose of lowering cholesterol. While low-carb diets may lead to short-term weight loss and potential cholesterol improvements, they have several drawbacks that make them less effective and sustainable for heart health in the long run.  Firstly, low-carb diets restrict the consumption of fruits, grains, and legumes, which are essential for a heart-healthy diet. These food groups are rich in fiber, vitamins, and minerals that help lower cholesterol levels and improve overall cardiovascular health. By limiting these foods, low-carb diets may lead to a decrease in fiber intake and an increase in saturated fat consumption, which can have negative effects on cholesterol levels and heart health.  Secondly, low-carb diets are often high in fat, without limiting saturated fats, which can increase cholesterol levels and heart disease risk. The AHA recommends limiting dietary saturated fat intake to no more than 5-6% of total daily calories.  Thirdly, low-carb diets are often highly restrictive and difficult to follow in the long term, which can lead to poor adherence and unsustainable weight loss. A diet that is effective for maintaining weight loss and improving heart health needs to be sustainable and culturally relevant.  Instead, the AHA recommends following a heart-healthy diet that emphasizes whole, unprocessed foods, such as fruits, vegetables, whole grains, lean proteins, and healthy fats. The DASH (Dietary Approaches to Stop Hypertension) eating plan, which scored a perfect 100 in the AHA's diet ranking, is a great example of a heart-healthy diet that can help lower cholesterol levels and improve overall cardiovascular health.  In conclusion, while low-carb diets may have some short-term benefits, they are not the most effective or sustainable choice for lowering cholesterol and improving heart health. A balanced and varied diet that emphasizes whole, unprocessed foods and is culturally relevant is a better approach for achieving and maintaining heart health.  Sources:  American Heart Association. (2023). Here's How 10 Popular Diets Scored for Heart Health. Retrieved from <https://www.heart.org/en/news/2023/04/27/heres-how-10-popular-diets-scored-for-heart-health>  American Heart Association. (2023). 2023 Presidential Advisory: Dietary Guidance to Improve Cardiovascular Health. Retrieved from <https://www.ahajournals.org/doi/full/10.1161/CIR.0000000000001146>  American Heart Association. (2020). Healthy Eating Plate. Retrieved from <https://www.heart.org/en/healthy-living/healthy-eating/eat-smart/nutrition-basics/healthy-eating-plate> |
| **LLaMA3+RAG model Answer 3** | According to the American Heart Association (AHA) it is not recommended to follow a low-carb diet solely for the purpose of lowering cholesterol. According to the American Heart Association (AHA), low-carb diets fall into the third tier of diets, which restrict foods that are emphasized in the AHA's dietary guidance. Specifically, low-carb diets restrict consumption of fruits, grains, and legumes, which can lead to eating less fiber and higher amounts of saturated fats.  The AHA's scoring system, which ranged from 31 to 100, placed low-carb diets at a score of 64, indicating that they do not align well with heart-healthy eating guidelines. Furthermore, the AHA notes that very low-carb/ketogenic diets, which are even more restrictive, fall into the bottom tier with a score of 31.  Instead, the AHA recommends following a diet that is rich in non-starchy vegetables, fruits, whole grains, and legumes, such as the DASH eating plan, which received a perfect score of 100. This eating pattern is low in salt, added sugar, tropical oil, alcohol, and processed foods and high in nutrients from plant-based sources.  It's also important to note that a diet should be sustainable and culturally relevant to ensure its effectiveness. As Dr. Gardner mentioned, ""A diet that's effective at helping an individual maintain weight-loss goals, from a practical perspective, needs to be sustainable.""  In conclusion, while a low-carb diet may lead to short-term weight loss, it is not a recommended approach for lowering cholesterol or promoting overall heart health due to its restrictive nature and potential negative impact on fiber and saturated fat intake.  Sources:  1. American Heart Association. (2023). Here's How 10 Popular Diets Scored for Heart Health. Retrieved from <https://www.heart.org/en/news/2023/04/27/heres-how-10-popular-diets-scored-for-heart-health>  2. American Heart Association. (2023). 2023 AHA Diet and Lifestyle Recommendations. Retrieved from <https://www.ahajournals.org/doi/full/10.1161/CIR.0000000000001146>  Note: The provided context is based on the American Heart Association's guidelines and research, which are considered credible sources in the field of nutrition and heart health. |
| **Question 26** | **How much protein should I eat per day as a part of a heart healthy diet?** |
| **Perplexity Answer 1** | To maintain a heart-healthy diet, experts recommend getting 10-30% of your daily calories from protein[2]. The specific amount depends on your individual calorie needs, but a general guideline is:  - Women (ages 19+): 46 grams of protein per day[2]  - Men (ages 19+): 56 grams of protein per day[2]  However, consuming more than 22% of daily calories from protein may increase the risk of atherosclerosis and worsen heart health[1]. Eating 58-74 grams of protein per day, which is slightly higher than the recommended amount, was associated with a 5-8% lower risk of atrial fibrillation in women[3].  The type of protein is also important. Plant-based proteins like legumes, nuts, and soy are ideal, as they provide protein along with beneficial nutrients like fiber, vitamins, and minerals[2][5]. Animal proteins should be lean and low in saturated fat, such as poultry, fish, and low-fat dairy[2].  Spreading protein intake evenly throughout the day, aiming for 20-30 grams per meal, is recommended[1]. Consuming excessive amounts of protein in one sitting is not beneficial and may even be harmful to heart health[1].  In summary, aim for 10-30% of your daily calories from a variety of lean, plant-based and low-fat animal proteins, spread evenly throughout the day. Consult with a registered dietitian to determine your individual protein needs as part of an overall heart-healthy diet.  Citations:  [1] https://www.medicalnewstoday.com/articles/eating-this-much-protein-can-be-bad-for-your-heart-heath  [2] https://www.healthline.com/health/heart-disease/heart-healthy-proteins  [3] https://www.bicycling.com/news/a31979809/eating-more-protein-could-help-your-heart-study/  [4] https://www.ncbi.nlm.nih.gov/pmc/articles/PMC10058321/  [5] https://www.heartfoundation.org.au/healthy-living/healthy-eating/protein-and-heart-health |
| **Perplexity Answer 2** | To maintain a heart-healthy diet, experts recommend getting 10-30% of your daily calories from protein[2]. The specific amount depends on your individual calorie needs, but a general guideline is:  - Women (ages 19+): 46 grams of protein per day[2]  - Men (ages 19+): 56 grams of protein per day[2]  However, consuming more than 22% of daily calories from protein may increase the risk of atherosclerosis and worsen heart health[1]. Eating 58-74 grams of protein per day has been associated with a 5-8% lower risk of atrial fibrillation in women[3].  The type of protein is also important. Plant-based proteins like legumes, nuts, and soy are ideal, as they provide protein along with beneficial nutrients like fiber, vitamins, and minerals[2][4][5]. Animal proteins should be lean, such as poultry, fish, and low-fat dairy[2][5].  In summary, aim for 20-30 grams of protein per meal from a variety of healthy sources, which equates to about 10-30% of total daily calories for most adults[1][2][4]. Avoid excessive protein intake, especially from animal sources, to maintain a heart-healthy diet.  Citations:  [1] https://www.medicalnewstoday.com/articles/eating-this-much-protein-can-be-bad-for-your-heart-heath  [2] https://www.healthline.com/health/heart-disease/heart-healthy-proteins  [3] https://www.bicycling.com/news/a31979809/eating-more-protein-could-help-your-heart-study/  [4] https://www.ncbi.nlm.nih.gov/pmc/articles/PMC10058321/  [5] https://www.heartfoundation.org.au/healthy-living/healthy-eating/protein-and-heart-health |
| **Perplexity Answer 3** | To maintain a heart-healthy diet, experts recommend getting 10-30% of your daily calories from protein[2]. The specific amount depends on your individual calorie needs, but a general guideline is:  - Women (ages 19+): 46 grams of protein per day[2]  - Men (ages 19+): 56 grams of protein per day[2]  However, consuming more than 22% of daily calories from protein may increase the risk of atherosclerosis and worsen heart health[1]. Eating 58-74 grams of protein per day, which is slightly higher than the recommended amount, was associated with a 5-8% lower risk of atrial fibrillation in women[3].  The type of protein is also important. Plant-based proteins like legumes, nuts, and soy are ideal, as they provide protein along with beneficial nutrients like fiber, vitamins, and minerals[2][5]. Animal proteins should be lean cuts of poultry, fish, and low-fat dairy[2].  In summary, aim for 20-30 grams of protein per meal from a variety of healthy sources, which equates to about 10-30% of your total daily calories[1][2][4]. Consult with a registered dietitian to determine your individual protein needs as part of an overall heart-healthy diet.  Citations:  [1] https://www.medicalnewstoday.com/articles/eating-this-much-protein-can-be-bad-for-your-heart-heath  [2] https://www.healthline.com/health/heart-disease/heart-healthy-proteins  [3] https://www.bicycling.com/news/a31979809/eating-more-protein-could-help-your-heart-study/  [4] https://www.ncbi.nlm.nih.gov/pmc/articles/PMC10058321/  [5] https://www.heartfoundation.org.au/healthy-living/healthy-eating/protein-and-heart-health |
| **GPT4o Answer 1** | The amount of protein you should eat per day as part of a heart-healthy diet depends on various factors, including your age, sex, weight, physical activity level, and overall health goals. However, general guidelines can help you determine an appropriate amount:  1. Recommended Dietary Allowance (RDA): For the average adult, the RDA for protein is 0.8 grams per kilogram of body weight. For example, if you weigh 70 kg (about 154 pounds), you would need about 56 grams of protein per day.  2. Heart-Healthy Diet Considerations:  - Lean Protein Sources: Choose lean sources of protein such as poultry, fish, beans, legumes, nuts, and seeds. Fish high in omega-3 fatty acids (e.g., salmon, mackerel, sardines) are particularly beneficial for heart health.  - Plant-Based Proteins: Incorporating plant-based proteins like beans, lentils, tofu, and quinoa can help reduce cholesterol levels and provide additional nutrients beneficial for heart health.  - Low Saturated Fat: Limit red meat and high-fat dairy products, which are high in saturated fats and can raise cholesterol levels.  3. Distribution Throughout the Day: Aim to distribute your protein intake evenly throughout the day to help maintain muscle mass and support metabolic functions.  Example Protein Needs  For a 70 kg (154 lbs) adult:  - RDA Calculation: 70 kg x 0.8 g/kg = 56 grams of protein per day.  - Active Individuals: Those who are more physically active may require more protein, ranging from 1.0 to 1.6 grams per kilogram of body weight.  Practical Tips  - Include a source of protein in every meal and snack.  - Diversify your protein sources to ensure a broad spectrum of nutrients.  - Focus on portion control to avoid overconsumption of protein-rich foods, which can add extra calories and stress the kidneys.  Always consider consulting with a healthcare provider or a registered dietitian to tailor your protein intake to your specific health needs and goals. |
| **GPT4o Answer 2** | The amount of protein you should consume daily can depend on various factors, including your age, sex, physical activity level, and overall health. However, general guidelines for a heart-healthy diet and protein intake are as follows:  1. General Recommendations:  - The Dietary Reference Intake (DRI) for protein is 0.8 grams of protein per kilogram of body weight.  - For an average adult, this equates to about 46 grams per day for women and 56 grams per day for men.  2. Heart-Healthy Diet:  - Lean Protein Sources: Opt for lean meats, poultry, fish, beans, lentils, and plant-based proteins like tofu and tempeh.  - Fish: The American Heart Association recommends eating fish, particularly fatty fish like salmon, mackerel, and sardines, at least twice a week. These are rich in omega-3 fatty acids, which are beneficial for heart health.  - Plant-Based Proteins: Increasing intake of plant-based proteins such as beans, legumes, and nuts can improve heart health. These foods are generally lower in saturated fat and contain beneficial fiber and other nutrients.  - Limit Red Meat: Try to limit red meat and processed meats, as they can be high in saturated fat and cholesterol.  3. Protein Quality:  - Focus on high-quality protein sources. For instance, fatty fish not only provides protein but also omega-3 fatty acids.  - Plant-based sources like quinoa, chia seeds, and buckwheat are complete proteins, meaning they contain all essential amino acids.  4. Balanced Diet:  - Ensure your diet is balanced, with adequate amounts of fruits, vegetables, whole grains, and healthy fats. This balance is crucial for heart health.  - Monitor overall calorie intake to maintain a healthy weight, as obesity is a risk factor for heart disease.  Example Calculation  If you weigh 70 kg (approximately 154 lbs):  - Protein Requirement: 70 kg x 0.8 g/kg = 56 grams of protein per day.  Adjustments for Specific Needs  - Active Individuals: If you are highly active or an athlete, your protein needs might be higher, ranging from 1.2 to 2.0 grams per kilogram of body weight.  - Older Adults: Older adults may need slightly more protein to help maintain muscle mass, typically around 1.0 to 1.2 grams per kilogram of body weight.  Consulting with a healthcare provider or a registered dietitian can provide personalized recommendations based on your specific health needs and goals. |
| **GPT4o Answer 3** | The amount of protein you should consume daily as part of a heart-healthy diet can depend on several factors including your age, sex, weight, and level of physical activity. However, general guidelines can provide a starting point:  1. General Recommendations:  - The Dietary Reference Intake (DRI) suggests that the average adult should aim for about 0.8 grams of protein per kilogram of body weight. For example, if you weigh 70 kilograms (about 154 pounds), you should aim for about 56 grams of protein per day.  - For adults, protein should make up about 10-35% of total daily calories.  2. Heart-Healthy Considerations:  - Choose lean protein sources such as fish, poultry, beans, legumes, nuts, and seeds. Fish, especially fatty fish like salmon, mackerel, and sardines, are particularly good as they are rich in omega-3 fatty acids which are beneficial for heart health.  - Limit red meat and processed meats, which are high in saturated fats and can increase the risk of heart disease.  - Incorporate plant-based proteins such as soy, lentils, and quinoa which are associated with lower cholesterol levels and better heart health.  3. Age and Activity Level:  - Older adults: May need slightly more protein to help maintain muscle mass. Aim for around 1.0-1.2 grams per kilogram of body weight.  - Active individuals and athletes: May need more protein to support muscle repair and growth. The requirement can go up to 1.2-2.0 grams per kilogram of body weight, depending on the intensity and type of physical activity.  4. Specific Conditions:  - If you have certain health conditions, such as kidney disease, you may need to adjust your protein intake accordingly. It's important to consult with a healthcare provider for personalized advice.  Example Calculation:  For a 70 kg (154 lbs) adult with moderate activity levels:  - Minimum: 70 kg x 0.8 g/kg = 56 grams of protein per day.  - Moderate activity: 70 kg x 1.2 g/kg = 84 grams of protein per day.  Food Sources:  - Fish: 3 ounces of salmon has about 22 grams of protein.  - Poultry: 3 ounces of chicken breast has about 26 grams of protein.  - Legumes: 1 cup of cooked lentils has about 18 grams of protein.  - Nuts: 1 ounce of almonds has about 6 grams of protein.  To maintain a heart-healthy diet, focus on the quality of protein sources and balance it with other nutrient-dense foods like vegetables, fruits, whole grains, and healthy fats. |
| **Llama 3 Answer 1** | As part of a heart-healthy diet, the recommended daily protein intake varies based on factors such as age, sex, weight, and activity level. Here are some general guidelines:  General Recommendations:  * The American Heart Association (AHA) recommends that adults consume 0.8-1.2 grams of protein per kilogram of body weight (about 56-91 grams per day for a 154-pound person) as part of a heart-healthy diet.  * The Academy of Nutrition and Dietetics recommends 1.2-1.6 grams of protein per kilogram of body weight (about 82-109 grams per day for a 154-pound person) for overall health and well-being.  Additional Considerations:  * If you're physically active or engaging in resistance training, you may need more protein to support muscle growth and repair. Aim for 1.6-2.2 grams of protein per kilogram of body weight (about 109-136 grams per day for a 154-pound person).  * If you have kidney disease or are at risk for kidney disease, you may need to limit your protein intake to 0.8-1.0 gram per kilogram of body weight (about 56-77 grams per day for a 154-pound person).  Food Sources:  * Focus on whole, unprocessed foods like:  + Lean meats: poultry, fish, and pork tenderloin  + Legumes: beans, lentils, and peas  + Nuts and seeds: almonds, chia seeds, and flaxseeds  + Whole grains: quinoa, brown rice, and whole-wheat bread  + Dairy: low-fat milk, yogurt, and cheese  + Eggs and egg products  + Plant-based protein powders (if you're unable to get enough protein from whole foods)  Tips:  * Spread your protein intake throughout the day, aiming for 20-30 grams per meal and 10-15 grams per snack.  * Choose protein sources that are low in saturated fat, sodium, and added sugars.  * Consider consulting with a registered dietitian or healthcare professional to determine your individual protein needs and develop a personalized heart-healthy diet plan.  Remember, a heart-healthy diet is not just about protein intake, but also includes a balanced mix of fruits, vegetables, whole grains, lean protein sources, and healthy fats. |
| **Llama 3 Answer 2** | As part of a heart-healthy diet, the recommended daily intake of protein varies based on factors such as age, sex, weight, and overall health status. Here are some general guidelines:  General Recommendations:  * The American Heart Association (AHA) recommends that adults consume at least 0.8 grams of protein per kilogram of body weight (about 56 grams per day for a 154-pound person) as part of a balanced diet.  * The Dietary Reference Intake (DRI) established by the National Academies of Sciences, Engineering, and Medicine recommends a daily protein intake of 0.8-1.2 grams per kilogram of body weight (about 56-84 grams per day for a 154-pound person).  Heart-Healthy Protein Intake:  * The AHA suggests that increasing protein intake to 1.0-1.2 grams per kilogram of body weight (about 68-84 grams per day for a 154-pound person) may help with weight management and improve overall cardiovascular health.  * Some research suggests that a higher protein intake of 1.2-1.6 grams per kilogram of body weight (about 84-112 grams per day for a 154-pound person) may be beneficial for heart health, particularly in individuals with high blood pressure or cardiovascular disease.  Food Sources:  * Focus on consuming protein from plant-based sources, such as:  + Legumes (beans, lentils, peas)  + Nuts and seeds (almonds, chia seeds, hemp seeds)  + Whole grains (quinoa, brown rice, whole wheat)  + Soy products (tofu, tempeh, edamame)  * Also include protein from lean animal sources, such as:  + Poultry (chicken, turkey)  + Fish (fattier fish like salmon, tuna, and mackerel are high in omega-3 fatty acids)  + Low-fat dairy products (milk, yogurt, cheese)  Tips:  * Spread protein intake throughout the day, aiming for 20-30 grams per meal and 10-15 grams per snack.  * Choose a variety of protein sources to ensure you're getting all essential amino acids.  * Be mindful of overall calorie intake and balance protein with healthy fats and complex carbohydrates.  Remember, individual protein needs may vary. Consult with a healthcare professional or registered dietitian to determine the best protein intake for your specific needs and health goals. |
| **Llama 3 Answer 3** | As part of a heart-healthy diet, the recommended daily protein intake can vary based on factors such as age, sex, weight, and activity level. Here are some general guidelines:  General Recommendations:  * The American Heart Association (AHA) recommends that adult woman consume at least 46 grams of protein per day, and adult men consume at least 56 grams per day.  * The Dietary Reference Intake (DRI) established by the National Institutes of Health recommends a daily protein intake of 0.8 grams per kilogram of body weight (or about 56 grams per day for a 150-pound person).  Heart Health-Specific Recommendations:  * The AHA suggests that people with high blood pressure, high cholesterol, or heart failure may benefit from a higher protein intake, up to 1.2-1.6 grams per kilogram of body weight (or about 80-120 grams per day for a 150-pound person).  * The American College of Cardiology (ACC) and the AHA recommend a protein intake of 1.0-1.2 grams per kilogram of body weight (or about 70-90 grams per day for a 150-pound person) for individuals with heart disease or at high risk of heart disease.  Key Considerations:  * Choose protein sources that are rich in nutrients and low in saturated and trans fats, sodium, and added sugars. Good options include:  + Lean meats: poultry, fish, and pork tenderloin  + Legumes: beans, lentils, and peas  + Nuts and seeds: almonds, walnuts, chia seeds, and flaxseeds  + Whole grains: quinoa, brown rice, and whole-wheat bread  + Low-fat dairy: milk, yogurt, and cheese  * Consider your individual protein needs based on your age, sex, weight, and activity level. For example, older adults or those who are physically active may require more protein.  * Consult with a registered dietitian or healthcare provider to determine the best protein intake for your specific heart health needs.  Remember, a heart-healthy diet is not just about protein intake; it's also important to focus on consuming a balanced diet rich in fruits, vegetables, whole grains, and healthy fats, while limiting intake of sugary drinks, saturated and trans fats, and sodium. |
| **LLaMA3+RAG model Answer 1** | As a part of a heart-healthy diet, the recommended daily intake of protein varies based on age, weight, and other factors. According to the Centers for Disease Control and Prevention (CDC), the recommended daily allowance (RDA) for protein is 10% to 35% of your daily calories. This translates to approximately 46 grams of protein per day for adult women and 56 grams per day for adult men.  To put this into perspective, according to the American Heart Association here are some examples of protein-rich foods and their corresponding protein content:  8-ounce glass of milk: 8 grams of protein  1 cup of yogurt: 11 grams of protein  3-ounce piece of meat (about the size of a deck of cards): 21 grams of protein  1 cup of dry beans: 16 grams of protein  To achieve the recommended daily intake of protein, you can aim to include a variety of protein-rich foods in your diet, such as lean meats, fish, poultry, beans, lentils, nuts, and seeds. It's also important to choose low-fat options and watch portion sizes, aiming for 2- to 3-ounce servings.  In addition to the amount of protein, it's also important to consider the type of protein you're consuming. Opt for protein sources that are low in saturated fat, such as lean meats, skim milk, and legumes. Legumes, for example, are a low-fat and inexpensive alternative to meat and can provide about 16 grams of protein per cup.  By incorporating a balanced mix of protein-rich foods into your diet and being mindful ofportun sizes, you can help support heart health and overall well-being.  Sources:  American Heart Association. (2024). Protein and Heart Health. Retrieved from <https://www.heart.org/en/healthy-living/healthy-eating/eat-smart/nutrition-basics/protein-and-heart-health>  Centers for Disease Control and Prevention. (2020). Protein. Retrieved from <https://www.cdc.gov/nutrition/data-statistics/protein.html> |
| **LLaMA3+RAG model Answer 2** | As part of a heart-healthy diet, the recommended daily intake of protein varies based on age, weight, and other factors. According to the Centers for Disease Control and Prevention, it is recommended that 10% to 35% of daily calories come from protein. This translates to approximately 46 grams of protein per day for adult women and 56 grams per day for adult men.  To put this into perspective, according to the American Heart Association here are some examples of protein-rich foods and their corresponding protein content:  8-ounce glass of milk: 8 grams of protein  1 cup of yogurt: 11 grams of protein  3-ounce piece of meat (about the size of a deck of cards): 21 grams of protein  1 cup of dry beans: 16 grams of protein  To achieve the recommended daily intake, you can aim to include a variety of protein-rich foods in your diet, such as lean meats, legumes, dairy products, and plant-based options. It's also important to choose low-fat options and watch portion sizes, aiming for 2- to 3-ounce servings.  Additionally, consider the following tips to incorporate protein into your diet:  Opt for lean protein sources, such as poultry, fish, and legumes, which are lower in fat and higher in protein.  Choose main dishes that combine protein and vegetables, such as low-fat soups or stir-fries.  Incorporate plant-based protein sources, such as beans, lentils, and tofu, into your meals.  Limit portion sizes and avoid excessive protein intake, as this can put a strain on your kidneys and liver.  By following these guidelines and incorporating a balanced mix of protein-rich foods into your diet, you can support overall heart health and well-being.  Sources:  American Heart Association. (2024). Protein and Heart Health. Retrieved from <https://www.heart.org/en/healthy-living/healthy-eating/eat-smart/nutrition-basics/protein-and-heart-health>  Centers for Disease Control and Prevention. (2020). Protein. Retrieved from <https://www.cdc.gov/nutrition/data-statistics/protein.html> |
| **LLaMA3+RAG model Answer 3** | As part of a heart-healthy diet, the recommended daily intake of protein varies based on age, weight, and other factors. According to the Centers for Disease Control and Prevention (CDC), it is recommended that 10% to 35% of your daily calories come from protein. This translates to approximately 46 grams of protein per day for adult women and 56 grams per day for adult men.  To put this into perspective, according to the American Heart Association here are some examples of protein-rich foods and their corresponding protein content:  8-ounce glass of milk: 8 grams of protein  1 cup of yogurt: 11 grams of protein  3-ounce piece of meat (about the size of a deck of cards): 21 grams of protein  1 cup of dry beans: 16 grams of protein  To achieve the recommended daily intake of protein, you can aim to include a variety of protein-rich foods in your diet, such as lean meats, legumes, dairy products, and plant-based alternatives. It's also important to choose low-fat options and watch portion sizes to maintain a balanced diet.  Here are some additional tips for incorporating protein into your heart-healthy diet:  Opt for lean meats, such as poultry, fish, and pork tenderloin, which are lower in fat and higher in protein.  Choose low-fat dairy products, such as skim milk and low-fat yogurt, which are rich in protein and calcium.  Legumes, such as beans, lentils, and peas, are a low-fat and inexpensive alternative to meat and are rich in protein, fiber, and other essential nutrients.  Incorporate plant-based protein sources, such as tofu, tempeh, and seitan, into your diet.  Watch portion sizes and aim for 2- to 3-ounce servings of protein-rich foods per meal.  Combine protein-rich foods with vegetables and whole grains to create balanced and satisfying meals.  By following these guidelines and incorporating a variety of protein-rich foods into your diet, you can help maintain a healthy heart and overall well-being.  Sources:  American Heart Association. (2024). Protein and Heart Health. Retrieved from <https://www.heart.org/en/healthy-living/healthy-eating/eat-smart/nutrition-basics/protein-and-heart-health>  Centers for Disease Control and Prevention. (2020). Protein. Retrieved from <https://www.cdc.gov/nutrition/data-statistics/protein.html> |
| **Question 27** | **Can I drink caffeine on a heart healthy diet?** |
| **Perplexity Answer 1** | Yes, you can still drink caffeine in moderation as part of a heart-healthy diet. Here are the key points:  - Moderate caffeine consumption of 200-300 mg per day, equivalent to about 2-3 cups of coffee, is generally considered safe for heart health in healthy adults[1][2][4].  - Recent studies have found that regular coffee intake, especially 2-3 cups per day, is associated with a lower risk of heart disease, heart failure, heart rhythm problems, and death in people both with and without existing heart disease[4].  - Caffeine may have some beneficial effects on the cardiovascular system, such as improving blood vessel function and reducing inflammation[1][3].  - However, caffeine can affect each person differently based on factors like genetics, health conditions, and medications. People with certain heart rhythm disorders may need to limit or avoid caffeine[2].  - It's important to be mindful of how caffeine makes you feel individually. Consuming too much can cause jitteriness, insomnia, digestive issues and other side effects[2][3].  - The type of coffee matters too. Stick to plain black coffee and avoid sugary, creamy coffee drinks that can negate the health benefits[3].  So in moderation, caffeine can be part of an overall heart-healthy lifestyle that includes a nutritious diet, regular exercise, and stress management. But it's best to discuss your individual caffeine intake with your doctor, especially if you have heart disease or other health conditions.  Citations:  [1] https://www.premierhealth.com/your-health/articles/women-wisdom-wellness-/what-s-the-buzz-on-caffeine-and-heart-health-  [2] https://health.ucdavis.edu/news/headlines/qa-what-effect-does-caffeine-have-on-your-heart/2023/12  [3] https://www.heart.org/en/news/2018/09/28/is-coffee-good-for-you-or-not  [4] https://www.acc.org/About-ACC/Press-Releases/2022/03/23/17/55/Good-News-for-Coffee-Lovers-Daily-Coffee-May-Benefit-the-Heart  [5] https://www.ncbi.nlm.nih.gov/pmc/articles/PMC10262944/ |
| **Perplexity Answer 2** | Yes, you can still drink caffeine in moderation as part of a heart-healthy diet. Here are the key points:  - For most people, drinking up to 4-5 cups of coffee per day is considered safe and may even provide some health benefits[1][3]. However, the effects can vary from person to person.  - Caffeine can increase heart rate and blood pressure, but this is usually well-tolerated. However, those prone to atrial fibrillation or fast heart rates may see an increase with consumption[1].  - Drinking coffee, particularly 2-3 cups per day, is associated with a lower risk of heart disease, dangerous heart rhythms, and living longer, even for those with existing heart disease[4]. The maximum benefit is seen with 2-3 cups daily.  - Caffeine is found in many foods and drinks beyond just coffee, such as chocolate, sodas, energy drinks, and supplements[1]. Be mindful of your total caffeine intake from all sources.  - Avoid adding too much cream and sugar to coffee, as that can negate some of the health benefits[3]. Stick to plain black coffee or tea.  - Pay attention to how caffeine affects you individually. Some people are slow metabolizers and may experience jitters, palpitations or insomnia with intake[3].  So in moderation, caffeine can be part of an overall heart-healthy lifestyle that includes a balanced diet, regular exercise, and stress management. But be sure to discuss your specific caffeine intake with your doctor.  Citations:  [1] https://health.ucdavis.edu/news/headlines/qa-what-effect-does-caffeine-have-on-your-heart/2023/12  [2] https://www.healthline.com/health/heart-disease/diet-sugar-caffeine-calcium-alcohol  [3] https://www.heart.org/en/news/2018/09/28/is-coffee-good-for-you-or-not  [4] https://www.acc.org/About-ACC/Press-Releases/2022/03/23/17/55/Good-News-for-Coffee-Lovers-Daily-Coffee-May-Benefit-the-Heart  [5] https://www.ncbi.nlm.nih.gov/pmc/articles/PMC10262944/ |
| **Perplexity Answer 3** | Yes, you can still drink caffeine in moderation as part of a heart-healthy diet. Here are the key points:  - For most people, drinking up to 4-5 cups of coffee per day is considered safe and may even provide some health benefits[1][3]. However, the effects can vary from person to person.  - Caffeine can increase heart rate and blood pressure, but this is usually well-tolerated. However, those prone to atrial fibrillation or fast heart rates may see an increase with consumption[1].  - Drinking 2-3 cups of coffee per day is associated with a 10-15% lower risk of developing coronary heart disease, heart failure, heart rhythm problems, and lower mortality risk compared to no coffee consumption[4].  - The American Heart Association recommends limiting caffeine, especially from sugary energy drinks and sodas. Stick to plain black coffee or tea to avoid extra calories from cream and sugar[3].  - Pay attention to how caffeine affects you individually. Some people are slow caffeine metabolizers and may experience jitters, palpitations or insomnia from even moderate amounts[3].  So in moderation, caffeine can be part of a heart-healthy lifestyle. But be mindful of your personal tolerance and avoid excessive consumption, especially from sugary sources. Consult your doctor if you have any concerns about how caffeine affects your heart health.  Citations:  [1] https://health.ucdavis.edu/news/headlines/qa-what-effect-does-caffeine-have-on-your-heart/2023/12  [2] https://www.healthline.com/health/heart-disease/diet-sugar-caffeine-calcium-alcohol  [3] https://www.heart.org/en/news/2018/09/28/is-coffee-good-for-you-or-not  [4] https://www.acc.org/About-ACC/Press-Releases/2022/03/23/17/55/Good-News-for-Coffee-Lovers-Daily-Coffee-May-Benefit-the-Heart  [5] https://www.ncbi.nlm.nih.gov/pmc/articles/PMC10262944/ |
| **GPT4o Answer 1** | Yes, you can still drink caffeine on a heart-healthy diet, but it's important to consume it in moderation. Here are some guidelines to consider:  1. Moderation: Limit caffeine intake to moderate amounts. This typically means up to 400 milligrams per day, which is roughly equivalent to four 8-ounce cups of brewed coffee.  2. Monitor Effects: Pay attention to how your body reacts to caffeine. If you notice any adverse effects such as palpitations, increased heart rate, or blood pressure spikes, consider reducing your intake or avoiding caffeine altogether.  3. Sources of Caffeine: Be mindful of where your caffeine comes from. Coffee, tea, and certain types of soda are common sources, but energy drinks and certain medications also contain caffeine.  4. Decaf Options: If you enjoy the taste of coffee or tea but want to limit your caffeine intake, consider decaffeinated versions.  5. Overall Diet: Ensure that the rest of your diet supports heart health. This includes eating plenty of fruits, vegetables, whole grains, lean proteins, and healthy fats while minimizing sodium, saturated fats, and added sugars.  6. Consult Your Doctor: If you have specific heart conditions or concerns, it's best to consult with your healthcare provider. They can provide personalized advice based on your health status and medical history.  In summary, caffeine can be a part of a heart-healthy diet if consumed in moderation and as part of an overall balanced diet. |
| **GPT4o Answer 2** | Yes, you can still drink caffeine on a heart-healthy diet, but it's important to consume it in moderation. Here are some guidelines to help you incorporate caffeine into your heart-healthy lifestyle:  1. Moderation is Key: Limit your caffeine intake to moderate amounts. This typically means up to 400 milligrams of caffeine per day, which is roughly equivalent to four 8-ounce cups of brewed coffee.  2. Monitor Your Response: Pay attention to how your body reacts to caffeine. Some people are more sensitive to its effects, which can include increased heart rate, blood pressure, and anxiety.  3. Choose Your Sources Wisely: Opt for healthier sources of caffeine. Black coffee and tea are generally good choices. Avoid sugary caffeinated beverages like energy drinks and sodas, which can contribute to weight gain and other heart-related issues.  4. Watch the Additives: Be mindful of what you add to your caffeinated drinks. Excessive sugar, cream, and flavored syrups can negate the benefits of your heart-healthy diet.  5. Time Your Caffeine Intake: Try to avoid caffeine later in the day as it can interfere with your sleep. Poor sleep can negatively affect heart health.  6. Consult Your Doctor: If you have specific heart conditions or are on certain medications, it’s a good idea to talk to your doctor about your caffeine consumption to ensure it’s safe for you.  By following these guidelines, you can enjoy caffeine without compromising your heart health. |
| **GPT4o Answer 3** | Yes, you can still drink caffeine on a heart-healthy diet, but it’s important to do so in moderation. Here are some guidelines to help you enjoy caffeine while maintaining heart health:  1. Moderate Intake: Most health experts suggest that moderate caffeine intake, about 200-300 milligrams per day (roughly 2-3 cups of coffee), is generally safe for most people.  2. Choose Healthy Sources: Opt for healthier caffeine sources like plain coffee or tea rather than sugary or high-fat caffeinated drinks like specialty coffees with added syrups, whipped cream, or energy drinks.  3. Monitor Blood Pressure: Caffeine can temporarily raise blood pressure, so if you have hypertension or other cardiovascular conditions, monitor your blood pressure and consult with your healthcare provider about your caffeine intake.  4. Consider Your Overall Diet: A heart-healthy diet emphasizes fruits, vegetables, whole grains, lean proteins, and healthy fats. Make sure your caffeine consumption doesn't replace these essential components of your diet.  5. Stay Hydrated: Caffeine can have a diuretic effect, so ensure you drink enough water throughout the day to stay hydrated.  6. Limit Late-Day Consumption: To avoid sleep disturbances, try to limit your caffeine intake later in the day, as poor sleep can negatively impact heart health.  Always consult with your healthcare provider to tailor these recommendations to your specific health needs and conditions. |
| **Llama 3 Answer 1** | The eternal question: can I still have my daily cup (or two, or three) of coffee on a heart-healthy diet?  The good news is that moderate caffeine consumption is likely safe for most adults on a heart-healthy diet. However, it's essential to understand the nuances and potential interactions to make informed choices.  What is moderate caffeine consumption?  The American Heart Association (AHA) defines moderate caffeine consumption as up to 300-400 milligrams (mg) per day, which is equivalent to:  * 3-4 cups of brewed coffee (10 oz each)  * 10 cans of cola (12 oz each)  * 2-3 cups of tea (8 oz each)  How does caffeine affect heart health?  Caffeine can have both positive and negative effects on heart health:  Positive effects:  * Caffeine can improve blood flow and boost cardiovascular function.  * Moderate caffeine consumption may lower the risk of stroke, type 2 diabetes, and certain types of cancer.  Negative effects:  * High caffeine intake (more than 400 mg per day) can increase heart rate, blood pressure, and cardiac output, which may exacerbate existing heart conditions.  * Caffeine can interact with certain medications, such as blood thinners, and exacerbate arrhythmias (abnormal heart rhythms).  * Excessive caffeine consumption may lead to dehydration, which can negatively impact heart health.  Special considerations for heart health  If you have any of the following conditions, you may need to limit or avoid caffeine altogether:  * High blood pressure: Caffeine can increase blood pressure, so it's essential to monitor your blood pressure and adjust your caffeine intake accordingly.  * Heart failure: Caffeine can increase heart rate and cardiac output, which may worsen heart failure symptoms.  * Arrhythmias: Caffeine can trigger or exacerbate abnormal heart rhythms.  * Sleep disorders: Caffeine can disrupt sleep patterns, which is essential for overall heart health.  Tips for heart-healthy caffeine consumption  1. Start with a low dose: If you're new to caffeine or haven't consumed it in a while, start with a small amount (e.g., 1/2 cup of coffee) to assess your sensitivity.  2. Monitor your body's response: Pay attention to how caffeine affects your heart rate, blood pressure, and overall well-being.  3. Avoid mixing with other stimulants: Don't consume caffeine with other stimulants, such as energy drinks, medications, or certain supplements.  4. Choose heart-healthy coffee options: Opt for coffee with minimal added sugars, creamers, and syrups, which can increase calorie and sugar intake.  5. Stay hydrated: Drink plenty of water throughout the day to avoid dehydration, which can exacerbate caffeine's negative effects.  In summary, moderate caffeine consumption is likely safe for most adults on a heart-healthy diet. However, it's crucial to be aware of your individual sensitivity, potential interactions, and special considerations for heart health. Always consult with your healthcare provider if you have concerns about caffeine consumption and your heart health. |
| **Llama 3 Answer 2** | Caffeine! While a heart-healthy diet is essential, it's possible to consume caffeine in moderation as part of a balanced diet. Here's what you need to know:  The Good:  1. Moderate caffeine consumption is generally safe: For most adults, moderate caffeine consumption (up to 300-400 milligrams per day) is not associated with an increased risk of heart disease, high blood pressure, or stroke.  2. Caffeine can have benefits: Caffeine may improve exercise performance, boost mental alertness, and even have antioxidant properties.  The Not-So-Good:  1. Excessive caffeine consumption: Consuming more than 400 milligrams of caffeine per day (about 4 cups of brewed coffee) may lead to:  * Increased heart rate and blood pressure  * Insomnia and disrupted sleep patterns  * Anxiety, jitteriness, and irritability  * Dehydration and electrolyte imbalance  2. Individual sensitivity: Some people may be more sensitive to caffeine's effects, which can impact heart health. This includes:  * Children and adolescents  * Pregnant or breastfeeding women  * People with pre-existing heart conditions, high blood pressure, or anxiety disorders  * Older adults or those with sleep disorders  Tips for Heart-Healthy Caffeine Consumption:  1. Know your limits: Stick to moderate caffeine intake (up to 300-400 milligrams per day).  2. Monitor your body: Pay attention to how caffeine affects you. If you experience negative side effects, consider reducing or avoiding caffeine.  3. Choose caffeine sources wisely: Opt for coffee, tea, or dark chocolate, which contain antioxidants and other beneficial compounds. Avoid energy drinks, which often contain high amounts of caffeine and added sugars.  4. Balance with a healthy diet: Make sure your overall diet is rich in fruits, vegetables, whole grains, lean proteins, and healthy fats to counterbalance any potential negative effects of caffeine.  5. Stay hydrated: Drink plenty of water to compensate for caffeine's diuretic effects.  In summary, moderate caffeine consumption is generally safe as part of a heart-healthy diet. However, be mindful of your individual sensitivity, and adjust your caffeine intake accordingly. |
| **Llama 3 Answer 3** | While a heart-healthy diet focuses on whole, nutrient-dense foods, moderate caffeine consumption can be a part of it. Here's what you need to know:  The American Heart Association's (AHA) stance:  The AHA suggests that moderate caffeine consumption, defined as up to 300-400 milligrams (mg) per day, is unlikely to pose a significant risk for most healthy adults. This amount is equivalent to:  * 3-4 cups of brewed coffee (8 oz each)  * 10 cans of cola (12 oz each)  * 2 energy drinks (8 oz each)  Who may need to limit or avoid caffeine:  However, certain individuals may need to be more cautious or avoid caffeine altogether:  * Pregnant women: Limit caffeine to 200 mg or less per day, as high caffeine intake may increase the risk of miscarriage and growth restriction.  * People with high blood pressure: Caffeine can increase blood pressure and heart rate, so those with hypertension may need to limit or avoid it.  * Heart disease patients: If you have heart disease, your doctor may recommend limiting or avoiding caffeine, as it can increase heart rate and blood pressure.  * Sensitivity to caffeine: Some people may experience adverse effects, such as jitteriness, anxiety, or insomnia, even at moderate levels of caffeine consumption.  Tips for heart-healthy caffeine consumption:  If you choose to consume caffeine, follow these guidelines:  1. Be mindful of hidden sources: Caffeine is not only found in coffee, tea, and energy drinks but also in chocolate, some medications, and certain foods like guarana or yerba mate.  2. Monitor your intake: Keep track of your daily caffeine consumption to ensure you're staying within the moderate range.  3. Choose brewed coffee over unfiltered coffee: Unfiltered coffee, like French press or espresso, can contain higher levels of cholesterol-raising compounds like cafestol and kahweol.  4. Avoid adding large amounts of sugar, cream, or syrup: These can greatly increase calorie and sugar intake, which can negatively impact heart health.  5. Pay attention to your body: If you experience any adverse effects, consider reducing your caffeine intake or switching to decaf.  In summary, moderate caffeine consumption can be a part of a heart-healthy diet, but it's essential to be mindful of your individual tolerance, potential interactions with medications, and any underlying health conditions. Always consult with your healthcare provider or a registered dietitian for personalized guidance. |
| **LLaMA3+RAG model Answer 1** | According to the American Heart Association, moderate coffee consumption, defined as 4 to 5 cups per day, appears to be safe for the heart. This is supported by the Food and Drug Administration's guidelines, which suggest that healthy adults can consume up to 4 to 5 cups of coffee without harm. However, it's essential to note that individual tolerance to caffeine can vary greatly, and some people may be more sensitive to its effects.  Certain groups, such as pregnant and lactating women, should limit their caffeine intake to 2 to 3 cups per day. Children and adolescents, on the other hand, are advised to avoid caffeine and other stimulants altogether, as recommended by the American Academy of Pediatrics.  It's also important to consider the potential side effects of caffeine, including increased heart rate, blood pressure, and urination, which can lead to dehydration. Additionally, caffeine can interact with certain medications and exacerbate underlying health conditions.  If you're concerned about the impact of caffeine on your heart health, it's always best to consult with your healthcare professional. They can provide personalized guidance based on your unique health profile and medical history.  In summary, moderate caffeine consumption, as part of a balanced diet, is unlikely to pose a significant risk to heart health for most adults. However, it's crucial to be mindful of individual tolerance, potential interactions, and specific health conditions that may warrant caution or avoidance.  Sources:  American Heart Association. (2024). Caffeine and Heart Disease. Retrieved from <https://www.heart.org/en/healthy-living/healthy-eating/eat-smart/nutrition-basics/caffeine-and-heart-disease>  Food and Drug Administration. (2024). Spilling the Beans: How Much Caffeine is Too Much? Retrieved from <https://www.fda.gov/consumers/consumer-updates/spilling-beans-how-much-caffeine-too-much>  American Academy of Pediatrics. (2018). HealthyChildren.org: Caffeine and Kids. Retrieved from <https://www.healthychildren.org/English/healthy-living/nutrition/Pages/Caffeine-and-Kids.aspx> |
| **LLaMA3+RAG model Answer 2** | According to the American Heart Association, drinking coffee in moderation appears to be safe for the heart. In fact, the Food and Drug Administration suggests that healthy adults can drink 4 to 5 cups of coffee without harm. However, it's essential to note that individual tolerance to caffeine can vary greatly. Some people may be more sensitive to its effects, particularly those with certain health conditions or taking specific medications.  Pregnant and lactating women, for instance, should limit their caffeine intake to 2 to 3 cups per day. Children and adolescents, on the other hand, should avoid caffeine and other stimulants altogether, as advised by the American Academy of Pediatrics.  It's also important to be mindful of the potential negative effects of caffeine, such as increased heart rate, blood pressure, and urinary excretion, which can lead to dehydration. Additionally, caffeine can exacerbate certain health conditions, such as anxiety, insomnia, and high blood pressure.  If you're concerned about your caffeine intake or have questions about its impact on your heart health, it's always best to consult with your healthcare professional. They can provide personalized guidance based on your individual health needs and circumstances.  In summary, moderate caffeine consumption, defined as 4 to 5 cups of coffee per day, is generally considered safe for most adults. However, it's crucial to be aware of your individual sensitivity to caffeine, potential negative effects, and specific health conditions that may warrant caution or avoidance.  Sources:  American Heart Association. (2024). Caffeine and Heart Disease. Retrieved from <https://www.heart.org/en/healthy-living/healthy-eating/eat-smart/nutrition-basics/caffeine-and-heart-disease>  Food and Drug Administration. (2024). Spilling the Beans: How Much Caffeine is Too Much? Retrieved from <https://www.fda.gov/consumers/consumer-updates/spilling-beans-how-much-caffeine-too-much>  American Academy of Pediatrics. (2018). HealthyChildren.org: Caffeine and Kids. Retrieved from <https://www.healthychildren.org/English/healthy-living/nutrition/Pages/Caffeine-and-Kids.aspx> |
| **LLaMA3+RAG model Answer 3** | According to the American Heart Association (AHA), moderate coffee consumption, defined as 4 to 5 cups per day, appears to be safe for the heart. This is based on guidelines from the Food and Drug Administration (FDA). However, it's essential to note that individual tolerance to caffeine can vary greatly, and certain groups should limit or avoid caffeine altogether.  Pregnant and lactating women, for instance, should restrict their daily caffeine intake to 2 to 3 cups. The American Academy of Pediatrics advises that children and adolescents avoid caffeine and other stimulants altogether.  Additionally, people with certain health conditions or taking specific medications may need to be more cautious with their caffeine consumption. It's crucial to consult with a healthcare professional to determine the appropriate amount of caffeine for your individual situation.  It's also important to be mindful of the potential negative effects of caffeine, such as dehydration, anxiety, and fatigue, particularly if you're sensitive to its effects. Caffeine withdrawal, although not dangerous, can be unpleasant and may lead to headaches, anxiety, fatigue, and drowsiness.  To make informed choices, consider the following:  Be aware of the amount of caffeine in your beverages, including coffee, tea, soft drinks, chocolate, and energy drinks.  Monitor your body's response to caffeine and adjust your intake accordingly.  Don't forget to stay hydrated by drinking plenty of water throughout the day.  If you experience any adverse effects or have concerns, consult with your healthcare professional.  In summary, moderate caffeine consumption, defined as 4 to 5 cups of coffee per day, is generally considered safe for the heart. However, individual tolerance and certain health conditions may require adjustments to this recommendation. Always consult with a healthcare professional to determine the appropriate amount of caffeine for your unique situation.  Sources:  American Heart Association. (2024). Caffeine and Heart Disease. Retrieved from <https://www.heart.org/en/healthy-living/healthy-eating/eat-smart/nutrition-basics/caffeine-and-heart-disease>  Food and Drug Administration. (2024). Spilling the Beans: How Much Caffeine is Too Much? Retrieved from <https://www.fda.gov/consumers/consumer-updates/spilling-beans-how-much-caffeine-too-much>  American Academy of Pediatrics. (2024). Caffeine and Kids: What's the Buzz? Retrieved from <https://pediatrics.aappublications.org/content/142/2/e20182148> |
| **Question 28** | **Can I still drink soda on a heart healthy diet?** |
| **Perplexity Answer 1** | No, it's generally not recommended to drink soda, including diet soda, on a heart-healthy diet. Soda consumption has been linked to an increased risk of heart disease and other health issues[1][3].  Sugary sodas are high in added sugars and calories, which can lead to weight gain, high triglycerides, and low HDL (good) cholesterol levels - all risk factors for heart disease[3]. Even diet sodas have been associated with an increased risk of metabolic syndrome, diabetes, and cardiovascular events like heart attack and stroke[1].  Instead of soda, it's better to choose water, unsweetened teas, coffee, or other unsweetened beverages for a heart-healthy diet[2][4]. If you crave something fizzy, try sparkling water with a splash of 100% fruit juice or sliced fruit for flavor[4].  While an occasional soda may be okay for some people, regularly drinking soda, whether sugary or diet, is not recommended for optimal heart health. Making the effort to cut back or eliminate soda from your diet can have positive impacts on your cholesterol levels, blood pressure, and overall cardiovascular risk[1][3].  Citations:  [1] https://www.health.harvard.edu/blog/is-there-a-link-between-diet-soda-and-heart-disease-201202214296  [2] https://austinheart.com/blog/entry/the-best-and-worst-drinks-for-heart-health  [3] https://www.heart.org/en/news/2020/02/26/sugary-drinks-negatively-impact-these-two-risk-factors-for-heart-disease  [4] https://www.heartfoundation.org.au/healthy-living/healthy-eating/heart-healthy-drinks  [5] https://www.health.harvard.edu/heart-health/early-signs-of-heart-disease-in-people-who-drink-sugary-soda |
| **Perplexity Answer 2** | No, it's generally not recommended to drink soda, including diet soda, on a heart-healthy diet. Soda consumption has been linked to an increased risk of heart disease and other health issues[1][3].  Sugary sodas are high in added sugars and calories, which can lead to weight gain, high triglycerides, and low HDL (good) cholesterol levels - all risk factors for heart disease[3]. Even diet sodas have been associated with an increased risk of metabolic syndrome, diabetes, and cardiovascular events like heart attack and stroke[1].  Instead of soda, it's better to choose water, unsweetened teas, coffee, or other unsweetened beverages for a heart-healthy diet[2][4]. If you crave something fizzy, try sparkling water with a splash of 100% fruit juice or sliced fruit for flavor[4].  While an occasional soda may be okay for some people, regularly drinking soda, whether sugary or diet, is not recommended for optimal heart health. Making the effort to cut back or eliminate soda from your diet can have positive impacts on your cholesterol levels, blood pressure, and overall cardiovascular risk[1][3].  Citations:  [1] https://www.health.harvard.edu/blog/is-there-a-link-between-diet-soda-and-heart-disease-201202214296  [2] https://austinheart.com/blog/entry/the-best-and-worst-drinks-for-heart-health  [3] https://www.heart.org/en/news/2020/02/26/sugary-drinks-negatively-impact-these-two-risk-factors-for-heart-disease  [4] https://www.heartfoundation.org.au/healthy-living/healthy-eating/heart-healthy-drinks  [5] https://www.health.harvard.edu/heart-health/early-signs-of-heart-disease-in-people-who-drink-sugary-sodas |
| **Perplexity Answer 3** | No, it's generally not recommended to drink soda, including diet soda, on a heart-healthy diet. Soda consumption has been linked to an increased risk of heart disease and other health issues[1][3].  Sugary sodas are high in added sugars and calories, which can lead to weight gain, high triglycerides, and low HDL (good) cholesterol levels - all risk factors for heart disease[3]. Even diet sodas have been associated with an increased risk of metabolic syndrome, diabetes, and cardiovascular events like heart attack and stroke[1].  Instead of soda, it's better to choose water, unsweetened teas, coffee, or other unsweetened beverages for a heart-healthy diet[2][4]. If you crave something fizzy, try sparkling water with a splash of 100% fruit juice or sliced fruit for flavor[4].  While an occasional soda may be okay for some people, regularly drinking soda, whether sugary or diet, is not recommended for optimal heart health. Making the effort to cut back or eliminate soda from your diet can have positive impacts on your cholesterol levels, blood pressure, and overall cardiovascular risk[1][3].  Citations:  [1] https://www.health.harvard.edu/blog/is-there-a-link-between-diet-soda-and-heart-disease-201202214296  [2] https://austinheart.com/blog/entry/the-best-and-worst-drinks-for-heart-health  [3] https://www.heart.org/en/news/2020/02/26/sugary-drinks-negatively-impact-these-two-risk-factors-for-heart-disease  [4] https://www.heartfoundation.org.au/healthy-living/healthy-eating/heart-healthy-drinks  [5] https://www.health.harvard.edu/heart-health/early-signs-of-heart-disease-in-people-who-drink-sugary-sodas |
| **GPT4o Answer 1** | Drinking soda in moderation can be part of a heart-healthy diet, but there are some important considerations to keep in mind:  1. Sugar Content: Regular soda is high in added sugars, which can contribute to weight gain, obesity, and an increased risk of heart disease. Excessive sugar intake can also lead to high blood pressure and inflammation.  2. Artificial Sweeteners: Diet sodas contain artificial sweeteners instead of sugar. While they have fewer calories, the long-term effects of artificial sweeteners on heart health are still being studied. Some research suggests they might not be as benign as once thought.  3. Caffeine: Many sodas contain caffeine, which in moderate amounts is generally safe for most people. However, excessive caffeine intake can lead to increased heart rate and blood pressure.  4. Sodium Content: Some sodas contain sodium, which can contribute to high blood pressure, especially if consumed in large quantities.  5. Empty Calories: Sodas provide little to no nutritional value, meaning they can displace more nutritious beverages and foods from your diet.  For a heart-healthy diet, consider these alternatives:  - Water: The best option for staying hydrated without any added sugars or calories.  - Sparkling Water: Offers the fizz of soda without the sugar. Look for those without added sugars or artificial sweeteners.  - Herbal Teas: Many are caffeine-free and come in a variety of flavors.  - Diluted Fruit Juices: Mix a small amount of 100% fruit juice with water for a flavorful drink with fewer calories and sugar.  If you do choose to drink soda, aim to do so occasionally and in moderation. Opt for smaller portions and avoid making it a regular part of your diet. |
| **GPT4o Answer 2** | Drinking soda in moderation can be part of a heart-healthy diet, but it is important to be mindful of the following considerations:  1. Sugar Content: Regular sodas are high in added sugars, which can contribute to weight gain, diabetes, and heart disease. It's best to limit or avoid these beverages.  2. Artificial Sweeteners: Diet sodas often contain artificial sweeteners. While they are lower in calories, some studies suggest a potential link between artificial sweeteners and an increased risk of metabolic syndrome and cardiovascular issues. Moderation is key.  3. Caffeine: Some sodas contain caffeine, which can affect your heart health if consumed in large quantities. Monitor your overall caffeine intake from all sources.  4. Empty Calories: Sodas provide little to no nutritional value. It's better to choose beverages that offer health benefits, such as water, herbal teas, or beverages fortified with vitamins and minerals.  5. Sodium: Some sodas contain sodium, which can contribute to high blood pressure, a risk factor for heart disease.  Healthier Alternatives  - Water: The best choice for hydration. Add a slice of lemon, lime, or cucumber for flavor.  - Sparkling Water: Offers the fizz without the added sugars or artificial sweeteners. Look for options without added sodium.  - Herbal Teas: Naturally free of calories and can provide additional health benefits.  - Infused Water: Water infused with fruits, herbs, or vegetables can be a flavorful alternative to soda.  Recommendations  - If you enjoy soda, try to limit your intake to an occasional treat rather than a daily habit.  - Pay attention to serving sizes and avoid large portions.  - Balance your diet with plenty of fruits, vegetables, whole grains, and lean proteins to support overall heart health.  Consulting with a healthcare provider or a nutritionist can provide personalized advice based on your specific health needs and dietary preferences. |
| **GPT4o Answer 3** | While you can technically drink soda on a heart-healthy diet, it's generally recommended to limit or avoid it for several reasons:  1. High Sugar Content: Regular sodas are high in added sugars, which can contribute to weight gain, increase blood glucose levels, and lead to insulin resistance. These factors are risk factors for heart disease.  2. Empty Calories: Sodas provide a lot of calories without any nutritional benefits. Consuming high-calorie drinks can lead to obesity, which is a major risk factor for heart disease.  3. Blood Pressure: The high sugar content in sodas can contribute to high blood pressure, another risk factor for heart disease.  4. Artificial Sweeteners: Diet sodas, which use artificial sweeteners instead of sugar, may seem like a better alternative. However, some studies suggest that artificial sweeteners might still negatively impact heart health by affecting metabolism and leading to weight gain.  5. Caffeine and Additives: Many sodas contain caffeine and other additives that can have various effects on heart health, including increasing heart rate and blood pressure.  Alternatives to Soda  - Water: The best choice for hydration. You can add a splash of lemon or lime for flavor.  - Sparkling Water: A good alternative if you crave the carbonation of soda. Choose unsweetened varieties.  - Herbal Teas: Naturally free of caffeine and sugar.  - Infused Water: Water with added fruits, vegetables, or herbs for natural flavor.  Moderation  If you do choose to drink soda, it’s best to do so in moderation. Opting for smaller portions and limiting it to occasional treats rather than a daily habit can help minimize its impact on your heart health. Always read the labels to be aware of the sugar and calorie content.  Consulting a Healthcare Professional  It's important to consult with a healthcare provider or a dietitian to tailor dietary choices to your specific health needs, especially if you have preexisting heart conditions or other health concerns |
| **Llama 3 Answer 1** | The sweet temptation of soda! While it may be difficult to give up, the answer is a resounding "no" - or at least, a significant reduction is recommended. Here's why:  Soda and heart health  Regular consumption of soda, particularly sugar-sweetened beverages, is linked to an increased risk of heart disease, stroke, and other cardiovascular conditions. This is because soda contains:  1. High amounts of added sugars: Consuming high amounts of added sugars, like those found in soda, can lead to weight gain, insulin resistance, and increased triglycerides, all of which are risk factors for heart disease.  2. Artificial sweeteners: While artificially sweetened sodas may seem like a healthier alternative, some research suggests that they can still contribute to weight gain, metabolic problems, and an increased risk of heart disease.  3. Sodium: Soda can be high in sodium, which can increase blood pressure and contribute to heart disease.  4. Acidity: The acidity of soda can erode tooth enamel and lead to digestive issues, which can indirectly impact heart health.  Guidelines for heart healthy drinking  To reduce your risk of heart disease, the American Heart Association (AHA) recommends the following:  1. Limit sugary drinks: Drink no more than 100 calories (about 6 ounces) of sugary drinks per day. For soda, this means limiting yourself to no more than 1/2 can (12 ounces) per day.  2. Choose unsweetened beverages: Opt for water, unsweetened tea, coffee, and low-fat milk instead of soda.  3. Be mindful of artificial sweeteners: While artificially sweetened drinks may be a better alternative to sugary soda, it's still important to consume them in moderation.  Tips for reducing soda consumption  If you're struggling to give up soda, try these tips:  1. Gradual reduction: Gradually reduce your soda intake over time to minimize withdrawal symptoms.  2. Find alternatives: Try infused water, seltzer with a squeeze of fresh fruit, or unsweetened tea as a replacement.  3. Identify triggers: Be aware of situations that make you crave soda, such as eating out or watching movies, and plan ahead with healthier options.  4. Get support: Share your goals with friends and family, and consider enlisting a "soda-free buddy" to support you.  Remember, a heart healthy diet is all about balance and moderation. If you do choose to drink soda, make it an occasional treat and not a daily habit. |
| **Llama 3 Answer 2** | The sweet temptation of soda! While it's understandable that you might want to indulge in soda occasionally, the answer is a resounding "no" - or at least, "rarely" - when it comes to a heart-healthy diet.  Here's why:  1. Sugar content: Soda is loaded with added sugars, which are a major contributor to heart disease risk factors like high blood pressure, high triglycerides, and low HDL (good) cholesterol. The American Heart Association recommends that women consume no more than 25 grams (6 teaspoons) and men no more than 36 grams (9 teaspoons) of added sugars per day. A single 12-ounce can of soda often exceeds these daily limits.  2. Lack of essential nutrients: Soda is essentially empty calories, providing no vitamins, minerals, or fiber. It displaces more nutritious beverages, like water, milk, or 100% fruit juice, which are essential for a heart-healthy diet.  3. Impact on blood pressure: Regular soda consumption has been linked to higher blood pressure, which is a significant risk factor for heart disease, stroke, and kidney disease.  4. Contribution to weight gain: Soda is high in calories and low in satiety, leading to overconsumption and weight gain. Excess weight increases the risk of heart disease, type 2 diabetes, and certain types of cancer.  5. Acidity and gut health: Soda's acidity can erode tooth enamel and alter the gut microbiome, leading to digestive issues and potentially even influencing blood pressure and cardiovascular health.  If you're craving a fizzy drink, consider these alternatives:  1. Water: The ultimate thirst quencher and calorie-free!  2. Seltzer water: Add a squeeze of fresh lime or lemon juice for flavor.  3. Unsweetened tea or coffee: Rich in antioxidants and without added sugars.  4. Infused water: Try adding slices of cucumber, strawberries, or oranges to a pitcher of water for a refreshing twist.  5. Low-fat milk or 100% fruit juice: In moderation, these can be part of a heart-healthy diet.  If you still want to indulge in soda occasionally, consider these tips:  1. Limit frequency: Allow yourself a soda only on special occasions or as a rare treat.  2. Choose a smaller size: Opt for a smaller serving size, like a 7-ounce can or a mini bottle.  3. Pair with a healthy meal: Balance out the negative effects of soda by pairing it with a nutritious meal or snack.  Remember, a heart-healthy diet is all about balance, variety, and moderation. Enjoy your beverages wisely, and prioritize whole, nutrient-dense foods to support your overall well-being. |
| **Llama 3 Answer 3** | The sweet temptation of soda! While it's understandable to want to indulge in soda from time to time, it's generally not recommended as part of a heart-healthy diet. Here's why:  Why soda is not heart-healthy:  1. High sugar content: Soda is loaded with added sugars, which can lead to weight gain, insulin resistance, and an increased risk of type 2 diabetes, all of which are risk factors for heart disease.  2. Lack of essential nutrients: Soda is essentially empty calories, providing no vitamins, minerals, or fiber, while displacing more nutritious beverages and foods.  3. Increased blood pressure: Regular soda consumption has been linked to higher blood pressure, a major risk factor for heart disease, stroke, and kidney disease.  4. Inflammation and oxidative stress: The high sugar and artificial sweetener content in soda can lead to chronic inflammation and oxidative stress, which can damage blood vessels and increase cardiovascular risk.  5. Link to heart disease and stroke: Studies have consistently shown that frequent soda consumption is associated with a higher risk of heart disease, stroke, and cardiovascular mortality.  What about diet soda?  While diet soda may seem like a healthier alternative, research suggests that it may not be entirely harmless. Artificial sweeteners, such as aspartame and sucralose, have been linked to:  1. Metabolic changes: Diet soda consumption has been associated with changes in gut bacteria, which can affect glucose metabolism and insulin sensitivity.  2. Cardiovascular risk: Some studies have found that diet soda consumption is linked to an increased risk of heart disease, stroke, and cardiovascular mortality, although the evidence is not as strong as it is for regular soda.  So, can you still drink soda on a heart-healthy diet?  In moderation, perhaps. If you're craving soda, consider the following:  1. Limit frequency and amount: If you can't resist soda, try to limit your consumption to special occasions or weekends, and stick to a single serving (12 oz).  2. Choose a lower-sugar option: Opt for a soda with less sugar (e.g., ginger ale or root beer) or try a seltzer water with a squeeze of fresh fruit for a healthier alternative.  3. Make healthier swaps: Replace soda with water, unsweetened tea, coffee, or low-fat milk to quench your thirst and provide essential nutrients.  The best beverage choices for a heart-healthy diet:  1. Water: The ultimate thirst quencher, water is calorie-free and essential for overall health.  2. Unsweetened tea or coffee: Rich in antioxidants and flavonoids, these beverages may help lower blood pressure and improve cardiovascular health.  3. Low-fat milk or fortified plant-based milk: These provide calcium, protein, and other essential nutrients for heart health.  4. 100% fruit and vegetable juices: In moderation! These can provide essential vitamins, minerals, and antioxidants, but be mindful of sugar content.  Remember, a heart-healthy diet emphasizes whole, nutrient-dense foods, and beverages should support, not detract from, your overall health goals. |
| **LLaMA3+RAG model Answer 1** | Soda is a significant source of added sugar, which is a major contributor to high blood pressure, heart disease, and stroke. The American Heart Association recommends limiting items with added sugar, including sugar-sweetened beverages like soda. In fact, the AHA suggests that women consume no more than 25 grams (6 teaspoons) of added sugar per day, while men should limit their intake to no more than 36 grams (9 teaspoons) per day. A single 12-ounce can of soda can exceed these daily limits!  Furthermore, soda is often high in sodium, which is another major contributor to high blood pressure. The AHA recommends reducing sodium intake to less than 1,500 milligrams per day, and soda can quickly add up to a significant portion of that daily limit.  In addition to the negative impacts of added sugar and sodium, soda is also a source of empty calories, providing no nutritional value while displacing more nutritious beverages like water, milk, or 100% fruit juice.  So, what can you drink instead? The AHA recommends drinking plenty of water, as well as fat-free or low-fat milk, 100% fruit and vegetable juices, and tea or coffee without added sugars.  In summary, while it may be tempting to indulge in soda, it is not a heart-healthy choice. Instead, opt for beverages that are low in added sugar, sodium, and saturated fat, and high in nutritional value.  Sources:  American Heart Association. (2024). Five Simple Steps to Control Your Blood Pressure. Retrieved from <https://www.heart.org/en/health-topics/high-blood-pressure/the-facts-about-high-blood-pressure/five-simple-steps-to-control-your-blood-pressure>  American Heart Association. (2024). Managing Blood Pressure with a Heart-Healthy Diet. Retrieved from <https://www.heart.org/en/health-topics/high-blood-pressure/changes-you-can-make-to-manage-high-blood-pressure/managing-blood-pressure-with-a-heart-healthy-diet>  American Heart Association. (2017). Added Sugars and Health. Retrieved from <https://www.heart.org/en/healthy-living/healthy-eating/eat-smart/nutrition-basics/added-sugars-and-health> |
| **LLaMA3+RAG model Answer 2** | Soda is a significant source of added sugar, which is explicitly listed as an item to limit in a heart-healthy diet. Consuming high amounts of added sugar, like those found in soda, can lead to a range of negative health effects, including:  1. Increased blood pressure: High sugar intake has been linked to higher blood pressure, which is a major risk factor for heart disease.  2. Weight gain: Soda is high in empty calories, contributing to weight gain and obesity, which can further exacerbate blood pressure and heart health issues.  3. Insulin resistance: Regularly consuming high-sugar drinks like soda can lead to insulin resistance, a precursor to type 2 diabetes, which is a significant risk factor for heart disease.  Furthermore, soda is often high in sodium, which is another nutrient to limit in a heart-healthy diet. Excessive sodium consumption can lead to high blood pressure, heart disease, and stroke.  The American Heart Association recommends limiting items with added sugar, such as sugar-sweetened beverages like soda, to promote heart health. Instead, opt for water, unsweetened tea, or other low-calorie, nutrient-rich beverages.  In summary, soda is not a heart-healthy choice, and it's best to avoid it or severely limit its consumption to maintain a healthy diet and reduce the risk of heart disease.  Sources:  1. American Heart Association. (2024). Five Simple Steps to Control Your Blood Pressure. Retrieved from <https://www.heart.org/en/health-topics/high-blood-pressure/the-facts-about-high-blood-pressure/five-simple-steps-to-control-your-blood-pressure>  2. American Heart Association. (2024). Managing Blood Pressure with a Heart-Healthy Diet. Retrieved from <https://www.heart.org/en/health-topics/high-blood-pressure/changes-you-can-make-to-manage-high-blood-pressure/managing-blood-pressure-with-a-heart-healthy-diet>  3. Johnson, R. K., Appel, L. J., Brands, M. W., Howard, B. V., Lefevre, M., Lustig, R. H., ... & Wylie-Rosett, J. (2009). Dietary sugars intake and cardiovascular health: a scientific statement from the American Heart Association. Circulation, 120(11), 1011-1020. doi: 10.1161/CIRCULATIONAHA.109.192627 |
| **LLaMA3+RAG model Answer 3** | Soda is a significant source of added sugar, which is a major contributor to heart disease. Consuming high amounts of added sugar, like those found in soda, can lead to increased calorie intake, weight gain, and inflammation, all of which can raise blood pressure and increase the risk of heart disease (1).  Furthermore, soda is often high in sodium, which is a major concern for heart health. The American Heart Association recommends limiting sodium intake to less than 1,500 milligrams per day, and soda can quickly exceed that limit. Excessive sodium consumption can lead to high blood pressure, heart failure, and stroke (2).  In addition, soda is typically made with refined sugars and artificial sweeteners, which can be detrimental to overall health. Refined sugars can cause a rapid spike in blood sugar levels, leading to insulin resistance and an increased risk of type 2 diabetes, another major risk factor for heart disease (3).  The American Heart Association's guidelines for a heart-healthy diet emphasize the importance of limiting or avoiding foods and beverages that are high in added sugars, saturated fats, and sodium. Soda fails to meet these criteria, making it a poor choice for those seeking to maintain a heart-healthy diet.  Instead, opt for water, unsweetened tea, or other low-calorie, nutrient-rich beverages that can help you stay hydrated and support overall health.  Sources:  1. American Heart Association. (2017). Added Sugars and Health. Retrieved from <https://www.heart.org/en/healthy-living/healthy-eating/eat-smart/nutrition-basics/added-sugars-and-health>  2. American Heart Association. (2020). Changes You Can Make to Manage High Blood Pressure. Retrieved from <https://www.heart.org/en/health-topics/high-blood-pressure/changes-you-can-make-to-manage-high-blood-pressure/managing-blood-pressure-with-a-heart-healthy-diet>  3. American Diabetes Association. (2020). Sugar and Desserts. Retrieved from <https://www.diabetes.org/healthy-living/recipes-nutrition/eating-diabetes/sugar-and-desserts> |
| **Question 29** | **Can I eat nuts on a heart healthy diet?** |
| **Perplexity Answer 1** | Yes, nuts can be a great addition to a heart-healthy diet. Almost all nuts are packed with nutrients that are beneficial for heart health, such as vitamin E, omega-3 fatty acids, fiber, and unsaturated fats[9].  Eating a daily handful (about 1.5 ounces or 30g) of unsalted nuts can reduce your risk of heart disease by up to 27%[10]. Nuts can help lower cholesterol levels, reduce inflammation, improve blood vessel health, and lower the risk of heart disease[9].  Some of the most heart-healthy nuts include:  - Almonds - can help reduce blood sugar, lower cholesterol, and reduce inflammation[9]  - Pecans - contain healthy fats that may lower bad cholesterol and raise good cholesterol[9]  - Macadamia nuts - can positively impact cholesterol levels and reduce inflammation[9]  - Walnuts - considered the ultimate nut for heart health, they can help lower cholesterol, reduce inflammation, and lower blood pressure[9]  - Pistachios - eating 2-3 ounces per day can help raise good cholesterol levels and lower blood pressure[9]  - Peanuts (technically legumes) - can reduce the risk of heart disease and type 2 diabetes[9]  - Cashews - can help lower cholesterol levels[9]  The key is to choose unsalted, raw or dry-roasted nuts, as adding salt or sugar diminishes the heart-healthy benefits[9]. Portion control is also important, as nuts are high in calories and can contribute to weight gain if overeaten[9][10].  Citations:  [1] https://www.yelp.com/biz/kansai-oakland-3?adjust_creative=6zagVcKwffYZAA-51mlGNg  [2] https://www.yelp.com/biz/ohgane-korean-bbq-oakland-3?adjust_creative=6zagVcKwffYZAA-51mlGNg  [3] https://www.yelp.com/biz/koja-kitchen-emeryville-2?adjust_creative=6zagVcKwffYZAA-51mlGNg  [4] https://www.yelp.com/biz/koja-kitchen-berkeley-2?adjust_creative=6zagVcKwffYZAA-51mlGNg  [5] https://www.yelp.com/biz/jong-ga-house-oakland?adjust_creative=6zagVcKwffYZAA-51mlGNg  [6] https://www.yelp.com/biz/kirala-restaurant-berkeley-2?adjust_creative=6zagVcKwffYZAA-51mlGNg  [7] https://www.yelp.com/biz/spoon-korean-bistro-x-kawaii-berkeley?adjust_creative=6zagVcKwffYZAA-51mlGNg  [8] https://www.yelp.com/biz/tane-vegan-izakaya-berkeley?adjust_creative=6zagVcKwffYZAA-51mlGNg  [9] https://blog.bonsecours.com/heart/heart-healthy-nuts-list/  [10] https://www.sanitarium.com/au/health-nutrition/nutrition/daily-handful-of-nuts-can-keep-your-heart-healthy/  [11] https://www.hsph.harvard.edu/nutritionsource/nuts-for-the-heart/  [12] https://www.trulygoodfoods.com/2020/06/17/heart-healthy-nuts/  [13] https://www.healthline.com/nutrition/9-healthy-nut |
| **Perplexity Answer 2** | Yes, nuts can be a great addition to a heart-healthy diet. Almost all nuts are packed with nutrients that are beneficial for heart health, such as vitamin E, omega-3 fatty acids, fiber, and unsaturated fats[9].  Eating a daily handful of nuts (about 1.5 ounces or 30g) can reduce your risk of heart disease by up to 27%[10]. Nuts help lower cholesterol levels, reduce inflammation, improve blood vessel health, and lower the risk of heart disease[9].  Some of the most heart-healthy nuts include[9]:  - Almonds - help reduce blood sugar, lower cholesterol, and reduce inflammation  - Pecans - may lower bad cholesterol and raise good cholesterol  - Macadamia nuts - can positively impact cholesterol levels and reduce inflammation  - Walnuts - may help lower cholesterol, inflammation, and blood pressure  - Pistachios - can help raise good cholesterol levels and lower blood pressure  - Peanuts - reduce the risk of heart disease and type 2 diabetes  - Cashews - can help lower cholesterol levels  The key is to choose unsalted, raw or dry-roasted nuts, as adding salt or sugar reduces their heart-healthy benefits[9]. Limit portions to about 1.5 ounces per day, as nuts are high in calories and can contribute to weight gain if over-consumed[9][10].  Citations:  [1] https://www.yelp.com/biz/kansai-oakland-3?adjust_creative=6zagVcKwffYZAA-51mlGNg  [2] https://www.yelp.com/biz/ohgane-korean-bbq-oakland-3?adjust_creative=6zagVcKwffYZAA-51mlGNg  [3] https://www.yelp.com/biz/koja-kitchen-emeryville-2?adjust_creative=6zagVcKwffYZAA-51mlGNg  [4] https://www.yelp.com/biz/koja-kitchen-berkeley-2?adjust_creative=6zagVcKwffYZAA-51mlGNg  [5] https://www.yelp.com/biz/jong-ga-house-oakland?adjust_creative=6zagVcKwffYZAA-51mlGNg  [6] https://www.yelp.com/biz/kirala-restaurant-berkeley-2?adjust_creative=6zagVcKwffYZAA-51mlGNg  [7] https://www.yelp.com/biz/spoon-korean-bistro-x-kawaii-berkeley?adjust_creative=6zagVcKwffYZAA-51mlGNg  [8] https://www.yelp.com/biz/tane-vegan-izakaya-berkeley?adjust_creative=6zagVcKwffYZAA-51mlGNg  [9] https://blog.bonsecours.com/heart/heart-healthy-nuts-list/  [10] https://www.sanitarium.com/au/health-nutrition/nutrition/daily-handful-of-nuts-can-keep-your-heart-healthy/  [11] https://www.hsph.harvard.edu/nutritionsource/nuts-for-the-heart/  [12] https://www.trulygoodfoods.com/2020/06/17/heart-healthy-nuts/  [13] https://www.healthline.com/nutrition/9-healthy-nuts |
| **Perplexity Answer 3** | Yes, nuts can be a great addition to a heart-healthy diet. Almost all nuts are packed with nutrients that are beneficial for heart health, such as vitamin E, omega-3 fatty acids, fiber, and unsaturated fats[9].  Eating a daily handful of nuts (about 1.5 ounces or 30g) can reduce your risk of heart disease by up to 27%[10]. Nuts help lower cholesterol levels, reduce inflammation, improve blood vessel health, and lower the risk of heart disease[9].  Some of the most heart-healthy nuts include[9]:  - Almonds - help reduce blood sugar, lower cholesterol, and reduce inflammation  - Pecans - may lower bad cholesterol and raise good cholesterol  - Macadamia nuts - can positively impact cholesterol levels and reduce inflammation  - Walnuts - may help lower cholesterol, inflammation, and blood pressure  - Pistachios - can help raise good cholesterol levels and lower blood pressure  - Peanuts - reduce the risk of heart disease and type 2 diabetes  - Cashews - can help lower cholesterol levels  The key is to eat nuts in moderation, as they are high in calories. Adults should limit nuts to 4-6 servings (1.5 ounces or 2 tbsp nut butter) per week[9]. Choose unsalted, raw or dry-roasted nuts for the most benefits.  Citations:  [1] https://www.yelp.com/biz/kansai-oakland-3?adjust_creative=6zagVcKwffYZAA-51mlGNg  [2] https://www.yelp.com/biz/ohgane-korean-bbq-oakland-3?adjust_creative=6zagVcKwffYZAA-51mlGNg  [3] https://www.yelp.com/biz/koja-kitchen-emeryville-2?adjust_creative=6zagVcKwffYZAA-51mlGNg  [4] https://www.yelp.com/biz/koja-kitchen-berkeley-2?adjust_creative=6zagVcKwffYZAA-51mlGNg  [5] https://www.yelp.com/biz/jong-ga-house-oakland?adjust_creative=6zagVcKwffYZAA-51mlGNg  [6] https://www.yelp.com/biz/kirala-restaurant-berkeley-2?adjust_creative=6zagVcKwffYZAA-51mlGNg  [7] https://www.yelp.com/biz/spoon-korean-bistro-x-kawaii-berkeley?adjust_creative=6zagVcKwffYZAA-51mlGNg  [8] https://www.yelp.com/biz/tane-vegan-izakaya-berkeley?adjust_creative=6zagVcKwffYZAA-51mlGNg  [9] https://blog.bonsecours.com/heart/heart-healthy-nuts-list/  [10] https://www.sanitarium.com/au/health-nutrition/nutrition/daily-handful-of-nuts-can-keep-your-heart-healthy/  [11] https://www.hsph.harvard.edu/nutritionsource/nuts-for-the-heart/  [12] https://www.trulygoodfoods.com/2020/06/17/heart-healthy-nuts/  [13] https://www.healthline.com/nutrition/9-healthy-nuts |
| **GPT4o Answer 1** | Yes, you can eat nuts on a heart-healthy diet. In fact, nuts are often recommended as part of a heart-healthy diet because they are rich in unsaturated fats, fiber, protein, vitamins, minerals, and antioxidants. These nutrients can help improve heart health by lowering bad cholesterol (LDL) levels, reducing inflammation, and improving blood vessel function. Here are some heart-healthy nuts to consider:  1. Almonds: High in vitamin E, magnesium, and monounsaturated fats.  2. Walnuts: Rich in omega-3 fatty acids, which are beneficial for heart health.  3. Pistachios: Contain phytosterols that help lower cholesterol levels.  4. Cashews: Good source of monounsaturated fats and magnesium.  5. Pecans: Provide antioxidants, fiber, and heart-healthy fats.  6. Hazelnuts: Rich in monounsaturated fats, vitamins, and minerals.  7. Macadamia Nuts: High in monounsaturated fats, which can help improve cholesterol levels.  When incorporating nuts into your diet, it's important to:  - Eat them in moderation, as nuts are calorie-dense.  - Choose unsalted or lightly salted nuts to avoid excess sodium intake.  - Avoid nuts coated in sugar or unhealthy oils.  - Consider portion sizes; a small handful (about 1 ounce or 28 grams) is typically a good serving size.  Including a variety of nuts in your diet can provide a range of nutrients that support heart health.] |
| **GPT4o Answer 2** | Yes, you can eat nuts on a heart-healthy diet. In fact, nuts are often recommended as part of a heart-healthy eating plan due to their many beneficial nutrients. Here are some reasons why nuts can be good for your heart:  1. Healthy Fats: Nuts are rich in unsaturated fats, which can help lower bad cholesterol (LDL) levels and reduce the risk of heart disease.  2. Omega-3 Fatty Acids: Certain nuts, like walnuts, are a good source of omega-3 fatty acids, which are beneficial for heart health.  3. Fiber: Nuts contain dietary fiber, which can help lower cholesterol levels and improve heart health.  4. Antioxidants: Nuts are rich in antioxidants such as vitamin E and selenium, which help protect the body from oxidative stress and inflammation, both of which can contribute to heart disease.  5. Plant Sterols: Some nuts contain plant sterols, substances that can help lower cholesterol.  6. Protein: Nuts provide a good source of plant-based protein, which is beneficial for maintaining overall health and can be a part of a balanced diet.  Tips for Including Nuts in a Heart-Healthy Diet:  - Moderation: While nuts are healthy, they are also calorie-dense. A small handful (about 1 ounce or 28 grams) is typically a reasonable portion size.  - Unsalted and Unflavored: Opt for unsalted and unflavored nuts to avoid excess sodium and added sugars.  - Variety: Include a variety of nuts in your diet to benefit from their different nutrients. Almonds, walnuts, pistachios, pecans, and hazelnuts are all good choices.  - Whole Nuts: Choose whole nuts over nut butters or processed nut products, which can sometimes contain added oils and sugars.  Incorporating nuts into your diet as a snack, adding them to salads, or using them in cooking and baking are great ways to enjoy their health benefits. However, always consult with your healthcare provider or a nutritionist if you have specific dietary concerns or conditions. |
| **GPT4o Answer 3** | Yes, you can eat nuts on a heart-healthy diet. In fact, nuts are considered beneficial for heart health when consumed in moderation. Here are some reasons why nuts are good for your heart:  1. Healthy Fats: Nuts are rich in unsaturated fats, which are healthier than saturated fats and can help reduce bad cholesterol levels (LDL) and increase good cholesterol levels (HDL).  2. Omega-3 Fatty Acids: Some nuts, like walnuts, are high in omega-3 fatty acids, which have been shown to reduce the risk of heart disease.  3. Fiber: Nuts are a good source of dietary fiber, which can help lower cholesterol levels and improve overall heart health.  4. Antioxidants: Nuts contain antioxidants, such as vitamin E, which can help reduce inflammation and oxidative stress in the body, both of which are linked to heart disease.  5. Plant Sterols: Some nuts contain plant sterols, which can help lower cholesterol levels.  6. L-arginine: Nuts are a good source of L-arginine, an amino acid that can help improve the health of your artery walls by making them more flexible and less prone to blood clots.  Despite their benefits, nuts are also high in calories, so it's important to consume them in moderation. A recommended serving size is about a handful (1 ounce or 28 grams) per day. Additionally, opt for unsalted and raw or dry-roasted nuts to avoid added sugars, salts, and unhealthy fats.  If you have specific dietary concerns or conditions, it's always a good idea to consult with a healthcare provider or a registered dietitian for personalized advice. |
| **Llama 3 Answer 1** | Nuts can be a nutritious and delicious addition to a heart-healthy diet. In fact, many studies have shown that eating nuts regularly can help lower the risk of heart disease. Here's why:  1. Rich in healthy fats: Nuts are a good source of monounsaturated and polyunsaturated fats, which can help lower LDL (bad) cholesterol and triglycerides.  2. High in fiber: Nuts are a good source of dietary fiber, which can help lower cholesterol levels and improve blood lipid profiles.  3. Antioxidant-rich: Nuts are packed with antioxidants, such as vitamin E, flavonoids, and polyphenols, which can help protect against oxidative stress and inflammation in the body.  4. May help lower blood pressure: Regular nut consumption has been shown to help lower blood pressure in people with hypertension.  5. Can help with weight management: Nuts are filling and can help with weight loss and maintenance, which is an important factor in heart health.  However, it's important to keep in mind a few things:  1. Choose unsalted and unsweetened nuts: Added salt and sugar can negate the heart-healthy benefits of nuts.  2. Be mindful of portion sizes: While nuts are nutritious, they are high in calories. A serving size of nuts is about 1 ounce, or a small handful (about 23 almonds or 14 walnut halves).  3. Some nuts are higher in calories and fat than others: Macadamia nuts and pecans, for example, are higher in calories and fat than almonds and walnuts.  Here are some heart-healthy nuts to include in your diet:  1. Almonds  2. Walnuts  3. Pecans  4. Hazelnuts  5. Pistachios  6. Cashews (in moderation, as they are higher in sodium)  Remember to always choose unsalted and unsweetened nuts, and enjoy them in moderation as part of a balanced diet. |
| **Llama 3 Answer 2** | Nuts can be a nutritious and healthy addition to a heart-healthy diet, but it's important to consume them in moderation and choose the right types. Here's why:  The Good:  1. Rich in healthy fats: Nuts are a good source of monounsaturated and polyunsaturated fats, which can help lower total cholesterol and LDL ("bad") cholesterol levels.  2. High in antioxidants: Nuts are rich in antioxidants, such as vitamin E and polyphenols, which can help protect against oxidative stress and inflammation in the body.  3. Good source of fiber: Many nuts are a good source of dietary fiber, which can help lower cholesterol levels and promote digestive health.  4. May help with weight management: Nuts are filling and can help with weight management, which is important for heart health.  The Not-So-Good:  1. High in calories: Nuts are high in calories, so overconsumption can lead to weight gain and negate the heart-healthy benefits.  2. Some nuts are high in sodium: While nuts are naturally low in sodium, some varieties (like peanuts) may be high in sodium due to added salt.  3. Possible allergens: Tree nuts, such as walnuts and almonds, are common allergens, so individuals with nut allergies should avoid them.  Choosing the Right Nuts:  1. Almonds: Rich in vitamin E, magnesium, and healthy fats.  2. Walnuts: High in omega-3 fatty acids and antioxidants.  3. Pecans: Good source of antioxidants and fiber.  4. Hazelnuts: Rich in vitamin E and copper.  5. Cashews: Good source of magnesium and copper.  Tips for Incorporating Nuts into a Heart-Healthy Diet:  1. Eat them in moderation: Aim for 1 ounce (28g) or a small handful of nuts per day.  2. Choose unsalted and unsweetened nuts: Avoid added salt and sugar.  3. Pair with fruit or vegetables: Combine nuts with fruit or vegetables to help with portion control and add fiber and antioxidants.  4. Consider nut butters: If you're concerned about whole nuts, consider nut butters like peanut butter or almond butter, but be mindful of added sugars and portion sizes.  In summary, nuts can be a healthy addition to a heart-healthy diet when consumed in moderation, chosen wisely, and paired with a variety of other nutrient-dense foods. |
| **Llama 3 Answer 3** | Nuts are a nutritious and delicious addition to a heart-healthy diet. In fact, numerous studies have shown that eating nuts regularly can help lower the risk of heart disease. Here's why:  1. Rich in healthy fats: Nuts are an excellent source of healthy fats, including monounsaturated and polyunsaturated fats, which can help lower LDL (bad) cholesterol levels and improve overall heart health.  2. Good source of fiber: Nuts are high in dietary fiber, which can help lower cholesterol levels, improve blood sugar control, and promote digestive health.  3. Antioxidant-rich: Nuts are packed with antioxidants, such as vitamin E, flavonoids, and polyphenols, which can help protect against oxidative stress and inflammation in the body, both of which are associated with heart disease.  4. May help lower blood pressure: Regular nut consumption has been shown to help lower blood pressure in people with hypertension.  5. Supports healthy weight: Nuts are filling and can help with weight management, which is essential for heart health.  Some of the best nuts for heart health include:  1. Almonds: Rich in vitamin E, magnesium, and healthy fats.  2. Walnuts: High in alpha-linolenic acid (ALA), a type of omega-3 fatty acid that supports heart health.  3. Pecans: Rich in antioxidants and contain a variety of minerals, including potassium, magnesium, and zinc.  4. Hazelnuts: Good source of vitamin E, copper, and magnesium.  5. Cashews: Rich in magnesium, copper, and zinc.  Remember to enjoy nuts in moderation, as they are high in calories. A serving size of nuts is about 1 ounce, or a small handful (about 23 almonds or 14 walnuts). It's also important to choose unsalted and unsweetened nuts to avoid excess sodium and added sugars.  In summary, nuts can be a great addition to a heart-healthy diet when consumed in moderation and as part of a balanced diet that includes a variety of whole, nutrient-dense foods. |
| **LLaMA3+RAG model Answer 1** | Nuts are indeed a nutritious and tasty addition to a heart-healthy diet. They're packed with protein, fats, fiber, vitamins, minerals, and antioxidants, making them a satisfying snack or ingredient to incorporate into your meals. The American Heart Association recommends opting for healthier choices like almonds, hazelnuts, peanuts, pecans, pistachios, and walnuts.  Walnuts, in particular, stand out for their exceptionally high content of omega-3 fatty acids, similar to those found in oily fish. This makes them an excellent choice for heart health. However, it's essential to remember that nuts are high in calories, so portion control is crucial.  A serving size of nuts is a small handful or 1.5 ounces of whole nuts, equivalent to about 2 tablespoons of nut butter. When selecting nut butters, be sure to compare nutrition labels and choose options with the lowest sodium content and without added tropical oils (palm, coconut) or sugars.  To ensure the freshness and quality of your nuts, store them in the fridge to prevent rancidity. Rancid nuts can be identified by their unpleasant smell and bitter taste, which can ruin an entire dish. So, always taste a nut before using it in a recipe to ensure it's still fresh.  In summary, nuts can be a wonderful addition to a heart-healthy diet when consumed in moderation and chosen wisely. Enjoy your nuts, but remember to keep your portions in check and store them properly to maintain their nutritional benefits.  Sources:  American Heart Association. (2024). Go Nuts, but Just a Little. Retrieved from <https://www.heart.org/en/healthy-living/healthy-eating/eat-smart/fats/go-nuts-but-just-a-little>  Academy of Nutrition and Dietetics. (2020). Nuts and Seeds. Retrieved from <https://www.andjrnl.org/article/S2212-2672(20)30143-8/>  Harvard T.H. Chan School of Public Health. (2024). Nuts and Seeds. Retrieved from <https://www.hsph.harvard.edu/nutritionsource/nuts-and-seeds/> |
| **LLaMA3+RAG model Answer 2** | According to the American Heart Association, nuts are ""petite powerhouses of taste and nutrition"" that are rich in protein, fats, fiber, vitamins, minerals, and antioxidants. In fact, walnuts are particularly high in omega-3 fatty acids, similar to the heart-healthy fat found in oily fish. This makes them an excellent choice for those looking to incorporate more omega-3s into their diet.  The AHA recommends choosing healthier nut options such as almonds, hazelnuts, peanuts, pecans, pistachios, and walnuts. When it comes to nut butters, be sure to compare nutrition labels and opt for those with the lowest amounts of sodium and no added tropical oils (palm, coconut) or sugars.  It's also important to note that a serving size of nuts is a small handful or 1.5 ounces of whole nuts, or 2 tablespoons of nut butter. This is because nuts are high in calories, so moderation is key to maintaining a heart-healthy diet.  Finally, don't forget to store your nuts and nut butters properly to prevent them from going bad. Keep them in the fridge to prevent rancidity, which can result in an unpleasant smell and bitter taste.  In summary, nuts can be a wonderful addition to a heart-healthy diet when consumed in moderation and as part of a balanced diet. So go ahead, grab a small handful of your favorite nuts and enjoy!  Sources:  American Heart Association. (2024). Go Nuts, But Just a Little. Retrieved from <https://www.heart.org/en/healthy-living/healthy-eating/eat-smart/fats/go-nuts-but-just-a-little>  Academy of Nutrition and Dietetics. (2020). Nuts and Seeds. Retrieved from <https://www.andjrnl.org/article/S2212-2672(20)30123-6/>  Harvard T.H. Chan School of Public Health. (2024). Nuts and Seeds. Retrieved from <https://www.hsph.harvard.edu/nutritionsource/nuts-and-seeds/ |
| **LLaMA3+RAG model Answer 3** | According to the American Heart Association, nuts are ""petite powerhouses of taste and nutrition"" that offer a wealth of benefits. They're packed with protein, fats, fiber, vitamins, minerals, and antioxidants, making them a nutritious snack or addition to meals. Some nuts, like walnuts, are particularly high in omega-3 fatty acids, similar to the heart-healthy fat found in oily fish. This makes them an excellent choice for those looking to boost their heart health.  The AHA recommends choosing healthier nut options, such as almonds, hazelnuts, peanuts, pecans, pistachios, and walnuts. When selecting nut butters, be sure to compare nutrition labels and opt for those with the lowest amounts of sodium and no added tropical oils (like palm or coconut oil) or sugars.  Now, about that serving size: a small handful or 1.5 ounces of whole nuts or 2 tablespoons of nut butter is the recommended daily amount. This will help you reap the benefits of nuts while keeping your calorie intake in check.  One important note: nuts can go bad (rancid) over time, so be sure to store them in the fridge to maintain their freshness. If you're using nuts in a recipe, take a moment to taste one to ensure it's still fresh – you wouldn't want to ruin the whole dish with a rancid nut!  In summary, nuts are a heart-healthy choice when consumed in moderation and as part of a balanced diet. So go ahead, indulge in that small handful of nuts or spread some nut butter on your favorite fruit or veggie – your heart (and taste buds) will thank you!  Sources:  American Heart Association. (2024). Go Nuts, But Just a Little. Retrieved from <https://www.heart.org/en/healthy-living/healthy-eating/eat-smart/fats/go-nuts-but-just-a-little>  Harvard T.H. Chan School of Public Health. (2024). Nuts and Seeds. Retrieved from <https://www.hsph.harvard.edu/nutritionsource/what-should-you-eat/nuts-and-seeds |
| **Question 30** | **How much fish should I be eating on a heart healthy diet?** |
| **Perplexity Answer 1** | To maintain a heart-healthy diet, the American Heart Association recommends eating at least two 3.5-ounce servings of non-fried fish per week[4]. This equates to about 3/4 cup of flaked fish[4].  Eating fish, especially oily fish high in omega-3 fatty acids, provides numerous cardiovascular benefits[1][3][4]:  - Reduces risk of dying from heart disease by 36% when eating about 1-2 servings per week[3]  - Protects against abnormal heart rhythms, decreases triglyceride levels, and slows growth of fatty deposits in arteries[4]  - Lowers blood pressure and heart rate, improves blood vessel function[3]  Some of the healthiest fish high in omega-3s include[1][2][4]:  - Salmon  - Mackerel  - Herring  - Sardines  - Albacore tuna  Other good options are[2]:  - Atlantic mackerel  - Canned salmon  - Sardines  - Trout  - Herring  The benefits of eating fish regularly far outweigh any potential risks from mercury or contaminants for most adults[3][4]. However, pregnant women and young children should avoid high-mercury fish like shark and swordfish[3].  For optimal heart health, aim for a variety of fish 2-3 times per week as part of an overall healthy eating pattern[1][4]. Substituting fish for higher-fat meats can make a big difference[4].  Citations:  [1] https://www.heartfoundation.org.au/blog/not-eating-enough-fish  [2] https://www.eatingwell.com/article/69763/5-of-the-healthiest-fish-to-eat-and-5-to-avoid/  [3] https://www.hsph.harvard.edu/nutritionsource/fish/  [4] https://www.heart.org/en/news/2018/05/25/eating-fish-twice-a-week-reduces-heart-stroke-risk  [5] https://www.seafoodnutrition.org/seafood-101/nutrition/celebrate-heart-month-by-eating-seafood-twice-a-week/ |
| **Perplexity Answer 2** | To maintain a heart-healthy diet, the American Heart Association recommends eating at least two 3.5-ounce servings of non-fried fish per week[4]. This equates to about 3/4 cup of flaked fish[4].  Eating fish, especially oily fish high in omega-3 fatty acids, provides numerous cardiovascular benefits[1][3][4]:  - Reduces risk of dying from heart disease by 36% when eating about 1-2 servings per week[3]  - Protects against abnormal heart rhythms that can lead to sudden death[4]  - Decreases triglyceride levels and slows growth of fatty deposits in arteries[4]  - May reduce risk of stroke, depression, Alzheimer's disease and other chronic conditions[3]  The healthiest fish to eat are those high in omega-3s like salmon, mackerel, herring, lake trout, sardines and albacore tuna[2][4]. Eating a variety of fish is recommended to get the full benefits[4].  Some types of fish like shark and swordfish should be limited due to higher mercury levels, but for most adults the benefits of eating fish far outweigh any risks from contaminants[3][4]. Choosing sustainable seafood options is also important[2].  In summary, aim for at least two servings of non-fried, omega-3 rich fish per week as part of an overall heart-healthy diet. Substituting fish for higher saturated fat meats can provide a big boost to cardiovascular health[4].  Citations:  [1] https://www.heartfoundation.org.au/blog/not-eating-enough-fish  [2] https://www.eatingwell.com/article/69763/5-of-the-healthiest-fish-to-eat-and-5-to-avoid/  [3] https://www.hsph.harvard.edu/nutritionsource/fish/  [4] https://www.heart.org/en/news/2018/05/25/eating-fish-twice-a-week-reduces-heart-stroke-risk  [5] https://www.seafoodnutrition.org/seafood-101/nutrition/celebrate-heart-month-by-eating-seafood-twice-a-week/ |
| **Perplexity Answer 3** | To maintain a heart-healthy diet, the American Heart Association recommends eating at least two 3.5-ounce servings of non-fried fish per week[4]. This equates to about 3/4 cup of flaked fish[4].5  Eating fish, especially oily fish high in omega-3 fatty acids like salmon, mackerel, herring, lake trout, sardines and albacore tuna, provides many cardiovascular benefits[1][3][4]. Omega-3s help reduce the risk of abnormal heart rhythms, decrease triglyceride levels, and slow the growth of plaque in the arteries[4].  Eating fish once or twice a week may also reduce the risk of stroke, depression, Alzheimer's disease, and other chronic conditions[3]. The omega-3 fatty acids in fish are important for heart and brain health, and our bodies do not produce them naturally[1].  While some types of fish like shark and swordfish contain mercury, for most adults the benefits of eating fish far outweigh any potential risks[4]. Eating a variety of fish regularly is ideal to get the full heart-healthy benefits[4].  In summary, aim for at least two 3.5-ounce servings of non-fried, omega-3 rich fish per week as part of an overall heart-healthy diet. Choosing a variety of fish like salmon, mackerel, herring and sardines is recommended[1][4].  Citations:  [1] https://www.heartfoundation.org.au/blog/not-eating-enough-fish  [2] https://www.eatingwell.com/article/69763/5-of-the-healthiest-fish-to-eat-and-5-to-avoid/  [3] https://www.hsph.harvard.edu/nutritionsource/fish/  [4] https://www.heart.org/en/news/2018/05/25/eating-fish-twice-a-week-reduces-heart-stroke-risk  [5] https://www.seafoodnutrition.org/seafood-101/nutrition/celebrate-heart-month-by-eating-seafood-twice-a-week/ |
| **GPT4o Answer 1** | For a heart-healthy diet, it is recommended to consume fish, particularly fatty fish, due to their high omega-3 fatty acid content, which is beneficial for cardiovascular health. According to guidelines from the American Heart Association (AHA), you should aim to eat fish at least two times (two servings) per week. Each serving is typically about 3.5 ounces of cooked fish or about ¾ cup of flaked fish.  Here are a few tips to keep in mind:  1. Choose Fatty Fish: Focus on fatty fish that are high in omega-3 fatty acids. Examples include salmon, mackerel, sardines, albacore tuna, and trout.  2. Preparation Methods: Opt for baking, grilling, or steaming fish rather than frying to keep the meal heart-healthy. Avoid using high-calorie sauces or breading.  3. Variety: While fatty fish are recommended, varying your choices can help provide a range of nutrients. Including other types of seafood like shellfish, shrimp, and light canned tuna can also be beneficial.  4. Sustainability: Consider the sustainability and mercury content of the fish you consume. Some fish may contain higher levels of mercury, which can be harmful in large amounts. Examples of fish lower in mercury include salmon, sardines, and trout.  In summary, aim for at least two servings of fish per week, focusing on fatty fish prepared in healthy ways, to support a heart-healthy diet. |
| **GPT4o Answer 2** | For a heart-healthy diet, it's generally recommended to eat fish, particularly fatty fish, at least twice a week. Fatty fish, such as salmon, mackerel, sardines, trout, and albacore tuna, are rich in omega-3 fatty acids, which are beneficial for heart health.  Here's a more detailed breakdown:  1. Frequency: Aim for at least two servings of fish per week.  2. Serving Size: A serving size is typically about 3.5 ounces (100 grams) of cooked fish or about ¾ cup of flaked fish.  3. Type of Fish: Prioritize fatty fish that are high in omega-3 fatty acids. These include:  - Salmon  - Mackerel  - Sardines  - Trout  - Albacore tuna  Additional Tips:  - Variety: Include a variety of fish to avoid potential exposure to contaminants found in some fish species.  - Cooking Methods: Opt for healthy cooking methods such as baking, grilling, or steaming instead of frying to keep the dishes heart-healthy.  - Supplementation: If you don't like fish or have dietary restrictions, consider omega-3 supplements, but consult with a healthcare provider first.  Incorporating these guidelines can help support cardiovascular health and provide other nutritional benefits. |
| **GPT4o Answer 3** | For a heart-healthy diet, it's recommended to consume fish, especially fatty fish, which are high in omega-3 fatty acids. The general guideline is:  - At least two servings of fish per week: A serving is typically 3.5 ounces (about 100 grams) of cooked fish or about 3/4 cup of flaked fish.  Fatty fish, such as salmon, mackerel, sardines, albacore tuna, and lake trout, are particularly beneficial for heart health because they are rich in omega-3 fatty acids, which can help reduce inflammation, lower blood pressure, decrease triglycerides, and reduce the risk of arrhythmias.  If you have specific health concerns or dietary needs, it's always best to consult with a healthcare provider or a nutritionist for personalized advice. |
| **Llama 3 Answer 1** | Fish is an excellent addition to a heart-healthy diet, thanks to its high levels of omega-3 fatty acids, protein, and various nutrients. The American Heart Association (AHA) recommends eating fish at least two times a week, with a serving size of about 3.5 ounces or 100 grams.  Here are some guidelines to help you incorporate fish into your heart-healthy diet:  1. Fatty fish: Focus on fatty fish, which are rich in omega-3s. These include:  * Salmon (wild-caught Alaskan or Pacific salmon is a good choice)  * Sardines  * Mackerel  * Tuna (look for "light" or "skipjack" tuna, which is lower in mercury)  * Trout  2. Servings per week: Aim for 2-3 servings of fatty fish per week. A serving size is about 3.5 ounces or 100 grams, which is roughly the size of a deck of cards.  3. Variety is key: Mix up your fish choices to minimize exposure to mercury, a toxic substance found in some fish. Vary your fatty fish intake and include some lower-mercury options like:  * Shrimp  * Catfish  * Pollock  * Cod  4. Avoid high-mercury fish: Limit or avoid fish with high mercury levels, such as:  * Shark  * Swordfish  * King mackerel  * Tilefish  5. Farmed vs. wild-caught: While both farmed and wild-caught fish can be part of a heart-healthy diet, wild-caught fish tend to have higher levels of omega-3s and lower levels of contaminants like PCBs (polychlorinated biphenyls).  6. Cooking methods: Grilling, baking, or broiling fish are healthier cooking methods than frying, which can increase the calorie and fat content of your meal.  7. Supplements: If you're not a fan of fish or have trouble incorporating it into your diet, consider consulting with your healthcare provider about omega-3 supplements.  Remember to always choose fresh, sustainable, and responsibly sourced fish to minimize your environmental impact and ensure food safety.  Enjoy your fish-filled heart-healthy diet! |
| **Llama 3 Answer 2** | Eating fish as part of a heart-healthy diet is an excellent choice! Fish is an excellent source of protein, omega-3 fatty acids, and various nutrients that can help support heart health. Here's what you need to know:  Recommended intake:  The American Heart Association (AHA) recommends eating fatty fish at least two times a week, with a serving size of 3.5 ounces (about the size of a deck of cards) per serving. Fatty fish are high in omega-3 fatty acids, which are particularly beneficial for heart health.  Types of fish to focus on:  Choose fatty fish that are high in omega-3s, such as:  1. Salmon (wild-caught Alaskan or Pacific salmon is best)  2. Sardines  3. Mackerel  4. Tuna (look for skipjack or albacore)  5. Trout  Other fish options:  While not as high in omega-3s, these fish are still a good choice:  1. Cod  2. Tilapia  3. Catfish  4. Shrimp  5. Scallops  Tips for incorporating fish into your diet:  1. Variety is key: Mix up your fish choices to minimize exposure to mercury and other contaminants.  2. Choose wild-caught: Wild-caught fish tend to have higher omega-3 levels and lower mercury levels than farmed fish.  3. Avoid high-mercury fish: Limit or avoid fish with high mercury levels, such as shark, swordfish, king mackerel, and tilefish.  4. Cooking methods matter: Grilling, baking, or broiling fish can help retain omega-3s and other nutrients. Avoid frying, as it can increase saturated fat intake.  5. Consider supplements: If you're not a fan of fish or have trouble getting enough omega-3s from your diet, consult with your healthcare provider about fish oil supplements.  Additional heart-healthy diet tips:  1. Eat a balanced diet: Focus on whole, unprocessed foods like fruits, vegetables, whole grains, lean proteins, and healthy fats.  2. Limit saturated and trans fats: Opt for healthier fats like avocado, nuts, and olive oil.  3. Stay hydrated: Drink plenty of water and limit sugary drinks.  4. Maintain a healthy weight: Aim for a body mass index (BMI) between 18.5 and 24.9.  5. Exercise regularly: Aim for at least 150 minutes of moderate-intensity aerobic exercise or 75 minutes of vigorous-intensity aerobic exercise per week.  By following these guidelines, you'll be well on your way to a heart-healthy diet that includes a balanced intake of fish and other nutrient-dense foods. |
| **Llama 3 Answer 3** | Eating fish as part of a heart-healthy diet is an excellent choice! Fatty fish, in particular, are rich in omega-3 fatty acids, which have been shown to lower triglycerides, reduce blood pressure, and prevent blood clots. Here's a guide to help you navigate the recommended amount of fish to include in your diet:  American Heart Association (AHA) Recommendations:  * Eat fatty fish at least 2 times a week, with a minimum of 8 ounces (225g) per week.  * Fatty fish like salmon, mackerel, sardines, and albacore tuna are high in omega-3s.  * If you have heart disease or high triglycerides, your doctor may recommend higher doses of omega-3s, up to 1 gram (1000mg) per day.  General Guidelines:  * Aim for 2-3 servings of fish per week, with a serving size of 3 ounces (85g) cooked or about the size of a deck of cards.  * Vary your fish intake to minimize exposure to mercury and other contaminants. Choose a mix of fatty and lean fish.  Fish Options:  * Fatty fish (high in omega-3s):  + Salmon (wild-caught Alaskan or Pacific)  + Mackerel  + Sardines  + Albacore tuna  + Trout  * Lean fish (lower in omega-3s, but still heart-healthy):  + Cod  + Tilapia  + Catfish  + Pollock  + Shrimp  Important Considerations:  Pregnant women, breastfeeding mothers, and young children should avoid high-mercury fish like shark, swordfish, king mackerel, and tilefish.  If you have a fish allergy or are concerned about contaminants, consider omega-3 supplements after consulting with your healthcare provider.  Remember to always choose wild-caught, sustainably sourced fish whenever possible, and vary your fish intake to minimize exposure to contaminants. Enjoy your heart-healthy fish dishes! |
| **LLaMA3+RAG model Answer 1** | According to the American Heart Association, it is recommended to eat fish at least twice a week, with a serving size of 3 ounces cooked or about ¾ cup of flaked fish. This translates to a recommended 2-3 servings of fish per week, with an emphasis on fatty fish high in omega-3 fatty acids.  To incorporate more fish into your diet, you can try adding it to salads, sandwiches, or eating it as a snack. You can also experiment with different cooking methods such as grilling, steaming, barbequing, poaching, baking, or pan-frying using healthier oils like olive oil, canola oil, sunflower oil, or peanut oil.  It's also important to note that some types of fish contain high levels of mercury or other environmental contaminants, so it's essential to check the FDA website for the latest advisories, especially for pregnant women, nursing mothers, and young children.  Sources:  American Heart Association. (2024). Fish and Omega-3 Fatty Acids. Retrieved from <https://www.heart.org/en/healthy-living/healthy-eating/eat-smart/fats/fish-and-omega-3-fatty-acids>  Heart Foundation. (2024). Not Eating Enough Fish? Retrieved from <https://www.heartfoundation.org.au/blog/not-eating-enough-fish>  Additional sources:  U.S. Food and Drug Administration. (2024). Fish: What Pregnant Women and Parents Should Know. Retrieved from <https://www.fda.gov/food/consumers/fish-what-pregnant-women-and-parents-should-know>" |
| **LLaMA3+RAG model Answer 2** | According to the American Heart Association, it is recommended to eat fish at least twice a week, with a serving size of 3 ounces cooked, or about ¾ cup of flaked fish. This translates to a total of 2 servings of fish per week, with an emphasis on fatty fish that are high in omega-3 fatty acids.  To break it down further, the recommended intake is:  2 servings of fish per week  1 serving = 3 ounces cooked, or about ¾ cup of flaked fish  Fatty fish, such as anchovies, herring, mackerel, black cod, salmon, sardines, bluefin tuna, whitefish, striped bass, and cobia, are high in omega-3 fatty acids and should be prioritized  Eating a variety of fish will help minimize any potentially adverse effects due to environmental pollutants  It's also important to note that the American Heart Association recommends a dietary pattern that includes healthy sources of proteins, mostly from plant sources, and regularly eating fish and seafood. Additionally, it's essential to choose healthier cooking methods, such as grilling, steaming, barbequing, poaching, baking, or pan-frying, and to use healthier oils like olive oil, canola oil, sunflower oil, or peanut oil.  Sources:  American Heart Association. (2024). Fish and Omega-3 Fatty Acids. Retrieved from <https://www.heart.org/en/healthy-living/healthy-eating/eat-smart/fats/fish-and-omega-3-fatty-acids>  Heart Foundation. (2024). Not Eating Enough Fish? Retrieved from <https://www.heartfoundation.org.au/blog/not-eating-enough-fish> |
| **LLaMA3+RAG model Answer 3** | According to the American Heart Association, it is recommended to eat fish at least twice a week, with a serving size of 3 ounces cooked or about ¾ cup of flaked fish. This translates to a recommended 2-3 servings of fish per week. Fatty fish, such as anchovies, herring, mackerel, black cod, salmon, sardines, bluefin tuna, whitefish, striped bass, and cobia, are high in omega-3 fatty acids and should be included in your diet.  To incorporate more fish into your diet, you can try adding a tin of unflavored tuna or salmon to a healthy salad, sandwich, or eating it as a snack. You can also add seafood marinara mixes to stir-fries and pasta, or bake or steam fish parcels for a quick and tasty weekday dinner. Additionally, you can choose healthier cooking methods such as grilling, steaming, barbequing, poaching, baking, or pan-frying, and use healthier oils like olive oil, canola oil, sunflower oil, or peanut oil.  It's also important to note that pregnant women, babies, and young children should check the U.S. Food and Drug Administration (FDA) website for the latest advisories on fish consumption to avoid eating contaminated fish.  Sources:  American Heart Association. (2024). Fish and Omega-3 Fatty Acids. Retrieved from <https://www.heart.org/en/healthy-living/healthy-eating/eat-smart/fats/fish-and-omega-3-fatty-acids>  Heart Foundation. (2024). Not eating enough fish? Retrieved from <https://www.heartfoundation.org.au/blog/not-eating-enough-fish>  Note: The recommended serving size and frequency of fish consumption may vary depending on individual circumstances, such as pregnancy or specific health conditions. It's always best to consult with a healthcare professional or registered dietitian for personalized nutrition advice. |
